# ﻿An annotated plant checklist of the transboundary volcanic Mt Elgon, East Africa

**DOI:** 10.3897/phytokeys.223.97401

**Published:** 2023-04-04

**Authors:** Peninah Cheptoo Rono, Fredrick Munyao Mutie, Vivian Kathambi, Neng Wei, Benjamin Muema Watuma, Consolata Nanjala, Godfrey Kinyori Wagutu, Paul M. Kirika, Itambo Malombe, Guang-Wan Hu, Qing-Feng Wang

**Affiliations:** 1 CAS Key Laboratory of Plant Germplasm Enhancement and Specialty Agriculture, Wuhan Botanical Garden, Chinese Academy of Sciences, Wuhan 430074, Hubei, China University of Chinese Academy of Sciences Beijing China; 2 University of Chinese Academy of Sciences, Beijing 100049, China CAS Key Laboratory of Plant Germplasm Enhancement and Specialty Agriculture, Wuhan Botanical Garden, Chinese Academy of Sciences Wuhan China; 3 Sino-Africa Joint Research Center, Chinese Academy of Sciences, Wuhan 430074, Hubei, China Sino-Africa Joint Research Center, Chinese Academy of Sciences Wuhan China; 4 East African Herbarium, National Museums of Kenya, P.O. Box 45166 00100, Nairobi, Kenya East African Herbarium, National Museums of Kenya Nairobi Kenya; 5 Key Laboratory of Aquatic Botany and Watershed Ecology, Wuhan Botanical Garden, ChineseAcademy of Sciences, Wuhan, China Key Laboratory of Aquatic Botany and Watershed Ecology, Wuhan Botanical Garden, ChineseAcademy of Sciences Wuhan China

**Keywords:** Checklist, conservation, distribution, Mt Elgon, plant species

## Abstract

Mt Elgon is an ancient transboundary volcanic mountain found at the Kenya-Uganda boarder possessing high plant diversity. This study documents an updated checklist of the mountain’s vascular plants obtained through random-walk field excursions and retrieval of herbarium specimen tracing back to 1900. We compiled 1709 species from 673 genera in 131 families. One new species of the family Cucurbitaceae was also reported. This checklist records respective habitat, habits, elevation ranges, voucher numbers and global distribution ranges of each species. Native and exotic species were also distinguished, where 8.4% of the total species in 49 families were exotic species. There were 103 endemic species, while 14 species were found to be both rare and endemic. IUCN conservation status revealed 2 Critically Endangered, 4 Endangered, 9 Vulnerable and 2 Near Threatened species. This study presents the first and most comprehensive plant inventory of Mt Elgon that will facilitate further ecological and phylogenetic studies.

## ﻿Introduction

Plants form part of the most fundamental natural resources offered by nature. They provide essential requirements for life including fuel, food, medicine, fodder, timber, oils, and resins (Price 1998; Muhweezi et al. 2007; Okello et al. 2010; Mutie et al. 2020). Plants’ communities also play a key role in maintaining the general biodiversity (flora and fauna) as well as being significant in environmental conservation necessary for attaining sustainable environmental management (Mutie et al. 2020b). The first step in effective conservation and sustainable use of natural resources is acknowledging their existence and distribution (Gaston 2000; Sosef et al. 2017). Researchers across the world have been keen to conduct surveys, collect samples and store the acquired data (where and when the specimen was seen and collected) and a physical proof of the plant in the form of dried specimens in different herbaria of the world. Herbaria are major physical storage facilities of essential primary plant data in dried form or in a liquid such as alcohol (Bebber et al. 2010). So far, over 350,000,000 specimens have been curated and preserved in various herbaria (Sosef et al. 2017). Plant checklists offer foundational data in analysing ecological, evolutionary and biogeographical hypothesis used to determine land use management and environmental assessment policies governing biodiversity (Zhou et al. 2018). This is evident in the existence of a plethora of biodiversity databases hosting thousands of museum records and observations of species made during field excursions (Boyle et al. 2013). Mistakes during the compilation of checklists may trigger continuous environmental deterioration as a result of erroneous biodiversity policy conclusions. Therefore, conducting floristic surveys and documenting checklists is important in understanding these natural resources for economic development in both developed and developing countries.

Mountains, estimated to be 8,616 in number globally, are among the unique and diverse physical features of the world found in all continents, accounting for about 20–24% of the terrestrial surface area (Beniston 2000; Körner et al. 2006; Snethlage et al. 2022). Compared to flat lands, montane regions have rich biodiversity with high rates of species extinction and speciation, as well as regions of low immigration rates (Jacob et al. 2015; Lomolino 2016). These mountain ecosystems are also hotspots of biodiversity harboring rare and endemic species (Lomolino 2001; Kessler and Kluge 2008; Jacob et al. 2015). Field excursions and ecological studies have revealed that the high plant diversity found in mountains can be attributed to the different vegetation stratification, brief ecotones, enormous beta diversity, dynamic environmental factors, isolation, and climatic variation within a limited geographical range as result of altitudinal differences over a short horizontal distance (Hamilton 1975; Crausbay and Martin 2016). Furthermore, mountains are, in part, determinant factors of the floristic composition, economic activities, social activities, and climatic condition of the surrounding regions (Price 1998; Wu et al. 2020).

African mountains host species rich regions of the world. Several biodiversity hotspots have been identified within the African tropics including the eastern Afromontane biodiversity hotspot, the Eastern Arc Mountains, and the Coastal Forest Ecosystem (Myers et al. 2000; CEPF 2012). In East Africa, the majority of these mountains under the Eastern Afromontane biodiversity hotspot are of volcanic origin, including Mt Elgon, Meru, Kenya, Kilimanjaro, and Abardare ranges (CEPF 2012). However, their diversity is at high risk of extinction due to overexploitation, climate change, and habitat destruction (Stévart et al. 2019). Several floristic studies have been recently reported on mountains with the aim of formulating priority conservation polices (Mbuni et al. 2019; Kipkoech et al. 2020; Watuma et al. 2022; Zhou et al. 2022). However, transboundary mountains have encountered prolonged research barriers emanating from ethical and political disputes (Roga 2017). In East Africa, an example of a transboundary mountain that experienced conflicts is Mt Elgon (Petursson and Vedeld 2015). Several transboundary cooperations have been formulated and successfully mitigated cross-country disputes (Roga 2017). Mt Elgon was categorized as a Biosphere reserve in 2003 on the Kenyan side and in 2009 as a man and biosphere reserve for the Ugandan side by the United Nations Educational, Scientific and Cultural organization (UNESCO), because of its cultural significance, considerable plant diversity comprising of rare and endemic species of Afromontane flora, and its role as a water catchment area with more than two million people relying on it for livelihood (UWA 2000). However, depletion of its natural resources by local communities, wild fire caused by poachers, and encroachment to expand agricultural land has been escalating over time (Roga 2017). In addition, human conflicts have frequently been reported from the Kenyan side of Mt Elgon. For instance, conflicts intensified between the years 2006–2008 with the formation Sabaot Land Defense Forces that resulted in the death of more than 600 people, which consequently limited scientific exploration (Simiyu 2008).

Mt Elgon is among the oldest floristic regions in East Africa tracing back to about 22 million years ago and possesses the world’s largest intact caldera (UWA 2000). It forms part of the lake Victorian regional mosaic, which stretches through Rwanda, Uganda, Burundi and portions of Tanzania and Congo (Mukhwana 2002). Initial floristic studies on the mountain trace back to the 1910s with a study on the crater vegetation (Dummer 1919). Further explorations were done in the 1930s where 649 species were collected of which 32 were new species by Bullock (1932; 1933). Since then, the fascinating flora of Mt Elgon has been vastly studied by a number of botanists (Dale 1940; Hedberg 1955, 1957, 1964; Langdale-Brown et al. 1964; Synnott 1968; Tweedie and Agnew 1970; Hamilton 1975; Tweedie 1976; Hamilton and Perrott 1981; Beck et al. 1987; van Heist 1994; Davenport et al. 1996; Reed and Clokie 2000; Wesche 2002; Bakamwesiga et al. 2005; Russell et al. 2017). Significantly, Tweedie and Agnew (1970) reviewed Lugard’s initial plant samples of Mt Elgon, Dale in 1940 studied the forest types, while Tweedie (1976) classified plants according to their different habits. Hamilton and Perrott (1981) studied the altitudinal vegetation zones; van Heist in 1994 produced an aerial map; Reed and Clokie (2000) documented the effects of grazing and cultivation on forest communities; and Wesche in 2002 recorded the different plant communities of Mt Elgon. Okello et al. (2010) reported the ethno-botanical significance of Mt Elgon species to the Sabaoti community, while Russell et al. (2017) documented the forest trends and implication of governance regimes. Wanyama et al. (2020) implored remote sensing techniques to evaluate the vegetation cover of Mt Elgon, reporting cases of browning and greening with key drivers being human activities and natural occurrences. Masayi et al. (2021) studied forest degradation in Mt Elgon. This mountain ecosystem has been of interest due to its old age and physical isolation from other high montane regions despite conflicts emanating from its transboundary location between two countries (Kenya and Uganda). These studies all agree that Mt Elgon hosts a unique ecosystem that forms part of the African Afromontane centre of endemism. However, the number of species reported in this ecosystem has been changing over time (Bakamwesiga et al. 2005; MUIENR and NMK 2005).

The absence of a comprehensive up-to-date checklist hampers priority species conservation and implementation of biodiversity management policies in Mt Elgon. The present study aims to fill the knowledge gap by documenting an elaborate plant species checklist of Mt Elgon that: (i) distinguishes between native and exotic plants and their distribution ranges; (ii) evaluates the conservation status of extant taxa; and (iii) identifies rare and endemic species found in Mt Elgon. This study is fundamental in further evaluating phylogenetic diversity and ecological studies. This will be significant in formulating and implementation of conservation policies for the ecosystems of Mt Elgon.

## ﻿Materials and methods

### ﻿Study site

**Figure 1.**
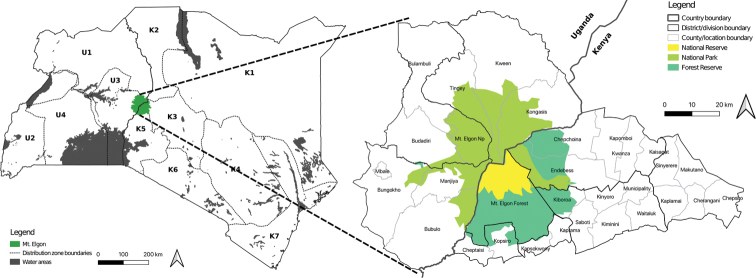
Floristic divisions of Kenya and Uganda and the location of Mt Elgon.

Mt Elgon is an extinct, solitary, transboundary volcanic mountain at the Kenya and Uganda border. It is located at 1°08'N, 34°45'E (Fig. 1). In the Kenyan side, it stands on Mt Elgon and Trans-Nzoia Counties on the western part of the country, whereas it lies in two provinces in Uganda, Kapchorwa and Mbale, on the Eastern side of the country (Fig. 1). It covers about 112,898 ha of land, which is the largest base area among East Africa volcanic mountains, stretching about 80 km from north to south and about 50 km from east to west (Muhweezi et al. 2007). It has an 8 km wide crater at the top and rises to a height of 4321 m asl with a gentle slope angle of less than 4° on average. Mt Elgon is believed to have once been the highest peak in Africa, but due to volcanic rock weathering, it is now the fourth highest mountain in East Africa and the eight highest in Africa. Significantly, it is the oldest among the East African volcanic mountains on the East African peneplain formed during the Miocene epoch about 24 million years ago (Lippard 1973; Baker et al. 1971).

The climate of Mt Elgon is characterized by two alternating seasonal air streams. The moist southwesterly and the dry northeasterly air streams provide it a bimodal pattern of rainfall (Okello et al. 2010; Obiri 2014). The months between April and October are the wettest with short dry seasons in June and July. The driest season occurs from November to February. Generally, the mountain receives an annual rainfall of 2700 mm, which is higher to the southern side (that receives about 2000 mm of precipitation annually) than to the northern side that records an annual precipitation of 1500 mm (Bussmann 2006). Maximum rainfall is experienced in the forest zone in the lower and mid altitudes. In the higher altitudes, rain falls in the form of drizzle rather than heavy downpours. Temperature ranges vary between 27–30 °C at the higher limit and 15–17 °C at the lower limit. As the altitude increases, the climatic conditions become more temperate and less seasonal. Distinctively in the alpine zone, temperatures drop to as low as 0 °C. Mt Elgon experiences wet and humid conditions throughout the year predicting a rich composition of woody climbers and epiphytes (Hitimana et al. 2004; Okello et al. 2010; Jiang et al. 2014). It is divided into vegetation zones including (i) mixed montane forests up to 2500 m, (ii) bamboo and low canopy montane forest at 2500–3000 m asl, (iii) high montane heath at 3000–3500 m, and (iv) moorland above 3500 m to the peak (Dale 1940; Howard 1991; van Heist 1994).

In 1932, Mt Elgon forest was gazetted as a montane forest, under the Ministry of Environment & Natural Resources (MENR). The forest covers 73,705 ha while that within the national park covers 16,900 ha on the northeastern slope of the mountain managed by the Kenya Wildlife Service (Petursson et al. 2013). On the Ugandan side, Mt Elgon forest is exclusively a National Park covering approximately 1,121 km^2^ having a park boundary of 211 km long (Sassen and Sheil 2013). The park is bordered by eight districts, making human encroachment into the forest ecosystem inevitable. In 1993, Mt Elgon in Uganda was re-assigned to being a National Park from a National Reserve formerly designated and gazetted in 1929.

### ﻿Data collection

We assembled an inclusive database of plants of Mt Elgon from field excursions tracing back to the 1900s. The Sino-Africa Joint Research Centre in collaboration with the National Museums of Kenya conducted field excursions between the year 2017 and 2019 in the vast Mt Elgon ecosystem. Plant specimen assembling were done using the random-walk survey where fertile plants sample (in fruiting or flowering stages) were collected in triplicates. Onsite plants and habitat characteristics were recorded and specimen voucher numbers assigned alongside scientific and/or local names, location, and collection date. Specimens were preserved by pressing and drying. Assigned scientific names were later confirmed using standard references (FTEA editors 1952–2012; Blundell 1987; Agnew and Agnew 1994; Agnew 2013). The identified and labelled herbarium specimens were stored at the East African herbarium in Kenya (EA) and duplicates transferred to the Hubei Institute of Botany herbarium (HIB), in China.

For inclusive tabulation of vascular plants of Mt Elgon, previous plant collections dating back to 1900 were retrieved from various online herbaria, field guides and published floras, such as Kenya Trees Shrubs and Lianas (Beentje 1994), Wild Flowers of East Africa (Blundell 1987), Flora of Tropical East Africa (FTEA editors 1952–2012), and Upland Kenya Wild Flowers and Ferns (Agnew 2013), as well as checklists (Bullock 1932, 1933; Tweedie 1976). We also retrieved information on habitat, altitudinal distribution ranges, collectors and voucher specimen numbers to augment our collections. In the cases of unavailable vouchers, descriptive monographs were cited. Corresponding herbaria hosting voucher specimens were indicated by their respective acronyms according to the Index Herbariorum (http://sweetgum.nybg.org/science/ih/). Distribution ranges of each taxon were extrapolated to be within the maximum and minimum possible record in relation to altitudinal ranges of Mt Elgon. Based on our collections, life forms were assigned as herbs, shrubs, climbers, lianas and trees following Agnew (2013) and Beentje (2016). Herbarium and additional collections from checklists and monography were categorized into different lifeforms based on standard references (FTEA editors 1952–2012; Blundell 1987; Agnew and Agnew 1994; Agnew 2013). Scientific name circumscription, classes and families of all assembled taxa were checked against the International Plant Name Index (IPNI 2022) (https://www.ipni.org/), Plants of the World Online (POWO 2022) (https://powo.science.kew.org), and Tropicos (Tropicos 2022) (https://www.tropicos.org).

Conservation status of each taxon were assigned as either Critically Endangered (CR), Endangered (EN), Vulnerable (VU) or Near Threatened (NT) based on International Union for Conservation of Nature criterion (IUCN) Red list of threatened species (IUCN 2021) (https://www.iucnredlist.org). In addition, taxa endemism was determined based on literature citation and online distribution ranges according to the Global Biodiverity Information Facility (https://www.gbif.org). We also inferred whether the taxa compiled was native or exotic.

## ﻿Results

### ﻿Species composition

**Figure 2.**
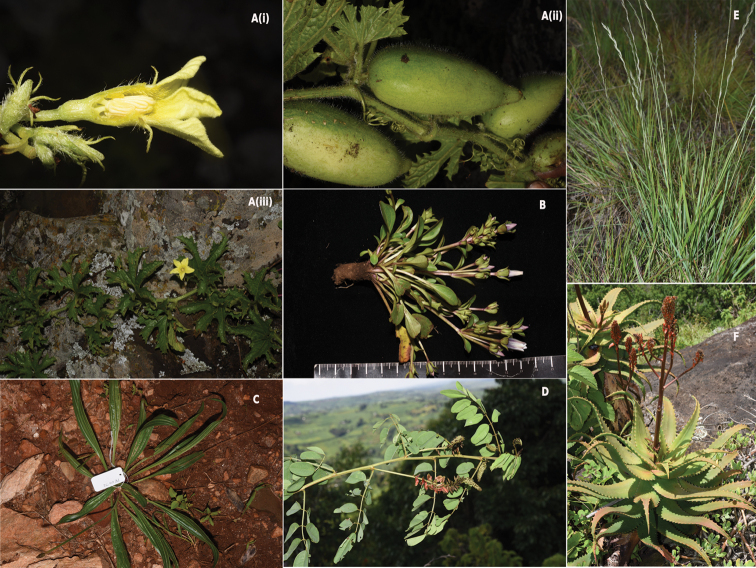
Photographs of specimens collected from Mt Elgon **A** (**i,ii,iii**) *Peponiumelgonense* (new species) **B***Swertiacrassiuscula* (regional endemic) **C***Plantagolanceolata* (exotic) **D***Indigoferahomblei* (exotic) **E***Tripogonmajor* (rare and endemic) **F***Aloeelgonica* (Endemic to Mt Elgon).

This study presents an expansive checklist of the flora of Mt Elgon constituting 1709 taxa (1589 species, 75 subspecies and 45 varieties) representing 673 genera in 131 families. The plant taxa were classified into five classes, namely: Magnoliopsida 1230 species (72%), Liliopsida 396 species (23%), Polypodiopsida 72 (4%), Lycopodiopsida 7 species (0.4%), and Pinopsida 4 species (0.2%) (Table 1). On comparison, the top 20 most species rich families recorded include; Asteraceae (191), Fabaceae (168), Orchidaceae (114), Poaceae (98), Lamiaceae (79), Cyperaceae (59), Rubiaceae (58), and Acanthaceae (45 species) (Table 2). The top species-rich genera were *Cyperus* (31), *Asplenium* (29), *Crotalaria* (27), *Habenaria* (25), *Helichrysum* (24), and *Coleus* (21) (Table 2). During field excursions between the year 2018 and 2019, a new species of the family Cucurbitaceae in the genus *Peponium* was discovered (*Peponiumelgonense*; Mutie et al. 2020). We also made herbarium collections and took photographs of plants endemic to Mt Elgon, as well as rare and endemic, regional endemic, and exotic species (Fig. 2).

**Table 1.** Number of plant families, genera, species, varieties and subspecies present in each class.

Class	Families	Genera	Species	Varieties	Subspecies
Magnoliopsida	98	502	1230	39	61
Liliopsida	19	136	396	6	13
Polypodiopsida	9	28	72	0	1
Lycopodiopsida	3	4	7	0	0
Pinopsida	2	3	4	0	0

**Table 2.** The 20 highest species-rich families and genera recorded from Mt Elgon.

Family	No species	No of genera	Genus	No of species
Asteraceae	191	68	*Crotalaria* L. (Fabaceae)	27
Fabaceae	168	48	*Habenaria* Willd. (Orchidaceae)	25
Orchidaceae	114	31	*Helichrysum* Mill. (Asteraceae)	24
Poaceae	98	50	*Coleus* Lour. (Lamiaceae)	21
Lamiaceae	79	25	*Indigofera* L. (Fabaceae)	18
Cyperaceae	59	7	*Solanum* L. (Solanaceae)	20
Rubiaceae	58	24	*Asplenium* L. (Aspleniaceae)	29
Acanthaceae	45	17	*Euphorbia* L. (Euphorbiaceae)	16
Apocynaceae	39	23	*Commelina* Plum. ex L. (Commelinaceae)	14
Malvaceae	36	13	*Carex* L. (Cyperaceae)	15
Asparagaceae	34	10	*Cyperus* L. (Cyperaceae)	31
Aspleniaceae	29	1	*Lobelia* Plum. ex L. (Campanulaceae)	14
Euphorbiaceae	29	8	*Polystachya* Hook. (Orchidaceae)	14
Commelinaceae	27	4	*Eulophia* R.Br. (Orchidaceae)	13
Orobanchaceae	26	9	*Trifolium* Tourn. ex L. (Fabaceae)	12
Apiaceae	25	18	*Senecio* L. (Asteraceae)	10
Campanulaceae	24	4	*Impatiens* Riv. ex L. (Balsaminaceae)	10
Solanaceae	24	5	*Vigna* Savi (Fabaceae)	11
Rosaceae	18	5	*Swertia* L. (Gentianaceae)	10
Iridaceae	17	8	*Hypericum* Tourn. ex L. (Hypericaceae)	10

### ﻿Growth habit

**Figure 3.**
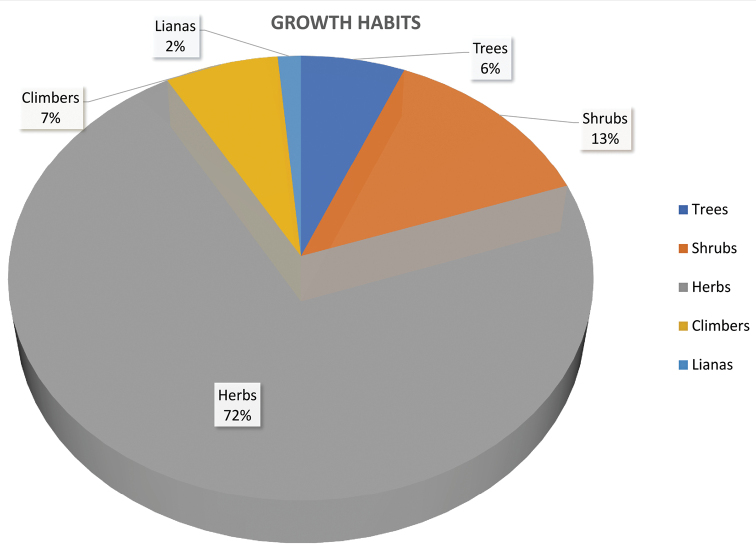
Different growth forms of the vascular plants of Mt Elgon.

The plant life forms of Mt Elgon were categorized as either trees, shrubs, climbers, lianas, or herbs. Herbs constituted the highest percentage of forest cover (72%) of the total species recorded, which consisted largely of terrestrial plants, few aquatic (9 species) and epiphytic (45 species) plants. The other growth habits constituted shrubs (13% with 228 species), climbers (6% with 118 species), trees (6% with 108 species), and lianas (2% having 24 species) (Fig. 3).

### ﻿Endemism and conservation status

**Figure 4.**
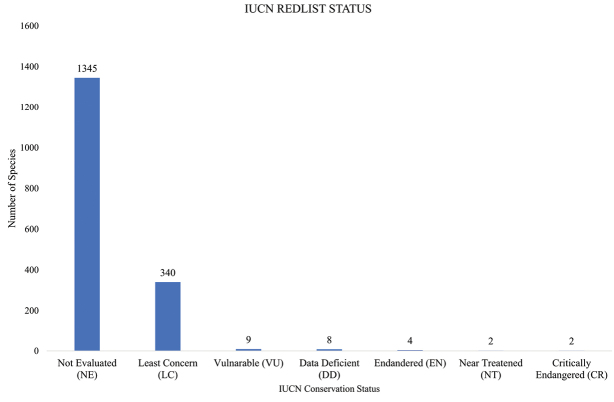
Number of species in each IUCN Red List category conservation status of vascular plants of Mt Elgon.

The conservation status of the plants of Mt Elgon are recorded according to the IUCN Red list of Threatened species (IUCN 2021). A large proportion of the species have not been evaluated adding up to 1345 species (Fig. 4). A total of 340 species were of Least Concern (LC), 2 Near Threatened (NT), 9 Vulnerable (VU), 4 Endangered (EN) and 2 Critically Endangered (CR) (Fig. 4, Table 3). *Bothrioclineauriculata* is the only critically endangered species native to the Ugandan side of Mt Elgon, known from 7 collections in 5 localities and has a decreasing population (IUCN 2021). The only exotic species recorded in Mt Elgon that is threatened (Critically Endangered with a decreasing population) in its native range (a limited distribution range in Northern Central and Eastern Central America) is *Fraxinusquadrangulata* (Wallander 2008; IUCN 2021).

**Table 3.** List of threatened plants species found in Mt Elgon, IUCN Red list category and population trends.

Family	Species	Habit	IUCN status	Population trend	Version
Acanthaceae	*Diclipteranilotica* C.B.Clarke	Herb	VU	Decreasing	3.1
Asteraceae	*Bothrioclineauriculata* (M.Taylor) C.Jeffrey	Herb or shrub	CR	Decreasing	3.1
Oleaceae	*Fraxinusquadrangulata* Michx.	Tree	CR	Decreasing	3.1
Asteraceae	*Helichrysumellipticifolium* Moeser	Shrub	VU	Unknown	3.1
Fabaceae	*Galegalindblomii* (Harms) J.B.Gillett	Herb	EN	Unknown	3.1
Cyperaceae	*Carexmonostachya* A.Rich.	Herb	VU	Unknown	3.1
Araliaceae	*Polysciaskikuyuensis* Summerh.	Tree	NT	Unknown	3.1
Fabaceae	*Angylocalyxbraunii* Harms	Tree	VU	Unspecified	3.1
Hydrocharitaceae	*Lagarosiphonhydrilloides* Rendle	Aquatic herb	EN	Unknown	3.1
Meliaceae	*Entandrophragmaangolense* C.CD.	Tree	NT	Threatened	3.1
Orchidaceae	*Platantheramicrantha* Schltr.	Herb	EN	Decreasing	3.1
Orchidaceae	*Anselliaafricana* Lindl.	Herb	VU	Decreasing	3.1
Orchidaceae	*Angraecopsistenerrima* Kraenzl.	Epiphytic herb	EN	Decreasing	3.1
Poaceae	*Colpodiumhedbergii* (Melderis) Tzvelev	Herb	VU	Unknown	3.1
Poaceae	*Deschampsiaangusta* Stapf & C.E.Hubb.	Herb	VU	Unknown	3.1
Polypodiaceae	*Arachniodeswebbiana* (A.Braun) Schelpe	Herb	VU	Stable	3.1
Rosaceae	*Prunusafricana* (Hook.f.) Kalkman	Tree	VU	Decreasing	3.1

**Figure 5.**
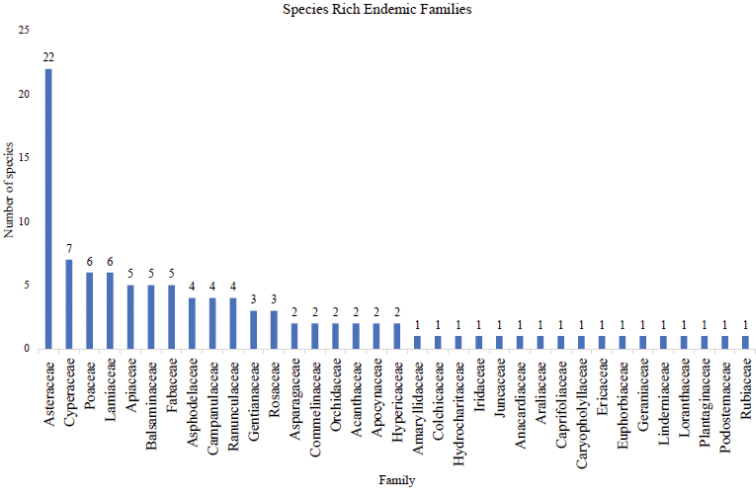
Families with high number of endemic species from Mt Elgon vascular plants.

**Figure 6.**
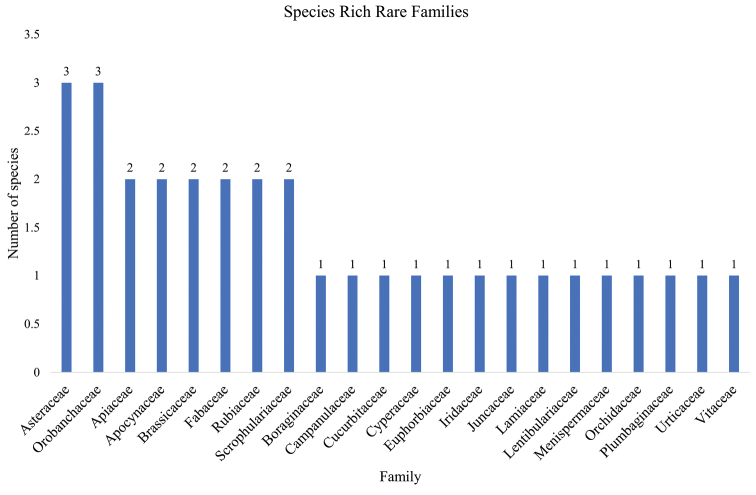
Families with high number of rare species from Mt Elgon.

**Figure 7.**
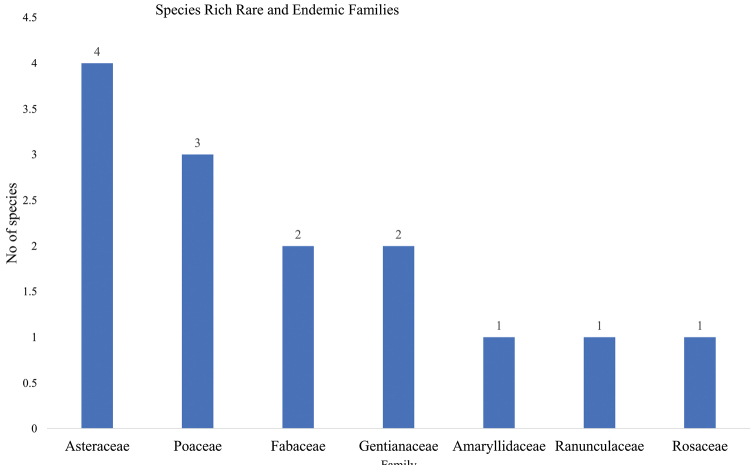
Families with high number of rare and endemic species from Mt Elgon.

A total of 143 species recorded were found to be rare, endemic, or both. They comprised 103 endemic species in 35 families, 32 rare species in 22 families, and 14 that were both rare and endemic belonging to seven families. The families with a high number of endemic species include Asteraceae (22) and Cyperaceae (7) (Fig. 5), while species-rich families of rare species were Asteraceae (3) and Orobanchaceae (3) (Fig. 6). Asteraceae (4) and Poaceae (3) constituted the highest number of both rare and endemic species (Fig. 7).

### ﻿Residence status

**Figure 8.**
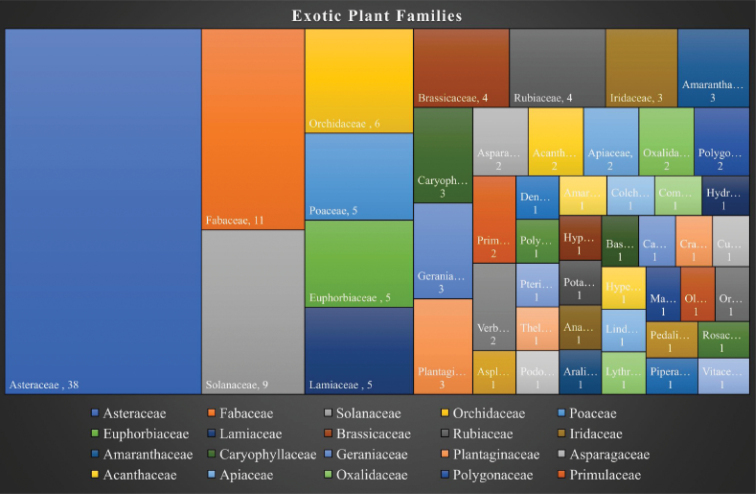
Families with highest number of exotic species of the vascular plants of Mt Elgon.

Species of Mt Elgon were categorized according to their natural place of origin. All introduced cultivated or naturalized species were termed as exotic according to the Plants of the World Online (POWO 2022) (https://powo.science.kew.org). In Mt Elgon, a total of 144 species from 49 families were determined as exotic (Table 4). This represents 8.4% of the total species richness observed in Mt Elgon from this study. Asteraceae (38), Fabaceae (11), Solanaceae (9), Orchidaceae (6), Lamiaceae (5), and Euphorbiaceae (5) constituted the highest number of exotic species (Fig. 8). Being a transboundary mountain, 127 species were exclusively native to Kenya, while 29 species were only native to Uganda. However, since the mountain has no other physical or geographical barrier, exclusively native species were considered native to Mt Elgon.

**Table 4.** List of exotic species and their growth habits recorded from Mt Elgon.

Family	Species	Habit	Status
Acanthaceae	*Justiciainsularis* T.Anderson	Herb	Exotic
Acanthaceae	*Diclipteracolorata* C.B.Clarke	Herb	Exotic
Anacardiaceae	*Searsianatalensis* (Bernh. ex Krauss) F.A.Barkley	Shrub or tree	Exotic
Apiaceae	*Torilisarvensis* (Huds.) Link	Herb	Exotic
Apiaceae	*Ammimajus* L.	Herb	Exotic
Araliaceae	*Hydrocotyleranunculoides* L.f.	Herb	Exotic
Asteraceae	*Achilleamillefolium* L.	Herb	Exotic
Asteraceae	*Adenostemmacaffrum* DC.	Herb	Exotic
Asteraceae	*Chrysanthellumamericanum* (L.) Vatke	Herb	Exotic
Asteraceae	*Sonchusoleraceus* L.	Herb	Exotic
Asteraceae	*Sonchusasper* (L.) Hill	Herb	Exotic
Asteraceae	*Ethuliaconyzoides* L.	Herb	Exotic
Asteraceae	Gutenbergiacordifoliavar.marginata (O.Hoffm.) C Jeffrey	Herb	Exotic
Asteraceae	Baccharoidescalvoanasubsp.leucocalyx (O.Hoffm.) Isawumi, El-Ghazaly & B.Nord.	Shrub, woody herb, rarely tree	Exotic
Asteraceae	*Vernoniaschweinfurthii* Oliv. & Hiern	Herb	Exotic
Asteraceae	*Gnaphaliumdeclinatum* L.f.	Herb	Exotic
Asteraceae	*Gamochaetapurpurea* (L.) Cabrera	Herb	Exotic
Asteraceae	*Helichrysumluteoalbum* (L.) Rchb.	Herb	Exotic
Asteraceae	*Helichrysumpanduratum* O.Hoffm. ex De Wild. & T.Durand	Shrub	Exotic
Asteraceae	*Helichrysumsetosum* Harv.	Herb	Exotic
Asteraceae	*Helichrysumstenopterum* DC.	Herb	Exotic
Asteraceae	*Eschenbachiastricta* (Willd.) Raizada	Herb	Exotic
Asteraceae	*Cotulaaustralis* Hook.f.	Herb	Exotic
Asteraceae	*Artemisiaafra* Jacq.	Shrub	Exotic
Asteraceae	*Cinerariadeltoidea* Sond.	Herb	Exotic
Asteraceae	*Cirsiumvulgare* (Savi) Ten.	Robust herb	Introduced
Asteraceae	*Emiliasonchifolia* (L.) DC.	Herb	Exotic
Asteraceae	*Emiliaintegrifolia* Baker	Herb	Exotic
Asteraceae	*Crassocephalumpicridifolium* S.Moore	Herb	Exotic
Asteraceae	*Crassocephalumcrepidioides* (Benth.) S.Moore	Herb	Exotic
Asteraceae	*Mikaniopsisclematoides* (Sch Bip. ex A Rich.) Milne-Redh.	Subshrub	Exotic
Asteraceae	*Dendroseneciojohnstonii* (H.H.Johnst.) B.Nord.	Tree or shrub	Exotic
Asteraceae	*Solanecioangulatus* (Vahl) C.Jeffrey	succulent herb	Exotic
Asteraceae	*Solaneciomannii* (Hook.F.) C.Jeffrey	Shrub or small tree	Exotic
Asteraceae	*Spilanthesmauritiana* (A.Rich ex Pers.) DC.	Herb	Exotic
Asteraceae	*Acmellacaulirhiza* Delile	Herb	Exotic
Asteraceae	*Galinsogaparviflora* Cav.	Herb	Exotic
Asteraceae	*Galinsogaquadriradiata* Ruiz & Pav.	Herb	Exotic
Asteraceae	*Bidensflagellata* (Sherff) Mesfin	Herb	Exotic
Asteraceae	*Bidenspilosa* L.	Herb	Exotic
Asteraceae	*Chromolaenacorymbosa* (Aubl.) R.M.King & H Rob.	Herb	Exotic
Asteraceae	*Ageratinaadenophora* (Spreng.) R.M.King & H Rob.	Herb or shrub	Exotic
Asteraceae	*Mikaniacordata* (Burm.f.) B.L.Rob.	Shrub or subshrub	Exotic
Campanulaceae	*Lobeliaheyneana* Roem. & Schult.	Herb	Exotic
Caryophyllaceae	*Corrigiolalitoralis* L.	Herb	Exotic
Euphorbiaceae	*Euphorbiaprostrata* Aiton	Herb	Exotic
Euphorbiaceae	*Euphorbiamurielii* N.E.Br.	Tree	Exotic
Euphorbiaceae	*Euphorbiarubella* Pax	Herb	Exotic
Fabaceae	Vachelliasieberianavar.woodii (Burtt Davy) Kyal. & Boatwr.	Tree or shrub	Exotic
Fabaceae	*Indigoferahomblei* Baker f. & Martin	Shrub	Exotic
Fabaceae	*Indigoferaemarginella* Steud. ex A.Rich.	Shrub	Exotic
Fabaceae	*Indigoferaschlechteri* Baker f.	Woody herb or shrub	Exotic
Fabaceae	Macrotylomaaxillarevar.glabrum (E.Mey.) Verdc.	Climbing or trailing herb	Exotic
Fabaceae	Rhynchosiaminimavar.prostrata (Harv.) Meikle	Climbing herb	Exotic
Fabaceae	*Viciaeriocarpa* (Hausskn.) Halácsy	Herb	Exotic
Fabaceae	*Viciavillosa* Roth	Herb	Exotic
Geraniaceae	*Geraniummascatense* Boiss.	Herb	Exotic
Geraniaceae	*Geraniumbequaertii* De Wild.	Herb	Exotic
Geraniaceae	*Pelargoniumalchemilloides* (L.) L’Hér.	Herb	Exotic
Hypericaceae	*Hypericumlanceolatum* Lam.	Shrub or tree	Exotic
Lamiaceae	*Leucastettensis* Vatke	Herb	Exotic
Lamiaceae	*Orthosiphonrubicundus* Benth.	Herb	Exotic
Lamiaceae	*Aeollanthuspubescens* Benth.	Herb	Exotic
Linderniaceae	*Bonnayaciliata* Spreng.	Herb	Exotic
Lythraceae	*Ammanniaaegyptiaca* Willd.	Herb	Exotic
Oleaceae	*Fraxinusquadrangulata* Michx.	Tree	Exotic
Orobanchaceae	*Sopubiatrifida* Buch.-Ham. ex D.Don	Herb	Exotic
Plantaginaceae	*Callitrichestagnalis* Scop.	Herb	Exotic
Plantaginaceae	*Veronicacalycina* R.Br.	Herb	Exotic
Podocarpaceae	*Afrocarpusfalcatus* (Thunb.) C.N.Page	Tree	Exotic
Polygonaceae	*Persicariapulchra* Soják	Herb	Exotic
Primulaceae	*Lysimachiatenella* L.	Herb	Exotic
Rubiaceae	PentasLanceolatasubsp.quartiniana (A.Rich) Verdc	Herb or subshrub	Exotic
Rubiaceae	*Galiumtanganyikense* Ehrend. & Verdc.	Herb	Exotic
Rubiaceae	*Galiumaparine* L.	Herb	Exotic
Solanaceae	*Daturastramonium* L.	Herb	Exotic
Solanaceae	*Physalisperuviana* L.	Herb	Exotic
Solanaceae	*Solanumpseudospinosum* C.H.Wright	Herb	Exotic
Solanaceae	*Solanumcapsicoides* All.	Herb or shrub	Exotic
Solanaceae	*Solanumdasyphyllum* Schumach. & Thonn.	Herb	Exotic
Solanaceae	*Solanumnigrum* L.	Herb	Exotic
Verbenaceae	*Lantanatrifolia* L.	shrub or subshrubby Herb	Exotic
Vitaceae	Cyphostemmajunceumsubsp.jatrophoides (Baker) Verdc.	Herb	Exotic
Amaryllidaceae	*Crinumzeylanicum* (L.) L.	Herb	Exotic
Asparagaceae	*Asparagusasiaticus* L.	Climber or shrub	Exotic
Asparagaceae	*Ornithogalumgussonei* Ten.	Herb	Exotic
Colchicaceae	*Androcymbiummelanthioides* Willd.	Herb	Exotic
Commelinaceae	Commelinaafricanavar.krebsiana (Kunth) C.B.Clarke	Herb	Exotic
Hydrocharitaceae	*Najaspectinata* Magnus	Herb	Exotic
Hypoxidaceae	*Hypoxisvillosa* L.f.	Herb	Exotic
Iridaceae	*Moraeacarsonii* Baker	Herb	Exotic
Iridaceae	*Moraeavegeta* L.	Herb	Exotic
Iridaceae	*Dieramapendulum* Baker	Herb	Exotic
Orchidaceae	*Arachnisflos-aeris* (L.) Rchb.f.	Herb	Exotic
Orchidaceae	*Platantheramicrantha* (Hochst. ex Seub.) Schltr.	Herb	Exotic
Orchidaceae	*Epidendrumibaguense* Kunth	Herb	Exotic
Orchidaceae	Satyriumneglectumsubsp.woodii (Schltr.) A.V.Hall	Herb	Exotic
Orchidaceae	Bulbophyllumjosephivar.mahonii (Rolfe) J.J.Verm.	Herb	Exotic
Orchidaceae	*Angraecopsistenerrima* Kraenzl.	Herb	Exotic
Poaceae	*Stipadregeana* Steud.	Herb	Exotic
Poaceae	*Festucacamusiana* St.-Yves	Herb	Exotic
Poaceae	*Eleusinecoracana* Gaertn.	Herb	Exotic
Potamogetonaceae	*Potamogetonnodosus* Poir.	Herb	Exotic
Aspleniaceae	*Aspleniumthunbergii* Kunze	Herb	Exotic
Dennstaedtiaceae	*Pteridiumaquilinum* (L.) Kuhn	Herb	Exotic
Polypodiaceae	*Polystichumsinense* (Christ) Christ	Herb	Exotic
Pteridaceae	*Doryopterisconcolor* (Langsd. & Fisch.) Kuhn	Herb	Exotic
Thelypteridaceae	*Thelypterispozoi* (Lag.) Morton	Herb	Exotic
Euphorbiaceae	*Ricinuscommunis* L.	Herb or subshrub	Doubtful for Kenya (Exotic)
Asteraceae	*Tagetesminuta* L.	Herb	Introduced to Kenya (Exotic)
Amaranthaceae	*Amaranthushybridus* L.	Herb	Introduced (Exotic)
Basellaceae	*Basellaalba* L.	Herb or shrub	Introduced (Exotic)
Brassicaceae	*Mummenhoffiaalliacea* (L.) Esmailbegi & Al-Shehbaz	Herb	Introduced (Exotic)
Brassicaceae	*Capsellabursa-pastoris* (L.) Medik.	Herb	Introduced (Exotic)
Caryophyllaceae	*Silenegallica* L.	Herb	Introduced (Exotic)
Euphorbiaceae	*Euphorbiahirta* L.	Herb	Introduced (Exotic)
Fabaceae	*Biancaeadecapetala* (Roth) O.Deg.	Shrub	Introduced (Exotic)
Fabaceae	*Sennabicapsularis* (L) Roxb.	Shrub or tree	Introduced (Exotic)
Malvaceae	*Malvaverticillata* L.	Herb	Introduced (Exotic)
Oxalidaceae	*Oxaliscorniculata* L.	Herb	Introduced (Exotic)
Oxalidaceae	*Oxalislatifolia* Kunth	Herb	Introduced (Exotic)
Pedaliaceae	*Sesamumindicum* L.	Herb	Introduced (Exotic)
Rubiaceae	*Spermacocepusilla* Wall.	Herb	Introduced (Exotic)
Solanaceae	*Solanumwendlandii* Hook.f.	Climber and liana	Introduced (Exotic)
Solanaceae	*Solanummauritianum* Scop.	Shrub or small tree	Introduced (Exotic)
Solanaceae	*Solanumaculeatissimum* Jacq.	Herb or Shrub	Introduced (Exotic)
Poaceae	*Cynodontransvaalensis* Burtt Davy	Herb	Introduced (Exotic)
Piperaceae	*Piperumbellatum* L.	shrub or woody herb	Introduced and Naturalised (Exotic)
Fabaceae	*Vignahosei* (Craib) Backer	Herb	Introduced to Kenya (Exotic)
Amaranthaceae	*Chenopodiastrummurale* (L.) S.Fuentes, Uotila & Borsch	Herb	Introduced to Kenya (Exotic)
Amaranthaceae	*Chenopodiumalbum* L.	Herb	Introduced to Kenya (Exotic)
Brassicaceae	*Brassicajuncea* (L.) Czern	Herb	Introduced to Kenya (Exotic)
Brassicaceae	*Brassicanapus* L.	Herb	Introduced to Kenya (Exotic)
Cucurbitaceae	*Sicyospolyacanthos* Cogn.	Climber	Introduced to Kenya (Exotic)
Lamiaceae	*Coleusrotundifolius* (Poir.) A. Chev. & Perrot	Herb	Introduced to Kenya (Exotic)
Plantaginaceae	*Plantagolanceolata* L.	Herb	Introduced to Kenya (Exotic)
Polygonaceae	*Fallopiaconvolvulus* (L.) Á.Löve	climbing herb	Introduced to Kenya (Exotic)
Primulaceae	*Lysimachiaarvensis* (L.) U.Manns & Anderb.	Herb	Introduced to Kenya (Exotic)
Rosaceae	*Rubusniveus* Thunb.	Shrub	Introduced to Kenya (Exotic)
Verbenaceae	*Verbenabrasiliensis* Vell.	Herb	Introduced to Kenya (Exotic)
Asteraceae	*Erigeronbonariensis* L.	Herb	Introduced to Kenya (Exotic))
Poaceae	*Brizamaxima* L.	Herb	Introduced to Kenya (Exotic))
Lamiaceae	*Salviacoccinea* Buc’hoz ex Etl.	Herb	Naturalised-introduced (Exotic)
Caryophyllaceae	*Stellariamedia* (L.) Vill.	Herb	Naturalized-introduced (Exotic)

## ﻿Discussion

Mt Elgon forms a significant ecosystem to both Kenya and Uganda for its ability to sustain both high numbers of plants as reported in this study, as well as supporting a large number of human populations (Russell et al. 2017). It is known to be among the oldest mountains in East Africa and hence serve as the potential origin from where some species emanated and dispersed to their current distribution ranges (Tweedie 1976). Besides the scarcity of recent floristic studies in this mountain, it is among the hotspot regions of Africa for scientific studies on flora, fauna, and archeological studies (Bower 1974; CEPF 2012). This is evidenced by the high number of species recorded in this study, as well as the similarity of its species to different floristic regions of both close and distant proximity (Mbuni et al. 2019). Out of more than 12,000 species recorded in the flora of Tropical Africa, Mt Elgon hosts about 1709 species representing 14.24% of the Tropical African flora (FTEA editors 1952–2012). The number is expected to fluctuate and to either increase due to the discovery of new species, or reduce as a result of forest degradation and human encroachment. This could only be properly monitored based on field observations.

This study reports high species richness as compared to the previously available checklists (Bullock 1933; Tweedie 1976) whose work remain paramount to the current knowledge of Mt Egon. In this study, the highest species-rich family was Asteraceae, which is consistent with other floristic studies of East Africa (Mbuni et al. 2019; Kipkoech et al 2020; Melly et al. 2020; Watuma et al. 2022). Other common high species families, though not in the same sequence of richness, include Poaceae, Orchidaceae, Lamiaceae, and Cyperaceae. Interestingly, the family Asteraceae also constitutes the highest number of rare and endemic species of Mt Elgon. This calls for higher conservation of threatened species and species in the family Asteraceae among tropical montane forest, due to their potential high phylogenetic diversity, which increases the ability of the associated community to adapt to environmental stress and climate change (Chaves et al. 2021).

Compared to resent floristic studies in East Africa, Mt Elgon accounts for the highest species richness in the region (Zhou et al. 2018; Mbuni et al. 2019; Kipkoech et al. 2020; Melly et al. 2020; Watuma et al. 2022). This could be as a result of its strategic position among Afromontane regions, being an intersection of several floristic regions, its old age and isolation from other high tropical montane forests (CEPF 2012). However, compared to other highland forests, the increasing encroachment and anthropogenic activities in and around the mountain pose higher threats to the mountain ecosystem (Sassen et al. 2013; Masayi et al. 2021). Similarly, the presence of many exotic species poses threats to the native species in terms of competition for resources. Exotic species may thrive better than native species and become invasive due to their ability to adapt to new habitats, as well as adjusting to the impact of climate change, causing partial or total displacement of native species (Michelan et al. 2010). For instance, *Solanumincanum* also known as bush encroacher, *Solanummauritianum* and *Lantanacamara*, have been recorded in Mt Elgon among the exotic species and are known to be among the 100 most noxious invasive species in East Africa (Lusweti et al. 2011). Furthermore, the native species are preferred for charcoal burning, timber and wood harvesting, as well as for medicinal purposes, and are thus rendered susceptible to depletion. Therefore, there is a need to strengthen conservation strategies, especially since human settlements are evident up to the moorland zone on the forest reserve (Ongugo et al. 2002).

Though there are evident efforts of forest regeneration through massive plantation and forest intrusion mitigation policies (Roga 2017), it is essential that the governments of both Kenya and Uganda join efforts to enforce planting native and indigenous trees, shrubs and herbs. This will, in turn, overcome the impending forest structure transformation and improve the capacity of Mt Elgon to sustain its ecosystem (flora and fauna), which forms the basis of livelihood to the surrounding population (Russell et al. 2017). Furthermore, it will secure springs of water that serve as sources of all permanent rivers, including the longest river in Africa, the Nile River.

## ﻿Conclusion

Conservation efforts of Mt Elgon have been in progress for many decades. This is because it forms a significant ecosystem of the East African mountains due to its unique flora shaped by its old age and isolation from other high tropical montane forests. This study provides a comprehensive list of plant species occurring on Mt Elgon and outlines characteristic species associated with this ecosystem that need priority conservation. It reports rare and endemic species, as well as the conservation status of evaluated species according to the IUCN. The existence of a high number of exotic species, which may alter the structure and species composition of the ecosystem, is of concern to Mt Elgon and needs further studies. The present study therefore builds on the foundation laid in previous studies and broadens the scope of possible ecological and phylogenetic studies in this region. Amid the biodiversity crisis, there is an urgent need for enhanced conservation of Mt Elgon’s natural resources in order to preserve the extant species and the landscape features associated with its evolutionary formation and diversification.

## ﻿Checklist

The annotated checklist of plants of Mt Elgon records species in Kenya and Uganda for the benefit for scientific exploration of its flora, as well as broader research on conservation. The checklist is arranged alphabetically at family, genus, and species level. The plants are grouped into five classes: (i) Lycopodiopsida; (ii) Polypodiopsida; (iii) Pinopsida; (iv) Liliopsida; and (v) Magnoliopsida. The native range of the species are recorded for the benefit of biodiversity studies. The Pteridophyte Phylogeny Group I (PPG I) (2016) was used as the basis of classification of Lycopodiopsida and Polypodiosida; Christenhusz et al. (2011) for Pinopsida; and APG IV (2016) for the classification of Magnoliopsida and Liliopsida. For each species, information on growth habit, habitat, collection vouchers, altitudinal occurrence range, and their native distribution ranges are provided. In the cases of unavailable vouchers, descriptive monographs were cited. Species endemic to Mt Elgon are indicated by (**) while those endemic to the region in which Mt Elgon is located are indicated by (*). Exotic species are also indicated in the checklist by (^EX^).

### ﻿Part 1 Lycopodiopsida

#### F1. ISOETACEAE

1 Genus, 1 species

***Isoetesabyssinica* Chiov.** – Habit: Herb. Habitat: Seasonal rock outcrop, 1520–2400 m. Voucher: Agnew ADQ (2013) (REF). Native distribution range: Eritrea to E Central & E Tropical Africa.

#### F2. LYCOPODIACEAE

2 Genera, 3 species

***Huperziadacrydioides* (Baker) Pic.Serm.** – Habit: Herb. Habitat: Montane Forest, 1500–2800 m. Vouchers: Holm Å 47 (S). Native distribution range: Ethiopia to S Africa, Comoros.

***Huperziasaururus* (Lam.) Trevis.** – Habit: Herb. Habitat: Moorland, Ericaceous zone, 2550–4300 m. Vouchers: Åke Strid 3584 (S), Holm Å 46 (S), Hedberg O 1037 (S). Native distribution range: Ascension, St Helena, Cameroon to Ethiopia and S Africa, W Indian Ocean, Peru to Subantarctic Islands.

***Lycopodiumclavatum* L.** – Habit: Herb. Habitat: Bamboo zone, roadside, montane forest, 2000–2800 m. Vouchers: Katende 1034 (K), Mwangangi OM 482 (WAG.). Native distribution range: Temperate Northern Hemisphere to Tropical Mountains.

#### F3. SELAGINELLACEAE

1 Genus, 3 species

***Selaginellacaffrorum* (Milde) Hieron.** – Habit: Herb. Habitat: Rock crevices, 1450–2300 m. Voucher: Wood GHS 449 (EA). Native distribution range: Sudan to S Africa.

***Selaginellagoudotiana* Spring** – Habit: Herb. Habitat: Moist forest, moist thickets, 1250–2450 m. Voucher: Brown EJ in EA 12492 (EA), Wesche K 391 (US). Native distribution range: Tropical Africa, SW Arabian Peninsula, Madagascar.

***Selaginellakraussiana* (Kunze) A.Braun** – Habit: Herb. Habitat: Moist forest floor, bamboo zone, 1300–3100 m. Vouchers: Wesche K 505 (US), Rono et al. SAJIT-PR 0271 (EA, HIB). Native distribution range: Macaronesia, Tropical & S Africa.

### ﻿Part 2 Polypodiopsida

#### F4. ASPLENIACEAE

1 Genus, 29 species

***Aspleniumabyssinicum* Fée** – Habit: Herb. Habitat: Bamboo zones, montane forest, 1850–3600 m. Vouchers: Katende T; Sheil D 2239 (K), Wood GHS 178 (MO), Hedberg O 151 (S). Native distribution range: Tropical Africa.

***Aspleniumactiniopteroides* Peter** – Habit: Herb. Habitat: Moorland, 2500–4250 m. Voucher: Wesche K 853 (US), 107 (EA), 1078 (US), Åke Strid 3561 (S), Hedberg O 954 (S), Molesworth-Allen BEG 3672 (S). Native distribution range: Ethiopia to E Central & E Tropical Africa.

***Aspleniumadamsii* Alston** – Habit: Herb. Habitat: Moorland, upper ericaceous zones, 2970–3400 m. Voucher: Agnew ADQ (2013) (REF). Native distribution range: Cameroon, Ethiopia to Tanzania.

***Aspleniumadiantum-nigrum* L.** – Habit: Herb. Habitat: Roadsides, bamboo zones, heath scrub, ericaceous zone, moorland, 3000–3800 m. Voucher: Hedberg O 906 (EA). Native distribution range: Temperate Eurasia, Macaronesia, NW & S Africa to Tropical African Mountains, W Central America to N Mexico, Hawaiian Islands.

***Aspleniumaethiopicum* (Burm.f.) Bech.** – Habit: Herb. Habitat: Moist bushland, moist forest, bamboo zone, rock crevices, moorland, 1430–3600 m. Vouchers: Charles E DeVol 5 (MICH), Bamps PRJ 6519 (BR), Sheil D; Katende T 836, 764 (K), Tiyoy LM 1242 (K), Wesche K 1412 (EA), 1271 (K), 897 (US), Wood C 173 (US), Beentje HJ 1932 (WAG). Native distribution range: Tropical & Subtropical Old World to Pacific.

***Aspleniumanisophyllum* Kunze** – Habit: Herb. Habitat: Moist forest, 1000–1600 m. Vouchers: Hedberg O 89 (S), Holm Å 44 (S). Native distribution range: Tropical & S Africa.

***Aspleniumauriculatum* Sw.** – Habit: Herb. Habitat: Moist forest, moorland, (2400–)2600–4200 m. Voucher: Synge PM 1033 (MO). Native distribution range: Tropical & Subtropical America.

***Aspleniumboltonii* Hook. ex Schelpe** – Habit: Herb. Habitat: Bamboo zone, montane forest, 1600–2750 m. Vouchers: Wesche K 1272 (S), Granvik H 343 (S). Native distribution range: Tanzania to S Africa, Madagascar, Réunion.

***Aspleniumbuettneri* Hieron.** – Habit: Herb. Habitat: Riverine forest, 1450–1700 m. Voucher: Molesworth-Allen BEG 3673 (US). Native distribution range: Tropical Africa, Comoros, Madagascar.

***Aspleniumbugoiense* Hieron.** – Habit: Herb. Habitat: Moist montane forest, bamboo forest, 2000–2700 m. Vouchers: Wesche K 579 (US), Molesworth-Allen BEG 3671 (US), Rono et al. SAJIT-PR 0199 (EA, HIB). Native distribution range: Ethiopia to Rwanda.

***Aspleniumceii* Pic.Serm.** – Habit: Herb. Habitat: Moist forest, 1700–2430 m. Voucher: Gardner 1028 (EA). Native distribution range: Gabon to Ethiopia and Mozambique.

***Aspleniumchristii* Hieron.** – Habit: Herb. Habitat: Moist Forest, 1450–1900 m. Voucher: Granvik H 361 (S). Native distribution range: Kenya to South Africa.

***Aspleniumelliottii* C.H.Wright** – Habit: Herb. Habitat: Moist montane forest, bamboo forest, 1200–2650 m. Vouchers: Tweedie EM 1899 (S), Molesworth-Allen BEG 3670 (US). Native distribution range: Ethiopia, E Central & E Tropical Africa.

***Aspleniumerectum* Bory ex Willd.** – Habit: Herb. Habitat: Moist forest, 1300–2750 m. Vouchers: Wesche K 690 (US), Hedberg O 90 (S). Native distribution range: Tropical & S Africa, W Indian Ocean, India, Sri Lanka, Jawa, Lesser Sunda Islands (Timor), Samoa (Savai’i).

***Aspleniumfriesiorum* C.Chr.** – Habit: Herb. Habitat: Riverine thickets, bamboo forest, moist forest, 2700–3000 m. Vouchers: Katende T; Sheil D 2258 (K), van Heist M 571 (K). Native distribution range: Tropical & S Africa, W Indian Ocean.

***Aspleniumgemmascens* Alston** – Habit: Herb. Habitat: Moist forest, 1100–1150 m. Voucher: Agnew ADQ (2013) (REF). Native distribution range: S Nigeria to Uganda and Angola.

***Aspleniumgemmiferum* Schrad.** – Habit: Herb. Habitat: Forest, 1500–2300 m. Voucher: Holm Å 48 (S). Native distribution range: Bioko to Uganda and S Africa, Réunion, Mauritius.

***Aspleniumhypomelas* Kuhn** – Habit: Herb. Habitat: Riverine, moist forest, rock outcrops, 1500–2400 m. Vouchers: Kisalye N; van Heist M 564 (K), Gerhard Lindblom 577 (S). Native distribution range: Tropical Africa to South Africa.

***Aspleniumloxoscaphoides* Baker** – Habit: Herb. Habitat: Bamboo zone, moist forest, 3100–3650 m. Vouchers: Wesche K 1301 (US), Hedberg O 116 (S). Native distribution range: E Tropical Africa to Malawi.

***Aspleniumlunulatum* Sw.** – Habit: Herb. Habitat: Forest, 2200–2500 m. Vouchers: Åke Strid 3174 (S, MO). Native distribution range: Bioko, Ascension, St Helena, Kenya to S Africa.

***Aspleniummonanthes* L.** – Habit: Herb. Habitat: Bamboo zone, dry montane forest, 2100–3400 m. Vouchers: Beentje HJ 1954 (WAG), Brown EJ EA 12493 (U), Katende T; Sheil D 1144 (K), Wesche K 655 (K), Brown, EJ EA 12493 (U). Native distribution range: Macaronesia, Tristan da Cunha, Bioko, Cameroon, Somalia to S Africa, Madagascar, Réunion, Hawaiian Islands, Tropical & Subtropical America.

***Aspleniumprotensum* Schrad.** – Habit: Herb. Habitat: Moist forest, 1250–2900(–3300) m. Vouchers: Katende T; Sheil D 1073 (K), Wesche K 502 (US), Wood G 179 (US). Native distribution range: Tropical & S Africa, W Indian Ocean, N Yemen.

***Aspleniumsandersonii* Hook.** – Habit: Herb. Habitat: Forest, rock outcrops, 2600–3100 m. Voucher: Wesche K 1339 (US). Native distribution range: Tropical & S Africa, W Indian Ocean.

***Aspleniumsmedsii* Pic.Serm.** – Habit: Herb. Habitat: Bamboo zone, moist forest, 1500–3000 m. Voucher: Beentje 1958 (US), Wesche K 571 (US). Native distribution range: Ethiopia to Malawi, W Indian Ocean.

***Aspleniumstuhlmannii* Hieron.** – Habit: Herb. Habitat: Grassland, riverine forest, 1200–2230 m. Vouchers: Faden RB & Evans AJ 69/718 (US). Native distribution range: W Tropical Africa to Sudan and Tanzania, Madagascar.

***Aspleniumtheciferum* (Kunth) Mett.** – Habit: Herb. Habitat: Moist, forest, bamboo zone, 850–2900 m. Voucher: Wesche K 898 (US). Native distribution range: S Mexico to Tropical America, Africa.

**^EX^*Aspleniumthunbergii* Kunze** – Habit: Herb. Habitat: Moorland, (2400–)2600–4200 m. Voucher: Wood 177 (MO). Native distribution range: Madagascar, Sri Lanka, Assam to New Guinea.

***Aspleniumtrichomanes* L.** – Habit: Herb. Habitat: Moist rocky forest, 2130–2700 m. Voucher: Wesche K 1648 (US). Native distribution range: Cosmopolitan.

***Aspleniumuhligii* Hieron.** – Habit: Herb. Habitat: Moorland, forest, giant heath, woodland (2400–)2600–4200 m. Vouchers: Wood G 268 (K), Rose F 10,266 (K), 269 (K), Dummer RA 3331 (US). Native distribution range: Cameroon, Ethiopia to E Tropical Africa.

#### F5. CYATHEACEAE

1 Genus, 1 species

***Alsophilacamerooniana* (Hook.) R.M.Tryon** – Habit: Herb. Habitat: Afromontane mixed forest, by streams, 1500–2835 m. Vouchers: Kisalye N; van Heist M 535 (K). Native distribution range: W Tropical Africa to Angola.

#### F6. DENNSTAEDTIACEAE

2 Genera, 2 species

**Hypolepisrugosulasubsp.viscida (Roxb.) Schwartsb. & J.Prado** – Habit: Herb. Habitat: Bamboo zone, moorland, forest, 2700–3000 m. Voucher: Agnew ADQ (2013) (REF). Native distribution range: St Helena, Bioko, Cameroon to E Central & E Tropical Africa, Madagascar.

^**EX**^***Pteridiumaquilinum* (L.) Kuhn** – Habit: Herb. Habitat: Forest, 300–2600 m. Voucher: Agnew ADQ (2013) (REF). Native distribution range: Macaronesia, N & NE Tropical Africa, Arabian Peninsula, Europe to Siberia and Iran.

#### F7. HYMENOPHYLLACEAE

1 Genus, 1 species

***Trichomanesmelanotrichum* Schltdl.** – Habit: Epiphytic or lithophytic herb. Habitat: Moist forest, on tree trunks, rocks, 1460–3050 m. Vouchers: Beentje HJ 1953 (WAG), Dummer RA 3703 (NMNH), 3699, 3703 (US). Native distribution range: Tropical & S Africa, Madagascar, Mauritius.

#### F8. OPHIOGLOSSACEAE

1 Genus, 2 species

***Ophioglossumpolyphyllum* A.Braun ex Seub.** – Habit: Herb. Habitat: Woodland, montane grassland, forests, 1100–1350 m. Voucher: Faden & Evans 71/472 (EA). Native distribution range: Tropical & Subtropical Old World, Arizona to Texas and Mexico.

***Ophioglossumreticulatum* L.** – Habit: Herb. Habitat: Grassland, moist woodland, forest margin, 0–2500 m. Vouchers: Tweedie 1881 (EA), Rono et al. SAJIT-PR 0057 (EA, HIB). Native distribution range: Tropics & Subtropics.

#### F9. POLYPODIACEAE

11 Genera, 18 species

***Arachniodeswebbiana* (A.Braun) Schelpe** – Habit: Herb. Habitat: Wet forest, streamside, 1300–2600 m. Vouchers: Hedberg O 151 (S), Beentje HJ 1986 (US). Native distribution range: Madeira, Kenya, Uganda, Rwanda, Malawi, Tanzania, Zimbabwe to South Africa.

***Arthropterismonocarpa* (Cordem.) C.Chr.** – Habit: Herb. Habitat: Moist montane forest, near streams, bamboo zone, 1250–2450 m. Vouchers: Snowden JD 797 (NHMUK), Schippers RR K 229 (WAG), Collector unknown 3679 (US). Native distribution range: Tropical & S Africa, Comoros, Madagascar.

***Arthropterisorientalis* (Gmel.) Posth.** – Habit: Herb. Habitat: Moist thickets, 900–2400 m. Voucher: Agnew ADQ (2013) (REF). Native distribution range: Tropical Africa, W Indian Ocean, N Yemen.

***Dryopteristricellularis* J.P.Roux** – Habit: Herb. Habitat: Montane forest, riverine, bamboo zone, 2075–3420 m. Vouchers: Wesche K 649 (US), 471 (US). Native distribution range: Kenya to Uganda

***Elaphoglossumangulatum* (Blume) T.Moore** – Habit: Herb. Habitat: Bamboo, ericaceous zone, 2470–2600 m. Voucher: Wesche K 1277 (K). Native distribution range: E Central & E Tropical Africa, Madagascar, Tropical & Subtropical Asia.

***Elaphoglossumaubertii* (Desv.) T.Moore** – Habit: epiphyte herb. Habitat: Montane forest, 1500–2600 m. Vouchers: Katende T; Sheil D 1075 (K), Dummer RA 3480 (K). Native distribution range: Tropical & S Africa, W Indian Ocean.

***Elaphoglossumdeckenii* (Kuhn) C.Chr.** – Habit: Herb. Habitat: Forest, bamboo to ericaceous zone, 2200–3300 m. Vouchers: Wesche K 1278 (K), Wood GHS 270 (K). Native distribution range: Ethiopia to S Tropical Africa, Comoros, Madagascar.

***Elaphoglossumhybridum* (Bory) T.Moore** – Habit: Herb. Habitat: Moist montane forest, moorland, 2000–3600 m. Voucher: Hedberg O 904 (K, S). Native distribution range: Cameroon to Ethiopia and S Africa, Comoros, Madagascar, Réunion, Mauritius.

***Elaphoglossumsubcinnamomeum* Hieron.** – Habit: Herb. Habitat: Moorland, ericaceous zone, 3000–3600 m. Voucher: Hedberg O 993 (K). Native distribution range: Cameroon, Kenya to Tanzania.

***Lepisorusexcavatus* (Bory ex Willd.) Ching** – Habit: Epiphytic herb. Habitat: Moist montane forest, 2378–3050 m. Vouchers: Wesche K 1276 (US), Schippers RR K 185 (WAG). Native distribution range: Tropical & S Africa, W Indian Ocean.

***Loxogrammeabyssinica* (Baker) M.G.Price** – Habit: Herb. Habitat: Dry, wet forest, 1000–2500 m. Vouchers: Kisalye N; van Heist M 499 (K), Wesche K 630 (US), Tiyoy L 1243 (K), Eggeling W 2456 (US), Granvik H 365 (S). Native distribution range: Tropical Africa, Comoros, Madagascar, N Yemen.

***Melpomeneflabelliformis* (Poir.) A.R.Sm. & R.C.Moran** – Habit: Herb. Habitat: Forest, moorland, bamboo zone, 2230–3500 m. Voucher: Wesche K 1217 (US), Molesworth-Allen BEG 3668 (EA, US). Native distribution range: Mexico (Oaxaca, Chiapas) to Tropical America, Tropical & S Africa, W Indian Ocean.

***Nephrolepisbiserrata* (Sw.) Schott** – Habit: Herb. Habitat: Moist upland forest, 1000–1500 m. Voucher: Chater-Jack 25 in CM 5848 (EA). Native distribution range: Tropics & Subtropics.

***Nephrolepisundulata* J.Sm.** – Habit: Herb. Habitat: Swamps, marshy grassland, bushland, forest, rocky cliffs, 750–2450 m. Voucher: Lugard 285 (EA). Native distribution range: Tropics & Subtropics.

***Pleopeltismacrocarpa* (Bory ex Willd.) Kaulf.** – Habit: Herb. Habitat: Moist forest, riverine forest, bamboo forest, moorland, 1099–3600 m. Vouchers: Tweedie 1820 (US), Wesche K 553 (US), Bamps PRJ 6491 (BR), Szczepanek K 278 (BR), Molesworth-Allen BEG 3681 (US). Native distribution range: Mexico to S Tropical America, Tropical Africa, Arabian Peninsula, S India, Sri Lanka.

***Polystichummagnificum* F.Ballard** – Habit: Herb. Habitat: Moorland, 3300–3750 m. Vouchers: Hedberg O 965 (K), Wesche K 1419 (US). Native distribution range: S Ethiopia to Tanzania.

^**EX**^***Polystichumsinense* Christ** – Habit: Herb. Habitat: Moist montane forest, streamsidde, bamboo zone, moorland, 2400–3800 m. Vouchers: RB Faden 71/464 (K), Granvik H 361 (S). Native distribution range: S Africa, Pakistan to China, Taiwan.

***Polystichumtransvaalense* N.C.Anthony** – Habit: Herb. Habitat: Moist forest, bamboo zone, 1500–2700 m. Vouchers: Tiyoy L 1241 (WAG, K). Native distribution range: Cameroon to Kenya and S Africa.

#### F10. PTERIDACEAE

8 Genera, 12 species

***Actiniopterissemiflabellata* Pic.Serm.** – Habit: Herb. Habitat: Open bushland, woodland, 600–2100 m. Vouchers: Tweedie EM 1838 (K). Native distribution range: Africa to Nepal.

***Adiantumpoiretii* Wikstr.** – Habit: Herb. Habitat: Montane forest, forest/bamboo boundary, 1625–3000 m. Vouchers: Schippers RR K 226 (WAG), Hedberg O 65 (K), Roscoe J sn (K), Brown EJ EA12489 (K), Tweedie 1883 (K), Tweedie 1883A (K), Wesche K 1360 (K). Native range: Tropical & Subtropical America, Nigeria to Ethiopia and S Africa, SW Arabian Peninsula, W Indian Ocean, SW India, Sri Lanka.

***Anogrammaleptophylla* (L.) Link** – Habit: Herb. Habitat: Forest edges and slopes, moorland, 1500–3400 m. Vouchers: Lewin NH 304 (EA), Lye KA; Katende AB 6406 (K), Hamilton 6627 (MO), Irwin PH 304 (K), Snowden JD 512 (EA). Native distribution range: S Central Europe to Tropics & Subtropics.

***Coniogrammeafricana* Hieron.** – Habit: Herb. Habitat: Stream sides, waterfall spray zones, 2134–2900 m. Vouchers: Katende T; Sheil D 359 (K), Katende; Sheil 1869 (K). Native distribution range: Tropical Africa.

**^EX^*Doryopterisconcolor* (Langsd. & Fisch.) Kuhn** – Habit: Herb. Habitat: Forest, 1000–2300 m. Voucher: Tweedie 2435 (EA). Native distribution range: S Mexico to Tropical America.

***Haplopterisvolkensii* (Hieron.) E.H.Crane** – Habit: Epiphytic herb. Habitat: Moist montane forest, 1750–2255 m. Voucher: Snowden JD 950 (K). Native distribution range: Ethiopia to S Tropical Africa.

***Hemionitiscalomelanos* (Sw.) Christenh.** – Habit: Herb. Habitat: Roadsides, rock crevices, 1350–2700 m. Voucher: Hemp A 5419 (US). Native distribution range: NE Spain, Ethiopia to S Africa, W Indian Ocean, Pakistan to China (S Sichuan, Yunnan).

***Hemionitisfarinosa* (Forssk.) Christenh.** – Habit: Herb. Habitat: Bamboo zone, moorland, 2150–3000 m. Vouchers: Lugard EJ 295 (K), Hedberg O 178 897(K), Wesche K 1048 (US), Granvik H 359 (S), Gardner HM 1035 (K). Native distribution range: Mexico to Venezuela and Peru, Africa to Arabian Peninsula.

***Hemionitisquadripinnata* (Forssk.) Christenh.** – Habit: Herb. Habitat: Roadside, banks, moist montane forests, bamboo zones, 1525–2890 m. Voucher: Sheil D; Katende T 775 (K), Wesche K 699 (US). Native distribution range: Tropical & S Africa, Comoros, Madagascar, Mauritius, N Yemen.

***Hemionitisviridis* (Forssk.) Christenh.** – Habit: Herb. Habitat: Rock outcrop, bushland, shady riverines, forested areas, 650–2250 m. Vouchers: Katende T; Sheil D 630 (K), Tweedie 1831 (EA). Native distribution range: Ethiopia to S Africa, W Indian Ocean, SW Arabian Peninsula.

***Pteriscretica* L.** – Habit: herb. Habitat: Montane forest, 1500–2700 m. Voucher: Agnew ADQ (2013) (REF). Native distribution range: Canary Islands (Gran Canaria), Ethiopia to S Africa, Réunion, Madagascar, St Helena, Ascension, Socotra, Yemen, Mediterranean to Japan and Tropical Asia.

***Pterisfriesii* Hieron.in Fries** – Habit: Herb. Habitat: Forest, swamps, bamboo zones, 1000–3050 m. Voucher: Taylor G 3415 (NHMUK). Native distribution range: Tropical & S Africa.

#### F11. THELYPTERIDACEAE

1 Genus, 5 species

***Thelypterisbergiana* (Schltdl.) Ching** – Habit: Herb. Habitat: By streams, rivers in moist montane forest, bamboo zones, 1800–2470 m. Voucher: Molesworth-Allen BEG 3680 (US). Native distribution range: Tropical & S Africa, W Indian Ocean.

***Thelypterisdentata* E.P.St.John** – Habit: Herb. Habitat: Along streams, moist forest, 2100 m. Voucher: Agnew ADQ (2013) (REF). Native distribution range: Tropical & Subtropical Old World to Pacific.

***Thelypterisoppositiformis* (C.Chr.in Bonap) Ching** – Habit: Herb. Habitat: By streams in Bamboo zone, ericaceous zone, montane forest, 1900–2950 m. Voucher: Wesche K 1302 (US). Native distribution range: Nigeria to Ethiopia and S Africa, Madagascar.

**^EX^*Thelypterispozoi* (Lag.) Morton** – Habit: Herb. Habitat: Bamboo zone, moist montane forest, ericaceous zone, 2050–3350 m. Voucher: Agnew ADQ (2013) (REF). Native distribution range: Azores, Madeira, SW France to Spain.

***Thelypterispulchra* (Bory ex Willd.) Schelpe** – Habit: Herb. Habitat: Riverine forest 1465–2135 m. Voucher: Agnew ADQ (2013) (REF). Native distribution range: Nigeria to Ethiopia and Mpulamanga, W Indian Ocean.

#### F12. WOODSIACEAE

2 Genera, 2 species

***Athyriumschimperi* Moug.; Fee** – Habit: Herb. Habitat: Montane forest edge, among rocks in montane grasslands, 1820–3050 m. Voucher: Tweedie 1882 (US). Native distribution range: Tropical & S Africa, Madagascar.

***Cystopterisfragilis* (L.) Bernh.** – Habit: Herb. Habitat: Bamboo zone, 1400–4300 m. Vouchers: Wesche K 1918 (US), Beentje HJ 1959 (WAG). Native distribution range: Cosmopolitan.

### ﻿Part 3 Pinopsida

#### F13. CUPRESSACEAE

1 Genus, 1 species

***Juniperusprocera* Hochst. ex Endl.** – Habit: Tree. Habitat: Drier upland forest, 1800–2950 m. Vouchers: Wesche K 2025 (K), Snowden JD 930 (BR), 844 (EA, MO), Styles BT 292 (K), Lugard EJ; Lugard C 408 (K), Bamps PRJ 6511 (WAG, MO), Thomas AS 2630 (MO, EA). Native distribution range: Eritrea to Zimbabwe, SW Arabian Peninsula.

#### F14. PODOCARPACEAE

2 Genera, 3 species

^**EX**^***Afrocarpusfalcatus* (Thunb.) C.N.Page** – Habit: Tree. Habitat: Forest, 1250–2700 m. Vouchers: EA. Native distribution range: Malawi to S Africa.

***Afrocarpusgracilior* (Pilg.) C.N.Page** – Habit: Tree. Habitat: Forest, 1500–2400 m. Vouchers: Vesey-Fitzgerald in C.M 18617 (EA), Eggeling 2474 (EA), Wesche K 2024 (K), Bamps PRJ 6496 (WAG), Hedberg O 320 (K), Mwangangi OM 429 (BR), Styles BT 269 (BR), Eggeling WJ 2479 (BR), 2474 (K). Native distribution range: Ethiopia to E Tropical Africa.

***Podocarpusmilanjianus* Rendle** – Habit: Tree. Habitat: Upland forest, 1500–3350m. Vouchers: Wesche K 616 (K), Katende T; Sheil D 325 (K), Wood GHS 914 (K), Snowden JD 964 (EA, K, US), Dummer RA 3623 (US), Rono et al. SAJIT-PR 0229 (EA, HIB). Native distribution range: Nigeria to S Sudan and S Tropical Africa.

### ﻿Part 4 Liliopsida

#### F15. ALISMATACEAE

1 Genus, 1 species

***Alismaplantago-aquatica* L.** – Habit: Herb. Habitat: Forest, streamside, 1450–2340 m. Vouchers: Tweedie 730 (EA). Native distribution range: Temperate Eurasia, North Africa to Tanzania

#### F16. AMARYLLIDACEAE

4 Genera, 6 species

*Ammocharisangolensis (Baker) Milne-Redh. – Habit: Herb. Habitat: Outcrop rock, 1800–2600 m. Vouchers: Lugard 421 (EA), Snowden JD 1015 (K), 1055 (EA). Native distribution range: Uganda to Angola.

***Boophonedisticha* Herb.** – Habit: Herb. Habitat: Rocky grassland, 1280–2700 m. Vouchers: Tweedie EM (1976) (REF), Agnew ADQ (2013) (REF). Native distribution range: SE South Sudan to S Africa

***Crinumkirkii* Baker** – Habit: Herb. Habitat: Grassland, bushlands, 1750–2250 m. Voucher: Tweedie EM (1976) (REF). Native distribution range: E Tropical Africa to Mozambique.

***Crinummacowanii* Baker** – Habit: Herb. Habitat: Upland grasslands, 1630–2310 m. Voucher: Snowden JD 827 (EA). Native distribution range: Eritrea to S Africa, Seychelles.

**^EX^*Crinumzeylanicum* (L.) L.** – Habit: Herb. Habitat: Grassland, riverine, old cultivations, 1000–1900 m. Voucher: Wahlstrom 3 (EA). Native distribution range: Seychelles, SW India, Sri Lanka.

***Scadoxusmultiflorus* (Martyn) Raf.** – Habit: Herb. Habitat: Montane forest, grassland, 1750–2700 m. Vouchers: Snowden JD 846 (EA), Holm Å 20 (K). Native distribution range: Tropical & S Africa to SW Arabian Peninsula.

#### F17. ARACEAE

3 Genera, 5 species

***Amorphophallusabyssinicus* (A.Rich.) N.E.Br.** – Habit: Herb. Habitat: Wooded grasslands, marshland, 1000–2300 m. Vouchers: Lugard 581, Tweedie 2591 (K). Native distribution range: Tropical & S Africa.

***Arisaemaenneaphyllum* Hochst. ex A.Rich.** – Habit: Herb. Habitat: Montane forest edges, bamboo zone, 2300–3000 m. Vouchers: Tweedie 1167 (K), Tweedie 1167B (2/3) (K), Tweedie EM 1167A (K), Osmaston 4010 (EA), Hedberg 1056 (EA), Rono et al. SAJIT-PR 0032 (EA, HIB). Native distribution range: Ethiopia to Uganda, Yemen.

***Arisaemamildbraedii* Engl.** – Habit: Herb. Habitat: Moist montane forest, bamboo zone, 1400–2620 m. Vouchers: EjJ & C Lugard (EA), Snowden JD 895 (EA), Rono et al. SAJIT-PR 0045, 0101 (EA, HIB). Native distribution range: E Central & E Tropical Africa.

***Arisaemaschimperianum* Schott** – Habit: Herb. Habitat: Forest, 2250–3000 m. Voucher: Tweedie EM (1976) (REF). Native distribution range: Ethiopia to E DR Congo.

***Sauromatumvenosum* (Aiton) Kunth** – Habit: Herb. Habitat: Moist woodland, riverine forest, 230(1000)–2140 m. Vouchers: Chater-Jack 245 (K), Major EJ; Lugard C 580 (K), Anderson 2773 (K). Native distribution range: Upland areas of Africa and Asia

#### F18. ASPARAGACEAE

10 Genera, 34 species

***Albucaabyssinica* Jacq.** – Habit: Herb. Habitat: Grassland, bushland, 0010–2500 m. Voucher: Polhill 399 (EA). Native distribution range: Tropical & S Africa, SW Arabian Peninsula.

***Albucavirens* (Lindl.) J.C.Manning & Goldblatt** – Habit: Herb. Habitat: Open woodland, seasonally wet grassland, 1750–3000 m. Vouchers: Tweedie 511 (K), Polhill, R 415 (K). Native distribution range: Eritrea to S Africa.

***Anthericumangustifolium* Hochst.** – Habit: Herb. Habitat: Grasses, seasonally waterlogged soils, 1800–2850 m. Vouchers: Naiga; Katende 700 (K), Symes 358 (K), Major EJ; Lugard C 592 (K), Irwin PH Mrs 69 (K), Tweedie 3449 (K), Chater-Jack 264 (K). Native distribution range: Eritrea to Kenya.

***Asparagusafricanus* Lam.** – Habit: Erect or climbing woody shrub. Habitat: Forest, woody bushes, grassland, 100–3500 m. Vouchers: Dummer RA 3737 (EA). Native distribution range: Tropical & S Africa, Arabian Peninsula, W India.

**^EX^*Asparagusasiaticus* L.** – Habit: Scrambling or woody climbing shrub. Habitat: Wet savanna, 1750–2250 m. Voucher: Tweedie EM (1976) (REF). Native distribution range: India.

***Asparagusasparagoides* (L.) Druce** – Habit: Climbing herb. Habitat: woodland, dry forest, forest margin, 1750–3000 m. Voucher: Wesche K 1311 (EA). Native distribution range: S Ethiopia to S Africa.

***Asparagusbuchananii* Baker** – Habit: Scrambling or woody climbing shrub. Habitat: Grassland, wooded grassland, thickets, woodland, 150–2250 m. Voucher: Snowden JD 1064 (EA, WAG). Native distribution range: SW Ethiopia to S Africa.

***Asparagusflagellaris* Baker** – Habit: Shrub. Habitat: Forest, 500–1000 m. Voucher: Agnew ADQ (2013) (REF). Native distribution range: Tropical & S Africa, W Arabian Peninsula.

***Asparagusracemosus* Willd.** – Habit: Scrambling or woody climbing shrub. Habitat: Forest margins, bushlands, 800–2900 m. Voucher: Snowden JD 1036 (EA). Native distribution range: Tropical Africa to N Australia.

**BowieavolubilisHarv. ex Hook.f.subsp.volubilis** – Habit: Herb. Habitat: Wet savanna, 1750–2250 m. Voucher: Tweedie EM (1976) (REF). Native distribution range: Uganda to S Africa.

***Bowieavolubilis* Harv. ex Hook.f.** – Habit: Herb. Habitat: Evergreen bushland, rocky woodland, 1680–2320 m. Vouchers: Rono et al. SAJIT-PR 0153 (EA, HIB). Native distribution range: Uganda to S Africa.

**Chlorophytumaffinevar.curviscapum (Poelln.) Hanid** – Habit: Herb. Habitat: Outcrop rock, 1800–2400 m. Voucher: Raymer 611 (EA). Native distribution range: Tropical Africa, Yemen.

**Chlorophytumafricanumvar.silvaticum (Dammer) Meerts** – Habit: Herb. Habitat: Grassland, rock outcrop, open woodlad, 1800–2400 m. Vouchers: Tweedie EM (1976) (REF), Agnew ADQ (2013) (REF). Native distribution range: S Ethiopia to S Tropical Africa.

***Chlorophytumblepharophyllum* Schweinf. ex Baker** – Habit: Herb. Habitat: Woodland, grassland, rocky outcrops, 1500–2450 m. Voucher: Polhill RM 413 (BR). Native distribution range: Tropical Africa.

***Chlorophytumcameronii* (Baker) Kativu** – Habit: Herb. Habitat: Grassland, rocky slopes, 1675–2550 m. Vouchers: Symes 373 (EA), Rayner WR 544 (EA), Lugard EJ 2804 (EA), Jack C 253 (EA), Padwa JH 22 (EA), Tweedie EM 5452 (EA), Adamson J 508 (508). Native distribution range: Tropical Africa.

***Chlorophytumcomosum* (Thunb.) Jacques** – Habit: Herb. Habitat: Forest, outcrop rock, 1800–3000 m. Voucher: Lugard EJ; Lugard C 629 (K). Native distribution range: W Tropical Africa to Cameroon, Ethiopia to S Africa.

***Chlorophytumgallabatense* Schweinf. ex Baker** – Habit: Herb. Habitat: Grassland, open woodland, 750–2439 m. Vouchers: Tweedie EM 1998 (K), Polhill RM 410 (BR), Tweedie DR 1354 (BR). Native distribution range: Tropical Africa to Namibia.

***Chlorophytumpolystachys* Baker** – Habit: Herb. Habitat: Outcrop rock, open woodland, 1750–2900 m. Voucher: Tweedie EM 1354 (K). Native distribution range: Tropical Africa.

***Chlorophytumsubpetiolatum* (Baker) Kativu** – Habit: Herb. Habitat: Outcrop rock, grassland, woodland, 1750–2450 m. Voucher: Polhill 398 (K). Native distribution range: Tropical Africa.

***Chlorophytumviridescens* Engl.** – Habit: Herb. Habitat: Rock outcrops, grassland, riverbanks, 1750–2700 m. Voucher: Polhill 410 (EA). Native distribution range: Cameroon, E Tropical Africa.

***Dracaenaafromontana* Mildbr.** – Habit: Shrub or tree. Habitat: Moist forest, bamboo forest, 1600–3200 m. Vouchers: Lye KA; Pocs T 23128 (K), Dummer RA 3604 (K), Snowden JD 804 (EA, K), Tiyoy LM 1280 (K), Rono et al. SAJIT-PR 0175 (EA, HIB). Native distribution range: South Sudan (Imatong Mountains) to Malawi.

***Dracaenaconspicua* (N.E.Br.) Byng & Christenh.** – Habit: Herb. Habitat: Outcrop rock, 1800–2400 m. Voucher: Tweedie 1441 (K). Native distribution range: Kenya to S Tropical Africa.

***Dracaenafragrans* (L.) Ker Gawl.** – Habit: Shrub or tree. Habitat: Forest, near streams, 600–2250 m. Vouchers: Jackson THE 338 (K)? Jack? 5195 (K). Native distribution range: Tropical Africa.

***Dracaenafrequens* (Chahin.) Byng & Christenh.** – Habit: Herb. Habitat: Bushland, rocky sites, secondary grassland, 50–2600 m. Voucher: Tweedie 1441 (EA). Native distribution range: Ethiopia to E Tropical Africa.

***Dracaenasteudneri* Engl.** – Habit: Tree. Habitat: Moist forest, 2250–3000 m. Voucher: Jack C 427 (EA). Native distribution range: Ethiopia to S Tropical Africa.

***Drimiaaltissima* (L.f.) Ker Gawl.** – Habit: Herb. Habitat: Wet savanna, grassland, 1750–2250 m. Voucher: Lugard EJ; Lugard C 558 (EA). Native distribution range: Tropical & S Africa.

*Drimiacongesta Bullock – Habit: Herb. Habitat: Grassland, rocky outcrop, moorland, 2800–3000 m. Vouchers: Lugard EJ; Lugard C 474 (K), Thoma AS 2628 (MO). Native distribution range: Uganda to Kenya.

***Drimiaelata* Jacq.** – Habit: Herb. Habitat: Wet savanna, grassland, 1250–2500 m. Vouchers: O’Brien (EA), Lugard EJ; Lugard C 563 (K). Native distribution range: South Sudan to S Africa.

*Drimiaporphyrantha (Bullock) Stedje – Habit: Herb. Habitat: Grassland, 2000–2300 m. Voucher: Lugard EJ; Lugard C 556 (K). Native distribution range: Kenya.

***Eriospermumabyssinicum* Baker** – Habit: Herb. Habitat: Wet rocky grassland, 2000–2500 m. Vouchers: Tweedie EM (1976) (REF), Agnew ADQ (2013) (REF). Native distribution range: Tropical & S Africa.

***Eriospermumtriphyllum* Baker** – Habit: Herb. Habitat: Grassland, outcrop rock, 1800–2400 m. Voucher: Tweedie 906 (EA). Native distribution range: S Ethiopia to S Tropical Africa.

***Ledebouriarevoluta* (L.f.) Jessop** – Habit: Herb. Habitat: Outcrop rock, bushland, grassland, woodland, 1800–2700 m. Vouchers: Tweedie EM (1976) (REF), Agnew ADQ (2013) (REF). Native distribution range: Eritrea to S Africa, SW Arabian Peninsula, India, Sri Lanka.

***Ornithogalumgracillimum* R.E.Fr.** – Habit: Herb. Habitat: Grassland, 2000–3000 m. Vouchers: Polhill RM 406 (EA, BR), Rono et al. SAJIT-PR 0110 (EA, HIB). Native distribution range: S Ethiopia to Kenya.

^**EX**^***Ornithogalumgussonei* Ten.** – Habit: Herb. Habitat: Grassland, bushland, woodland, swampy regions, 1000–3000 m. Voucher: Polhill 415 (EA). Native distribution range: Italy, Greece to SW Turkey.

#### F19. ASPHODELACEAE

3 Genera, 7 species

**Aloeelgonica Bullock – Habit: Shrub. Habitat: Grass covered slopes, rock pockets, 1980–2400 m. Vouchers: Tweedie 2406 (EA), Tweedie EM 343 (K), Brandham 98 (MO), Brandham PE; Cutler DF 11 (K), Lugard 299 (K), Brandham PE; Cutler DF 94 (K), Reynolds GW 8003 (EA), Rono et al. SAJIT-PR 0089 (EA, HIB). Native distribution range: Kenya (Mt Elgon).

*Aloewilsonii Reynolds – Habit: Herb. Habitat: Rocky slopes, cliffs, 1525–3000 m. Vouchers: Tweedie DR 1424 (K), Reynolds 9041 (K). Native distribution range: N Uganda to NW Kenya.

*Aloewollastonii Rendle – Habit: Shrub. Habitat: Grassland, woodland, 1750–2285 m. Vouchers: Tweedie 322 (EA), Irwin PH 355 (EA). Native distribution range: E Central Tropical Africa.

***Bulbineabyssinica* A.Rich.** – Habit: Herb. Habitat: Grassland, outcrop rock, 2000–3200 m. Vouchers: Hedberg O (UPS), Rono et al. SAJIT-PR 0107 (EA, HIB). Native distribution range: Ethiopia to S Africa, Yemen.

***Kniphofiagrantii* Baker** – Habit: Herb. Habitat: Damp grassland, 750–2700 m. Voucher: Snowden JD 847 (EA). Native distribution range: Uganda to Zambia.

***Kniphofiathomsonii* Baker** – Habit: Herb. Habitat: Forest, bushland, grassland, marshy grounds, 1900–3960 m. Vouchers: Hedberg O 4467 (EA), Snowden JD 944 (EA), Wright CH 5455 (EA), Lugard EJ 83 (EA), Rono et al. SAJIT-PR 0149 (EA, HIB). Native distribution range: Ethiopia to N Tanzania.

**Kniphofiathomsoniivar.snowdenii (C.H.Wright) Marais – Habit: Herb. Habitat: Montane grassland, bushland, 2100–4000 m. Vouchers: Tothill BH 2284 (EA), Hedberge 4467 (EA), Tweedie 1279 (EA), Snowden JL 437 (K). Native distribution range: Uganda to Kenya.

#### F20. COLCHICACEAE

4 Genera, 5 species

**^EX^*Androcymbiummelanthioides* Willd.** – Habit: Herb. Habitat: Outcrop rock, 1800–2400 m. Voucher: Tweedie EM (1976) (REF). Native distribution range: Zimbabwe to S Africa.

***Colchicumstriatum* (Hochst. ex A.Rich.) J.C.Manning & Vinn.** – Habit: Herb. Habitat: Rock outcrop, evergreen woodland, bushland, grassland, 1500–3480 m. Voucher: Symes 378 (EA). Native distribution range: Eritrea to S Africa.

***Gloriosasuperba* L.** – Habit: Herb. Habitat: Wet savanna, outcrop rock, woodland, evergreen forests and bushlands, 1750–2400 m. Vouchers: Cheseny CM 9 (BR), Lindblom G sn (S). Native distribution range: Widespread in tropical and southern Africa and in tropical Asia.

***Wurmbeatenuis* (Hook.f.) Baker** – Habit: Herb. Habitat: Upland grassland, forests, 2000–3500 m. Vouchers: Hamilton A; Wendelbo P 6616 (K), Rono et al. SAJIT-PR 0144 (EA, HIB). Native distribution range: Tropical & S Africa.

*Wurmbeatenuissubsp.hamiltonii (Wendelbo) B.Nord. – Habit: Herb. Habitat: Upland grassland, rock outcrop, 2000–3500 m. Vouchers: Hamilton A; Wendelbo P 6616 (K), Rono et al. SAJIT-PR 0020 (EA, HIB), Tweedie 575 (EA). Native distribution range: E Tropical Africa.

#### F21. COMMELINACEAE

4 Genera, 27 species

***Aneilemahirtum* A.Rich.** – Habit: Herb. Habitat: Grassland, woodland, 1750–2050 m. Vouchers: Tweedie 76B,175 (EA), Tweedie EM 464 (K). Native distribution range: Ethiopia to Zambia.

***Aneilemaleiocaule* K.Schum.** – Habit: Herb. Habitat: Upland Forest, forest edges, 1000–2750 m. Voucher: Tweedie EM 752 (K). Native distribution range: S Ethiopia to E DR Congo.

*Aneilemaminutiflorum Faden – Habit: Herb. Habitat: Montane, riverine forest, 1800–2500 m. Vouchers: Lewis WH 5973 (US), Hemp A 5442 (US), Wesche K 1965 (US). Native distribution range: E Tropical Africa.

***Aneilemaspekei* C.B.Clarke** – Habit: Herb. Habitat: Bushland, grassland, woodland, roadsides, 700–1800 m. Vouchers: Tweedie EM 759, 1885, (K). Native distribution range: Ethiopia to Zambia.

***Commelinaafricana* L.** – Habit: Herb. Habitat: Grassland, weed in disturbed places, 300–2900 m. Vouchers: Tweedie 785 (K), Wesche K 568 (K). Native distribution range: Africa, Arabian Peninsula.

^**EX**^**Commelinaafricanavar.krebsiana (Kunth) C.B.Clarke** – Habit: Herb. Habitat: Upper Forest edge, 3000–3150 m. Vouchers: Lind EM 460 (K), Tweedie 1392 (K). Native distribution range: S Tropical & S Africa, Comoros.

***Commelinabenghalensis* L.** – Habit: Herb. Habitat: Bushland, woodland, moist grassland, cultivation, 010–2350 m. Vouchers: Tweedie 787 (K), Lewis WH 5961 (US). Native distribution range: Tropical & Subtropical Old World.

***Commelinadiffusa* Burm.f.** – Habit: Herb. Habitat: Woodland, swamps, forest clearings, caltivations, 1750–3400 m. Voucher: Wesche K 1853 (US). Native distribution range: Tropical & Subtropical Old World.

**Commelinaecklonianasubsp.echinosperma (K.Schum.) Faden** – Habit: Herb. Habitat: Rocky grassland, 1050–2050 m. Voucher: Tweedie 1350, 3444 (EA). Native distribution range: Ethiopia to Zambia.

**Commelinaerectasubsp.erecta** – Habit: Herb. Habitat: Wet grassland, outcrop rock, 550–1500 m. Voucher: Tweedie EM 4165 (EA). Native distribution range: America, Tropical & S Africa, Arabian Peninsula.

**Commelinakitaleensis Faden – Habit: Herb. Habitat: Swamps, seasonally wet grassland, forest edge, 1800–2000 m. Vouchers: Irwin 146 (EA), Tweedie EM EM; Faden RB; Evans A 69/715 (K). Native distribution range: Kenya (E Mt Elgon).

***Commelinalatifolia* Hochst. ex A.Rich.** – Habit: Herb. Habitat: Wet savanna, among rocks, forests, 1100–2100 m. Vouchers: Faden RB & Evans AJ 69/714 (US). Native distribution range: Congo to Ethiopia and N Tanzania, SW Arabian Peninsula.

***Commelinapurpurea* C.B.Clarke** – Habit: Herb. Habitat: Wooded grassland, woodlands, forest edge, outcrop rock, 1800–2250 m. Vouchers: Lugard 145 (K), Wesche K 1507 (US), Faden RB & Evans AJ 69/737B (US), Lewis WH 5960 (US), Rono et al. SAJIT-PR 0023 (EA, HIB). Native distribution range: Congo to South Sudan and Tanzania.

***Commelinareptans* Brenan** – Habit: Herb. Habitat: Outcrop rock, seasonally moist grasslands, 1800–2550 m. Voucher: Tweedie EM (1976) (REF). Native distribution range: S Ethiopia to Burundi and N Tanzania.

***Commelinaschweinfurthii* C.B.Clarke** – Habit: Herb. Habitat: grassland, rocky araes, swamps, forests edges, roadside, 1750–2350 m. Vouchers: Lewis WH 5974, Rono et al. SAJIT-PR 0022 (EA, HIB). Native distribution range: Tropical Africa.

**Commelinaschweinfurthiisubsp.schweinfurthii** – Habit: Herb. Habitat: Wet savanna, 1750–2250 m. Vouchers: Lugard EJ; Lugard C 549 (K), Tweedie 786 (K), Lewis WH 5974 (US), Faden RB & Evans AJ 69/717 (US). Native distribution range: Tropical Africa.

***Commelinasubulata* Roth** – Habit: Herb. Habitat: Forest, rock outcrop, wet grasslands, bushlands, 1750–2300 m. Voucher: Lewis WH 5975 (US). Native distribution range: Tropical & S Africa, Arabian Peninsula, S India.

***Commelinatriangulispatha* Mildbr.** – Habit: Herb. Habitat: Moist roadside banks, damp pastures, wooded grasslands and woodland, 1650–2300 m. Voucher: Thomas 414 (EA). Native distribution range: DR Congo to E Tropical Africa.

***Cyanotisarachnoidea* C.B.Clarke** – Habit: Herb. Habitat: outcrop rock, 1800–2600 m. Vouchers: Rono et al. SAJIT-PR 0027, SAJIT-PR 0128 (EA, HIB). Native distribution range: W Tropical Africa to Tanzania, India to Taiwan.

***Cyanotiscaespitosa* Kotschy & Peyr.** – Habit: Herb. Habitat: Grassland, rock outcrop, 2150–2600 m. Voucher: Agnew ADQ (2013) (REF). Native distribution range: Tropical Africa.

***Cyanotisfoecunda* DC. ex Hassk.** – Habit: Herb. Habitat: Outcrop rock near rivers, bushland, woodlands, occasionally in swampy places, 1800–2400 m. Vouchers: Tweedie EM 3466 (K), Lewis WH 5964 (US). Native distribution range: Cameroon to Ethiopia and N Botswana, Arabian Peninsula.

***Cyanotislanata* Benth.** – Habit: Herb. Habitat: Grassland, woodland, 1750–2150 m. Vouchers: Tweedie EM 782 (K), Lewis WH 5976 (US), Rono et al. SAJIT-PR 0142 (EA, HIB). Native distribution range: Tropical & S Africa, Arabian Peninsula.

***Cyanotislongifolia* Benth.** – Habit: Herb. Habitat: Wet savanna, grassland, bamboo clumps, 1750–2250 m. Voucher: Stein W Bie (UPS). Native distribution range: Tropical Africa to NE Namibia.

***Cyanotispaludosa* Brenan** – Habit: Herb. Habitat: Marshes, open grassland., 1100–2000 m. Vouchers: Tweedie 3655 (EA), Faden RB & Evans AJ 69/716 (US). Native distribution range: DR Congo to E Tropical Africa.

***Cyanotisvaga* (Lour.) Schult. & Schult.f.** – Habit: Herb. Habitat: Grassland, forest edge clearing, moorland, rock outcrop, 1650–4150 m. Vouchers: Tweedie Mrs 1420, 1351 (K), Wesche K 1800, 1655 (US), Dummer RA 3383 (EA). Native distribution range: Tropical Africa, SW Arabian Peninsula, Himalaya to W Malesia.

***Murdanniasemiteres* (Dalzell) Santapau** – Habit: Herb. Habitat: Outcrop rock, 1800–2400 m. Vouchers: Tweedie EM 926 (K), Tweedie EM 161 (K). Native distribution range: Uganda to Zambia, Iran to Indo-China.

***Murdanniasimplex* (Vahl) Brenan** – Habit: Herb. Habitat: Grassland, bushland, woodland, 1750–2200 m. Vouchers: Lind EM 450 (EA), Tweedie EM 161 (K). Native distribution range: Tropical & S Africa, Madagascar, Tropical & Subtropical Asia to N. Australia.

#### F22. CYPERACEAE

7 Genera, 59 species

***Abildgaardiaovata* (Burm.f.) Kral** – Habit: Herb. Habitat: Grassland, 0–2000 m. Vouchers: Human observation sn (EA). Native distribution range: Tropics & Subtropics.

***Bulbostylisatrosanguinea* C.B.Clarke** – Habit: Herb. Habitat: Afroalpine grassland, rocky moorland, 2000–3700 m. Voucher: Wesche K 1369 (K). Native distribution range: Eritrea to Angola, Yemen.

***Bulbostyliscongolensis* De Wild.** – Habit: Herb. Habitat: Grassland, bushland, rocky outcrops, 1050–1400 m. Voucher: Human observation sn (EA). Native distribution range: Tropical Africa.

**Bulbostylisdensasubsp.afromontana (Lye) R.W.Haines** – Habit: Herb. Habitat: Upland grassland, forest, 1200–2600 m. Voucher: Lind EM 271 (EA). Native distribution range: Tropical & S Africa.

*Bulbostylisglaberrima Kük. – Habit: Herb. Habitat: Moorland rocks, 3000–3600 m. Vouchers: Hedberg 4545 (EA), Hamilton 233 (EA). Native distribution range: E Uganda to W Central Kenya.

***Bulbostylisoligostachys* C.B.Clarke** – Habit: Herb. Habitat: Wet rocks, wooded grassland, 1800–2500 m. Voucher: Wesche K 1422 (EA). Native distribution range: Eritrea to Angola.

***Bulbostylispilosa* (Willd.) Cherm.** – Habit: Herb. Habitat: Flooded wooded grassland, bushland, 1–1400 m. Voucher: Human observation sn (EA). Native distribution range: Tropical Africa.

***Carexbequaertii* De Wild.** – Habit: Herb. Habitat: Wet montane, alpine grasslands, 2000–3600 m. Vouchers: Wesche K 1208 (EA), Haines W 4165 (K), Lugard C; Verdcourt B 482 (K). Native distribution range: Ethiopia to S Africa.

***Carexcastanostachya* K.Schum. ex Kük.** – Habit: Herb. Habitat: Moist Forest, bamboo thickets, 1850–2050 m. Voucher: Human observation sn (EA). Native distribution range: Kenya (Taita Hills) to Tanzania.

***Carexconferta* Hochst. ex A.Rich.** – Habit: Herb. Habitat: Swamps, bamboo forest, moorland, upland forest, 2250–3650 m. Vouchers: Wesche K 77 (K), 1939 (K), Mwangangi OM 364 (BR), Hedberg O 1034 (K), Dummer RA 3461 (EA). Native distribution range: Ethiopia to E Tropical Africa.

***Carexechinochloe* Kunze** – Habit: Herb. Habitat: Woodland edges, grasslands, bamboo, marshes, 900–2750 m. Voucher: Agnew ADQ (2013) (REF). Native distribution range: Bioko to Ethiopia and Tanzania.

**Carexelgonensis Nelmes – Habit: Herb. Habitat: Heath zones, bamboo forest, 2400–3650 m. Voucher: Adamson J 493 (K, EA), Hedberg 4555 (EA), Wesche K 1170 (EA), Bogdan A 5409 (K), George Taylor 3474 (NHMUK), Lye, Katende & Swinscow 5741 (EA). Native distribution range: E Tropical Africa (Mt Elgon).

***Carexerythrorrhiza* Boeckeler** – Habit: Herb. Habitat: Streamside, heath zone, rock outcrops, 2400–3500 m. Vouchers: Bogdan 5425A, 5425B (EA), Thomas AS 2667 (EA), Wesche K 1947 (EA). Native distribution range: Ethiopia to E DR Congo and N Tanzania.

***Carexfischeri* K.Schum.** – Habit: Herb. Habitat: Moist Forest, Hagenia and bamboo zone, 21500–4300 m. Vouchers: Dummer RA 3465 (K, S), Lisowski S 10936 (BR). Native distribution range: Ethiopia to E Central and E Tropical Africa.

***Carexjohnstonii* Boeckeler** – Habit: Herb. Habitat: Forest, 2200–3300 m. Vouchers: Tothill BH 2266 (EA), Bogdan A 4130 (K), Smith SAL 185 (K). Native distribution range: Ethiopia to E Central & E Tropical Africa.

**Carexlycurussubsp.scabrida (Kük.) Verdc.** – Habit: Herb. Habitat: Swampy grassland, riverine montane forest, 2350–3300 m. Vouchers: Haines 4154 (EA), Bogdan 3935 (EA). Native distribution range: Cameroon, E Central, Tropical Africa (Mountains).

***Carexmannii* E.A.Bruce** – Habit: Herb. Habitat: Swamps in upland forest, 2450–3100 m. Vouchers: Liebenberg L 1708 (US), Dummer RA 3459 (US). Native distribution range: Bioko, SW Cameroon, Ethiopia to Burundi and Kenya.

***Carexmonostachya* A.Rich.** – Habit: Herb. Habitat: Alpine bushland, forest, moorland, upper bamboo forest, 2700–4321 m. Voucher: Hedberg 127 (K). Native distribution range: N Ethiopia to Tanzania (Mt Kilimanjaro).

***Carexpetitiana* A.Rich.** – Habit: Herb. Habitat: Forest, bamboo forest, grassland, heath by streams, 2400–3450 m. Vouchers: Wood G 121 (EA), Bogdan A 4088 (K), 5388 (K), 4129, Hedberg O 192 (K), 192 (K), Haines W 4164 (K), Trelawny BR 4343 (K), Mwangangi OM 373 (BR). Native distribution range: Nigeria to Cameroon, Ethiopia to Zimbabwe.

*Carexrunssoroensis K.Schum. – Habit: Herb. Habitat: Moorland, 2700–4100 m. Vouchers: Liebenberg L sn (US), Dummer RA 3361 (US), Bogdan 3934, 5413 (EA), Haines 4156 (EA). Native distribution range: NE DR Congo to Kenya (Mountains).

***Carexsimensis* Hochst. ex A.Rich.** – Habit: Herb. Habitat: Swampy grassland, montane forest, moorland, 1850–3900 m. Vouchers: Lugard 680 (EA), Herdberg 1043 (EA), Wesche K 1809 (EA). Native distribution range: Ethiopia to E DR Congo.

***Carexsteudneri* Boeckeler** – Habit: Herb. Habitat: Montane grassland, montane bushlands, bamboo zone, rock crevices, woodland, 2300–3050 m. Voucher: Agnew ADQ (2013) (REF). Native distribution range: Ethiopia to S Africa.

**Cyperusalatussubsp.albus (Nees) Lye** – Habit: Herb. Habitat: Dry bushlands, 500–2500 m. Voucher: Agnew ADQ (2013) (REF). Native distribution range: Tropical & S Africa, Seychelles.

***Cyperusalbosanguineus* Kük.** – Habit: Herb. Habitat: Seasonally wet grassland, moorland, seepages in rock crevices, 1550–4000 m. Vouchers: Granvik H 323 (S), Wood G 146 (EA), Hedberg O 43 (K). Native distribution range: South Sudan to E Tropical Africa.

***Cyperusamauropus* Steud.** – Habit: Herb. Habitat: Dry rocky grassland, 500–2000 m. Voucher: Agnew ADQ (2013) (REF). Native distribution range: Eritrea to Zimbabwe, Arabian Peninsula.

***Cyperusaromaticus* (Ridl.) Mattf. & Kük.** – Habit: Herb. Habitat: Streamside, forest margin, wet grassland, 1950–2400 m. Voucher: Agnew ADQ (2013) (REF). Native distribution range: Tropical & S Africa, W Indian Ocean.

***Cyperusassimilis* Steud.** – Habit: Herb. Habitat: Streamside, seasonal pools, rock outcrops, 250–2100 m. Voucher: Lind EM 236 (EA). Native distribution range: Eritrea to S Africa, Madagascar.

*Cyperusboreochrysocephalus Lye – Habit: Herb. Habitat: Dry wooded grassland, 1000–2200 m. Voucher: Mabberley 1128 (EA). Native distribution range: South Sudan to NW Tanzania.

***Cyperusbracheilema* (Steud.) Mattf. & Kük.** – Habit: Herb. Habitat: Woodland, 1500–3400 m. Voucher: Agnew ADQ (2013) (REF). Native distribution range: Eritrea to Tanzania, S Africa.

***Cyperusbrevifolius* (Rottb.) Endl. ex Hassk.** – Habit: Herb. Habitat: Swampy grassland, forest margins, 1600–3000 m. Voucher: Agnew ADQ (2013) (REF). Native distribution range: Tropics & Subtropics.

***Cyperuscostatus* Mattf. & Kük.** – Habit: Herb. Habitat: Dry rocky grassland, 1250–2900 m. Voucher: Agnew ADQ (2013) (REF). Native distribution range: Eritrea to S. Tropical Africa.

***Cyperuscyperoides* (L.) Kuntze** – Habit: Herb. Habitat: Grassland, woodland, 100–2200 m. Voucher: Agnew ADQ (2013) (REF). Native distribution range: Africa to Pacific.

***Cyperusderreilema* Steud.** – Habit: Herb. Habitat: Moist montane forest, bamboo forest, along streams, 2100–3050 m. Voucher: Wesche K 627 (EA). Native distribution range: Ethiopia to Malawi.

***Cyperusdistans* L.f.** – Habit: Herb. Habitat: Moist site, streamside in forest edges, cultivation, 1800–2100 m. Voucher: Human observation sn (EA). Native distribution range: Tropics & Subtropics.

***Cyperuselegantulus* Steud.** – Habit: Herb. Habitat: Swamps, riverine edges, moist forest margins, 1100–3050 m. Voucher: Beentje HJ 1961 (WAG). Native distribution range: Tropical & S Africa to Arabian Peninsula.

***Cyperusfischerianus* Schimp. ex A.Rich.** – Habit: Herb. Habitat: Moist montane, riverine forest, 1750–2650 m. Voucher: Lye & Katende 6410 (EA). Native distribution range: Eritrea to S Tropical Africa.

***Cyperusisolepis* (Nees ex Arn.) Bauters** – Habit: Herb. Habitat: Grassland, 1750–2000 m. Voucher: Gilbert & Mesfin 6549 (EA). Native distribution range: Tropical & Subtropical Old World.

*Cyperuskarisimbiensis (Cherm.) Kük. – Habit: Herb. Habitat: Woodland, alpine streamsides, 1850–3050 m. Voucher: Agnew ADQ (2013) (REF). Native distribution range: E Central & E Tropical African Mountains.

*Cyperuskerstenii Boeckeler – Habit: Herb. Habitat: Alpine moor, montane grassland, 2500–3600 m. Voucher: Lye 5750 (EA). Native distribution range: South Sudan to E Tropical African Mountains.

***Cyperusluteus* Boeckeler** – Habit: Herb. Habitat: Grassland, forest plantation, 2400–3600 m. Voucher: Agnew ADQ (2013) (REF). Native distribution range: SW Cameroon, Gabon, Uganda to Malawi, W Indian Ocean.

***Cyperusmapanioides* C.B.Clarke** – Habit: Herb. Habitat: Forest paths, 900–1800 m. Voucher: Agnew ADQ (2013) (REF). Native distribution range: Tropical Africa.

***Cyperusmaranguensis* K.Schum.** – Habit: Herb. Habitat: Grassland, swampy grasslands, cultivations, 800–2150 m. Voucher: Lye & Katende 6423 (EA). Native distribution range: S Ethiopia to Tanzania.

***Cyperusmelanospermus* (Nees) Valck.Sur.** – Habit: Herb. Habitat: Swampy grassland, streamside, 1000–2000 m. Voucher: Agnew ADQ (2013) (REF). Native distribution range: Tropical Old World.

***Cyperusmicroaureus* Lye** – Habit: clustered annual. Habitat: Bushlands, wet rocky outcrops, grassland, 1750–1950 m. Voucher: fide Haine & Lye,Napper (EA). Native distribution range: Ethiopia to Zambia.

***Cyperusmollipes* K.Schum.** – Habit: Herb. Habitat: Grasslands, 2100–2750 m. Voucher: Agnew ADQ (2013) (REF). Native distribution range: Central African Republic to Ethiopia and S Tropical Africa, India to Thailand.

***Cyperusniveus* Retz.** – Habit: Herb. Habitat: Grassland in rocky pockets, 100–2200 m. Voucher: Hedberg O 813 (K). Native distribution range: Tropical and S Africa to S Central China.

**Cyperusniveusvar.leucocephalus (Kunth) Fosberg** – Habit: Herb. Habitat: Dry grassland, recently burnt areas, 0–2100 m. Voucher: Dale IR 4117 (BR). Native distribution range: Tropical & S Africa, Aldabra, S Madagascar, Arabian Peninsula.

***Cyperusplateilema* (Steud.) Kük.** – Habit: Herb. Habitat: Mountain grassland, giant heath zones, roadside, moist forest, 1750–3650 m. Vouchers: Bogdan 5398 (EA), Wesche K 544 (EA). Native distribution range: Eritrea to N Tanzania, Yemen (J Ta’kar).

***Cyperusplatycaulis* Baker** – Habit: Herb. Habitat: Wet areas, swamps in bamboo zone, 1000–3000 m. Voucher: Naiga 419 (EA). Native distribution range: Chad to South Africa, Madagascar.

***Cyperusrigidifolius* Steud.** – Habit: Herb. Habitat: Swamps, bushlands, wet grassland, 1800–2100 m. Vouchers: Snowden JD 1030 (EA), Dummer RA 3627 (US), Padwa JH 31 (WAG). Native distribution range: Eritrea to S Africa, Arabian Peninsula.

***Cyperusrubicundus* Vahl** – Habit: Herb. Habitat: Wet grassland, rocky wooded grassland, 0–2300 m. Voucher: Human observation sn (EA). Native distribution range: Africa to India, Lesser Sunda Islands to N Australia.

**Cyperussesquiflorus(Torr.)Mattf. & Kük.subsp.sesquiflorus** – Habit: Herb. Habitat: Upper forest levels, 1300–3300 m. Voucher: Agnew ADQ (2013) (REF). Native distribution range: Tropics & Subtropics.

***Cyperususitatus* Burch. ex Roem. & Schult.** – Habit: Herb. Habitat: Rocky slopes and outcrop, flooded grassland, 800–2150 m. Voucher: Lye & Katende 6422 (EA). Native distribution range: Ethiopia to S Africa.

***Ficiniagracilis* Schrad.** – Habit: Herb. Habitat: Upland grassland, moorland, alpine grassland, 2400–4321 m. Voucher: Smith, Beentje & Muasya 179 (EA). Native distribution range: Uganda to S Africa.

***Isolepiscostata* Hochst. ex A.Rich.** – Habit: Herb. Habitat: Montane forest, stream banks, seepage area, 1700–3500 m. Vouchers: Lisowski S 10799 (EA), Dummer RA 3543 (US). Native distribution range: Ethiopia to S Africa, Madagascar.

***Isolepisfluitans* (L.) R.Br.** – Habit: Herb. Habitat: Montane forest, on or in shallow water, bog, 1200–3700 m. Vouchers: Dummer RA 3501 (US), Hedberg O (UPS). Native distribution range: Old World.

**Isolepisgraminoides (R.W.Haines & Lye) Lye – Habit: Herb. Habitat: Alpine bogs, 3200–3500 m. Vouchers: Hamilton 1418, 4319, Forbes 277 (EA). Native distribution range: E Tropical Africa (Ruwenzori, Mt Elgon).

***Isolepissetacea* R.Br.** – Habit: Herb. Habitat: Wet open grassland, 2400–3800 m. Vouchers: Haines 4151, Dummer RA 3494 (EA, US). Native distribution range: Temperate & Subtropical Eurasia, Africa.

***Scleriawoodii* C.B.Clarke** – Habit: Herb. Habitat: Wet grassland, 1000–2100 m. Vouchers: Lugard C Mrs 667B, 667A (EA). Native distribution range: Tropical & S Africa.

#### F23. DIOSCOREACEAE

1 Genus, 2 species

***Dioscoreaquartiniana* A.Rich.** – Habit: Climber. Habitat: Bushland, wooded grassland, 1750–2300 m. Vouchers: Tweedie 1404 (EA), Snowden JD 520, 910 (K), Lugard C 674 (K), Lye KA; Pócs T 23134 (K), Tweedie DR O 205 (BR). Native distribution range: Tropical & S Africa, Comoros, Madagascar.

***Dioscoreaschimperiana* Hochst. ex Kunth** – Habit: Twinning herb. Habitat: Forest margin, wooded grassland, riverine forest, 1600–2150 m. Vouchers: Tweedie EM 1839 (K), 2845 (K) 2847 (K), Irwin PH 185 (K), Jack C 274 (K), Coll S 453 (K). Native distribution range: Tropical Africa.

#### F24. ERIOCAULACEAE

1 Genus, 3 species

***Eriocaulonabyssinicum* Hochst.** – Habit: Herb. Habitat: Mist zone of waterfall, wet grassland, 1650–2750 m. Vouchers: Lind EM 247 (EA), Wood 446 (EA). Native distribution range: N. Nigeria to Eritrea and S Africa, Madagascar.

***Eriocaulonmesanthemoides* Ruhland** – Habit: Herb. Habitat: Upper edge of forest, montane grassland, moorland, 2400–3150 m. Voucher: Tweedie EM (1976) (REF). Native distribution range: Kenya to Malawi.

***Eriocaulonschimperi* Körn. ex Ruhland** – Habit: Herb. Habitat: Afroalpine grassland, bamboo zone, 2440–3500 m. Voucher: Hooper SS & Townsend CC 1392 (EA). Native distribution range: Ethiopia to Malawi.

#### F25. HYDROCHARITACEAE

3 Genera, 3 species

**Lagarosiphonhydrilloides Rendle – Habit: Aquatic herb. Habitat: Still or flowing fresh water, 1650–3500 m. Voucher: Lugard EJ; Lugard C 534 (K). Native distribution range: Kenya to Uganda.

^**EX**^***Najaspectinata* Magnus** – Habit: Herb. Habitat: Wet savanna, 1750–2250 m. Voucher: Agnew ADQ (2013) (REF). Native distribution range: Senegal to Sinai.

***Otteliaulvifolia* (Planch.) Walp.** – Habit: Herb. Habitat: Wetland, streams, 1750–2700 m. Voucher: Tweedie EM (1976) (REF). Native distribution range: Tropical & S Africa, Madagascar.

#### F26. HYPOXIDACEAE

1 Genus, 4 species

***Hypoxisangustifolia* Lam.** – Habit: Herb. Habitat: Wet savanna, 1300–3000 m. Voucher: Hedberg O (UPS). Native distribution range: Tropical & S Africa, W Indian Ocean, SW Arabian Peninsula.

***Hypoxisfischeri* Pax** – Habit: Herb. Habitat: Grassland, wooded grassland, 1500–2600 m. Voucher: Snowden JD 822 (EA). Native distribution range: Cameroon, S Sudan to S Tropical Africa.

***Hypoxisobtusa* Burch.** – Habit: Herb. Habitat: Wet savanna, swamp edges, 1750–2750 m. Voucher: Tweedie EM (1976) (REF). Native distribution range: Uganda to S Africa.

**^EX^*Hypoxisvillosa* L.f.** – Habit: Herb. Habitat: Burnt grassland, 1700–2300 m. Voucher: Agnew ADQ (2013) (REF). Native distribution range: S Africa.

#### F27. IRIDACEAE

8 Genera, 17 species

***Aristeaabyssinica* Pax ex Engl.** – Habit: Herb. Habitat: Wet savanna, woodland, upper edge of forest, grassland, 1400–3800 m. Voucher: Mwangangi OM 363 (EA). Native distribution range: Tropical & S Africa.

***Aristeaalata* Baker** – Habit: Herb. Habitat: Grass glades, forest edges, bamboo edge, 1800–3500 m. Voucher: Rono et al. SAJIT-PR 0125 (EA, HIB). Native distribution range: Ethiopia to E Tropical Africa.

***Aristeaangolensis* Baker** – Habit: Herb. Habitat: Wet grassland, 1700–2700 m. Vouchers: Tweedie EM (1976) (REF), Agnew ADQ (2013) (REF). Native distribution range: Tropical & S Africa.

***Dieramacupuliflorum* Klatt** – Habit: Herb. Habitat: Montane grassland, heath, 2000–3900 m. Vouchers: Adamson J 451 (K), Ludanga RI 337 (K), Dummer RA 3315 (EA), Tothill BH 2422 (EA), Rono et al. SAJIT-PR 0136 (EA, HIB). Native distribution range: Ethiopia to N Malawi.

**^EX^*Dieramapendulum* Baker** – Habit: Herb. Habitat: Moorland, 3150–4321 m. Voucher: Svein Manum (O). Native distribution range: South Africa.

***Dietesiridioides* (L.) Sweet ex Klatt** – Habit: Herb. Habitat: Thickets, riverine forests, 1500–2300 m. Voucher: Tweedie 165 (EA). Native distribution range: Ethiopia to S Africa.

***Freesialaxa* (Thunb.) Goldblatt & J.C.Manning** – Habit: Herb. Habitat: Rocky Mountain slopes, forest margin, 1800–2000 m. Voucher: Tweedie EM (1976) (REF). Native distribution range: W Kenya to S Africa.

***Gladiolusdalenii* Van Geel** – Habit: Herb. Habitat: Wet upland grassland, 1750–3100 m. Voucher: Tweedie EM (1976) (REF). Native distribution range: Tropical & S Africa, Madagascar, SW Arabian Peninsula.

***Gladiolusdichrous* (Bullock) Goldblatt** – Habit: Herb. Habitat: Outcrop rocks on cliffs, 2000–3500 m. Vouchers: Saunders & Hancock 34 (EA), Lankester CH sn (K). Native distribution range: South Sudan to W Kenya.

***Hesperanthapetitiana* Baker** – Habit: Herb. Habitat: Subalpine, alpine grassland, 1800–4000 m. Vouchers: Tweedie 42, 9, 121 (EA), Bickford N 53, 34 (EA), J. Wilson 1196 (EA). Native distribution range: Cameroon, Ethiopia to Zimbabwe.

*Moraeaafro-orientalis Goldblatt – Habit: Herb. Habitat: Wet grasslands, 1400–2400 m. Vouchers: Friis I; Hansen OJ 2568 (EA, K), Lugard EJ; Lugard C 573 (K), Adamson J 513 (K), Wesche K 1318 (K), Bridson DM 64 (K), Polhill 411 (K). Native distribution range: South Sudan to E Tropical Africa.

**^EX^*Moraeacarsonii* Baker** – Habit: Herb. Habitat: Open short grassland, outcrop rock, 1600–2400 m. Voucher: Polhill R 411 (K). Native distribution range: Sudan to W Botswana.

***Moraeastricta* Baker** – Habit: Herb. Habitat: Rocky grasslands, 1600–3000 m. Voucher: Rono et al. SAJIT-PR 0021 (EA, HIB). Native distribution range: Ethiopia to S Africa.

**^EX^*Moraeavegeta* L.** – Habit: Herb. Habitat: Wet savanna, woodland, 1750–2400 m. Voucher: Tweedie EM (1976) (REF). Native distribution range: South Africa.

***Romuleacamerooniana* Baker** – Habit: Herb. Habitat: Woodland, upper edge forest, wet grassland and subalpine stony soils, 1750–3150 m. Vouchers: Tweedie EM (1976) (REF), Agnew ADQ (2013) (REF). Native distribution range: Cameroon, S Ethiopia to S Africa.

***Romuleacongoensis* Bég.** – Habit: Herb. Habitat: Alpine grassland, mossy seeps, 3300–4200 m. Vouchers: Hamilton & Perrott 30 (EA). Native distribution range: Ethiopia to Rwanda.

***Romuleafischeri* Pax** – Habit: Herb. Habitat: Wet upland and subalpine stony grassland, 2700–4200 m. Vouchers: Tweedie EM 2051 (K), Snowden JD 470 (EA), Rono et al. SAJIT-PR 0108, SAJIT-PR 0126 (EA, HIB). Native distribution range: NE Tropical Africa to Uganda, Socotra, SW Arabian Peninsula.

#### F28. JUNCACEAE

2 Genera, 6 species

***Juncuscapitatus* Weigel** – Habit: Herb. Habitat: Ephemeral wetland, 4000 m. Voucher: Agnew ADQ (2013) (REF). Native distribution range: Europe to Kazakhstan, Macaronesia, Mediterranean to Kenya, Cameroon, S Africa.

***Juncusdregeanus* Kunth** – Habit: Herb. Habitat: Alpine forest, streamside, moor grassland, bog, 2900–3400 m. Vouchers: Lye KA, Katende A & Swinscow D (UPS). Native distribution range: Cameroon to Sudan and S Africa.

***Juncuseffusus* L.** – Habit: Herb. Habitat: Moorland marshes, 1500–3300 m. Voucher: Hedberg 180 (EA). Native distribution range: Temperate Northern Hemisphere to W South America, Rwanda to S Africa, W Indian Ocean.

***Luzulaabyssinica* Parl.** – Habit: Herb. Habitat: Wet alpine forest, streamside, seepages, 2000–4300 m. Vouchers: Bogdan 4139, Ekkens DB 623 (EA), Mwangangi OM 350 (EA), Svein Manum 44 (O). Native distribution range: Ethiopia to E & E Central Tropical Africa.

***Luzulajohnstonii* Buchenau** – Habit: Herb. Habitat: Alpine wet grasslands, 2400–4200 m. Vouchers: Hedberg 213 (EA), Dummer RA 3548 (EA), Bogdan A 4128 (EA), Hedberg O 975 (EA). Native distribution range: Ethiopia to E Central & E Tropical Africa.

**Luzulamanniisubsp.gracilis (S.Carter) Kirschner & Cheek – Habit: Herb. Habitat: Upland grassland, wet places, by streams, moorland, 2700–3700 m. Vouchers: Hedberg 186 (EA), Dummer RA 3545 (EA), Liebenberg 1704 (EA), Wood GHS 126 (EA). Native distribution range: E Tropical Africa (Mt Elgon).

#### F29. ORCHIDACEAE

31 Genera, 114 species

***Aerangisluteoalba* Schltr.** – Habit: Epiphytic herb. Habitat: Moist shady forest, woodland, 1500–2330 m. Voucher: Gerh. Lindblom (S). Native distribution range: W Central & E Tropical Africa to Ethiopia.

***Aerangisthomsonii* Schltr.** – Habit: Epiphytic herb. Habitat: Upland Forest, woodland, forest, 1750–3000 m. Vouchers: Snowden JD 809 (EA), Major C & Lugard EJ 468 (EA), (K), Tweedie 30 (K). Native distribution range: SW Ethiopia to E Tropical Africa.

***Aerangisugandensis* Summerh.** – Habit: Epiphytic herb. Habitat: Forest trees, 1330–2300 m. Vouchers: Snowden JD 879 (K, NHMUK). Native distribution range: E DR Congo to Kenya.

***Angraecopsisgracillima* (Rolfe) Summerh.** – Habit: Epiphytic herb. Habitat: Woodland, evergreen forest, 1750–2250 m. Vouchers: Snowden JD 505 (EA), Snowden JD 202 (EA). Native distribution range: Uganda to Zambia.

**^EX^*Angraecopsistenerrima* Kraenzl.** – Habit: Epiphytic herb. Habitat: Woodland, 1750–3000 m. Voucher: Tweedie EM (1976) (REF). Native distribution range: Tanzania.

***Angraecumerectum* Summerh.** – Habit: sub-epiphytic herb. Habitat: Riverine Forest, 1350–2350 m. Voucher: Agnew ADQ (2013) (REF). Native distribution range: E Tropical Africa to Zambia.

***Angraecumsacciferum* Lindl.** – Habit: Epiphytic herb. Habitat: Shady Forest, 1650–2650 m. Vouchers: Irwin PH 178 (EA), Tweedie EM 463 (K), Rono et al. SAJIT-PR 0010 (EA, HIB). Native distribution range: São Tomé to Kenya and S Africa.

***Anselliaafricana* Lindl.** – Habit: Herb. Habitat: Woodland, wooded grassland on rocks, tree roots, 1750–2350 m. Vouchers: Tweedie EM 12962 (EA), Snowden JD 942 (EA), Tweedie EM 553 (BR), Laan FM van der 1031 (WAG). Native distribution range: Tropical & S Africa.

**^EX^*Arachnisflos-aeris* (L.) Rchb.f.** – Habit: Herb. Habitat: Moist forest, 1000 m. Voucher: Adamson J (6) (EA). Native distribution range: S Indo-China to W Malesia and Phillipines.

***Bonateasteudneri* T.Durand & Schinz** – Habit: Herb. Habitat: Moist forest on rocks, Bushland, 1700–2700 m. Vouchers: Tweedie EM (1976) (REF), Agnew ADQ (2013) (REF). Native distribution range: NE Sudan to South Africa, SW Arabian Peninsula.

***Brachycorythiskalbreyeri* Rchb.f.** – Habit: Herb. Habitat: Riverine Forest, upland rain forest, 1800–2350 m. Voucher: Bush RZ 244 (EA). Native distribution range: W Tropical Africa to Kenya and Zambia.

***Brachycorythisovata* Lindl.** – Habit: Herb. Habitat: Grassland, 1200–2550 m. Vouchers: Snowden JD 877 (EA, K). Native distribution range: Tropical & S Africa.

**Brachycorythisovatasubsp.schweinfurthii (Rchb.f.) Summerh.** – Habit: Herb. Habitat: Wet savanna, 1750–2250 m. Voucher: Tweedie 8 (EA). Native distribution range: W Tropical Africa to Ethiopia and Tanzania.

***Brachycorythispleistophylla* Rchb.f.** – Habit: Herb. Habitat: Grassland and edges of deciduous woodland, 2000–2700 m. Voucher: Chater-Jack 847 (EA). Native distribution range: Tropical & S Africa, Madagascar.

***Brachycorythispubescens* Harv.** – Habit: Herb. Habitat: Upland grassland, woodland, 2000–2700 m. Vouchers: Tweedie EM 14 (K), Snowden JD 928 (AMES). Native distribution range: Tropical & S Africa.

***Bulbophyllumcochleatum* Lindl.** – Habit: Epiphytic herb. Habitat: Shady Forest, 1450–2400 m. Vouchers: Snowden JD 940 (EA), Tweedie 47 (EA). Native distribution range: Tropical Africa.

***Bulbophyllumintertextum* Lindl.** – Habit: Herb. Habitat: Moist and riverine forest, 1750–2400 m. Voucher: Tweedie EM (1976) (REF). Native distribution range: Tropical Africa, Madagascar, Seychelles.

**^EX^Bulbophyllumjosephivar.mahonii (Rolfe) J.J.Verm.** – Habit: Epiphytic herb. Habitat: Montane forest, 850–2100 m. Voucher: Snowden JD 923 (EA). Native distribution range: W Tropical Africa to Malawi.

***Cynorkisanacamptoides* Kraenzl.** – Habit: herb. Habitat: Upland moorland, grassland, upland rain forest, 2250–3350 m. Vouchers: Irwin PH 247 (EA), Tweedie 69/58 (EA), Tweedie EM 1191 (K). Native distribution range: Tropical Africa.

***Cyrtorchisarcuata* Schltr.** – Habit: Epiphytic herb. Habitat: Woodland, wet savanna, open forest, 1750–3300 m. Vouchers: Symes YE 357 (EA), Chater-Jack 225 (EA), Major C & Lugard EJ 565 (EA), Rono et al. SAJIT-PR 0005 (EA, HIB). Native distribution range: Tropical & S Africa.

***Diaphananthelorifolia* Summerh.** – Habit: Epiphytic herb. Habitat: Forest, 1830–2330 m. Vouchers: Snowden JD sn (NMHUK). Native distribution range: Ethiopia to Rwanda and Tanzania.

***Diaphanantheodoratissima* (Rchb.f.) P.J.Cribb & Carlsward** – Habit: Epiphytic herb. Habitat: Forest trees, 1500–2430 m. Vouchers: Tweedie 31 (EA), Chater-Jack 36 (EA), Laan, FM van der 1066 (WAG). Native distribution range: Tropical Africa.

***Diaphananthesarcophylla* (Schltr. ex Prain) P.J.Cribb & Carlsward** – Habit: Epiphytic herb. Habitat: Montane Forest, 1500–2400 m. Vouchers: Snowden JD 880 (EA), Tweedie 559 (EA), Tweedie EM 482 (K). Native distribution range: Central & E Tropical Africa.

***Diaphananthevesicata* (Lindl.) P.J.Cribb & Carlsward** – Habit: Epiphytic herb. Habitat: Montane moist forest, 350–2200 m. Voucher: Tweedie EM 2481 (K). Native distribution range: W Tropical Africa to Tanzania.

***Disaaconitoides* Sond.** – Habit: Herb. Habitat: Grassland, 2300–2500 m. Vouchers: Tweedie EM 12954 (EA), Chater-Jack 244 (EA), Tweedie 66/147 (EA), Major C & Lugard EJ 594 (EA). Native distribution range: Ethiopia to S Africa.

**Disaaconitoidessubsp.concinna (N.E.Br.) H.P.Linder** – Habit: Herb. Habitat: wet savanna, woodland, 1450–2850 m. Vouchers: Chater-Jack 244 (EA), Snowden JD 1086 (EA). Native distribution range: Ethiopia to S Tropical Africa.

***Disaerubescens* Rendle** – Habit: Herb. Habitat: Upland grasslands, 1500–2850 m. Vouchers: Tweedie 66/148 (EA), Dale IR 3135 (AMES). Native distribution range: Tropical Africa.

***Disahircicornis* Rchb.f.** – Habit: Herb. Habitat: Wet savanna, 1750–2250 m. Voucher: Tweedie EM 572 (K). Native distribution range: Tropical & S Africa.

***Disascutellifera* A.Rich.** – Habit: Herb. Habitat: Damp grassland, grassy rocky slopes, 1800–2700 m. Voucher: Tweedie EM 12953 (EA). Native distribution range: NE & E Tropical Africa.

***Disastairsii* Kraenzl.** – Habit: Herb. Habitat: Alpine grassland, moorland, rocky areas 3000–4200 m. Vouchers: Tweedie DR 5446 (EA), 3 (EA), Adamson J 464 (EA), Tweedie EM 1807A (K). Native distribution range: Congo to E Tropical Africa.

***Disawelwitschii* Rchb.f.** – Habit: Herb. Habitat: Damp grasslands, 1700–2000 m. Vouchers: Lindblom, S s.n. (K). Native distribution range: Tropical & S Africa.

***Disperisanthoceros* Rchb.f.** – Habit: Herb. Habitat: Evergreen forest, bamboo forests on ground litter, 1800–3000 m. Vouchers: Tweedie 560 (EA), Tweedie EM 2852(K). Native distribution range: Tropical & S Africa, Madagascar.

***Disperisdicerochila* Summerh.** – Habit: Herb. Habitat: Upland moist forest in leaf litter, mossy branches, rocks, 1650–2830 m. Voucher: Sellow, Miss (K). Native distribution range: Ethiopia to S Tropical Africa.

***Disperiskilimanjarica* Rendle** – Habit: Herb. Habitat: Dense shade forest on branches covered with moss and liverworts, 2300–3000 m. Vouchers: Tweedie 920 (EA, K). Native distribution range: Kenya to Zambia.

***Disperisreichenbachiana* Welw. ex Rchb.f.** – Habit: Herb. Habitat: Grassland, forest floor on leaf mould, rock crevices, 1500–2330 m. Vouchers: Tweedie DR 9 (EA), Rono et al. SAJIT-PR 0012 (EA, HIB). Native distribution range: Tropical Africa.

**^EX^*Epidendrumibaguense* Kunth** – Habit: Herb. Habitat: Forest, grassland, 1300–1750 m. Voucher: Webster MVB 9025 (EA). Native distribution range: Colombia to Trinidad and W Bolivia.

***Epipactisafricana* Rendle** – Habit: Herb. Habitat: Riverbanks in forest, bamboo forest, 2330–3750 m. Vouchers: Irwin PH 149 (EA), Napper DM & Irwin D 1495 (EA), Mwangangi OM 426 (K), Tweedie EM 1091 (K, BR). Native distribution range: SW Ethiopia to N Mozambique.

***Epipogiumroseum* Lindl.** – Habit: Herb. Habitat: Leaf mold in wet forest floor, 1300–2250 m. Vouchers: Tweedie (Icon). Native distribution range: Tropical Africa to SW Pacific.

***Eulophiacucullata* (Afzel. ex Sw.) Steud.** – Habit: Herb. Habitat: Grassland, 1750–2300 m. Vouchers: Webster MVB 9037 (EA), Chater-Jack 231 (EA), Tweedie 67/57 (EA), Major C & Lugard EJ 596 (EA), Tweedie EM 5 (K). Native distribution range: Tropical & S Africa, Comoros, Madagascar.

***Eulophiaeustachya* (Rchb.f.) Geerinck** – Habit: Herb. Habitat: Wet grassland, 1650–2000 m. Voucher: Agnew ADQ (2013) (REF). Native distribution range: Ethiopia to Zimbabwe.

***Eulophiaguineensis* Lindl.** – Habit: Herb. Habitat: Outcrop rock, woodland, 1800–2400 m. Vouchers: Tweedie 2586, 67/42 (EA), Tweedie DR 1 (EA), Chater-Jack 226 (EA). Native distribution range: Cape Verde, Tropical Africa to Botswana, Arabian Peninsula.

***Eulophiahorsfallii* (Bateman) Summerh.** – Habit: Herb. Habitat: Open Forest, riverine, outcrop rock, 1500–2700 m. Vouchers: Snowden JD 816 (EA), Symes YE 629 (EA). Native distribution range: Tropical & S Africa.

***Eulophialatilabris* Summerh.** – Habit: Herb. Habitat: Seasonally wet grassland, 1300–1600 m. Voucher: Snowden JD 876 (EA). Native distribution range: Nigeria to Sudan and Botswana.

***Eulophialivingstoneana* (Rchb.f.) Summerh.** – Habit: Herb. Habitat: Grassland, 1500–2350 m. Vouchers: Chater-Jack 230 (EA), 3314 (EA). Native distribution range: Ethiopia to South Africa, Comoros, Madagascar.

***Eulophiamechowii* T.Durand & Schinz** – Habit: Herb. Habitat: Grassland, wooded grassland, 2000–2350 m. Voucher: Lugard 634 (EA). Native distribution range: Nigeria to W Ethiopia and S Africa.

***Eulophiamontis-elgonis* Summerh.** – Habit: Herb. Habitat: Wet grassland among rocks, 2000–2350 m. Vouchers: Dale IR 3109 (EA), Irwin PH 285 (EA), Tweedie 91 (EA), Chater-Jack 275 (EA), Lugard EJ 663 (EA) (K). Native distribution range: S Sudan to Tanzania.

***Eulophiaodontoglossa* Rchb.f.** – Habit: Herb. Habitat: Grassland, bushland, rocky areas, 1750–2350 m. Vouchers: Tweedie 620 (EA). Native distribution range: Tropical & S Africa.

***Eulophiaorthoplectra* (Rchb.f.) Summerh.** – Habit: Herb. Habitat: Grassland, wooded grassland, swamp woodland, 1200–2350 m. Vouchers: Lugard 11 (EA), Gardner HM 2277 (EA), Chater-Jack 96 (EA). Native distribution range: Tropical Africa.

***Eulophiaruwenzoriensis* Rendle** – Habit: Herb. Habitat: Grassland, 1650–2530 m. Voucher: Agnew ADQ (2013) (REF). Native distribution range: E Tropical Africa to Zambia, E Bolivia to Brazil and NE Argentina.

***Eulophiastreptopetala* Lindl.** – Habit: Herb. Habitat: Forest, bush, grassland, 1750–2250 m. Vouchers: Chater-Jack 87 (EA), 159 (EA), Tweedie DR 4 (EA), Tweedie 66/149 (EA). Native distribution range: Eritrea to S Africa, SW Arabian Peninsula.

***Eulophiasubulata* Rendle** – Habit: Herb. Habitat: Swamps, seasonally wet grassland, 1100–1700 m. Voucher: Human observation sn (EA). Native distribution range: E Tropical Africa to Angola.

***Habenariaaltior* Rendle** – Habit: Herb. Habitat: Grassland, streams, forest, 2000–3700 m. Vouchers: Irwin PH 425 (EA), 429 (EA), Tweedie EM 727 (K), Tweedie 1266 (K). Native distribution range: E Tropical Africa.

***Habenariaattenuata* Hook.f.** – Habit: Herb. Habitat: Moist upland forest, moorland, 2400–4000 m. Voucher: Tweedie 360 (EA). Native distribution range: Ethiopia to Cameroon and Tanzania SW Arabian Peninsula.

***Habenariabracteosa* Hochst. ex A.Rich.** – Habit: Herb. Habitat: Mountain forest, 2500–4000 m. Voucher: Tweedie EM 537 (K). Native distribution range: Bioko to Ethiopia and Tanzania.

***Habenariachirensis* Rchb.f.** – Habit: Herb. Habitat: Wet grassland, swamps, wet rocks outcrop, 1850–2300 m. Vouchers: Chater-Jack 302 (EA), Symes YE 395 (EA), Tweedie 66/145 (EA). Native distribution range: Nigeria to Ethiopia and Tanzania.

***Habenariacornuta* Lindl.** – Habit: Herb. Habitat: Grassland, 1700–2700 m. Voucher: Tweedie EM 523 (K). Native distribution range: Tropical & S Africa.

***Habenariadecorata* Hochst. ex A.Rich.** – Habit: Herb. Habitat: Rock outcrop, upland moor, 2200–3300 m. Voucher: Thomas AS 642 (EA). Native distribution range: Ethiopia to Uganda.

***Habenariafilicornis* Lindl.** – Habit: Herb. Habitat: Damp areas, grassland, woodland, 1200–2850 m. Vouchers: Tweedie 395(N19) (EA), Tweedie EM 321 (EA). Native distribution range: Tropical & S Africa.

***Habenariahologlossa* Summerh.** – Habit: Herb. Habitat: Damp grassland, open bushland, 1750– 3300 m. Vouchers: Tweedie 469 (EA), Tweedie DR 201 (EA), 209 (EA), Tweedie EM 323 (EA), 324 (EA), 325 (EA). Native distribution range: Kenya, Malawi, Angola.

***Habenariaholubii* Rolfe** – Habit: Herb. Habitat: Wet grassland, streamside, 1200–2300 m. Voucher: Chater-Jack 301 A (EA). Native distribution range: Tropical Africa to Namibia.

***Habenariahuillensis* Rchb.f.** – Habit: Herb. Habitat: Grassland, 1200–2500 m. Vouchers: Irwin PH 224 (EA), Tweedie 67/228 (EA), Tweedie DR 10 (EA), Symes YE 164 (EA), Tweedie 2671 (EA). Native distribution range: Tropical Africa.

***Habenariahumilior* Rchb.f.** – Habit: Herb. Habitat: Grassland, 1200–2700 m. Vouchers: Symes 164 (EA), Rono et al. SAJIT-PR 0114 (EA, HIB). Native distribution range: Ethiopia to S Africa.

*Habenariakeniensis Summerh. – Habit: Herb. Habitat: Woodland, upland rainforest edges, 1950–2950 m. Vouchers: Tweedie in Bally 7619 (EA), Irwin PH 448 (EA), Tweedie 703 (EA), 387 (K), Tweedie EM 505a (K). Native distribution range: Kenya.

***Habenarialaurentii* De Wild.** – Habit: Herb. Habitat: Grassland, bushland, 1800–2100 m. Vouchers: Chater-Jack 310 (EA), 301B, Tweedie EM 630 (K). Native distribution range: Tropical Africa.

***Habenarialindblomii* Schltr.** – Habit: Herb. Habitat: Grassland, 1800–2200 m. Vouchers: Tweedie 522 ,66 (K), Tweedie 2667 (EA), Tweedie EM 12951 (EA). Native distribution range: Kenya to S Tropical Africa.

***Habenariamacruroides* Summerh.** – Habit: Herb. Habitat: Grassland, swamps 1700–2000 m. Voucher: Tweedie 32 (EA). Native distribution range: W Central & E Tropical Africa.

***Habenariamalacophylla* Rchb.f.** – Habit: Herb. Habitat: Upland moist forest, grassland, forest, 1200–2700 m. Vouchers: Irwin PH 63 (EA), Tweedie 17 (EA). Native distribution range: Tropical & S Africa, S Arabian Peninsula.

***Habenariamicrosaccus* Kraenzl.** – Habit: Herb. Habitat: Upland grassland, forest, 2100–3000 m. Vouchers: Tweedie EM 728 (K), 1725 (K). Native distribution range: Uganda to Burundi.

***Habenariandiana* Rendle** – Habit: Herb. Habitat: Forest, grassland, 1700–3000 m. Vouchers: Tweedie 404 (EA), Tweedie EM 471 (K). Native distribution range: E Tropical Africa to Malawi.

***Habenariaperistyloides* A.Rich.** – Habit: Herb. Habitat: Upland grassland, marshes, 2000–2850 m. Vouchers: Tweedie DR 11 (EA), Tweedie 67/189 (EA). Native distribution range: Nigeria to SW Arabian Peninsula.

***Habenariapetitiana* T.Durand & Schinz** – Habit: Herb. Habitat: Grassland, forest edges, 1700–3300 m. Vouchers: Lucas GLl & Williams JG 177 (EA), G. Ll. Lucas 177 (AMES). Native distribution range: Cameroon to Eritrea and S Africa.

***Habenariaquartiniana* A.Rich.** – Habit: Herb. Habitat: Upland grassland, thick bush, forest edge, 2100–2850 m. Vouchers: Tweedie EM 702 (K), Rono et al. SAJIT-PR 0133 (EA, HIB). Native distribution range: Eritrea to Uganda.

***Habenariasplendens* Rendle** – Habit: Herb. Habitat: Upland grassland, forest margin, 1700–2700 m. Vouchers: Tweedie (EA), Symes YE 628 (EA), Chater-Jack 300 (EA), Snowden JD 913 (AMES). Native distribution range: Ethiopia to Zambia.

***Habenariatweedieae* Summerh.** – Habit: Herb. Habitat: Rock outcrop, woodland, 2200–2600 m. Vouchers: Irwin PH 524 (EA), Tweedie DR 55 (EA), 25 (EA), Tweedie EM 680 (K). Native distribution range: SW Ethiopia to Rwanda.

***Habenariavaginata* A.Rich.** – Habit: Herb. Habitat: Damp grassland, forest edges, 1300–3000 m. Vouchers: Tweedie EM 12949 (EA), Tweedie 2672 (EA), Tweedie EM 507 (K). Native distribution range: Eritrea to Tanzania.

***Habenariawalleri* Rchb.f.** – Habit: Herb. Habitat: Swampy grassland, 1200–2300 m. Vouchers: Tweedie 12946 (EA), 66/121 (EA). Native distribution range: Tropical Africa.

***Holothrixaphylla* Rchb.f.** – Habit: Herb. Habitat: Upland grassland, forest, 1500–2600 m. Vouchers: Chater-Jack 253, 252 (EA). Native distribution range: Nigeria to Yemen.

**Holothrixelgonensis Summerh. – Habit: Herb. Habitat: Upland grassland, forest, 3300–3900 m. Vouchers: Lugard 379 (EA), Tweedie 199 (EA), Thomas 2682 (EA), Tweedie DR 31, 109 (EA), Tweedie EM 359 (EA), Lugard EJ 379 (EA), Lugard EJ; Lugard C 379 (K), Lugard EJ; Lugard C 379a (K). Native distribution range: E Tropical Africa (Mt Elgon).

***Kylicantherohrii* (Rchb.f.) Descourv. & Farminhão** – Habit: Epiphytic herb. Habitat: Montane forest, 2100–3000 m. Vouchers: C Leaky 24 (EA), Tweedie 2015 (EA, K), Snowden JD 921 (NHMUK). Native distribution range: Tropical Africa.

***Liparisbowkeri* Harv.** – Habit: Herb. Habitat: Mossy banks, forest trees, 1350–2700 m. Voucher: Agnew ADQ (2013) (REF). Native distribution range: Ethiopia to S Africa.

***Liparisdeistelii* Schltr.** – Habit: Herb. Habitat: Forest on mossy banks on tree and tree fern trunks, 1700–2750 m. Vouchers: Tweedie 506 (EA), Tweedie EM 457 (K), Synge PM 980 (EA, BR). Native distribution range: W Central & E Tropical Africa to W Ethiopia and Malawi.

^**EX**^***Platantheramicrantha* Schltr.** – Habit: Herb. Habitat: Upland grassland, upland moor, 2500–3000 m. Voucher: Lankester 13 (EA). Native distribution range: Azores.

***Platycorynecrocea* Rolfe** – Habit: Herb. Habitat: Grassland, 1300–2600 m. Vouchers: Dale IR 3137 (EA), Chater-Jack 255 (EA), Tweedie EM 12963 (EA), Symes YE 355 (EA), Major C & Lugard EJ 647 (EA), Tweedie 66/102 (EA). Native distribution range: Cameroon to Ethiopia and Zimbabwe.

***Polystachyaadansoniae* Rchb.f.** – Habit: Epiphytic herb. Habitat: Forest, woodland, 1650–2330 m. Vouchers: Tweedie 172 (EA), Chater-Jack 4818 (EA), Tweedie EM 12945 (EA). Native distribution range: Tropical Africa.

***Polystachyabella* Summerh.** – Habit: Epiphytic herb. Habitat: Forest trees, 1800–2350 m. Vouchers: Bush RZ 245 (EA). Native distribution range: SW Kenya.

***Polystachyabennettiana* Rchb.f.** – Habit: Epiphytic herb. Habitat: wet savanna, woodland, dry forest, 1750–2400 m. Vouchers: Yohana 2523 (EA), Chater-Jack 215 (EA). Native distribution range: Ivory Coast to Eritrea and Zambia.

***Polystachyabicarinata* Rendle** – Habit: Epiphytic herb. Habitat: Upland riverine forest, 1800–2830 m. Vouchers: Snowden JD 960 (EA, MO), Tisdall 313 (MO), Rono et al. SAJIT-PR 0034 (EA, HIB). Native distribution range: E Central & E Tropical Africa.

***Polystachyacaespitifica* Kraenzl. ex Engl.** – Habit: Epiphytic herb. Habitat: Forest, 1800–2700 m. Voucher: Agnew ADQ (2013) (REF). Native distribution range: Kenya to Zimbabwe.

***Polystachyacampyloglossa* Rolfe** – Habit: Epiphytic herb. Habitat: Dry Forest, 1700–3000 m. Voucher: Tweedie EM 1085 (K). Native distribution range: E Tropical Africa to E Zimbabwe.

***Polystachyacultriformis* Lindl. ex Spreng.** – Habit: Epiphytic herb. Habitat: Forest near rivers, 330–2700 m. Vouchers: Tweedie DR 7 (EA), Bamps PRJ sn (EA), Tweedie EM 556 (K). Native distribution range: Bioko to Ethiopia and S Africa, W Indian Ocean.

***Polystachyaeurychila* Summerh.** – Habit: Epiphytic herb. Habitat: Dry forest, 1830–2300 m. Vouchers: Tweedie EM 555 (K), Eggeling WJ 2438 (K), Eggeling WJ 2438 (K, US), Meyer, H. 291 (K), Dummer RA 3679 (K). Native distribution range: Ethiopia to NW Kenya.

***Polystachyalindblomii* Schltr.** – Habit: Epiphytic herb. Habitat: Forest, 1000–2330 m. Vouchers: Tweedie EM 470 (K), Rono et al. SAJIT-PR 0009 (EA, HIB). Native distribution range: SW Ethiopia to S Tropical Africa.

***Polystachyasimplex* Rendle** – Habit: Epiphytic herb. Habitat: Dry montane forest, 2000–2350 m. Vouchers: Snowden JD 875 (EA), Tweedie DR 81 (EA). Native distribution range: SW Ethiopia to E Zimbabwe.

***Polystachyaspatella* Kraenzl.** – Habit: Epiphytic herb. Habitat: Forest, 1600–2830 m. Vouchers: Tweedie 315, Major C & Lugard EJ 640 (EA), Bamps PRJ sn (EA), Tweedie EM 447 (K). Native distribution range: E Central & E Tropical Africa.

***Polystachyasteudneri* Rchb.f.** – Habit: Epiphytic herb. Habitat: Dry forest, 1850–2500 m. Vouchers: Lugard 504 (EA), Snowden JD 959 (EA), Jack C 401 (EA). Native distribution range: Nigeria to Eritrea and Burundi.

***Polystachyatenuissima* Kraenzl.** – Habit: Epiphytic herb. Habitat: Upland forest, 1700–2500 m. Vouchers: Bush RZ 246 (EA), Hedberg O 303 (EA), Tweedie 2634 (EA), Tweedie DR 6 (EA), Tweedie EM 448 (K). Native distribution range: W Tropical Africa to Uganda and N Tanzania.

***Polystachyatransvaalensis* Schltr.** – Habit: Epiphytic herb. Habitat: Upland forest, 1650–3350 m. Vouchers: Tweedie DR 20 (EA), sn (EA). Native distribution range: South Sudan and S Africa.

***Porpaxrepens* (Rolfe) Schuit., Y.P.Ng & H.A.Pedersen** – Habit: Herb. Habitat: Riverine forest, 1950–2830 m. Vouchers: Tweedie EM 1066 (K). Native distribution range: Tropical Africa.

***Rangaerismuscicola* (Rchb.f.) Summerh.** – Habit: Epiphytic herb. Habitat: Evergreen forest, woodland, 1700–2350 m. Vouchers: Snowden JD 25 (EA), Major C & Lugard EJ 645 (EA), Chater-Jack 222 (EA). Native distribution range: Tropical & S Africa.

***Rhipidoglossumbrachyceras* (Summerh.) Farminhão & Stévart** – Habit: Epiphytic herb. Habitat: Forest, 1700–2000 m. Vouchers: Dummer RA 3686 (EA), Snowden JD 900 (EA, NMHUK). Native distribution range: Tropical Africa.

***Rhipidoglossumpulchellum* (Summerh.) Garay** – Habit: Epiphytic herb. Habitat: Dry forest, 1500–2330 m. Vouchers: Tweedie EM 571, 570A (K). Native distribution range: Central & E Tropical Africa.

***Rhipidoglossumsubsimplex* (Summerh.) Garay** – Habit: Epiphytic herb. Habitat: Forest, 1850–2850 m. Voucher: Rono et al. SAJIT-PR 0063 (EA, HIB). Native distribution range: E & S Tropical Africa.

***Satyriumcarsonii* Rolfe** – Habit: Herb. Habitat: Open grasslands, woodland, 1450–2330 m. Vouchers: Tweedie 161 (EA), Tweedie DR 8 (EA), Webster MVB 9031 (EA). Native distribution range: Nigeria to Kenya and Zambia.

***Satyriumcoriophoroides* A.Rich.** – Habit: Herb. Habitat: Grassland, rocky slopes, 1700–2800 m. Vouchers: Tweedie EM (1976) (REF), Agnew ADQ (2013) (REF). Native distribution range: Cameroon to Ethiopia and S Tropical Africa.

***Satyriumcrassicaule* Rendle** – Habit: Herb. Habitat: Wet grassland, 1850–3300 m. Vouchers: Snowden JD 922 (EA), Major C & Lugard EJ 424 (EA), Synge PM 820 (WAG), Rono et al. SAJIT-PR 0258 (EA, HIB). Native distribution range: Nigeria to Ethiopia and Zambia.

**^EX^Satyriumneglectumsubsp.woodii (Schltr.) A.V.Hall** – Habit: Herb. Habitat: Upland grassland, 2150–3000 m. Voucher: Agnew ADQ (2013) (REF). Native distribution range: South Africa.

***Satyriumrobustum* Schltr.** – Habit: Herb. Habitat: Swampy grasslands, bog near streams, 1350–3000 m. Vouchers: Tweedie EM 810 (EA, K, UPS), Bickford N 14 (EA), Tweedie EM 837 (EA), Hedberg O 171 (EA). Native distribution range: E Tropical Africa.

***Satyriumsceptrum* Schltr.** – Habit: Herb. Habitat: upland grassland, and open bushland, upper forest edge in damp places, 1500–2700 m. Voucher: Tylor in Tweedie 624 (2) (EA). Native distribution range: South Sudan to S Tropical Africa.

***Satyriumvolkensii* Schltr.** – Habit: Herb. Habitat: Upland grassland, open woodland, bushland, 2200–2700 m. Vouchers: Chater-Jack 245 in CM 4816 (EA), 246 (EA), Tweedie EM 12957 (EA), Major C & Lugard EJ 595 (EA). Native distribution range: Nigeria to Kenya and Zimbabwe.

***Sphyrarhynchusamaniense* (Summerh.) Bytebier** – Habit: Epiphytic herb. Habitat: Shady montane forest, 1600–2300 m. Vouchers: Tweedie 603 (EA), Carroll, EW (K). Native distribution range: Kenya to S Tropical Africa.

***Tridactylebicaudata* Schltr.** – Habit: Epiphytic herb. Habitat: Dry forests, riverine forest, 0–2500 m. Vouchers: Blancowe J sn (EA), Chater-Jack 104 (EA). Native distribution range: Tropical & S Africa.

***Tridactylescottellii* Schltr.** – Habit: Epiphytic herb. Habitat: Evergreen riverine forest, 2000–3000 m. Vouchers: Snowden JD 903 (EA), Tweedie 88 (EA), Rono et al. SAJIT-PR 0106 (EA, HIB). Native distribution range: Gabon to Kenya.

***Tridactyletridactylites* Schltr.** – Habit: Epiphytic herb. Habitat: Montane rain forest, 1000–1800 m. Voucher: Snowden JD 525 (EA). Native distribution range: Tropical Africa.

***Ypsilopusamaniensis* (Kraenzl.) D’haijère & Stévart** – Habit: Epiphytic herb. Habitat: Dry forest, open woodland 1500–2350 m. Voucher: Agnew ADQ (2013) (REF). Native distribution range: S Ethiopia to Zimbabwe.

#### F30. POACEAE

50 Genera, 98 species

***Adenochloahymeniochila* (Nees) Zuloaga** – Habit: Herb. Habitat: River margins, moist meadows, 1000–3000 m. Vouchers: Snowden JD 118 (US, MO, K). Native distribution range: Tropical & S Africa, Madagascar.

***Agrostisgracilifolia* C.E.Hubb.** – Habit: Herb. Habitat: Moorland, highland grasses, 2800–4300 m. Vouchers: Hedberg 192 (EA), Bogdan 3934 (EA), Thomas AS 2671 (EA), Dummer RA (B) 3339, 3475 (US), Liebenberg LCC 1689 (K), Johnston HB 876 (K), Taylor Dr G 3709 (K). Native distribution range: Ethiopia to Tanzania.

***Agrostiskeniensis* Pilg.** – Habit: Herb. Habitat: Upland forest edges, wet upland grassland, 2300–3000 m. Voucher: Tothill BH 2262 (EA). Native distribution range: Ethiopia to E Tropical Africa.

***Agrostiskilimandscharica* Mez** – Habit: Herb. Habitat: Bamboo, forest clearing, upland grassland, 2100–3700 m. Vouchers: Bodgan A 3930 (EA), Thomas AS 2718 (EA), Taylor G 3703 (K), Johnston HB 876 (K), Liebenberg LCC 1689 (K), Hedberg KO 4461 (BR), Dummer RA 3569 (US). Native distribution range: Congo to Ethiopia and Tanzania.

***Agrostisproducta* Pilg.** – Habit: Herb. Habitat: Forest edges, wet upland grassland, 2400–3800 m. Voucher: Thomas AS 2685, 2712 (EA). Native distribution range: South Sudan to Zimbabwe.

***Agrostisquinqueseta* (Steud.) Hochst.** – Habit: Herb. Habitat: Moorland, alpine heath, 3000–4000 m. Vouchers: Knight J 4501 (K), Ekkens DB 630 (EA), Bogdan A 3931 (EA). Native distribution range: Cameroon to Ethiopia and Tanzania.

***Agrostisvolkensii* Stapf** – Habit: Herb. Habitat: Forest edges, wet upland grassland, 3000–4300 m. Voucher: Dummer RA 3339A (EA). Native distribution range: Ethiopia to E Tropical Africa.

***Airacaryophyllea* L.** – Habit: Herb. Habitat: Grassland, moorland, 2100–4300 m. Vouchers: Dummer RA 3367 (EA), Bogdan 3399 (EA), Bogdan A 3941, 3945 (EA). Native distribution range: Macaronesia, Mediterranean to African Mountains, Europe to Caucasus, Tibet to W Himalaya.

***Andropogonamethystinus* Steud.** – Habit: Herb. Habitat: Alpine grasslands, montane grassland, moorland, 2000–4000 m. Vouchers: Bogdan A 4136 (EA), Liebenberg L 1686 (US). Native distribution range: Tropical & S Africa, Arabian Peninsula, S India, Myanmar.

***Andropogonlima* Stapf** – Habit: Herb. Habitat: Montane grassland, dry moorland, 2400–4000 m. Vouchers: Katende T 911 (MO), Hedberg 260, Bie SW 275 (EA), Osterkamp M 111 (EA), Bogdan A 4497 (EA), Melderis 260 (EA), Hedberg O 869 (EA). Native distribution range: Cameroon, South Sudan to Malawi.

***Anthoxanthumnivale* K.Schum.** – Habit: Herb. Habitat: Grassland, moorland, 2400–4800 m. Vouchers: Bogdan 3927 (K), 4494 (K), Liebenberg L 1701 (US), Hedberg O 191 (K). Native distribution range: NE DR Congo to E Tropical Africa.

***Arthraxonhispidus* (Thunb.) Makino** – Habit: Herb. Habitat: Grassland, woodland, 650–2600 m. Voucher: Snowden JD 1209 (US). Native distribution range: Tropical Africa, W Indian Ocean, Asia to E Australia.

***Arthraxonprionodes* (Steud.) Dandy** – Habit: Herb. Habitat: Rocky slopes, 1000–2000 m. Voucher: Human observation sn (EA). Native distribution range: NE & E Tropical Africa, Arabian Peninsula, Afghanistan to China and Peninsula Malaysia.

***Avenellaflexuosa* (L.) Drejer** – Habit: Herb. Habitat: Upland moorland, 2600–4300 m. Vouchers: Bogdan3950 (EA), Hedberg O 931 (K). Native distribution range: Europe to Japan and Malesian Mountains, Macaronesia, NW & Tropical Montane Africa, Greenland to Central & E America, S South America to Falkland Islands.

***Brachypodiumflexum* Nees** – Habit: Herb. Habitat: Upland Forest shade, bamboo thickets, bushland, 2000–3000 m. Voucher: Bogdan 5389 (EA). Native distribution range: Tropical & S Africa, Madagascar.

**^EX^*Brizamaxima* L.** – Habit: Herb. Habitat: Roadsides, cultivations, 2400–2700 m. Voucher: Agnew ADQ (2013) (REF). Native distribution range: Macaronesia to Mediterranean.

***Bromusleptoclados* Nees** – Habit: Herb. Habitat: Upland forest edge, grassland, 2300–4300 m. Vouchers: Bogdan A 4121 (K), 5391 (K), 5408 (K), Wesche K 1977 (K), Mwangangi OM 351 (K), Johnston HB 859 (BR), Hedberg O 223 (K), Dummer RA 3342 (US). Native distribution range: Cameroon to Eritrea and S Africa, SW Arabian Peninsula.

***Cenchrusgeniculatus* Thunb.** – Habit: Herb. Habitat: Upland pastures, old cultivation, roadsides, 1500–3500 m. Voucher: Snowden JD 1181 (K). Native distribution range: Nigeria to Eritrea and S Africa, SW Arabian Peninsula.

***Cenchrusriparius* (Hochst. ex A.Rich.) Morrone** – Habit: Herb. Habitat: Swamps, cultivation, 1400–2600 m. Vouchers: Snowden JD 1214 (EA), 1227 (EA, K). Native distribution range: Ethiopia to E Tropical Africa.

***Cenchrustrisetus* (Leeke) Morrone** – Habit: Herb. Habitat: Evergreen forest, 1000–2800 m. Voucher: Agnew ADQ (2013) (REF). Native distribution range: Ethiopia to Zimbabwe.

***Cenchrusunisetus* (Nees) Morrone** – Habit: Herb. Habitat: Bushland, wooded grassland, 300–2300 m. Vouchers: Snowden JD 1244 (EA, S), Maitland 1179 (EA). Native distribution range: Tropical & S Africa, Arabian Peninsula.

*Colpodiumhedbergii (Melderis) Tzvelev – Habit: Herb. Habitat: Upland moor, stream sides, 3580–4000 m. Vouchers: Hedberg O 908 (MO, S, K, UPS). Native distribution range: Ethiopia, Kenya.

***Crinipeslongifolius* C.E.Hubb.** – Habit: Herb. Habitat: Wet grassland, forest edges, 1869–2400 m. Vouchers: Dummer RA 3643 (EA), Thomas AS 296 (EA, MO), 2572 (EA). Native distribution range: Ethiopia, South Sudan to Uganda.

***Cymbopogongiganteus* Chiov.** – Habit: Herb. Habitat: Bushland, wooded grassland, 0–2300 m. Vouchers: Snowden JD 1246 (EA), Maitland 1179 (EA). Native distribution range: Tropical Africa to Botswana, Madagascar.

***Cynodonnlemfuensis* Vanderyst** – Habit: Herb. Habitat: Wet grasslands, 300–2300 m. Vouchers: Snowden JD 1218 (EA), Forbes LM 214 (EA). Native distribution range: Ethiopia to S Tropical Africa.

**^EX^*Cynodontransvaalensis* Burtt Davy** – Habit: Herb. Habitat: Weedy places, roadside, 1500–2600 m. Voucher: Snowden JD 1212 (EA). Native distribution range: S Africa.

*Deschampsiaangusta Stapf & C.E.Hubb. – Habit: Herb. Habitat: Streams in moorland, 3600–4300 m. Voucher: Bogdan 3937 (EA). Native distribution range: E Central Tropical Africa (Ruwenzori, Mt Elgon).

***Deschampsiacespitosa* (L.) P.Beauv.** – Habit: Herb. Habitat: Moorland, streambanks, 2900–4000 m. Voucher: Hedberg 860 (EA). Native distribution range: Subarctic & Temperate to Tropical Mountains.

***Digitariaabyssinica* (Hochst. ex A.Rich.) Stapf** – Habit: Herb. Habitat: Roadside, grassland, grazing land, 0–3000 m. Vouchers: Snowden JD 1232 (K), 1022 (US). Native distribution range: Tropical & S Africa to Sri Lanka, Vietnam, Peninsula Malaysia, New Guinea, SE Queensland.

***Echinochloapyramidalis* Hitchc. & Chase** – Habit: Herb. Habitat: Swamps, riverside, 0–2400 m. Voucher: Snowden JD 1092 (US). Native distribution range: Africa to Arabian Peninsula.

***Eleusineafricana* Kenn.-O’Byrne** – Habit: Herb. Habitat: Upland grassland, in cultivation, 1300 m. Voucher: Dummer RA 3655 (US). Native distribution range: Africa to Arabian Peninsula.

^**EX**^***Eleusinecoracana* Gaertn.** – Habit: Herb. Habitat: Upland grassland, in cultivation. Voucher: Snowden JD 1235, 1236 (US). Native distribution range: W Tropical Africa to Socotra and Angola.

***Eleusineindica* (L.) Gaertn.** – Habit: Herb. Habitat: Wet grassland, forest edges, 0–2070 m. Vouchers: Snowden JD 1110 (EA, US). Native distribution range: Tropical & Subtropical Old World.

*Eleusinejaegeri Pilg. – Habit: Herb. Habitat: Upland grassland, forest clearings, bushland, 1800–3300 m. Vouchers: Dummer RA 3655 (MO), Wesche K 1555 (MO). Native distribution range: Ethiopia to E Tropical Africa.

***Elionurusmuticus* (Spreng.) Kuntze** – Habit: Herb. Habitat: Open bushland, 1400–2800 m. Vouchers: Snowden JD 1260 (EA, US). Native distribution range: S Tropical America, Tropical & S Africa, SW Arabian Peninsula.

***Eragrostisexasperata* Peter** – Habit: Herb. Habitat: Moist grassland, rock outcrop, 300–2300 m. Voucher: Agnew ADQ (2013) (REF). Native distribution range: Kenya to S Tropical Africa.

***Eragrostisheteromera* Stapf** – Habit: Herb. Habitat: Roadsides, old cultivations, water logged soils, 1200–3000 m. Voucher: Agnew ADQ (2013) (REF). Native distribution range: Eritrea to S Africa.

***Eragrostishispida* K.Schum.** – Habit: Herb. Habitat: Swampy places, rock outcrop seepage, 1000–2600 m. Vouchers: Hemp A (W), Rono et al. SAJIT-PR 0019 (EA, HIB). Native distribution range: South Sudan to S Tropical Africa.

***Eragrostispaniciformis* (A.Braun) Steud.** – Habit: Herb. Habitat: Upland grassland, streamsides, swamp grassland, 1300–2600 m. Vouchers: Lugard EJ & Lugard C 604 (BR, US). Native distribution range: Eritrea to Zambia.

***Eragrostisracemosa* Steud.** – Habit: Herb. Habitat: Dry grassland, 1200–2300 m. Voucher: Agnew ADQ (2013) (REF). Native distribution range: Eritrea to S Africa, Seychelles, Madagascar.

***Eragrostisschweinfurthii* Chiov.** – Habit: Herb. Habitat: Grassland, 1300–3000 m. Voucher: Agnew ADQ (2013) (REF). Native distribution range: Eritrea to Malawi, SW Arabian Peninsula, Sri Lanka.

***Eriochloabarbatus* (Trin.) S.Yadav & M.R.Almeida** – Habit: Herb. Habitat: Depressions in swampy areas, 0–1800 m. Voucher: Human observation sn (EA). Native distribution range: Tropical & S Africa, Madagascar, Arabian Peninsula.

***Exothecaabyssinica* Andersson** – Habit: Herb. Habitat: Upland, montane grassland, 2000–4000 m. Vouchers: Hedberg 128 (EA), Mwangangi OM 355 (EA), Stein W Bie (UPS). Native distribution range: Eritrea to S Tropical Africa, Indo-China.

***Festucaabyssinica* Hochst.** – Habit: Herb. Habitat: Grassland, forest edges, moor, 2800–4300 m. Vouchers: Dale JRD (EA), Wilson J 1227 (K), A Thomas Th644 (US). Native distribution range: Gulf of Guinea Islands to Cameroon, Ethiopia to S Tropical Africa.

***Festucabromoides* L.** – Habit: Herb. Habitat: Wet grassland, forest edges, 2300–3900 m. Voucher: Dummer RA 3778 (EA). Native distribution range: Macaronesia, Europe to Caucasus, Sahara to Kenya, SW Cameroon, SW Arabian Peninsula.

**^EX^*Festucacamusiana* St.-Yves** – Habit: Herb. Habitat: Upland forest, bamboo tickets, 2100–3500 m. Vouchers: Bogdan 5386 (EA), Dummer RA 3506 (EA). Native distribution range: Madagascar.

***Festucachodatiana* (St.-Yves) E.B.Alexeev** – Habit: Herb. Habitat: Forest edge, grassland, bamboo thickets, 2100–3500 m. Voucher: Bogdan 5386 (EA). Native distribution range: Gulf of Guinea Islands, SW Cameroon, South Sudan (Imatong Mountains), Central Ethiopia to E Tropical Africa.

**Festucaclaytonii E.B.Alexeev – Habit: Herb. Habitat: Moorland of Elgon, above 2500 m. Voucher: Thomas SA 2727 (K). Native distribution range: Kenya.

***Festucamekiste* Clayton** – Habit: Herb. Habitat: upland forest, 2300–3000 m. Voucher: Bogdan 5390 (EA). Native distribution range: Bioko, SW Cameroon, Ethiopia.

***Festucaobturbans* St.-Yves** – Habit: Herb. Habitat: Upland moor, 2400–4000 m. Voucher: Mwangangi OM 312 (EA). Native distribution range: Kenya to Tanzania, Yemen.

*Festucapilgeri St.-Yves – Habit: Herb. Habitat: Dry upland moor, 2700–4250 m. Vouchers: Liebenberg 1699 (EA), Thomas AS 2729 (EA), Bogdan A 3942 (K, MO), Wesche K 257 (K, MO), Hedberg KO 1006 (EA, MO), HB Johnston 870 (US). Native distribution range: E Tropical Africa (Mountains).

***Festucasimensis* Hochst. ex A.Rich.** – Habit: Herb. Habitat: Upland Forest shade, bamboo thickets, 2000–3300 m. Voucher: Bogdan 5402 (EA). Native distribution range: Cameroon to Ethiopia and N Tanzania.

***Hyparrheniacymbaria* Stapf** – Habit: Herb. Habitat: Wooded grassland, forest, 1000–3000 m. Voucher: Snowden JD 1217, 1162 (US). Native distribution range: Nigeria to Eritrea and S Africa, Comoros, Madagascar, India.

***Hyparrheniadregeana* Stapf ex Stent** – Habit: Herb. Habitat: Grazed grasslands, 2000–2600 m. Voucher: Agnew ADQ (2013) (REF). Native distribution range: Eritrea to S Africa, SW Arabian Peninsula.

***Hyparrheniamobukensis* Chiov.** – Habit: Herb. Habitat: Subalpine forest, bamboo zone, 2500–3300 m. Vouchers: Dummer RA 3498 (EA), Thomas AS 2664 (EA, MO). Native distribution range: Ethiopia to S Tropical Africa.

***Hyparrheniarufa* Stapf** – Habit: Herb. Habitat: Bushlands, wooded grasslands, 0–2300 m. Voucher: Snowden JD 1029, 1102 (US). Native distribution range: Tropical & S Africa, W Indian Ocean, S Central China to Indo-China.

***Hyparrheniaschimperi* Andersson** – Habit: Herb. Habitat: Open moist bushland, wooded grassland, 700–1700 m. Vouchers: Snowden JD 1168 (EA). Native distribution range: Ethiopia to S Africa, Madagascar.

***Hypertheliadissoluta* (Nees) Clayton** – Habit: Herb. Habitat: Bushland, wooded grassland, 0–2400 m. Voucher: Snowden JD 1173 (US). Native distribution range: Tropical & S Africa, Madagascar.

***Koeleriacapensis* Nees** – Habit: Herb. Habitat: Upland grassland, moorland, 1800–4300 m. Vouchers: Liebenberg 1696 (EA), Tothill BH 2434 (EA), Granvik H (S), A Bogdan AB 3947 (US), Dummer RA 3341 (US). Native distribution range: Cameroon, Ethiopia to S Africa, SW Arabian Peninsula.

***Koordersiochloalongiarista* (A.Rich.) Veldkamp** – Habit: Herb. Habitat: Forest floor, heath zone, bamboo clearing, 1500–3000 m. Voucher: Agnew ADQ (2013) (REF). Native distribution range: Nigeria to Ethiopia and S Africa, Jawa to Lesser Sunda Islands, Philippines.

***Loudetiasimplex* (Nees) C.E.Hubb.** – Habit: Herb. Habitat: Rocky dry woodland, wooded grassland, 1000–2600 m. Vouchers: Snowden JD 1242 (US), Hemp A (W). Native distribution range: Tropical & S Africa, Madagascar.

***Melinisrepens* (Willd.) Zizka** – Habit: Herb. Habitat: Rocky grassland, 0–2500 m. Vouchers: Snowden JD 1090 (US, MO). Native distribution range: Africa to Arabian Peninsula.

***Micrachnepatentiflora* (Stent & J.M.Rattray) P.M.Peterson** – Habit: Herb. Habitat: Seasonally flooded shallow soils, 1400–2300 m. Voucher: Bogdan 4075 (BR, EA). Native distribution range: Kenya to Botswana.

***Microchloaindica* (L.f.) P.Beauv.** – Habit: Herb. Habitat: Upland grassland, bushland, 800–1600 m. Voucher: Agnew ADQ (2013) (REF). Native distribution range: Tropical & Subtropical Old World to N Australia.

***Moorochloaeruciformis* (Sm.) Veldkamp** – Habit: Herb. Habitat: Wet grassland, 500–2200 m. Voucher: Strange R 70 (EA). Native distribution range: Mediterranean to Indo-China and Africa.

***Oldeaniaalpina* (K.Schum.) Stapleton** – Habit: Herb (bamboo). Habitat: Montane forest, 2400–3000 m. Vouchers: Dummer RA 3508 (EA, US), Eggeling W 2472 (EA, US). Native distribution range: Ethiopia to Zambia.

***Oplismenushirtellus* (L.) P.Beauv.** – Habit: Herb. Habitat: Evergreen Forest, 0–2500 m. Vouchers: Snowden JD 1213 (EA, S) 1230 (US). Native distribution range: Tropics & Subtropics.

***Oxytenantheraabyssinica* Munro** – Habit: Herb (bamboo). Habitat: Grassland, woodland, thickets, 2400–3000 m. Voucher: Snowden JD 1051 (K, US). Native distribution range: Tropical & S Africa.

***Panicumchionachne* Mez** – Habit: Herb. Habitat: Forest, 1000–3000 m. Voucher: Agnew ADQ (2013) (REF). Native distribution range: South Sudan to S Tropical Africa.

***Panicumhochstetteri* Steud.** – Habit: Herb. Habitat: Forest, 1600–3300 m. Vouchers: Thomas AS 2665 (EA), Dummer RA 3567 (EA, US). Native distribution range: Sierra Leone to Eritrea and Tanzania.

***Panicummonticola* Hook.f.** – Habit: Herb. Habitat: Forest shade, 600–2800 m. Voucher: Agnew ADQ (2013) (REF). Native distribution range: Nigeria to Ethiopia and South Africa.

***Panicumpusillum* Hook.f.** – Habit: Herb. Habitat: Forest, grassland, bushland, 1350–3000 m. Voucher: Dummer RA 3551 (EA). Native distribution range: Tropical Africa.

***Pentamerisborussica* (K.Schum.) Galley & H.P.Linder** – Habit: Herb. Habitat: Upland grassland, moorland, 3000–4680 m. Vouchers: Dummer RA 3379 (EA), Thomas AS 2686 (EA), Wesche K 238 (K), 237 (K), Thomas AS 2686 (K), Bogdan A 3939 (K), Nouga 133 (K), Lisowski S 10468 ((BR), Katende 878 (K). Native distribution range: Ethiopia to E Tropical Africa.

***Pentamerisminor* (F.Ballard & C.E.Hubb.) Galley & H.P.Linder** – Habit: Herb. Habitat: Moorland, open grassland, moorland, 3000–4800 m. Voucher: Bodgan 4499 (EA), Bogdan A 3946, AB3940, AB4123 (K), Thomas AS 2721 (K, UPS), Hedberg O 4487 (K), Lisowski S 10468 (K), Dale IR 3189 (K), Mwangangi OM 314 (K), Knight J 4499 (K). Native distribution range: Ethiopia to E Tropical Africa.

***Pentamerispictigluma* (Steud.) Galley & H.P.Linder** – Habit: Herb. Habitat: Upland grassland, montane path side, 2600–4300 m. Vouchers: Wood GH 160 (EA), Wesche K 316 (K), 310 (K). Native distribution range: Cameroon, Ethiopia to Tanzania, Yemen.

***Poaannua* L.** – Habit: Herb. Habitat: Upland path sides, 2000–3550 m. Voucher: Agnew ADQ (2013) (REF). Native distribution range: Temperate Old World to Tropical Mountains.

***Poaleptoclada* Hochst. ex A.Rich.** – Habit: Herb. Habitat: Forest edges, upland grassland, heath, moor, 1800–4300 m. Vouchers: Hedberg O 1028 (NHMUK), Dummer RA 3524 (US), Liebenberg L 1697 (US). Native distribution range: Bioko to Eritrea and S Africa, Arabian Peninsula.

***Poaschimperiana* Hochst. ex A.Rich.** – Habit: Herb. Habitat: Stream sides, moorland, 2300–4300 m. Vouchers: Bogdan A 3933 (BR), (K), Wesche K 1335 (K), Liebenberg L 1698 (S). Native distribution range: Nigeria to Ethiopia and Malawi, Arabian Peninsula.

***Poecilostachysoplismenoides* (Hack.) Clayton** – Habit: Herb. Habitat: Shady forest, 1000–2500 m. Vouchers: Katende AB; Sheil 2290 (K). Native distribution range: Nigeria to Kenya and S Tropical Africa.

***Polypogonschimperianus* (Hochst. ex Steud.) Cope** – Habit: Herb. Habitat: Upland grassland, moorland, 1500–4000 m. Voucher: Hedberg 3580 (EA). Native distribution range: Ethiopia to S Tropical Africa, Arabian Peninsula.

***Pseudechinolaenapolystachya* (Kunth) Stapf in Prain** – Habit: Herb. Habitat: Upland forest shade, 1000–2500 m. Voucher: Snowden JD 1183 (EA). Native distribution range: Tropics & Subtropics.

***Pseudobromusafricanus* Stapf** – Habit: Herb. Habitat: Bamboo thickets, upland forest, 2100–2700 m. Voucher: Bodgan 2832 (EA). Native distribution range: Sudan to S Africa.

***Setariasphacelata* Stapf & C.E.Hubb. ex M.B.Moss** – Habit: Herb. Habitat: Wooded grasslands, swamps, riversides, 0–3300 m. Vouchers: Snowden JD 1103 (US, K), 1194 (US). Native distribution range: Tropical & S Africa, Madagascar.

***Sporobolusnervosus* Hochst.** – Habit: Herb. Habitat: Grazed bushland, 300–1830 m. Voucher: Human observation sn (EA). Native distribution range: Mauritania, Ethiopia to Tanzania, S Africa, Arabian Peninsula, Pakistan.

***Sporobolusolivaceus* Napper** – Habit: Herb. Habitat: Grassland, moorland, 2300–4000 m. Vouchers: Bogdan 3948, Dale in FD 3190 (EA), Liebenberg 1717 (EA). Native distribution range: Ethiopia to Zambia.

***Sporoboluspaniculatus* T.Durand & Schinz** – Habit: Herb. Habitat: Roadsides, bushland, wooded grassland, 1000–2000 m. Voucher: Snowden JD 1239 (EA, US). Native distribution range: Tropical & S Africa, Madagascar.

***Sporoboluspyramidalis* P.Beauv.** – Habit: Herb. Habitat: Disturbed grassland, 2000–3000 m. Voucher: Snowden JD 1097 (US). Native distribution range: Tropical & Subtropical America, Africa to Arabian Peninsula.

*Sporobolusscitulus Clayton – Habit: Herb. Habitat: Rare in swampy rock outcrop, at 2500 m. Voucher: Bogdan A AB 4072 (K). Native distribution range: Uganda to Kenya.

***Sporobolusstapfianus* Gand.** – Habit: Herb. Habitat: Bushlands, 500–2400 m. Voucher: Agnew ADQ (2013) (REF). Native distribution range: Niger to Ethiopia and S Africa, Madagascar.

**^EX^*Stipadregeana* Steud.** – Habit: Herb. Habitat: Upland forest shade, roadside, 2000–2600 m. Voucher: Agnew ADQ (2013) (REF). Native distribution range: South Africa.

***Themedatriandra* Forssk.** – Habit: Herb. Habitat: Grassland, 0–3000 m. Voucher: Hedberg O 1060 (S, EA), Tweedie 21 (EA), Snowden JD 1220, 1171 (US). Native distribution range: Africa, Tropical & Subtropical Asia to Australia.

***Tripogonmajor* Hook.f.** – Habit: Herb. Habitat: Upland rocky grassland, 1200–3700 m. Vouchers: Snowden JD 1187 (K), Bodgan 3932 (US, EA), 5419 (EA), Rono et al. SAJIT-PR 0111 (EA, HIB). Native distribution range: Sierra Leone, Nigeria to Ethiopia and Malawi, S India.

***Trisetopsisangusta* (C.E.Hubb.) Röser & A.Wölk** – Habit: Herb. Habitat: Upland grassland, 2300–2600 m. Voucher: Bogdan A (B). Native distribution range: Kenya, Yemen.

***Trisetopsismilanjiana* (Rendle) Röser & A.Wölk** – Habit: Herb. Habitat: Upland forest egde, bamboo thickets, 2300–3500 m. Voucher: Agnew ADQ (2013) (REF). Native distribution range: Ethiopia to Malawi, Madagascar.

***Trisetopsisumbrosa* (Hochst. ex Steud.) Röser & A.Wölk** – Habit: Herb. Habitat: Upland bamboo thickets, grassland, moor, 1850–4000 m. Vouchers: Bogdan 3929 (EA), 3936 (EA). Native distribution range: Ethiopia to E Tropical Africa.

***Urochloabrizantha* (A.Rich.) R.D.Webster** – Habit: Herb. Habitat: Grassland, wooded grassland, woodland, 300–2500 m. Voucher: Tweedie 20 (EA). Native distribution range: Tropical & S Africa, W Indian Ocean, SW Arabian Peninsula.

***Urochloacomata* (A.Rich.) Sosef** – Habit: Herb. Habitat: Bushland, 600–2200 m. Vouchers: Snowden JD 1202 (EA, US). Native distribution range: Tropical Africa, SW Arabian Peninsula.

***Urochloajubata* (Fig. & De Not.) Sosef** – Habit: Herb. Habitat: Bushlands, swamp margins, 500–2900 m. Voucher: Jack C 20 (EA). Native distribution range: Tropical Africa, Madagascar.

#### F31. POTAMOGETONACEAE

1 Genus, 2 species

**^EX^*Potamogetonnodosus* Poir.** – Habit: Herb. Habitat: Fresh water wetlands, 1510–4250 m. Voucher: Agnew ADQ (2013) (REF). Native distribution range: Cosmopolitan.

***Potamogetonrichardii* Solms** – Habit: Herb. Habitat: Fresh water wetlands, 1150–3450 m. Vouchers: Ross R 1346 (BR, EA). Native distribution range: Cameroon, Eritrea to S Africa, Central Madagascar.

#### F32. TYPHACEAE

1 Genus, 1 species

***Typhalatifolia* L.** – Habit: Herb. Habitat: Permanent swamps, river, 1300–2200 m. Voucher: Snowden JD 1223 (EA). Native distribution range: Temperate Northern Hemisphere to Colombia, W Bolivia to S South America, Nigeria to Kenya.

#### F33. XYRIDACEAE

1 Genus, 2 species

***Xyriscapensis* Thunb.** – Habit: Herb. Habitat: Upland marsh, bogs, 1100–3000 m. Vouchers: Tweedie EM (1976) (REF), Agnew ADQ (2013) (REF). Native distribution range: Tropical & Subtropical Old World, Brazil.

***Xyrisstraminea* L.A.Nilsson** – Habit: Herb. Habitat: Wet grassland, outcrop rock, 1750–2400 m. Voucher: Wood GHS 477 (K). Native distribution range: Tropical & S Africa.

### ﻿Part 5 Magnoliopsida

#### F34. ACANTHACEAE

17 Genera, 45 species

***Acanthopalepubescens* C.B.Clarke** – Habit: Shrub or shrubby herb. Habitat: Wet montane forest, forest swamps, 1655–2790 m. Voucher: Dale IR 50 (EA, BR). Native distribution range: Ethiopia to S Tropical Africa.

***Acanthuseminens* C.B.Clarke** – Habit: Woody shrub. Habitat: Forest margins, undergrowth of montane forest, scrub, thicket clumps in dump areas, 1500–2800 m. Vouchers: Snowden JD 828 (EA), Bamps PRJ 6497 (WAG). Native distribution range: Ethiopia to South Sudan and Central Kenya.

***Acanthuspolystachius* Delile** – Habit: Shrub. Habitat: Montane wet evergreen forest, woodland, forest margins, rocky hills, 1100–2800 m. Vouchers: Dale 50 (EA), James E sn (K). Native distribution range: Ethiopia to NW Tanzania.

***Barleriagrandicalyx* Lindau** – Habit: Herb. Habitat: Dry savanna, wooded grassland, acacia combraetum woodland, 1100–2500 m. Vouchers: Tweedie EM (1976) (REF), Agnew ADQ (2013) (REF). Native distribution range: Central African Republic to Ethiopia and Tanzania, Angola.

***Barleriaventricosa* Hochst. ex Nees** – Habit: Herb or shrub. Habitat: Forest undergrowth, woodland, thickets, in grassland, 1700–3590 m. Vouchers: Tweedie 131 (K), 838 (K), Rono et al. SAJIT-PR 0218 (EA, HIB). Native distribution range: Eritrea to S Africa, Yemen.

***Crabbeavelutina* S.Moore** – Habit: Herb. Habitat: Acacia bushland, riverine forest, thickets, 1750–2250 m. Voucher: Tweedie 1828 (EA). Native distribution range: S Ethiopia to S Africa.

^**EX**^***Diclipteracolorata* C.B.Clarke** – Habit: Herb. Habitat: Woodland, forest, 1750–3000 m. Voucher: Tweedie EM (1976) (REF). Native distribution range: Rwanda to N Malawi.

***Diclipteralaxata* C.B.Clarke** – Habit: Herb. Habitat: Woodland, undergrowth mountain rain forest, 1750–3000 m. Voucher: Tweedie EM 828 (K). Native distribution range: Nigeria to Ethiopia and N Malawi.

*Diclipteramaculatasubsp.usambarica (Lindau) I.Darbysh. – Habit: Herb. Habitat: Forest margins, wooded grasslands, disturbed areas, montane and submontane forest, (1150 –)1300–2700(–3050). Vouchers: Dummer RA 3618 (EA), Naiga 505A (EA). Native distribution range: E Uganda to N Tanzania.

*Diclipteranilotica C.B.Clarke – Habit: Herb. Habitat: Recently burnt areas on roadsides and open woodland, short grassland, 1050–2350 m. Voucher: Tweedie 1524 (EA). Native distribution range: Uganda to W Kenya.

***Diclipterapumila* (Lindau) Dandy** – Habit: Herb. Habitat: Short grassland, open woodland, recently burnt montane grassland, 1750–2850 m. Voucher: Tweedie EM (1976) (REF). Native distribution range: SW Ethiopia to S Tropical Africa.

***Dyschoristemulticaulis* Kuntze** – Habit: Herb. Habitat: Disturbed grassland, 1705–2400 m. Voucher: Agnew ADQ (2013) (REF). Native distribution range: Eritrea to E Central & E Tropical Africa, Yemen.

***Dyschoristeradicans* Nees** – Habit: Herb. Habitat: Low altitude grassland, 1750–2500 m. Voucher: Tweedie EM (1976) (Ref). Native distribution range: Tropical Africa, SW Arabian Peninsula.

***Hygrophilaauriculata* (Schumach.) Heine** – Habit: Herb. Habitat: Swamps, wet savanna and open woodland, 1750–2250 m. Voucher: Tweedie EM (1976) (REF). Native distribution range: Tropical & S Africa, Indian Subcontinent to Indo-China.

***Hypoestesaristata* (Vahl) Roem. & Schult.** – Habit: Herb. Habitat: Forest margins and clearings, open woodland, wet grassland, 1750–3000 m. Vouchers: Tweedie EM 812 (K), 823 (K), 829 (K). Native distribution range: Tropical & S Africa.

**Hypoestesforskaoliisubsp.hildebrandtii (Lindau) I.Darbysh.** – Habit: Herb. Habitat: Dry grassland, bushland, forest edges, 1750–2820 m. Voucher: Tweedie EM (1976) (REF). Native distribution range: NE Tropical Africa to Kenya.

***Hypoestestriflora* Roem. & Schult.** – Habit: Herb. Habitat: Forest margins and undergrowth, forest clearings and grassland, 1600–3300 m. Vouchers: Dawkins 777 (EA), Tweedie EM 830 (K), Tweedie 886 (K), Vollesen K 682 (K), Townsend CC 2335 (BR), Rono et al. SAJIT-PR 0204 (EA, HIB). Native distribution range: Tropical & S Africa, SW Arabian Peninsula, Himalaya to China (Yunnan) and Indo-China.

***Isoglossagregorii* (S.Moore) Lindau** – Habit: Herb. Habitat: Higher montane forest, bamboo zone undergrowth, woodland, 1790–2900 m. Vouchers: Wesche K 587 (EA), Lye 25497 (EA), Mwangangi OM 453 (BR). Native distribution range: Kenya to E Zimbabwe.

***Isoglossalaxa* Oliv.** – Habit: Herb. Habitat: Montane Forest understory, clearances, secondary growth, 1750–2550 m. Voucher: Tweedie EM (1976) (REF). Native distribution range: Ethiopia to N Tanzania, Madagascar.

***Isoglossapunctata* (Vahl) Brummitt & J.R.I.Wood** – Habit: Herb. Habitat: Montane rain forest undergrowth, margin, secondary bushland, 1350–2750 m. Vouchers: Tweedie 1896 (EA), Dale IR U89 (BR). Native distribution range: Ethiopia to E Central & E Tropical Africa, SW Arabian Peninsula.

***Isoglossasubstrobilina* C.B.Clarke** – Habit: Herb. Habitat: Montane rain forest, woodland, 1600–2600 m. Vouchers: Tweedie 891 (K), Napper DM & Tweedie EM 2128 (BR). Native distribution range: E Tropical Africa.

***Justiciaanagalloides* T.Anderson** – Habit: Herb. Habitat: Grassland, woodland bushland, 1020–2500 m. Voucher: Tweedie EM 512 (K). Native distribution range: Ethiopia to S Africa.

***Justiciaanselliana* T.Anderson** – Habit: Herb. Habitat: Marshy grassland, 1300–1920 m. Vouchers: Tweedie EM (1976) (REF), Agnew ADQ (2013) (REF). Native distribution range: Tropical & S Africa.

***Justiciacalyculata* Deflers** – Habit: Herb. Habitat: Roadside, lawn, grassland weed, 100–2200 m. Vouchers: Agnew ADQ (2013) (REF), Rono et al. SAJIT-PR 0014 (EA, HIB). Native distribution range: NE Tropical Africa to N Tanzania, S Arabian Peninsula.

***Justiciadiclipteroides* Lindau** – Habit: Herb. Habitat: Woodland edges, forest, 1300–2325 m. Voucher: Rono et al. SAJIT-PR 0170 (EA, HIB). Native distribution range: Ethiopia to E Central & E Tropical Africa.

***Justiciaeminii* Lindau** – Habit: Shrubby herb. Habitat: Woodland and bushland on rocky hill, forest margin, montane grassland, riverine woodland, 1200–2100 m. Voucher: Tweedie 1723 (EA). Native distribution range: Uganda to S Tropical Africa.

***Justiciaexigua* S.Moore** – Habit: Herb. Habitat: Wet savanna, outcrop rock, 1720–2400 m. Voucher: Tweedie EM (1976) (REF). Native distribution range: Ethiopia to South Africa.

***Justiciaflava* Vahl** – Habit: Herb. Habitat: Open habitat, savanna, woodland, 1750–2500 m. Voucher: Tweedie EM 674 (K). Native distribution range: Tropical & S Africa, SW Arabian Peninsula.

**^EX^*Justiciainsularis* T.Anderson** – Habit: Herb. Habitat: Wet savanna, 1750–2250 m. Voucher: Tweedie EM (1976) (REF). Native distribution range: W Tropical Africa to Chad.

***Justiciastriata* (Klotzsch) Bullock** – Habit: Herb. Habitat: Evergreen woodland edges, bushed grassland, 2000–2600 m. Voucher: Tweedie EM 3673 (K). Native distribution range: Tropical Africa.

***Justiciaunyorensis* S.Moore** – Habit: Herb. Habitat: Edges of montane rain forest, montane grassland, woodland, bushland, 2170–3100 m. Vouchers: Tweedie 3717 (EA), Bridson DM 67 (WAG). Native distribution range: Nigeria to SW Ethiopia and NW Tanzania.

***Lepidagathiscollina* (Endl.) Milne-Redh.** – Habit: Herb or subshrub. Habitat: Open fire prone grass, rocky outcrop, 1750–2170 m. Voucher: Tweedie EM (1976) (REF). Native distribution range: W Tropical Africa to Eritrea and Kenya.

***Lepidagathisglandulosa* Nees** – Habit: Herb or subshrub. Habitat: Open wooded grassland, woodland, 850–2350 m. Vouchers: Tweedie 1370 (EA), Tweedie 1370A (K). Native distribution range: Cameroon to Ethiopia and Zambia.

***Lepidagathisscariosa* Nees** – Habit: Herb or subshrub. Habitat: Wet savanna, woodland, 1750–2400 m. Voucher: Tweedie EM (1976) (REF). Native distribution range: Dry Tropical Africa to Namibia, Arabian Peninsula, Madagascar.

***Mimulopsisalpina* Chiov.** – Habit: Woody herb. Habitat: Montane forest, bushland, bamboo forest, ericaceous zone, 1900–3300 m. Vouchers: Dummer RA 3464 (EA), Tweedie 1379 (EA), Dale IR 65 (BR), Thomas AS 2744 (BR), Mwangangi OM 391 (BR). Native distribution range: E Tropical Africa to N Malawi.

***Mimulopsisarborescens* C.B.Clarke** – Habit: Shrub or tree. Habitat: Wet montane forest, bamboo forest, along clearings, gaps, streams, 1750–3000 m. Vouchers: Hancork 216/37 (EA). Native distribution range: Kenya to E DR Congo, Mozambique.

***Mimulopsissolmsii* Schweinf.** – Habit: woody herb or undershrub. Habitat: Montane forest, bamboo forest, secondary forest, grassland, bushland, 1500–2700 m. Vouchers: Tweedie 2234 (EA), Snowden JD 947 (EA), Tweedie EM 1090 (K), Rono et al. SAJIT-PR 0222 (EA, HIB). Native distribution range: Tropical Africa.

***Monechmadebile* Nees** – Habit: Herb. Habitat: Montane grassland, woodland, 1750–2400 m. Voucher: Tweedie EM (1976) (REF). Native distribution range: Eritrea to S Africa, Arabian Peninsula, India.

***Nicotebabetonica* Lindau** – Habit: Herb or shrub. Habitat: Wet forest, riverside, 700–2200 m. Voucher: Agnew ADQ (2013) (REF). Native distribution range: Tropical & S Africa, Indian Subcontinent.

***Phaulopsisimbricata* (Forssk.) Sweet** – Habit: Herb. Habitat: Woodland, forest, 1750–2400 m. Vouchers: Irwin PH 53, Wesche K 1877 (K), Tweedie 803 (K). Native distribution range: Tropical & S Africa, Comoros, Madagascar, Réunion, SW Arabian Peninsula.

***Ruelliapatula* Jacq.** – Habit: Shrubby herb or subshrub. Habitat: Bushland, grassland, open places in forest, 1750–2100 m. Voucher: Polhill 402 (EA). Native distribution range: Tropical & S Africa to Indian Subcontinent to Indo-China.

***Thunbergiaalata* Bojer ex Sims** – Habit: Trailing or twinning herb. Habitat: Disturbed areas, secondary vegetation, wet forest, bushlands and thickets, woodland and forest, 1750–3000 m. Vouchers: Symes YE 380, 365 (EA), Lugard EJ 65, 52 (EA), Mwangangi OM 515 (EA). Native distribution range: Tropical & S Africa, Madagascar.

***Thunbergiabattiscombei* Turrill** – Habit: Herb. Habitat: Burnt grassland, wooded grassland, 1500–2500 m. Voucher: Mr and Mrs Tweedie 281 (BR). Native distribution range: South Sudan to Kenya.

***Thunbergiagregorii* S.Moore** – Habit: Herb. Habitat: Montane grasslands, bushlands and forest edges, 1350–2300 (–2450). Voucher: Katende & Sheil 1921 (EA). Native distribution range: S Ethiopia to Burundi and Tanzania.

***Thunbergiapaulitschkeana* Beck** – Habit: Herb. Habitat: Grassland, bushlands, montane forest, roadsides, 1450–2800 m. Vouchers: Lye & Katende 6407 (EA), Tweedie 3674 (EA), Rono et al. SAJIT-PR 0118 (EA, HIB). Native distribution range: E & S Ethiopia to E Tropical

#### F35. AIZOACEAE

1 Genus, 1 species

***Zaleyapentandra* (L.) C.Jeffrey** – Habit: Succulent herb. Habitat: Woodland, grassland, waste places, cultivated ground, roadside, on alkaline soils, 800–2000 m. Voucher: Agnew ADQ (2013) (REF). Native distribution range: Africa to Israel and Arabian Peninsula, Pakistan to India.

#### F36. AMARANTHACEAE

6 Genera, 12 species

***Achyranthesaspera* L.** – Habit: Herb. Habitat: Wet savanna, woodland, forest, 0–3080 m. Vouchers: Katende 1012 (K). Native distribution range: Tropical & Subtropical Old World.

**Achyranthesasperavar.sicula L.** – Habit: Herb. Habitat: Mist forest, cultivation or disturbed ground, hardpan patches between rocks, seasonal swamps, open grassland, along forest edges and trails, 0–3000 m. Voucher: Dummer RA 3486 (EA). Native distribution range: Macaronesia to W Asia and Arabian Peninsula, Africa.

**^EX^*Amaranthushybridus* L.** – Habit: Herb. Habitat: Cultivated weed, wet savanna, introduced, 1500–2600 m. Vouchers: Hooper SS; Townsend, CC 1380 (WAG). Native distribution range: S Ontario to W South America.

***Celosiaschweinfurthiana* Schinz** – Habit: Herb. Habitat: Forest ridges, clearings and margins, thicker forest, 0–1650 m. Voucher: Human observation sn (EA). Native distribution range: Ethiopia to S Tropical Africa.

**Chenopodiastrumfasciculosumvar.schimperi (Asch.) Mosyakin** – Habit: Herb. Habitat: Weed in cultivated areas, waste places, evergreen upland forest, roadside, 1300–2600 m. Vouchers: Lugard EJ 272 (MO), (K). Native distribution range: Ethiopia to N Tanzania.

^**EX**^***Chenopodiastrummurale* (L.) S.Fuentes, Uotila & Borsch** – Habit: Herb. Habitat: Wet savanna, 1520–2750 m. Voucher: Tweedie EM (1976) (REF). Native distribution range: Macaronesia, Europe, Mediterranean to NE Tropical Africa and Sri Lanka.

**^EX^*Chenopodiumalbum* L.** – Habit: Herb. Habitat: Weed in cultivation, upland evergreen forest, 1650–2600 m. Voucher: Agnew ADQ (2013) (REF). Native distribution range: Temperate Eurasia to Indian Subcontinent.

***Chenopodiumopulifolium* Schrad. ex W.D.J.Koch & Ziz** – Habit: Herb. Habitat: Weed of cultivation, upland evergreen forest, 1600–2300 m. Voucher: Agnew ADQ (2013) (REF). Native distribution range: Europe, Mediterranean to Nepal, Eritrea to S Tropical Africa.

***Cyathulacylindrica* Moq.** – Habit: Shrubby herb. Habitat: Podocarpus-bamboo zone, forest edge and rocky scarps, 1300–3240 m. Voucher: Beentje HJ 1990 (WAG), Rono et al. SAJIT-PR 0179 (EA, HIB). Native distribution range: Tropical & S Africa, Madagascar.

***Cyathulauncinulata* (Schrad.) Schinz** – Habit: Prostrate or climbing herb or shrub. Habitat: Dense forest and forest edge, 1500–2900 m. Voucher: Stein W Bie (BOT), Rono et al. SAJIT-PR 0216 (EA, HIB). Native distribution range: Tropical & S Africa, Madagascar.

***Dysphaniaprocera* (Hochst. ex Moq.) Mosyakin & Clemants** – Habit: Herb. Habitat: Upland grassland, waste places, weed of cultivation, 1000–2800 m. Voucher: Dummer RA 3630 (EA). Native distribution range: Ethiopia to Zimbabwe, SW Arabian Peninsula.

***Dysphaniaschraderiana* (Schult.) Mosyakin & Clemants** – Habit: Herb. Habitat: Wet savanna, 1750–2300 m. Vouchers: Hedberg O 7 (BR), Rono et al. SAJIT-PR 0095 (EA, HIB). Native distribution range: Eritrea to S Africa, Arabian Peninsula, Pakistan.

#### F37. ANACARDIACEAE

3 Genera, 8 species

***Lanneaedulis* Engl.** – Habit: Shrublet. Habitat: Deciduous woodland, wooded and open grassland, prone to fire and floods, 1750–2250 m. Voucher: Tweedie EM (1976) (REF). Native distribution range: Ivory Coast, W Ethiopia to S Africa.

***Lanneaschimperi* Engl.** – Habit: Tree. Habitat: Wooded savanna and deciduous woodland, 1750–2250 m. Voucher: Dale 3204 (BR). Native distribution range: Nigeria to Eritrea and S Tropical Africa.

**Ozoroainsignissubsp.reticulata (Baker f.) J.B.Gillett** – Habit: Tree or shrub. Habitat: Wooded grassland, bushland, bushland, 1750–2250 m. Vouchers: Snowden JD 1062 (EA), Goldschmidt W 17 (EA), Jarchner HM 6960 (EA) Chalufack W 281 (EA). Native distribution range: South Sudan to Namibia.

***Searsialongipes* (Engl.) Moffett** – Habit: Shrub or small tree. Habitat: Riverine Forest, wet savanna, woodland, 1750–2400 m. Voucher: Ross R 1352 (BR). Native distribution range: Tropical Africa.

*Searsialongipesvar.elgonensis (Kokwaro) Moffett – Habit: Shrub or small tree. Habitat: Upland bushland, forest edges, riverine associations, 1800–2600 m. Vouchers: Eggeling 5761 (EA), Jackson THE 312 (EA, K), Lugard EJ sn (EA, K). Native distribution range: Uganda to W Central Kenya.

^**EX**^***Searsianatalensis* (Bernh. ex Krauss) F.A.Barkley** – Habit: Shrub or small tree. Habitat: Deciduous and evergreen forest, riverine associations, forest edges, 1750–3000 m. Vouchers: Lugard 136 (EA), Taiti S 611 (BR). Native distribution range: Coastal Mozambique to South Africa.

***Searsiapyroides* (Burch.) Moffett** – Habit: Shrub or small tree. Habitat: Wet savanna and woodland, 1750–2700 m. Vouchers: Jackson 326 (EA), Podwa SH 11 (EA), Jackson HE & Lugard EJ 326 (EA), Rono et al. SAJIT-PR 0035 (EA, HIB). Native distribution range: Ethiopia to S Africa.

***Searsiaruspolii* (Engl.) Moffett** – Habit: Shrub or small tree. Habitat: Upland evergreen bushland, forest edges, riverine associations, 1750–2400 m. Voucher: Dale IR 3195 (BR). Native distribution range: Ethiopia to E DR Congo.

#### F38. ANNONACEAE

2 Genera, 2 species

***Annonasenegalensis* Pers.** – Habit: Shrub or small tree. Habitat: Evergreen Forest along riverine fringes, 1140–1800 m. Vouchers: Goldschmidt W 22 (EA), Snowden JD 1045(EA). Native distribution range: Africa

***Monodoramyristica* Dunal** – Habit: Tree. Habitat: Evergreen forest of riverine fringing type, 1550–1650 m. Voucher: Childs- clarke B 337 (EA). Native distribution range: West Tropical Africa to SW Kenya and NW Angola.

#### F39. APIACEAE

18 Genera, 25 species

*Afroligusticumaculeolatum (Engl.) P.J.D.Winter – Habit: Herb. Habitat: Montane forest, woodland, 1900–3030 m. Vouchers: Snowden JD 453 (EA), Tweedie EM 1897. Native distribution range: E Central & E Tropical African Mountains.

*Afroligusticumelgonense (H.Wolff) P.J.D.Winter – Habit: Herb. Habitat: Montane forest edges, glades, bamboo forest margins, damp grasslands, streamside, marshes in subalpine zones, 1640–3300 m. Vouchers: Tothill BH 2317 (EA), Tweedie 3409, Synge PM S1004 (BR), Tothill BH 2317 (EA). Native distribution range: E Tropical Africa (Mountains).

***Afrosciadiumkerstenii* (Engl.) P.J.D.Winter** – Habit: Herb. Habitat: Beside streams in montane zones, bamboo zone, 2700–4300 m. Vouchers: Thomas AS 2333 (EA), Naiga 74 (K). Native distribution range: E Central & E Tropical African Mountains.

***Alepideapeduncularis* Steud. ex A.Rich.** – Habit: Herb. Habitat: Montane grassland, burnt areas, shallow soils, 1530–3600 m. Voucher: Rono et al. SAJIT-PR 0139 (EA, HIB). Native distribution range: Ethiopia to S Africa.

**^EX^*Ammimajus* L.** – Habit: Herb. Habitat: Disturbed ground and cultivation, at 1680 m. Voucher: Agnew ADQ (2013) (REF). Native distribution range: Macaronesia, Mediterranean to Iran and Arabian Peninsula.

***Anthriscussylvestris* (L.) Hoffm.** – Habit: Herb. Habitat: Bamboo, forest zones, 2100–3970 m. Vouchers: Katende T; Sheil D 817 (K), Thomas AS 2746 (BR), Synge PM S951 (BR), Naiga 228 (K), Kisalye N; van Heist M 547 (K), Rono et al. SAJIT-PR 0117 (EA, HIB). Native distribution range: Ethiopia to S Africa.

***Cryptotaeniaafricana* Drude** – Habit: Herb. Habitat: Wet montane forest floor, 1600–3000 m. Voucher: Hedberg O (BOT). Native distribution range: Nigeria to SW Ethiopia and Tanzania.

***Daucusincognitus* (C.Norman) Spalik, Reduron & Banasiak** – Habit: Herb. Habitat: Forest edges, grassland, 1600–3600 m. Vouchers: Agnew ADQ 7268 (MO), Tiyoy L 1481 (K), Rono et al. SAJIT-PR 0037 (EA, HIB). Native distribution range: Ethiopia to S Tropical Africa.

***Daucusmelananthus* (Hochst.) Reduron, Spalik & Banasiak** – Habit: Herb. Habitat: Woodland, upper forest edge, forest glades, 1750–3600 m. Vouchers: Hedberg O 445, 1953 (EA). Native distribution range: Tropical & S Africa, Madagascar, SW Arabian Peninsula.

***Ferulacommunis* L.** – Habit: Herb. Habitat: Disturbed areas of evergreen woodland, 1500–3720 m. Vouchers: Gillett JB 20955 (BR), Lugard EJ; Lugard C 425 (K). Native distribution range: Mediterranean to Arabian Peninsula and Tanzania.

***Haplosciadiumabyssinicum* Hochst.** – Habit: Herb. Habitat: Disturbed places, alpine, upland grassland, 2150–4600 m. Vouchers: Tweedie EM (1976) (REF), Agnew ADQ (2013) (REF). Native distribution range: Ethiopia to E Tropical African Mountains.

***Heracleumabyssinicum* (Boiss.) C.Norman** – Habit: Herb. Habitat: Upland grassland, forest margins, volcanic crater, burnt bushlands in the alpine zone, 1680–3790 m. Vouchers: Dummer RA 3712 (EA), Lugard EJ; Lugard C 425, Gillett JB 20953 (EA). Native distribution range: Eritrea to N Malawi.

*Heracleumelgonense (H.Wolff) Bullock – Habit: Herb. Habitat: Alpine zones, marshes, open grassy woodland, 1080–4200 m. Vouchers: Clifford C Townsend CC 2327 (MO), Wesche K 1715 (MO), Tothill BH 2384 (MO), Dummer RA 3527 (EA, MO), Thomas AS 522 (MO), Hooper SS|Clifford C Townsend CC 1381 (MO), Gillett JB 18487 (BR), Tothill BH 1939 (EA), Synge PM 925, 1003 (MO), Taylor G 3439 (MO), Hedberg KO 217 (MO), Mwangangi OM 353 (BR). Native distribution range: Ethiopia to E Tropical African Mountains.

**Heteromorphaarborescensvar.abyssinica (Hochst. ex A.Rich.) H.Wolff** – Habit: Herb, shrub or tree. Habitat: Woodland, 1750–2400 m. Vouchers: Tweedie 1423 (EA), Rono et al. SAJIT-PR 0088 (EA, HIB). Native distribution range: Eritrea to S Africa, Yemen.

***Lefebvreaatropurpurea* (A.Rich.) P.J.D.Winter** – Habit: Herb. Habitat: Upland pasture, disturbed places, scattered bushes, scrub, 1800–2600 m. Voucher: Agnew ADQ (2013) (REF). Native distribution range: Ethiopia to Uganda.

***Lefebvrealongipedicellata* Engl.** – Habit: Herb. Habitat: Grasslands at forest edge, roadside and shades in upland forest, 1860–2250 m. Voucher: Agnew ADQ (2013) (REF). Native distribution range: Uganda to Mozambique.

***Oenanthepalustris* (Chiov.) C.Norman** – Habit: Herb. Habitat: Open waters and by streamside at or in forest edge, 1200–3260 m. Voucher: Agnew ADQ (2013) (REF). Native distribution range: Ethiopia to E Central & E Tropical Africa.

*Oreoschimperellaaberdarensis (C.Norman) Rauschert – Habit: Herb. Habitat: Disturbed places of montane forest and stream edges, 2200–2910 m. Voucher: Agnew ADQ (2013) (REF). Native distribution range: Kenya.

***Pimpinellahirtella* A.Rich.** – Habit: Herb. Habitat: Cultivated grounds, open forest, on stony rock ground, upland grassland, 1800–2850 m. Voucher: Tweedie 1285 (EA). Native distribution range: Guinea to Mali, Nigeria to Cameroon, Eritrea to N Tanzania.

***Pimpinellakeniensis* C.Norman** – Habit: Herb. Habitat: Woodland, swampy grounds, 1750–2400 m. Vouchers: Tweedie DR 1177 (BR), Rono et al. SAJIT-PR 0185 (EA, HIB). Native distribution range: Kenya to N Tanzania.

*Pimpinellalindblomii H.Wolff – Habit: Herb. Habitat: Open montane forest, upland grassland, mountain slope, well-drained soil, 1530–2600 m. Vouchers: Tweedie 1177 (EA), Gerh. Lindblom sn (S). Native distribution range: Uganda to Kenya.

***Pimpinellaoreophila* Hook.f.** – Habit: Stoloniferous herb. Habitat: Upper montane forest, Streams in bamboo forest, heath zone, alpine grassland, 3000–4100 m. Vouchers: Dummer RA 3313 (EA), Thomas AS 634 (EA), Hedberg KO 4500 (BR), Naiga 84 (K), Katende T; D Sheil 907 (K), Rose F 10203 (BR), Taylor G 3476 (EA), Tweedie DR 118 (EA). Native distribution range: Bioko, SW Cameroon, Ethiopia to N Tanzania.

***Saniculaelata* Buch.-Ham. ex D.Don** – Habit: Herb. Habitat: Shady Forest floor of bamboo zone and adjacent forest, 1500–3220 m. Vouchers: Naiga 241 (K), Beentje HJ 1972 (WAG). Native distribution range: Eritrea to S Africa, W Indian Ocean, SW Arabian Peninsula, Tropical & Subtropical Asia.

***Steganotaeniaaraliacea* Hochst.** – Habit: Tree. Habitat: Wet savanna, 1750–2250 m. Voucher: Tweedie EM (1976) (REF). Native distribution range: Tropical & S Africa.

**^EX^*Torilisarvensis* (Huds.) Link** – Habit: Herb. Habitat: Along paths of upland forests, 1560–2850 m. Vouchers: Tweedie EM (1976) (REF), Agnew ADQ (2013) (REF). Native distribution range: Europe to Central Asia and Pakistan, Macaronesia, N Africa to Arabian Peninsula.

#### F40. APOCYNACEAE

23 Genera, 39 species

***Asclepiasfulva* N.E.Br.** – Habit: Herb. Habitat: Grassland, mixed deciduous woodland, 1750–2250 m. Voucher: Tweedie EM 565 (K). Native distribution range: Uganda to S Africa.

***Aspidoglossumangustissimum* (K.Schum.) Bullock** – Habit: Herb. Habitat: Wooded grassland, openwoodland, marshy areas, 1200–2250 m. Voucher: Tweedie EM 918 (K). Native distribution range: Cameroon to South Sudan and Zimbabwe.

***Aspidoglossummasaicum* (N.E.Br.) Kupicha** – Habit: Herb. Habitat: Grasslands, 1700–2800 m. Voucher: Agnew ADQ (2013) (REF). Native distribution range: S Ethiopia to NE Namibia.

***Carissaspinarum* L.** – Habit: Scrambling spiny shrub. Habitat: Forest edges, wet savanna, 1200–2250 m. Vouchers: Tweedie EM (1976) (REF), Agnew ADQ (2013) (REF). Native distribution range: Africa to Indo-China, Australia to New Caledonia.

***Ceropegiaabyssinica* Decne.** – Habit: Twiner herb. Habitat: Outcrop rock, grassland, 1800–2400 m. Vouchers: Tweedie EM 767 (K), Tweedie 819 (K). Native distribution range: Central African Republic to Eritrea and S Tropical Africa.

***Ceropegiacufodontii* Chiov.** – Habit: Climbing Herb. Habitat: Forest edge, rocky grassland and bushland, 1450–2350 m. Voucher: Tweedie EM 865 (K). Native distribution range: Ethiopia, Kenya, Uganda.

***Ceropegiadenticulata* K.Schum. ex Engl.** – Habit: Twiner herb. Habitat: Outcrop rock, 1800–2400 m. Voucher: Tweedie EM (1976) (REF). Native distribution range: E Tropical Africa.

**Ceropegiafilicorona Masinde – Habit: Twiner herb. Habitat: Bushland in stony ground, 1750–1900 m. Voucher: Irwin PH 339 (K). Native distribution range: Kenya.

***Ceropegiajohnstonii* (N.E.Br.) Bruyns** – Habit: Herb. Habitat: Wet grassland, rock outcrop, 1800–2250 m. Vouchers: Tweedie 2017 (EA), Tweedie, M. 3979 (K), Tweedie 2137 (K). Native distribution range: W Tropical Africa to Kenya.

***Ceropegiameyeri-johannis* Engl.** – Habit: Twiner herb. Habitat: Forest edge and clearing, thickets, woodland, 940–2500 m. Voucher: Rono et al. SAJIT-PR 0090 (EA, HIB). Native distribution range: Kenya to Zimbabwe.

***Cynanchumabyssinicum* Decne.** – Habit: Climbing shrub. Habitat: Upland rain forest edges, woodland, 2000–3000 m. Voucher: Tweedie EM 758 (K). Native distribution range: Eritrea to DR Congo and Tanzania

***Cynanchumaltiscandens* K.Schum.** – Habit: Climbing shrub. Habitat: Upland forest edges, 1700–2500 m. Voucher: Agnew ADQ (2013) (REF). Native distribution range: Eritrea to E DR Congo and E Tropical Africa.

***Cynanchumgonoloboides* Schltr.** – Habit: Woody climber. Habitat: Montane rain forest, 2400–3600 m. Vouchers: Wesche K 1256 (EA), Tweedie DR 2103 (BR), Rono et al. SAJIT-PR 0234 (EA, HIB). Native distribution range: S Ethiopia to Rwanda and N Tanzania.

***Cynanchumviminale* (L.) L.** – Habit: Succulent twiner. Habitat: Wet savanna, outcrop rock, 1750–2400 m. Voucher: Tweedie EM (1976) (REF). Native distribution range: Tropical & S Africa, W Indian Ocean, Tropical Asia to Australia

***Gomphocarpusfruticosus* (L.) W.T.Aiton** – Habit: Shrub. Habitat: Upland grassland, 900–2900 m. Voucher: Rono et al. SAJIT-PR 0124 (EA, HIB). Native distribution range: Eritrea to Africa, Arabian Peninsula.

***Gomphocarpussemilunatus* A.Rich.** – Habit: Shrubby herb. Habitat: Disturbed places, flooded grassland, roadside, 1500–2650 m. Vouchers: Tweedie EM (1976) (REF), Agnew ADQ (2013) (REF). Native distribution range: Nigeria to Ethiopia and Zambia.

***Gomphocarpusstenophyllus* Oliv.** – Habit: Shrubby herb. Habitat: Grassland, disturbed or rocky soils, 1600–3050 m. Voucher: Rono et al. SAJIT-PR 0244 (EA, HIB). Native distribution range: S Ethiopia to Tanzania

*Huerniakeniensis R.E.Fr. – Habit: Herb. Habitat: Outcrop rock, 1800–2400 m. Vouchers: Tweedie DR 299 (K), Tweedie EM 236 (K), Jex-Blake, M. 8417 (K). Native distribution range: Kenya to Tanzania.

***Landolphiabuchananii* Stapf** – Habit: Woody climbing shrub. Habitat: Forest near water, woodland, 1400–2400 m. Voucher: Tweedie DR 3253 (BR). Native distribution range: Tropical Africa.

***Margarettarosea* Oliv.** – Habit: Herb. Habitat: Wet savanna, burnt montane grassland, 1750–2250 m. Voucher: Tweedie EM (1976) (REF). Native distribution range: Nigeria to South Sudan and S Tropical Africa.

***Marsdeniaangolensis* N.E.Br.** – Habit: Herbaceous or woody scrambler. Habitat: Moist forest, bushland, 700–1900 m. Voucher: Tweedie 1886 (K). Native distribution range: Tropical Africa.

***Marsdeniaschimperi* Decne.** – Habit: Robust climber. Habitat: Secondary vegetation, upland forest edges, 1800–2650 m. Vouchers: Tweedie DR 736 (BR), Rono et al. SAJIT-PR 0103 (EA, HIB). Native distribution range: Nigeria to Angola and SW Arabian Peninsula.

***Mondiawhitei* Skeels** – Habit: Climbing shrub. Habitat: Moist upland forest edges, disturbed forest, 1600–2000 m. Voucher: Rono et al. SAJIT-PR 0193 (EA, HIB). Native distribution range: Tropical & S Africa.

***Orbeadummeri* (N.E.Br.) Bruyns** – Habit: Succulent herb. Habitat: Woodland, grassland, on rocky sandy soils, 1200–1650 m. Voucher: Tweedie EM 1114 (K). Native distribution range: E DR Congo to E Tropical Africa.

***Pachycarpusbisacculatus* (Oliv.) Goyder** – Habit: Herb. Habitat: Seasonally waterlogged grassland, deciduous woodland, 0–2300 m. Voucher: Tweedie EM 1198 (K). Native distribution range: Tropical Africa.

***Pachycarpuseximius* (Schltr.) Bullock** – Habit: Herb. Habitat: Grassland, 1600–2500 m. Vouchers: Tweedie EM (1976) (REF), Agnew ADQ (2013) (REF). Native distribution range: South Sudan to Burundi and Tanzania.

***Pachycarpusgrantii* (Oliv.) Bullock** – Habit: Herb. Habitat: Wooded grassland, 1000–2000 m. Vouchers: E & C Lugard 570 (EA), Irwin PH 278 (EA). Native distribution range: S Sudan to NW Tanzania.

***Pachycarpuslineolatus* (Decne.) Bullock** – Habit: Herb. Habitat: Wooded grasslands, 1000–2700 m. Voucher: Agnew ADQ (2013) (REF). Native distribution range: Tropical Africa to N Namibia.

***Pergulariadaemia* (Forssk.) Chiov.** – Habit: Herbaceous twiner. Habitat: Woodland, 1750–2400 m. Voucher: Tweedie EM (1976) (REF). Native distribution range: Tropical & S Africa, Sinai to Arabian Peninsula, Iran to W Indo-China.

***Periplocalinearifolia* Quart.-Dill. & A.Rich.** – Habit: Woody climber. Habitat: Upland forest edges, 1900–2900 m. Voucher: Tweedie EM 735 (BR). Native distribution range: Ethiopia to N Zambia.

***Raphionacmemadiensis* S.Moore** – Habit: Herb. Habitat: Wooded grassland on outcrop rock, 1800–2400 m. Voucher: Tweedie 1119 (EA). Native distribution range: Uganda to S Tropical Africa.

***Rauvolfiacaffra* Sond.** – Habit: Shrub or tree. Habitat: Woodland, forest, 1800–3000 m. Voucher: Tweedie EM (1976) (REF). Native distribution range: Tropical & S Africa.

***Sabacomorensis* (Bojer) Pichon** – Habit: Scrambling or climbing bush. Habitat: Moist and riverine forest, 1750–2000 m. Voucher: Agnew ADQ (2013) (REF). Native distribution range: Tropical Africa, Comoros, Madagascar.

***Secamonepunctulata* Decne.** – Habit: Climber or small shrub. Habitat: Thickets and riverine forest, 1500–2400 m. Voucher: Andersen R 139 (S). Native distribution range: Dry Tropical Africa to NW Namibia.

***Stathmostelmarhacodes* K. Schum.** – Habit: Herb. Habitat: Seasonally wet and waterlogged grassland, 1500–2650 m. Vouchers: Tweedie EM (1976) (REF), Agnew ADQ (2013) (REF). Native distribution range: South Sudan to NW Tanzania.

***Tabernaemontanastapfiana* Britten** – Habit: Tree. Habitat: Moist Forest, 1400–2300 m. Vouchers: Snowden JD 860 (BR, EA), Rono et al. SAJIT-PR 0189 (EA, HIB). Native distribution range: Uganda to S Tropical Africa.

***Vincetoxicumcaffrum* Kuntze** – Habit: Herb. Habitat: Seasonally burnt grassland, woodland, rocky slopes, 1750–2400 m. Voucher: Lugard 618 (EA). Native distribution range: Tropical & S Africa.

***Vincetoxicumlugardiae* (Bullock) Meve & Liede** – Habit: Herbaceous twinning vine. Habitat: Wet savanna, 1750–2250 m. Voucher: Lugard C 656 (K). Native distribution range: S Ethiopia to Kenya.

***Xysmalobiumundulatum* (L.) W.T.Aiton** – Habit: Herb. Habitat: Montane grassland, water logged depressions, roadsides, 1750–2400 m. Voucher: Tweedie EM (1976) (REF). Native distribution range: Ethiopia to S Africa.

#### F41. AQUIFOLIACEAE

1 Genus, 1 species

***Ilexmitis* (L.) Radlk.** – Habit: Tree. Habitat: Woodland, forest, upper forest edge, 1750–3150 m. Vouchers: Tweedie DR 1521 (BR), Battiscombe 643, Dale IR 57 (EA), Ceae 10 (EA), Gardner HM 2250 (EA). Native distribution range: Tropical & S Africa, Madagascar.

#### F42. ARALIACEAE

4 Genera, 9 species

***Astropanaxabyssinicus* Seem.** – Habit: Tree. Habitat: Upland rain forest, 1750–2770 m. Vouchers: St Clair-Thompson in Eggeling 3952 (EA), Dale IR 3097 (BR). Native distribution range: SE Nigeria and Ethiopia to Zambia.

***Astropanaxvolkensii* (Harms) Lowry, G.M.Plunkett, Gostel & Frodin** – Habit: Scandent shrub or tall tree. Habitat: Upland rainforest, 1600–3000 m. Vouchers: Dummer RA 3605 (EA), St Clair-Thompson in Eggeling 3958 (EA), Snowden JD 805 (EA, P), Hedberg 162, Taylor G 3414 (BR). Native distribution range: Ethiopia to E Tropical Africa.

***Cussoniaarborea* Hochst. ex A.Rich.** – Habit: Tree. Habitat: Wooded grassland, forest, 300–2470 m. Voucher: Snowden JD 850 (EA). Native distribution range: Tropical Africa.

***Cussoniaspicata* Thunb.** – Habit: Tree. Habitat: Upland rain forest, woodland, 1750–2550 m. Vouchers: Snowden JD 937 (EA, BR), Eggeling WJ 2492 (BR). Native distribution range: South Sudan to S Africa, Comoros.

***Hydrocotylemannii* Hook.f.** – Habit: Herb. Habitat: Damp Forest floor, bamboo zone, 650–3000 m. Vouchers: Katende T; Sheil D 611 (K), van Heist, M. 578 (K). Native distribution range: Tropical Africa, Madagascar.

**^EX^*Hydrocotyleranunculoides* L.f.** – Habit: Herb. Habitat: Muddy streams, ponds and marshes, 700–2400 m. Voucher: Agnew ADQ (2013) (REF). Native distribution range: America.

***Hydrocotylesibthorpioides* Lam.** – Habit: Herb. Habitat: Muddy and peaty stream banks, dump grasslands, upper forest zone and alpine, 1130–4000 m. Vouchers: Rose 10.177 (EA), Agnew ADQ & Agnew S 7277 (EA). Native distribution range: Tropical & Subtropical Old World.

***Polysciasfulva* (Hiern) Harms** – Habit: Tree. Habitat: Upland forest, riverine forest, grassland, 2250–3000 m. Voucher: St Clair-Thompson in Eggeling 3953 (EA). Native distribution range: Tropical Africa, SW Arabian Peninsula.

*Polysciaskikuyuensis Summerh. – Habit: Tree. Habitat: Upland rain forest, 2250–3000 m. Voucher: Tweedie EM (1976) (REF). Native distribution range: Kenya.

#### F43. ASTERACEAE

68 Genera, 191 species

^**EX**^***Achilleamillefolium* L.** – Habit: Herb. Habitat: Weed or in cultivation, forest, woodland, roadsides, 1950 m. Voucher: Jack C 163 (EA). Native distribution range: Subarctic & Temperate Northern Hemisphere to Guatemala

**^EX^*Acmellacaulirhiza* Delile** – Habit: Herb. Habitat: Woodland, forest, Riverside grassland, 1750–3100 m. Vouchers: Mwangangi OM 475 (EA), Hedberg O 30 (S), Granvik H 78 (S). Native distribution range: Tropical & S Africa, W Indian Ocean.

**^EX^*Adenostemmacaffrum* DC.** – Habit: Herb. Habitat: Wet savanna, 1750–2300 m. Voucher: Lugard 574 (EA). Native distribution range: Nigeria to Ethiopia and S Africa, W Yemen.

***Adenostemmamauritianum* DC.** – Habit: Herb. Habitat: Forest, forest edge, stream banks, bamboo zone, 1300–3000 m. Vouchers: Tweedie 921 (EA), Hedberg O 274 (BOT). Native distribution range: Tropical Africa, W Indian Ocean.

^**EX**^***Ageratinaadenophora* (Spreng.) R.M.King & H.Rob.** – Habit: Herb or shrub. Habitat: Montane forest and forest margins, bamboo zone, 1960–2600 m. Vouchers: Tweedie 2679 (EA), Lavranos JJ;Newton I 17773 (WAG, US), Lye & Pocs 23135 (EA), Rono et al. SAJIT-PR 0211 (EA, HIB). Native distribution range: Mexico.

***Ambassahochstetteri* Steetz** – Habit: Shrub, herb or small tree. Habitat: Bushland, forest, forest edges, 1000–2400 m. Vouchers: Tweedie 705 (EA), Granvik H (S), Hedberg O 175 (UPS). Native distribution range: Congo to Ethiopia and Angola.

***Anisopappuschinensis* (L.) Hook. & Arn.** – Habit: Herb. Habitat: Upland grassland, dump marshy sites, 1500–2500 m. Vouchers: Irwin PH hb (MNHN), Irwin PH 49 (S). Native distribution range: Sikkim to S China and Indo-China, Philippines.

**Anisopappuschinensissubsp.africanus (Hook.f.) S.Ortiz & Paiva** – Habit: Herb. Habitat: Wet savanna, 1750–2320 m. Voucher: Tweedie 825 (S). Native distribution range: Tropical Africa, Madagascar.

***Anthemistigreensis* J.Gay ex A.Rich.** – Habit: Herb. Habitat: Montane and Afroalpine grasslands, woodland, forest, moorland, 2500–4300 m. Vouchers: Thomas AS 598 (EA), Gillett JB 18464 (BR), Taylor G 3496 (S), Rono et al. SAJIT-PR 0146 (EA, HIB). Native distribution range: NE & E Tropical Africa.

**^EX^*Artemisiaafra* Jacq.** – Habit: Shrub. Habitat: Montane grassland, woodland, forest, moorland, 2000–4000 m. Vouchers: Kokwaro JO 2482 (EA), Major C & Lugard EJ 77 (EA), Tweedie D.R 126 (EA), Bickford N 39 (EA). Native distribution range: Ethiopia to S Africa.

***Aspiliakotschyi* (Sch.Bip. ex Hochst.) Oliv.** – Habit: Herb. Habitat: Wet savanna, 010–1900 m. Vouchers: Tweedie EM (1976) (REF), Agnew ADQ (2013) (REF). Native distribution range: Tropical & South Africa, W Yemen.

***Aspiliamossambicensis* (Oliv.) Wild** – Habit: Herb or shrub. Habitat: Ruderal sites, seasonal swamps, woodland, wooded grassland, 005–2400 m. Vouchers: Tweedie EM (1976) (REF), Agnew ADQ (2013) (REF). Native distribution range: Eritrea to S Africa, W Yemen.

***Aspiliapluriseta* Schweinf. ex Engl.** – Habit: Herb or shrub. Habitat: Grassland, woodland, 1050–2600 m. Vouchers: Tweedie EM (1976) (REF), Agnew ADQ (2013) (REF). Native distribution range: South Sudan to S Africa.

***Athrixiarosmarinifolia* Oliv. & Hiern** – Habit: Shrub. Habitat: Montane or alpine grassland, forest clearing, 2600–3500 m. Voucher: Hedberg 4507 (EA). Native distribution range: Ethiopia to S Tropical Africa.

***Baccharoidesadoensis* (Sch.Bip. ex Walp.) H.Rob.** – Habit: Woody herb or subshrub. Habitat: Grassland and wooded grassland, deciduous woodland, riverine and old cultivation, 800–2750 m. Voucher: Symes 239 (EA). Native distribution range: Tropical Africa.

**^EX^Baccharoidescalvoanasubsp.leucocalyx (O.Hoffm.) Isawumi, El-Ghazaly & B.Nord.** – Habit: Shrub, woody herb, rarely a tree. Habitat: Riverine, forest margin, bamboo forest margins, upland bushland, savanna, 1500–3000 m. Voucher: Tothill BH 2283 (EA). Native distribution range: Tanzania.

***Baccharoidesdumicola* (S.Moore) Isawumi, El-Ghazaly & B.Nord.** – Habit: Herb or small shrub. Habitat: Wet grassland, swamps, 1100–2300 m. Vouchers: Tweedie 3677 (EA), Gilbert & Mesfin 6561 (EA). Native distribution range: South Sudan to E Central & E Tropical Africa.

***Baccharoideshymenolepis* (A.Rich.) Isawumi, El-Ghazaly & B.Nord.** – Habit: Woody herb, shrub or tree. Habitat: forest and bamboo margins, upland bushland, savanna, 150–2950 m. Voucher: Rono et al. SAJIT-PR 0215 (EA, HIB). Native distribution range: Ethiopia to Kenya.

***Baccharoideslasiopus* (O.Hoffm.) H.Rob.** – Habit: Shrub or woody herb. Habitat: Forest clearings, margins, riverine thickets, secondary grasslands, old cultivations, 1100–2800 m. Voucher: Agnew ADQ (2013) (REF). Native distribution range: Ethiopia to Mozambique.

***Baccharoidespumila* (Kotschy & Peyr.) Isawumi** – Habit: Herb. Habitat: Burnt wooded grassland, wet grassland, 1750–1850 m. Voucher: Tweedie 124 (EA). Native distribution range: W Tropical Africa to South Sudan.

***Berkheyaspekeana* Oliv.** – Habit: Herb. Habitat: Wooded grassland, 1800–3810 m. Vouchers: Lewis WH 5968 (US), Hedberg O (S), Holm Å 63 (S), Gerh. Lindblom sn (S). Native distribution range: Nigeria to Ethiopia and Tanzania.

***Bidensbiternata* (Lour.) Merr. & Sherff** – Habit: Herb. Habitat: Woodland, forest margin, weed of cultivation, ruderal sites, 1750–2500 m. Vouchers: Tweedie EM (1976) (REF), Agnew ADQ (2013) (REF). Native distribution range: Tropical & Subtropical Old World.

***Bidensbuchneri* Sherff** – Habit: Herb or shrub. Habitat: Wooded grassland among rocks, 1450–2500 m. Vouchers: symes 219 (EA), Gerh. Lindblom (S). Native distribution range: South Sudan to Angola.

*Bidenselgonensis (Sherff) Agnew – Habit: Herb or shrub. Habitat: Grassland and Erica bushland, 2500–4000 m. Vouchers: Wesche K 1680, 858 (EA), Lind EM 2099 (EA), Smith SA, Beentje HJ & Muasya A 163 (US), Taylor G 3586 (S), Dummer RA 3304 (S), Thomas AS 2689 (EA). Native distribution range: Uganda to Kenya.

^**EX**^***Bidensflagellata* (Sherff) Mesfin** – Habit: Herb. Habitat: Grassland, 1600–3300 m. Voucher: Rono et al. SAJIT-PR 0116 (EA, HIB). Native distribution range: Ethiopia to Burundi.

***Bidensgrantii* Sherff** – Habit: Herb. Habitat: Grassland, ruderal sites, 1100–2700 m. Voucher: Agnew ADQ (2013) (REF). Native distribution range: Central & E Tropical Africa.

**^EX^*Bidenspilosa* L.** – Habit: Herb. Habitat: wet savanna, woodland, 400–2500 m. Vouchers: Lugard EJ 95 (EA), Hedberg O 17 (S), Holm Å 75 (S). Native distribution range: Tropical & Subtropical America.

***Bidensternata* (Chiov.) Sherff** – Habit: Herb. Habitat: Wooded grassland, bushland, forest clearing, 1500–2700 m. Vouchers: Tweedie 394 (EA, MO). Native distribution range: Cameroon to Ethiopia and Tanzania.

**Bothrioclineauriculata (M.Taylor) C.Jeffrey – Habit: Herb or shrub. Habitat: Bamboo thickets, montane forest, 2700–3150 m. Vouchers: Dummer RA 3563 (EA, MO), Wesche K 164, 1252 (MO), Kisalye 270 (MO), Liebenberg 1584 (EA)), Tothill BH 2330 (EA). Native distribution range: Uganda.

***Bothrioclinefusca* (S.Moore) M.G.Gilbert** – Habit: Shrub or shrubby herb. Habitat: Roadside and disturbed places, 2000–3600 m. Vouchers: Kahuho SK 9 (K), Major EJ; Lugard C 370 (K), Smith SAL; Beentje HJ; Muasya AM 162 (K), Hedberg O 88 (K), Taylor G 3443 (S), Tweedie 2552 (K), Rono et al. SAJIT-PR 0267 (EA, HIB). Native distribution range: E Tropical Africa to Zambia.

***Bothrioclinelongipes* N.E.Br.** – Habit: Herb or shrub. Habitat: Forest margin and clearing, Upland grassland, 1500–2800 m. Vouchers: Dawkins H 784 (K), Holm Å 71 (S), Granvik H 24 (S). Native distribution range: Gabon to Kenya and S Tropical Africa.

*Bothrioclinemonticola (M.Taylor) Wech. – Habit: Shrub. Habitat: Rare in margins of montane forest, 2550–3000 m. Voucher: Agnew ADQ (2013) (REF). Native distribution range: South Sudan to Kenya.

***Carduusafromontanus* R.E.Fr.** – Habit: Herb. Habitat: Forest clearings, bamboo zones, 2350–3430 m. Voucher: Dummer RA 3509 (EA). Native distribution range: Kenya to Uganda.

*Carduuskeniensis R.E.Fr. – Habit: Herb. Habitat: Moorland, heath zone, 2950–4300 m. Vouchers: Muasya J & Berit Gehrke 144 (EA), Hamilton & Perrott 76/569 (EA), Tweedie R 39 (EA), 107 (EA), Liebenberg 1610 (EA), Dummer RA 3349 (EA). Native distribution range: E Tropical Africa.

***Carduusleptacanthus* Fresen.** – Habit: Herb. Habitat: Wooded grassland, forest glades, margins, 1550–2450 m. Vouchers: Lugard 191 (EA). Native distribution range: Ethiopia to E Central & E Tropical Africa.

***Carduusnyassanus* R.E.Fr.** – Habit: Herb. Habitat: Woodland, montane forest, wet disturbed ground, 1750–3350 m. Vouchers: Jack 343 (EA), Lewis WH 5972 (US). Native distribution range: Nigeria to Ethiopia and S Tropical Africa.

**Carduusnyassanussubsp.kikuyorum (R.E.Fr.) C.Jeffrey** – Habit: Herb. Habitat: Upland grassland, moorland, forest glades, bamboo margins, 1500–3450 m. Voucher: Tothill BH 2368 (EA). Native distribution range: E Central & E Tropical Africa.

***Carduusschimperi* Sch.Bip.** – Habit: Herb. Habitat: Grassland, alpine and subalpine zone, 1800–4100 m. Vouchers: Tweedie DR 103 (EA), Lisowski S 84147 (BR), Rono et al. SAJIT-PR 0232 (EA, HIB). Native distribution range: Ethiopia to Tanzania.

**Carduusschimperisubsp.nanus (R.E.Fr.) C.Jeffrey** – Habit: Herb. Habitat: Grassland, moorland, 2250–4050 m. Vouchers: Hedberg O 136 (S, EA), Dummer RA 3311 (EA), Liebenberg 1602 (EA), Tothill BH 2328 (EA). Native distribution range: E Central & E Tropical Africa.

***Centaureapraecox* Oliv. & Hiern** – Habit: Herb. Habitat: Wet savanna, grassland, wooded grassland, 1750–2600 m. Voucher: Tweedie EM (1976) (REF). Native distribution range: Tropical Africa.

**^EX^*Chromolaenacorymbosa* (Aubl.) R.M.King & H.Rob.** – Habit: Herb. Habitat: Disturbed grounds, wet savanna, 30–2500 m. Vouchers: Tweedie EM (1976) (REF), Agnew ADQ (2013) (REF). Native distribution range: Caribbean to French Guiana.

**^EX^*Chrysanthellumamericanum* (L.) Vatke** – Habit: Herb. Habitat: Wet savanna, 1750–2250 m. Voucher: Tweedie EM (1976) (REF). Native distribution range: Mexico (Chiapas, Michoacán) to Central America, Caribbean.

^**EX**^***Cinerariadeltoidea* Sond.** – Habit: Herb. Habitat: Woodland, grassland, forest edge, moorland, 1750–4300 m. Vouchers: Taylor G 3519, (S, EA), 3440 (S), Adamson J 478, 498 (EA), Hedberg O 118 (S) 4484 (EA), 891, Ekkens DB 405 (EA), Morris 268 (EA), Synge PM S 1916, 1085 (S), Granvik H 163 (S), Tothill BH J T2427 (US), Rono et al. SAJIT-PR 0243 (EA, HIB). Native distribution range: Ethiopia to S Africa.

***Cirsiumbuchwaldii* O.Hoffm.** – Habit: Herb. Habitat: Swampy grassland, 1835–3050 m. Vouchers: Taylor G 3588 (EA, S). Native distribution range: South Sudan to Zambia.

^**EX**^***Cirsiumvulgare* (Savi) Ten.** – Habit: Herb. Habitat: In cultivation, 1790–2400 m. Voucher: Rono et al. SAJIT-PR 0168 (EA, HIB). Native distribution range: Europe to Siberia and Arabian Peninsula, NW Africa.

***Conyzaclarenceana* Oliv. & Hiern** – Habit: Herb. Habitat: Swamps, along streams, moorland subalpine marshes, 2300–3300 m. Voucher: Agnew ADQ (2013) (REF). Native distribution range: Cameroon, Ethiopia, Gulf of Guinea Island, Kenya, Uganda, Zambia.

***Conyzanewii* Oliv. & Hiern** – Habit: Shrub or woody herb. Habitat: Forest, montane scrub woodland, 1750–3050 m. Voucher: Dummer RA 3608 (EA). Native distribution range: Ethiopia, Kenya, Rwanda, Sudan, Tanzania, Uganda, Zambia, Zaïre

***Conyzapallidiflora* R.E.Fr.** – Habit: Woody herb. Habitat: Disturbed areas of forest, woodland, 2000–3000 m. Vouchers: Major EJ & Lugard C 401 (EA), Rono et al. SAJIT-PR 0262 (EA, HIB), Cheseny CMC 46 (EA). Native distribution range: Kenya.

***Cotulaabyssinica* Sch.Bip. ex A.Rich.** – Habit: Herb. Habitat: Short alpine grassland, forest margin, heath zones, bamboo zone, 2100–3650 m. Vouchers: Dummer RA 3579 (EA), Thomas AS 536, 2704 (EA), Lye KA 5715 (EA), Beentje HJ 1944 (EA, WAG), Hedberg O 945 (S). Native distribution range: Eritrea to E Central & E Tropical Africa.

**^EX^*Cotulaaustralis* Hook.f.** – Habit: Herb. Habitat: moist cultivation, 1200–2300 m. Voucher: Agnew ADQ (2013) (REF). Native distribution range: Zimbabwe to S Africa, Madagascar, Australasia.

*Cotulacryptocephala Sch.Bip. ex A.Rich. – Habit: Herb. Habitat: Rare along paths in alpine grassland, 3140–3800 m. Vouchers: Hedberg 996 (S), Hedberg O 4515 (BR, EA), Klotzli F et al. 1953 (EA). Native distribution range: Ethiopia to Kenya.

^**EX**^***Crassocephalumcrepidioides* (Benth.) S.Moore** – Habit: Herb. Habitat: Grassland, forest margins, secondary vegetation, 1700–2500 m. Voucher: Holm Å 70 (S). Native distribution range: Tropical & S Africa, Madagascar.

***Crassocephalummontuosum* (S.Moore) Milne-Redh.** – Habit: Herb or wooded shrub. Habitat: Clearing of montane forest, disturbed forest, bamboo zone, 1500–3260 m. Vouchers: Morrison 264 (EA), Dawkins HC 785 (K, EA), Mwangangi OM 486 (BR, EA), 395 (EA), Jack C 156 (EA), Granvik H 329 (S). Native distribution range: Tropical Africa, Madagascar.

***Crassocephalumpaludum* C.Jeffrey** – Habit: Herb. Habitat: Swamps, grassland, 2250–3000 m. Voucher: Tweedie 3646 (EA). Native distribution range: South Sudan to Zambia.

**^EX^*Crassocephalumpicridifolium* S.Moore** – Habit: Herb. Habitat: Swampy grasslands, wet savanna, woodland, 1750–2700 m. Vouchers: Symes YE 345 (EA), Major C & Lugard EJ 190 (EA), Jack C 2 (EA), Wiltshire FM 19 (K), Tweedie 3577 (US), Native distribution range: Tropical & S Africa.

***Crassocephalumrubens* (Jacq.) S.Moore** – Habit: Herb. Habitat: Disturbed cultivated areas, grassland, 1750–2500 m. Voucher: Lugard 439 (EA). Native distribution range: Africa, Arabian Peninsula.

**Crassocephalumrubensvar.sarcobasis (DC.) C.Jeffrey & Beentje** – Habit: Herb. Habitat: disturbed cultivated areas, grassland, 1750–2400 m. Vouchers: Tweedie EM (1976) (REF), Agnew ADQ (2013) (REF). Native distribution range: Tropical Africa to South Africa, Comoros, Madagascar, SW Arabian Peninsula.

***Crassocephalumvitellinum* S.Moore** – Habit: Herb. Habitat: Grassland clearing, forest, woodland, bushlands, 1500–2800 m. Voucher: Hedberg O 313 (S). Native distribution range: Nigeria to South Sudan and Zambia.

***Crepiscarbonaria* Sch.Bip.** – Habit: Herb. Habitat: heath zones, short montane grassland, 1350–4000 m. Vouchers: Morris MES 272 (EA), Battiscombe E 674 (EA), Hedberg O 848 (EA), Wesche K 1348 (K), Smith SAL; Beentje HJ; Muasya AM 166 (K), Tweedie 26 (K), Irwin PH 14 (K), Tweedie 2549 (K). Native distribution range: Ethiopia to E Central & E Tropical Africa.

***Crepisdianthoseris* N.Kilian, Enke, Sileshi & Gemeinholzer** – Habit: Herb. Habitat: Alpine zone by streamside, 3600–4300 m. Vouchers: Wesche K 434 (EA), Rono et al. SAJIT-PR 0239 (EA, HIB). Native distribution range: Ethiopia to Tanzania.

***Crepisrueppellii* Sch.Bip.** – Habit: Herb. Habitat: Grassland, 1600–3200 m. Vouchers: Tweedie 729. Native distribution range: NE & E Tropical Africa, Arabian Peninsula.

***Crepisschultzii* Hochst. ex Oliv.** – Habit: Herb. Habitat: Clearing in bamboo zone. Voucher: Tothill BH 2325 (EA). Native distribution range: Ethiopia to Uganda.

**Dendrosenecioelgonensissubsp.barbatipes (Hedberg) E.B.Knox – Habit: Tree or shrub. Habitat: Upper afro alpine zone, 3750–4225 m. Vouchers: Gardener 2269 (K), Hedberg O 872 (K), Thomas AS 627 (K), Ekkens 622 (EA), Fairbairn G 3159 (BR), Synge P M 922 (BR), Dale A2/41 (EA), Eggeling 5753 (EA). Native distribution range: Uganda to Kenya (Mt Elgon).

**Dendrosenecioelgonensis(T.C.E.Fr.)E.B.Knoxsubsp.elgonensis – Habit: Tree or shrub. Habitat: Upper Afromontane Forest, 2750–4200 m. Vouchers: Tweedie 370 (K), Tweedie DR 2 (K), Lugard & Lugard 699 (K), Osmaston HA 4017 (K), Porter 2732, Synge 888 (EA), Dale K 3211 (EA), Thomas AS 628 (EA). Native distribution range: Uganda to Kenya (Mt Elgon).

**^EX^*Dendroseneciojohnstonii* (H.H.Johnst.) B.Nord.** – Habit: Tree or shrub. Habitat: Upper afro alpine zone, 2500–4000 m. Voucher: Fairbairn G 3159 (US). Native distribution range: Tanzania (Mt Kilimanjaro).

***Dichrocephalachrysanthemifolia* (Blume) DC.** – Habit: Herb. Habitat: Woodland, forest and upper wet montane forest, 2200–3724 m. Vouchers: Morrison MES 270 (EA), Major EJ & Lugard C 241 (EA), Hedberg O 947 (S), Taylor G 3600 (S), Lisowski S 58054 (BR), Rwaburundindore 444 (EA). Native distribution range: Tropical & Subtropical Old World.

**Dichrocephalachrysanthemifoliavar.alpina (R.E.Fr.) Beentje** – Habit: Herb. Habitat: Alpine zone, 2600–3600 m. Vouchers: Tweedie EM (1976) (REF), Agnew ADQ (2013) (REF). Native distribution range: Ethiopia to E DR Congo and Tanzania.

***Dichrocephalaintegrifolia* (L.f.) Kuntze** – Habit: Herb. Habitat: Disturbed wet montane forest, cultivation, grassland, bamboo zone, 1700–3670 m. Vouchers: Jack C 199 (EA), Agnew ADQ & Agnew S 7266 (EA), Holm Å 56 (S). Native distribution range: Turkey to Asia and Pacific.

***Echinopsaberdaricus* R.E.Fr.** – Habit: Herb. Habitat: Montane grassland, moorland, forest margin, clearing, 2400–3600 m. Voucher: Human observation sn (EA). Native distribution range: Kenya to Tanzania.

***Echinopsamplexicaulis* Oliv.** – Habit: Herb. Habitat: Wooded grasslands, edges of cultivated lands, 1300–2700 m. Vouchers: Lugard C & Lugard EJ 82 (EA). Native distribution range: Nigeria to Ethiopia and Tanzania.

***Echinopsangustilobus* S.Moore** – Habit: Herb. Habitat: Grassland, forest edges, 2000–2850 m. Vouchers: Tweedie EM (1976) (REF), Agnew ADQ (2013) (REF). Native distribution range: Ethiopia to Kenya.

***Echinopseryngiifolius* O.Hoffm.** – Habit: Herb. Habitat: Grassland, woodland, 1050–1850 m. Vouchers: Lugard & Lugard 553. Native distribution range: E Central & E Tropical Africa.

***Echinopshispidus* Fresen.** – Habit: Herb. Habitat: Upland grassland, scattered tree grassland, 1500–2300 m. Vouchers: Major C & Lugard EJ 158 (EA), Jack C 83 (EA). Native distribution range: Eritrea to Tanzania.

***Echinopshoehnelii* Schweinf.** – Habit: Herb. Habitat: Forest edges, woodland, heathland, 2200–3500 m. Vouchers: Adamson J 487 (EA), Lisowski S 55110 (BR), Hamilton & Perrott 76/400 (EA), Thomas 638 (EA). Native distribution range: Ethiopia to E Central & E Tropical Africa.

*Echinopslanatus C.Jeffrey & Mesfin – Habit: Herb. Habitat: Montane grasslands, stoebe bushlands, 2400–3600 m. Vouchers: Townsend CC 2334 (EA), Fries re (EA), Freidberg A 152 (EA). Native distribution range: Cameroon, E Tropical Africa.

***Emiliacoccinea* G.Don** – Habit: Herb. Habitat: Roadside, grassland, waste grounds, 1800–2400 m. Voucher: Webster 8860 (EA). Native distribution range: Tropical Africa.

***Emiliadebilis* S.Moore** – Habit: Herb. Habitat: Moist grassland, 1750–2750 m. Voucher: Tweedie EM (1976) (REF). Native distribution range: E Central & E Tropical Africa.

***Emiliadiscifolia* (Oliv.) C.Jeffrey** – Habit: Herb. Habitat: Disturbed forests, 500–2500 m. Voucher: Holm Å 59 (S). Native distribution range: Ethiopia to S Tropical Africa.

**^EX^*Emiliaintegrifolia* Baker** – Habit: Herb. Habitat: Disturbed swamp, moist grasslands, 1900–2800 m. Vouchers: Webster 8859 (EA), Lugard EJ 101 (EA). Native distribution range: DR Congo to S Tropical Africa, Madagascar.

**^EX^*Emiliasonchifolia* (L.) DC.** – Habit: Herb. Habitat: Wet savanna, 1750–2250 m. Voucher: Tweedie EM (1976) (REF). Native distribution range: Tropical & Subtropical Old World.

*Emiliatenera (O.Hoffm.) C.Jeffrey – Habit: Herb. Habitat: Grassland, swampy grassland, 1800–2700 m. Voucher: Lind EM 269 (EA). Native distribution range: E Tropical Africa.

***Emiliatricholepis* C.Jeffrey** – Habit: Herb. Habitat: Rock crevices, cultivated lands, 700–2000 m. Voucher: Wesche K 366 (K). Native distribution range: Kenya.

**^EX^*Erigeronbonariensis* L.** – Habit: Herb. Habitat: Grassland, roadside, 400–2600 m. Voucher: Holm Å 73 (S). Native distribution range: Mexico to Tropical America.

***Erigeronschimperi* Sch.Bip. ex Schweinf.** – Habit: Woody herb or small shrub. Habitat: Grassland, impeded drainage, woodland and upper forest edge, 2000–3000 m. Vouchers: Tweedie 1412 (EA), Hedberg O 1061 (UPS), J Wilson 1237 (EA). Native distribution range: Ethiopia to E Central & E Tropical Africa, Yemen.

***Eschenbachiagouanii* (L.) G.L.Nesom** – Habit: Herb. Habitat: Disturbed grassland, 1500–3600 m. Vouchers: Hedberg O 911 (S), Rono et al. SAJIT-PR 0180 (EA, HIB). Native distribution range: Africa, Arabian Peninsula.

**^EX^*Eschenbachiastricta* (Willd.) Raizada** – Habit: Herb. Habitat: Disturbed grassland, 1500–2000 m. Vouchers: Waston J 1195 (EA), Tweedie 2394 (EA), Lugard C & Major EJ 31 (EA). Native distribution range: Sahara & Sahel to S Jordan and Arabian Peninsula, Himalaya to China (Sichuan, Yunnan) and N Indo-China.

***Eschenbachiasubscaposa* (O.Hoffm.) G.L.Nesom** – Habit: Herb. Habitat: Woodland, alpine and subalpine grassland, 1750–4000 m. Vouchers: Marrison MES 271 (EA), Tweedie 53 (EA), Major EJ & Lugard J 616 (EA), Adamson J 501 (EA), Hedberg O 122, 1026 (S), Lisowski S 57185 (BR), Rono et al. SAJIT-PR 0240 (EA, HIB). Native distribution range: Nigeria to Kenya and S Tropical Africa.

**^EX^*Ethuliaconyzoides* L.** – Habit: Herb. Habitat: Swampy grassland, 1160–1670 m. Voucher: Agnew ADQ (2013) (REF). Native distribution range: Africa to Sinai, Indian Subcontinent to China (Yunnan) and Peninsula Malaysia, Taiwan to Philippines.

***Ethuliavernonioides* (Schweinf.) M.G.Gilbert** – Habit: Herb. Habitat: Grasslands, 1650–3000 m. Voucher: Agnew ADQ (2013) (REF). Native distribution range: Kenya to Tanzania.

**Euryopselgonensis Mattf. – Habit: Shrub. Habitat: Heath zone, bamboo zone, moorland, 300–4200 m. Vouchers: Lugard C & Lugard EJ 368 (K, EA), Tweedie R 18 (EA), 76 (EA), Hedberg 863 (K, EA), Hedberg O 4525 (EA), Ekkens DB 407 (EA), Dummer RA 3386 (K), Lisowski S 53797 (BR), Kokwaro JO 2459 (EA), Freidberg A 145 (EA), Holm Å 77 (S), Taylor G 3529 (S), Granvik H 155 (S), Tothill BH 269 (EA), Thomas AS 2738 (EA). Native distribution range: Uganda.

^**EX**^***Galinsogaparviflora* Cav.** – Habit: Herb. Habitat: Moist grassland, woodland, forest margin, 2300–3000 m. Vouchers: Major C & Lugard EJ 107 (EA), Dawkins, H.C. 778 (K), Hedberg O 8 (S), Holm Å 58 (S), Granvik H 294 (S). Native distribution range: Mexico to Tropical America.

^**EX**^***Galinsogaquadriradiata* Ruiz & Pav.** – Habit: Herb. Habitat: waste places, roadsides, 1800–2600 m. Voucher: Beentje HJ 1943 (EA). Native distribution range: Mexico to S Tropical America.

**^EX^*Gamochaetapurpurea* (L.) Cabrera** – Habit: Herb. Habitat: Upland grassland, waste places, roadside, 1750–2400 m. Voucher: Tweedie 2244 (EA). Native distribution range: E Canada to Tropical America.

***Gerberaviridifolia* (DC.) Sch.Bip.** – Habit: Herb. Habitat: Wooded grassland, grassland, woodland, 1600–2750 m. Vouchers: Lewis WH 5969 (S), Major C & Lugard EJ 515 (EA), Podwa SH 24 (BR, EA), Beentje HJ 1930 (EA). Native distribution range: Cameroon to Eritrea and S Africa.

^***EX***^***Gnaphaliumdeclinatum* L.f.** – Habit: Herb. Habitat: Woodland, forest, 1750–3150 m. Vouchers: Beentje HJ 1940 (WAG), Hedberg O 110 (S). Native distribution range: Zambia to S Africa.

***Gnaphaliumunionis* Sch.Bip. ex Oliv. & Hiern** – Habit: Herb. Habitat: Montane grassland, montane forest and heath zone, 1600–3800 m. Vouchers: Thomas AS 604 (EA), Forbes 264 (EA), Wood G 147 (EA). Native distribution range: Eritrea to Burundi.

**Gnaphaliumunionisvar.rubriflorum (Hilliard) Beentje** – Habit: Herb. Habitat: Wet montane grassland, montane forest, heath zone, shallow soils, 1600–3150 m. Vouchers: Mwangangi OM 473 (EA), Tweedie 1296 (K), Wilson J 1193 (EA), Rono et al. SAJIT-PR 0264 (EA, HIB). Native distribution range: Ethiopia to E Central & E Tropical Africa.

*Guizotiajacksonii (S.Moore) J.Baagoe – Habit: Herb. Habitat: Moist Forest glades, short grassland in bamboo, moorland, heath zones, 2350–3900 m. Voucher: Wesche K 1940 (EA). Native distribution range: Uganda to Kenya.

***Guizotiascabra* Chiov.** – Habit: Herb. Habitat: Grassland, forest clearing, 1300–3200 m. Vouchers: Dummer RA 3599 (US), Granvik H 260 (S), Synge PM S 847 (S), Holm Å 81 (S), Thomas AS 2701 (EA), Rono et al. SAJIT-PR 0203 (EA, HIB). Native distribution range: Tropical Africa, W Yemen.

**^EX^Gutenbergiacordifoliavar.marginata (O.Hoffm.) C.Jeffrey** – Habit: Herb. Habitat: Woodland, 1750–2400 m. Voucher: Tweedie EM (1976) (REF). Native distribution range: Tanzania to S Tropical Africa.

***Gutenbergiapetersii* Steetz** – Habit: Herb. Habitat: Outcrop rock, 1750–2400 m. Voucher: Tweedie EM (1976) (REF). Native distribution range: Kenya to Tanzania.

***Gutenbergiarueppellii* Sch.Bip.** – Habit: Herb. Habitat: Rocky eroded grassland, 900–2300 m. Vouchers: Gilbert & Mesfin 6546 (EA), Rono et al. SAJIT-PR 0028 (EA, HIB). Native distribution range: Eritrea to E Central & E Tropical Africa.

***Gymnanthemumamygdalinum* (Delile) Sch.Bip.** – Habit: Shrub or small tree. Habitat: Woodland, wooded grassland, secondary bushlands, forest margin, 1750–2400 m. Vouchers: Jackson 303 (EA), Freidberg A 27 (EA). Native distribution range: E Bolivia to Brazil, Tropical Africa, W Yemen.

***Gymnanthemumauriculiferum* (Hiern) Isawumi** – Habit: Shrub or small tree. Habitat: Disturbed bushland, montane forest margins, riverine forest, 1100–3000 m. Vouchers: Rono et al. SAJIT-PR 0154 (EA, HIB), Mwangangi OM 412 (BR), Hedberg O (UPS), Snowden JD 823 (MNHN). Native distribution range: Nigeria to Ethiopia and Angola.

***Gymnanthemumurticifolium* (A.Rich.) H.Rob.** – Habit: Shrub. Habitat: Secondary Forest, montane forest edges, 2160–3000 m. Vouchers: Tweedie EM (1976) (REF), Agnew ADQ (2013) (REF). Native distribution range: Ethiopia, Kenya, DR Congo.

***Gynuraamplexicaulis* Oliv. & Hiern** – Habit: Herb. Habitat: Disturbed places, swampy sites in grassland, weed in cultivations, 2000–3000 m. Voucher: Lugard 240 (EA). Native distribution range: Central African Republic to South Sudan and Tanzania.

***Gynurascandens* O.Hoffm.** – Habit: Herb. Habitat: mist forest clearing, about 2200 m. Voucher: Agnew ADQ (2013) (REF). Native distribution range: Cameroon to Kenya and S Tropical Africa.

***Haplocarpharueppelii* Beauverd** – Habit: Herb. Habitat: Montane grassland, moorland, upper bamboo zone, 2600–4300 m. Vouchers: Lienberg 1578 (EA), Wood 127 (EA), Gillet 18461 (EA), Rono et al. SAJIT-PR 0145 (EA, HIB). Native distribution range: Ethiopia to E Tropical Africa.

**Helichrysumamblyphyllum Mattf. – Habit: Shrub. Habitat: Alpine stony grassland, 3200–4200 m. Vouchers: Dummer RA 3319 (K, US), Hedberg O 4454 (UPS), Hedberg O 864 (UPS, S), Åke Strid 3549 (S), Gardner HM 2271 (NMNH), Synge PM 1904 (BR). Native distribution range: Uganda (Mt Elgon).

***Helichrysumargyranthum* O.Hoffm.** – Habit: Shrub. Habitat: Forest edges, streamside in heathland, 2100–3900 m. Vouchers: Dummer RA 3597 (MNHN), Lisowski S 57327 (BR), Lisowski S 57305 (BR), Lisowski S 57293 (BR), Thomas AS 2743 (BR), Synge PM 836 (BR), Synge PM 959 (BR), Ross R 1357 (BR), Mwangangi OM 501 (BR), Rono et al. SAJIT-PR 0242 (EA, HIB). Native distribution range: Ethiopia to E Central & E Tropical Africa.

*Helichrysumbrownei S.Moore – Habit: Shrublet or prostrate herb. Habitat: Moorland, alpine slopes, 3150–4321 m. Vouchers: Taylor G 3507 (BR), Arambourg C|Jeannel R|Chappuis PA hn 179 (MNHN). Native distribution range: Kenya (Mt Kenya).

***Helichrysumcitrispinum* Delile** – Habit: Shrub or shrubby herb. Habitat: Alpine grasslands, 2900–4321 m. Vouchers: Åke Strid 3560 (S), Hedberg O 262 (S), Hedberg O 865 (S), Dummer RA 3317(US), Lisowski S 57263 (BR), Lisowski S 57262 (BR). Native distribution range: Ethiopia.

**Helichrysumcitrispinumvar.hoehnelii (Schweinf.) Hedberg** – Habit: Shrub or shrubby herb. Habitat: Moorland, 3150–4321 m. Vouchers: Hedberg O 262 (K), (BR), Dale IR 3185 (K), Mabberley, D.J. 456 (K), Tweedie 1989 (K), Tweedie R 95 (K), Raumfoid C 502 (K), Synge PM S1902 (BR), Taylor G 3505 (BR). Native distribution range: Ethiopia to E Tropical Africa.

***Helichrysumellipticifolium* Moeser** – Habit: Shrub. Habitat: Montane grassland, swamps in bamboo clearing, 2500–3600 m. Vouchers: (EA). Native distribution range: E Central Tropical Africa, Kenya.

***Helichrysumfoetidum* Moench** – Habit: Herb. Habitat: Woodland, montane forest, 1750–3150 m. Vouchers: Gerh Lindblom (S), Rono et al. SAJIT-PR 0158 (EA, HIB). Native distribution range: Tropical & S Africa, Madagascar, Arabian Peninsula.

***Helichrysumformosissimum* Sch.Bip.** – Habit: Shrub. Habitat: Roadside, alpine zone, burnt grassland in moorland, 2300–4200 m. Vouchers: Dummer RA 3320 (K), Hedberg O 179 (S), Hedberg O 845 (S), Holm Å 65 (S), Thomas AS 2708 (MNHN), Taylor G 3612 (S), Arambourg C|Jeannel R|Chapuis PA 89, 178, 177 (P), Rono et al. SAJIT-PR 0253 (EA, HIB). Native distribution range: Ethiopia to E Central & E Tropical Africa.

***Helichrysumforskahlii* (J.F.Gmel.) Hilliard & B.L.Burtt** – Habit: Herb or shrub. Habitat: Alpine grassland, bushland, bamboo, forest and heath, 1750–4321 m. Vouchers: Tweedie 114 (EA), Kokwaro JO 2465 (EA), Hedberg O 1953 (EA), Dale IR 3212 (EA), Rono et al. SAJIT-PR 0252 (EA, HIB). Native distribution range: Tropical Africa, Comoros, SW Arabian Peninsula.

**Helichrysumforskahliivar.compactum (Vatke) Mesfin** – Habit: Herb. Habitat: Moorland grassland, tussock grassland, bushland, 2800–4300 m. Vouchers: Snowden JD 476 (EA), Dale 3212 (K), Knox EB 3757 (K), Kokwaro JO 2465 (EA), Tweedie DR 7 (EA), Major C & Lugard EJ 392 (EA), Smith SAL; Beentje HJ; Muasya AM 165 (K), 176 (K), Hedberg O 972 (K). Native distribution range: Ethiopia to Tanzania.

***Helichrysumglobosum* Sch.Bip.** – Habit: herb. Habitat: Upland grassland, riverine forest, bamboo glades, 1200–3100 m. Vouchers: Mwangangi OM 476 (EA), Tothill BH 2362 (EA), Rono et al. SAJIT-PR 0166 (EA, HIB). Native distribution range: Nigeria to Ethiopia and S Tropical Africa, Madagascar.

***Helichrysumkilimanjari* Oliv.** – Habit: Herb. Habitat: Disturbed burnt alpine zone, 2800–4000 m. Vouchers: Åke Strid 3428 (S), Hedberg O 890 (S), Granvik H 113 (S), Holm Å 66 (S). Native distribution range: Kenya to S Tropical Africa.

**^EX^*Helichrysumluteoalbum* (L.) Rchb.** – Habit: Herb. Habitat: Montane grassland, 1750–2250 m. Voucher: Tweedie EM (1976) (REF). Native distribution range: Old World.

***Helichrysummaranguense* O.Hoffm.** – Habit: Scrambling Shrub. Habitat: Montane rain forest, bamboo zone, 1950–3600 m. Vouchers: Irwin PH 147 (EA), Beentje HJ 1997 (WAG), Agnew, ADQ 7260 (WAG), Dummer RA 3591 (EA), Rono et al. SAJIT-PR 0198, SAJIT-PR 0237 (EA, HIB). Native distribution range: South Sudan to E Central & E Tropical Africa.

*Helichrysummeyeri-johannis Engl. – Habit: Herb. Habitat: Forest, afro alpine grassland, 2250–4321 m. Vouchers: Thomas AS 605, Tothill BH 2405, Thomas AS 2677, Mwangangi OM 322 (WAG, BR), Åke Strid 3427 (S), Hedberg O 130 (S), Gerh Lindblom (S), Arambourg C|Jeannel R|Chapuis PA 83 (P), Lisowski S 57256 (BR), Williams JG 74/19 (BR), Rono et al. SAJIT-PR 0255 (EA, HIB). Native distribution range: E Tropical Africa.

***Helichrysumnewii* Oliv. & Hiern** – Habit: Shrub. Habitat: Alpine stony grassland, 3000–4321 m. Vouchers: Hedberg O 968 (S, EA), Tweedie DR 94, 96, 107, 4020 (EA), Taylor G 3721 (EA), Mabberley DJ 453 (EA), Major EJ & Lugard C (EA), Gardner HM 2275 (EA), Bickford N 35 (EA), Gillett JB 18447 (EA), Knox EB 2626, 3758 (EA), Lisowski S 57278, 57279 (BR). Native distribution range: E Central & E Tropical Africa.

***Helichrysumnudifolium* (L.) Less.** – Habit: Herb. Habitat: Wet savanna, 1750–2900 m. Voucher: Holm Å 66 (S). Native distribution range: Cameroon to Ethiopia and S Africa, W Yemen.

**Helichrysumnudifoliumvar.oxyphyllum (DC.) Beentje** – Habit: Herb. Habitat: Wet savanna, 1750–2250 m. Voucher: Irwin PH 15 (BR). Native distribution range: Cameroon to Uganda and S Africa.

***Helichrysumodoratissimum* Sweet** – Habit: Herb. Habitat: Montane grassland, bushland, giant heat zones, forest margin, 1000–4000 m. Vouchers: Dummer RA 3607 (K), Granvik H 29 (S), Taiti S 5 (BR, EA), Taylor G 3790 (EA), Tweedie R 15 (EA), Joy Adamson 492 (EA), Katende T; Sheil D 980 (K), 2071 (K), Irwin PH 51 (S), Katende 916 (K), Tothill BH 2367 (EA). Native distribution range: Tropical & S Africa.

**^EX^*Helichrysumpanduratum* O.Hoffm. ex De Wild. & T.Durand** – Habit: Shrub. Habitat: Forest, 1600–2900 m. Voucher: Aldasoro J AS-65 (BC). Native distribution range: Gabon to Rwanda and S Africa.

***Helichrysumschimperi* (Sch.Bip. ex A.Rich.) Moeser** – Habit: Shrub. Habitat: Montane grassland, secondary bushland, riverine forest, giant heath zones, 1600–3400 m. Vouchers: Dale 3416 (BR), Beentje HJ 1937 (WAG), Lisowski S 57312 (WAG, BR), Agnew ADQ; Agnew S 7255 (WAG), Taylor G 3741 (S), Snowden JD 812 (P), Mwangangi OM 474 (BR), Synge PM S871 (BR), Wood G 152 (EA), Rono et al. SAJIT-PR 0214 (EA, HIB). Native distribution range: Eritrea to S Tropical Africa, Socotra, SW Arabian Peninsula.

**^EX^*Helichrysumsetosum* Harv.** – Habit: Herb. Habitat: Montane Forest, 1900–2750 m. Vouchers: Tweedie DR (BR, S), Mwangangi OM 415 (BR). Native distribution range: S Tropical & S Africa.

**^EX^*Helichrysumstenopterum* DC.** – Habit: Herb. Habitat: Montane grassland, forest, bamboo and heath zone, 600–2800 m. Vouchers: Kisalye N; van Heist M 405 (K), Naiga 714 (K), Aldasoro J AS-50 (BC), Rono et al. SAJIT-PR 0167 (EA, HIB). Native distribution range: Bioko, S Tropical & S Africa.

***Helichrysumtraversii* Chiov.** – Habit: Herb or shrub. Habitat: Woodland edges, 1750–3000 m. Vouchers: Irwin PH 142 (EA). Native distribution range: Ethiopia to Tanzania.

***Hilliardiellasmithiana* (Less.) H.Rob.** – Habit: Herb. Habitat: Wooded grassland especially on regularly burnt regions, 1350–2700 m. Vouchers: Tweedie EM (1976) (REF), Agnew ADQ (2013) (REF). Native distribution range: Tropical Africa.

***Hoffmannanthusabbotianus* (O.Hoffm.) H.Rob., S.C.Keeley & Skvarla** – Habit: Shrub or small tree. Habitat: Forest edges, riverine forest, 1000–2400 m. Vouchers: Tweedie (1976), Upland flowers and ferns. Native distribution range: Uganda to S Tropical Africa.

***Inulamannii* (Hook.f.) Oliv. & Hiern** – Habit: Herb. Habitat: Montane Forest edge, afro-alpine bamboo, scrub, 1700–3350 m. Voucher: Maitland TD sn (BR). Native distribution range: Cameroon to Ethiopia and S Tropical Africa.

***Kleiniaabyssinica* (A.Rich.) A.Berger** – Habit: Herb. Habitat: Grassland with scattered trees, wooded grassland, 1600–2800 m. Vouchers: Holm Å 60 (S), Rono et al. SAJIT-PR 0219 (EA, HIB). Native distribution range: Tropical Africa.

***Kleiniapetraea* (R.E.Fr.) C.Jeffrey** – Habit: Herb. Habitat: Outcrop rock, grassland, bushland, 1800–2450 m. Voucher: Tweedie EM (1976) (REF). Native distribution range: Kenya to Tanzania.

***Lactucaglandulifera* Hook.f.** – Habit: Herb. Habitat: Montane rain forest margin, grassland, bushland, giant heath, 1900–3600 m. Voucher: Dummer RA 3542 (K). Native distribution range: Tropical Africa.

***Lactucainermis* Forssk.** – Habit: Herb. Habitat: Grassland, disturbed ground along roadside, 500–3450 m. Voucher: Arambourg C|Jeannel R|Chappuis PA 109 (MNHN). Native distribution range: Tropical & S Africa, Madagascar, Arabian Peninsula.

***Lactucaparadoxa* Sch.Bip. ex A.Rich.** – Habit: Herb. Habitat: Forest margins, riverine vegetation, thickets, 1950–2650 m. Vouchers: Dummer RA 3639 (EA) Native distribution range: Ethiopia to Malawi.

***Laggerabrevipes* Oliv. & Hiern** – Habit: Herb. Habitat: Disturbed upland grasslands and forest edges, 1050–2400 m. Voucher: Agnew ADQ (2013) (REF). Native distribution range: Uganda to S Tropical Africa, Madagascar.

***Laggeracrispata* (Vahl) Hepper & J.R.I.Wood** – Habit: Herb. Habitat: Waste ground, moist grassland, wooded grassland, 150–2550 m. Voucher: Tweedie 2507 (EA), Rono et al. SAJIT-PR 0156 (EA, HIB). Native distribution range: Tropical & Subtropical Old World.

***Laggeraelatior* R.E.Fr.** – Habit: Herb. Habitat: Wet montane forest margins, bamboo zones, in *Hagenia* woodland, 1600–3300 m. Vouchers: Dummer 3694 (EA), Åke Strid 3414 (S), Hedberg O 152 (UPS). Native distribution range: Ethiopia to E Central & E Tropical Africa.

***Launaeacornuta* (Hochst. ex Oliv. & Hiern) C.Jeffrey** – Habit: Herb. Habitat: Wet savanna, grassland, cultivations and ruderal sites, 1750–2300 m. Voucher: Tweedie EM (1976) (REF). Native distribution range: Nigeria to Eritrea and S Tropical Africa.

***Launaeanana* (Bak.) Chiov.** – Habit: Herb. Habitat: Burnt wooded grassland, wet grassland, 1800–2600 m. Voucher: Lugard & Lugard 518 (EA). Native distribution range: Tropical & S Africa.

***Linziagerberiformis* (Oliv. & Hiern) H.Rob.** – Habit: Herb. Habitat: Burnt grassland, 1100–2400 m. Vouchers: Tweedie EM (1976) (REF), Agnew ADQ (2013) (REF). Native distribution range: Tropical & S Africa.

***Linziaituriensis* (Muschl.) H.Rob.** – Habit: Woody herb. Habitat: Forest, 1200–2550 m. Voucher: Lisowski S 57143 (BR). Native distribution range: Cameroon to South Sudan and Tanzania.

***Lipotrichepungens* (Oliv. & Hiern) Orchard** – Habit: Herb. Habitat: Wooded grassland, 1650–2300 m. Voucher: Tweedie 459 (BR, EA). Native distribution range: Tropical Africa to Namibia.

***Micractisbojeri* DC.** – Habit: Herb. Habitat: Streamside in grassland or forest, seasonally flooded grassland, 1700–2700 m. Vouchers: Dummer RA 3599 (S), Tweedie 884 (BR). Native distribution range: Nigeria to Ethiopia and Malawi, Madagascar.

***Microglossadensiflora* Hook.f.** – Habit: Shrub. Habitat: Disturbed forests, 1700–2800 m. Vouchers: Hedberg O 84 (UPS), Dummer RA 3615 (EA). Native distribution range: Tropical Africa.

***Microglossapyrifolia* (Lam.) Kuntze** – Habit: Shrub. Habitat: woodland, 1750–2650 m. Voucher: Tweedie 1532 (EA). Native distribution range: Tropical & Subtropical Old World.

**^EX^*Mikaniacordata* (Burm.f.) B.L.Rob.** – Habit: Shrub or subshrub, liane. Habitat: Riverine Forest, 0–3000 m. Voucher: Dawkins HC 780 (K), Rono et al. SAJIT-PR 0184 (EA, HIB). Native distribution range: Tropical Old World.

***Mikaniopsisbambuseti* (R.E.Fr.) C.Jeffrey** – Habit: Subshrub. Habitat: Montane forest, bamboo forest, 260–31500 m. Voucher: Wesche K 1982 (K), 474, 691 (EA). Native distribution range: Kenya to Uganda.

^**EX**^***Mikaniopsisclematoides* (Sch.Bip. ex A.Rich.) Milne-Redh.** – Habit: Subshrub. Habitat: Forest, 2250–3000 m. Voucher: Tweedie EM (1976) (REF). Native distribution range: Ethiopia.

***Monactinocephaluspaniculatus* Klatt** – Habit: Herb. Habitat: Wooded upland grassland, 1590–2500 m. Voucher: Tweedie 1466 (EA). Native distribution range: Ethiopia to S Africa.

***Nidorellaaegyptiaca* (L.) J.C.Manning & Goldblatt** – Habit: Herb. Habitat: Wet savanna, 1750–2600 m. Voucher: Lugard C & Lugard EJ 210 (EA). Native distribution range: Africa to Indo-China.

***Nidorellavernonioides* Sch.Bip. ex A.Rich.** – Habit: Shrub or small weak tree. Habitat: Upper mountain steep slopes, bamboo zone, 3500–4000 m. Vouchers: Thomas AS 555 (EA), Mwangangi OM 366 (EA), Taylor G 3670 (EA), Holm Å 52, 53 (S), Taylor G 3683 (S), Arambourg C|Jeannel R|Chappuis PA 157 158 (MNHN). Native distribution range: Djibouti to E Central & E Tropical Africa.

***Nidorellawelwitschii* S.Moore** – Habit: Herb or shrub. Habitat: Grassland, alpine zone, moorland, 1800–4321 m. Voucher: Tweedie 20 (EA), Taylor 3521 (EA). Native distribution range: South Sudan to S Tropical Africa.

***Orbivestuskaraguensis* (Oliv. & Hiern) H.Rob.** – Habit: Herb or weak shrub. Habitat: Wooded grassland, grassland, forest margins, old cultivations, 1200–2850 m. Vouchers: Hedberg O 1073 (UPS), Holm Å 72 (S). Native distribution range: Ethiopia to S Tropical Africa.

***Orbivestusturbinata* (Oliv. & Hiern ex Oliv.) H.Rob.** – Habit: Herb. Habitat: Wooded grassland, grassland, 1400–2400 m. Voucher: Irwin PH 56 (S, BR). Native distribution range: DR Congo to Ethiopia and Kenya.

***Osteospermumvaillantii* (Decne.) Norl.** – Habit: Herb. Habitat: Woodland, forest edge, 600–3150 m. Vouchers: Bamps PRJ 6502 (BR), Tweedie (S). Native distribution range: S Jordan to NE & E Tropical Africa, Arabian Peninsula.

***Piloselloideshirsuta* (Forssk.) C.Jeffrey ex Cufod.** – Habit: Herb. Habitat: Upland forest, grasslands, woodlands, heath and moor, 1500–3700 m. Vouchers: Hedberg O 847 (S), Irwin PH 1001 (BR). Native distribution range: Tropical & S Africa to China and Indo-China, Lesser Sunda Islands (Bali).

*Seneciocrispatipilosus C.Jeffrey – Habit: Herb. Habitat: Bamboo Forest, heath zone, 2050–3050 m. Vouchers: Wesche K 601 (K), Tothill BH T2372 (K), T2290 (K), 2372 (K), Dummer RA 3598 (K). Native distribution range: Uganda to Kenya.

***Seneciohadiensis* Forssk.** – Habit: Herb or shrub succulent trailer or climber. Habitat: Upland forest edges, 1000–2600 m. Vouchers: Irwin PH 74 (S), Rono et al. SAJIT-PR 0031 (EA, HIB). Native distribution range: Eritrea to Zimbabwe, Madagascar, Arabian Peninsula.

***Seneciohochstetteri* Sch.Bip. ex A.Rich.** – Habit: Herb or shrub with woody ascending stems. Habitat: Grassland, forest margin, wooded grassland, 1200–3700 m. Vouchers: Major EJ; Lugard C 541 (K, EA), Irwin PH 141 (K), Tweedie 1175 (K). Native distribution range: Tropical & S Africa.

**Seneciojacksonii S.Moore – Habit: Herb. Habitat: Alpine zone, outcrop rock, 3250–4150 m. Vouchers: Mwangangi OM 315 (WAG), (K), Wesche K 30 (K), Lye KA; Pocs T 23089 (K), Tweedie R 8 (K), Hedberg O 4485 (EA, K), Liebenberg LCC 1583 (EA, K) Ekkens DB 401 (EA), Adamson J 466 (EA), Tothill BH 2416 (EA). Native distribution range: Uganda to Kenya (Mt Elgon).

*Seneciomoorei R.E.Fr. – Habit: Woody herb or shrub. Habitat: Grassland, forest clearing, 1800–3500 m. Vouchers: Wood G 153 (EA), Rono et al. SAJIT-PR 0236 (EA, HIB). Native distribution range: Kenya to Uganda.

**Seneciorhammatophyllus Mattf. – Habit: Shrub. Habitat: Moorland with grassland and giant heath, forest edges, 3000–4150 m. Vouchers: Taylor G 3500 (EA), Wesche K 90 (K), Hedberg O 866 (K), (S), Lugard C 306 (EA, K), Osmaston HA 4000 (K), Saunder &Hancock 57, Osmaston 4000 (EA), Hedberg 4513 (EA). Native distribution range: Uganda to Kenya (Mt Elgon).

***Senecioruwenzoriensis* S.Moore** – Habit: Herb. Habitat: Wooded grassland, shallow soil among rocks, cultivations, 1700–3000 m. Vouchers: Polhill 401 (EA), Hedberg O 811 (UPS). Native distribution range: Tropical & S Africa.

**Seneciosnowdenii Hutch. – Habit: Herb. Habitat: Clearing from bamboo zone, grassland, 2700–4250 m. Vouchers: Lind EM 2086 (EA), Friedberg A 146 (EA), Gardner HM 2268 (K), Mwangangi OM 320 (K), Townsend CC 2328 (K), Smith SAL; Beentje, Muasya AM 175 (K), Wesche K 4 (K), Hedberg O 227 (K), Gillett 1867 (EA), Tothill BH 2314 (EA), Thomas AS 2681 (EA), Hedberg 4455 (EA). Native distribution range: Uganda to Kenya (Mt Elgon).

**Seneciosotikensis S.Moore – Habit: Woody herb or soft shrub. Habitat: Marshy grassland, 3000–4300 m. Vouchers: Lugard C; Lugard EJ 416 (EA, K), Adamson J 481 (EA), Hedberg O 871 (EA), Kokwaro JO 2464 (EA), Gillett JB 18435, (BR), Kokwaro JO 2464 (K), Gillett JB 18435 (K), Snowden JD 482 (K), Wesche K 207 (K), Hedberg O 4453 (UPS), Dummer RA 3324 (EA), Lye et al. 5726 (EA). Native distribution range: Uganda to Kenya (Mt Elgon).

***Seneciosyringifolius* O.Hoffm.** – Habit: Twining liana. Habitat: Montane forest, bamboo zone, 1500–3300 m. Voucher: Hedberg O 58 (S). Native distribution range: Kenya to S Tropical Africa.

***Seriphiumkilimandscharicum* (O.Hoffm.) Koek.** – Habit: Herb. Habitat: Heath zone, moorland, upper forest edge, (1950–)2400–3900 m. Vouchers: Tweedie EM 1297 (K), Lisowski S 54497 (BR), Dummer RA 3532 (P), Snowden JD 803 (P), Taiti S 582 (EA), Hedberg O 134 (S). Native distribution range: Uganda to N Malawi.

**^EX^*Solanecioangulatus* (Vahl) C.Jeffrey** – Habit: Succulent herb. Habitat: Upland forest, woodland edges, riverine and streams, 1000–2500 m. Voucher: Agnew ADQ (2013) (REF). Native distribution range: Tropical & S Africa, Mayotte, Madagascar, SW Arabian Peninsula.

**^EX^*Solaneciomannii* (Hook.f.) C.Jeffrey** – Habit: Shrub or small tree. Habitat: Disturbed forest, 1400–2700 m. Vouchers: Hedberg O 55 (S), Taylor G 3436 (S), Synge PM S 104 (S), Bamps PRJ 6501 (BR). Native distribution range: Nigeria to Ethiopia and S Tropical Africa.

***Solaneciotuberosus* (Sch.Bip. ex A.Rich.) C.Jeffrey** – Habit: Herb. Habitat: Swampy grassland, 1200–2000 m. Voucher: Tweedie DR 162 (K). Native distribution range: Eritrea to Uganda.

***Sonchusafromontanus* R.E.Fr.** – Habit: Herb. Habitat: Grassland, along streamside, forest clearing, 2200–3700 m. Vouchers: Lye 5734 (EA), Tweedie 24 (EA), Tweedie R 25 (K), Tweedie 2550 (K). Native distribution range: E Central & E Tropical Africa.

^**EX**^***Sonchusasper* (L.) Hill** – Habit: Herb. Habitat: Wet savanna, cultivation, 1750–2550 m. Voucher: Tweedie 1336 (EA). Native distribution range: Temperate Eurasia, N Africa to Sahel and Somalia.

***Sonchusbipontini* Asch.** – Habit: Herb. Habitat: Wooded grassland, bamboo zone, 1700–3370 m. Vouchers: Hedberg O 66 (K), Tweedie 888 (K), Mwangangi OM 392 (K), Kokwaro JO 2448 (K), Hooper SS; Townsend CC 1389 (K), Hemp A 5416 (W). Native distribution range: Ethiopia to S Tropical Africa.

*Sonchuscamporum (R.E.Fr.) Boulos ex C.Jeffrey – Habit: Herb. Habitat: Rare in upland burnt grassland, 1800–2300 m. Voucher: Lugard & Lugard 546 (K). Native distribution range: Kenya.

***Sonchusluxurians* (R.E.Fr.) C.Jeffrey** – Habit: Herb. Habitat: Roadsides, grassland, montane forest, 1600–3800 m. Voucher: Rono et al. SAJIT-PR 0155 (EA, HIB). Native distribution range: Ethiopia to Burundi and Mozambique.

^**EX**^***Sonchusoleraceus* L.** – Habit: Herb. Habitat: Roadsides, cultivations, 1500–2700 m. Voucher: Major EJ & Lugard C 457 (EA). Native distribution range: Macaronesia, Europe to Mediterranean, Sahara to Arabian Peninsula.

***Sonchusschweinfurthii* Oliv. & Hiern** – Habit: Herb. Habitat: Grassland, weed of cultivtion, roadside, 1700–2800 m. Vouchers: Mwangangi OM 392 (BR), Agnew ADQ; Agnew S 7250 (WAG). Native distribution range: Tropical Africa.

*Sonchusstenophyllus R.E.Fr. – Habit: Herb. Habitat: Forest glades, grassland, 1700–3300 m. Voucher: Agnew ADQ (2013) (REF). Native distribution range: Kenya to Tanzania.

***Sphaeranthussuaveolens* DC.** – Habit: Herb. Habitat: Near fresh stream beds, wet savanna, 1200–2500 m. Vouchers: Beentje HJ 1963 (WAG), Hedberg O 1953 (S), Holm Å 74 (S), Small W 1184 (K). Native distribution range: N Sinai, Egypt to S Tropical Africa.

^**EX**^***Tagetesminuta* L.** – Habit: Herb. Habitat: Upland arable land, wet savanna, 850–2750 m. Voucher: Hedberg O 6 (S). Native distribution range: Brazil to S South America.

***Vernonellachthonocephala* (O.Hoffm.) H.Rob. & Skvarla** – Habit: Herb. Habitat: Seasonally burnt grasslands, 1050–2100 m. Voucher: James (EA). Native distribution range: Tropical Africa.

***Vernoniagalamensis* (Cass.) Less.** – Habit: Herb. Habitat: Woodland edges, 800–2800 m. Vouchers: Tweedie 726 (K), Lugard C; Lugard EJ 252 (K), Bridson DM 70 (WAG), Smith CE; Njoroge D 4640 (K). Native distribution range: Africa.

***Vernoniaholstii* O.Hoffm.** – Habit: Herb or shrub. Habitat: Forest margin, secondary bushland, 1000–2100 m. Voucher: Tweedie 3729 (EA). Native distribution range: Cameroon to Kenya and Zimbabwe.

^***EX***^***Vernoniaschweinfurthii* Oliv. & Hiern** – Habit: Herb. Habitat: Wet savanna, short grassland, scattered tree grassland, 1750–2250 m. Voucher: Tweedie 1115 (EA). Native distribution range: Tropical Africa.

***Vernoniasyringifolia* O.Hoffm.** – Habit: Woody herb or weak shrub. Habitat: Montane rain forest, forest margin, secondary woodland or bush, 2000–3050 m. Vouchers: Dummer RA 3500 (EA), Agnew ADQ; Agnew S 7283 (WAG), Taylor G 3794 (S), Irwin PH 45 (S). Native distribution range: South Sudan to S Tropical Africa.

#### F44. BALSAMINACEAE

1 Genus, 10 species

**Impatiensdigitatasubsp.phlyctidoceras (Bullock) Grey-Wilson – Habit: Herb. Habitat: Forest, 3500–3700 m. Vouchers: Sheil|Musinguzi RD 1809 (MO), Dale IR, Dale in FD 3201 (MO), Lugard CE 313 (MO). Native distribution range: E Tropical Africa (Mt Elgon).

*Impatiensdigitata Warb. – Habit: Herb. Habitat: Forest floor, moorland, about 3500 m. Voucher: Lugard C 313 (K, EA). Native distribution range: Kenya, Uganda, Tanzania (Mt Kilimanjaro).

***Impatienshochstetteri* Warb.** – Habit: Herb. Habitat: Stream banks in forest, woodland, 1000–2800 m. Vouchers: Knox EB 2633 (EA), Tweedie EM 3603 (K), Synge PM S1056 (BR), Synge PM S 1037 (WAG), Rono et al. SAJIT-PR 0004 (EA, HIB). Native distribution range: Tropical & S Africa.

***Impatiensmeruensis* Gilg** – Habit: Herb. Habitat: streamside, marshes, forest, 1000–3550 m. Vouchers: Knox EB 2640, 3832 (EA), Bridson DM 72 (EA), Jack C 110 (EA), Rono et al. SAJIT-PR 0048 (EA, HIB). Native distribution range: N Tanzania, Kenya, Uganda, Sudan.

*Impatiensmeruensissubsp.cruciata (T.C.E.Fr.) Grey-Wilson – Habit: Herb. Habitat: Moist shaded forest fringes, bushland, bamboo, 2250–3630 m. Vouchers: Wesche K 886 (K), Bridson DM 72 (K), Tweedie 365 (K), Tweedie 1437 (K). Native distribution range: Sudan to Kenya.

**Impatiensminiata Grey-Wilson – Habit: Herb. Habitat: Montane forest, forest fringes, bamboo thickets, 2850–3700 m. Vouchers: Tweedie 7A (EA), Thomas AS 510, 225 (EA), Lye 5748 (EA). Native distribution range: E Tropical Africa (Mt Elgon).

***Impatienssodenii* Engl. & Warb. ex Engl.** – Habit: Herb. Habitat: Escarpment zones and waterfalls, 1000–2700 m. Voucher: Granvik H sn (S). Native distribution range: Kenya to N & E Tanzania.

***Impatienstinctoria* A.Rich.** – Habit: Herb. Habitat: Waterfalls and stream banks, forests, 1800–3630 m. Vouchers: Knox EB 2639 (EA), Rono et al. SAJIT-PR 0043 (EA, HIB). Native distribution range: Eritrea to N Uganda.

**Impatienstinctoriasubsp.elegantissima (Gilg) Grey-Wilson** – Habit: Herb. Habitat: Outcrop rock, forest, 1800–3000 m. Vouchers: Tweedie (K), Lind EM 437 (K), Snowden JD 507 (K), Bridson DM 73 (K). Native distribution range: SE Uganda to Kenya.

**Impatienstweedieae E.A.Bruce – Habit: Herb. Habitat: Forest and forest floor, upland grassland, 3000–3750 m. Vouchers: Wesche K 1926 (MO), Synge 891 (EA), Morrison MES 258a (MO), Tweedie 366 (MO, BR), 367 (K), Adamson 53 (EA), Adamson J 503 (MO), Thomas AS 2709 (MO), Thomas AS 585 (MO), 2709 (EA), Tweedie EM 367 (MO), Taylor 3737 (S). Native distribution range: E Tropical Africa (Mt Elgon).

#### F45. BASELLACEAE

1 Genus, 1 species

**^EX^*Basellaalba* L.** – Habit: Herb or shrub. Habitat: Riverine forest, forest edges, margins of cultivated land, 1500–3000 m. Vouchers: Tweedie 2043 (EA), Irwin PH 526 (EA). Native distribution range: Tropical Asia.

#### F46. BEGONIACEAE

1 Genus, 1 species

***Begoniawollastonii* Baker f.** – Habit: Herb. Habitat: Spray of waterfall, forest, 1750–2900 m. Vouchers: Thomas AS 2578 (EA), Wesche 600 (EA), Tweedie 1485 (EA), Synge PM 1036 (WAG). Native distribution range: Ethiopia to SW Tanzania.

#### F47. BIGNONIACEAE

3 Genera, 4 species

***Kigeliaafricana* (Lam.) Benth.** – Habit: Tree. Habitat: Wet savanna, 1750–2250 m. Voucher: Tweedie EM (1976) (REF). Native distribution range: Tropical & S Africa

**Kigeliaafricanasubsp.moosa (Sprague) Bidgood & Verdc.** – Habit: Tree. Habitat: Forest, upland bushland, spray of waterfall, 1750–2400 m. Vouchers: Snowden JD 866 (EA, MO). Native distribution range: Tropical Africa.

***Markhamialutea* K.Schum.** – Habit: Tree. Habitat: Woodland, 1750–2400 m. Voucher: Tweedie EM (1976) (REF). Native distribution range: Ghana to South Sudan and Tanzania.

***Stereospermumkunthianum* Cham.** – Habit: Tree or shrub. Habitat: Wet savanna, 1750–2250 m. Voucher: Rwaburindore PK 5621 (MO). Native distribution range: Tropical Africa.

#### F48. BORAGINACEAE

6 Genera, 16 species

***Cordiaafricana* Lam.** – Habit: Shrub or small tree. Habitat: Forest, grassland, 1750–2400 m. Voucher: Snowden JD 926 (EA). Native distribution range: Tropical & S Africa, SW Arabian Peninsula, Comoros, Madagascar.

***Cynoglossumaequinoctiale* T.C.E.Fr.** – Habit: Herb. Habitat: Montane grassland, 1770–2870 m. Vouchers: Irwin PH 12 (K), Saundy; Hancock 95 (EA, K). Native distribution range: Kenya to N Zambia.

***Cynoglossumamplifolium* Hochst. ex A.DC.** – Habit: Herb. Habitat: Grassland, woodland, forest, bamboo zonne, 1800–3430 m. Voucher: Tweedie 4097 (EA). Native distribution range: Ethiopia to S Tropical Africa.

**Cynoglossumamplifoliumvar.subalpinum (T.C.E.Fr.) Verdc.** – Habit: Herb or subshrub. Habitat: Upper forest edge, acacia woodland, bamboo forest, 1800–3200 m. Voucher: Tweedie 4097 (EA). Native distribution range: Nigeria, Cameroon, SE Ethiopia to Tanzania.

***Cynoglossumcheranganiense* Verdc.** – Habit: Herb. Habitat: Grassland with scattered bamboo, Junipera-Hagenia clumps, 2400–3270 m. Voucher: Wesche K 1644 (EA). Native distribution range: Kenya.

***Cynoglossumcoeruleum* Hochst. ex DC.** – Habit: Herb. Habitat: Forest clearing and pathside, 1100–3200 m. Vouchers: Symes YE 241 (EA), Lind EM 430 (EA), Rono et al. SAJIT-PR 0119 (EA, HIB). Native distribution range: Ethiopia to S Africa.

**Cynoglossumcoeruleumsubsp.johnstonii (Baker) Verdc.** – Habit: Herb. Habitat: Grassland, overgrazed grassland, waste ground, 2400–3650 m. Voucher: Bei 66283 (EA). Native distribution range: Cameroon, Ethiopia to N Malawi.

**Cynoglossumcoeruleumsubsp.kenyense Verdc.** – Habit: Herb. Habitat: Grassland, grassland with scattered trees, 1440–3150 m. Voucher: Symes 241 (EA). Native distribution range: Kenya to N Tanzania.

**Cynoglossumcoeruleumvar.mannii (Baker & C.H.Wright) Verdc.** – Habit: Herb. Habitat: Submontane forest, swamp, bamboo thickets, margins of cultivated land, 1740–3150 m. Voucher: Dummer RA 3601 (EA). Native distribution range: Cameroon to Ethiopia and S Africa.

***Cynoglossumlanceolatum* Forssk.** – Habit: Herb. Habitat: Weed and in disturbed grasslands, 1100–3220 m. Vouchers: Lugard 175 (EA), Snowden JD 886 (EA), Rono et al. SAJIT-PR 0134 (EA, HIB). Native distribution range: Tropical & S Africa to Tropical & Subtropical Asia.

**Ehretiacymosavar.silvatica (Gurke) Brenan** – Habit: Tree. Habitat: Forest, riverine forest,1750–2250 m. Vouchers: Lugard EJ & Lugard C 554a, Jackson THE 305 (EA), Tweedie 2587 (EA), Jack C 166, 276 (EA). Native distribution range: Eritrea to S Tropical Africa.

***Lithospermumafromontanum* Weim.** – Habit: Herb. Habitat: Montane forest edges, moorland, upper Hagenia- Rapanea woodland, heath zone, 2100–4000 m. Voucher: Dummer RA 3433 (EA). Native distribution range: Ethiopia to South Africa.

***Myosotisabyssinica* Boiss. & Reut.** – Habit: Herb. Habitat: Woodland, forest, grasslands, moorland zone, 2000–3960 m. Voucher: Tweedie 1898 (EA), Hedberg O 187, 4565 (EA). Native distribution range: Bioko to Cameroon, Ethiopia to Tanzania.

***Myosotiskeniensis* T.C.E.Fr.** – Habit: Mat-forming herb. Habitat: Grassland tussock in dendrosenecio associations, 3350–4320 m. Voucher: Bie SW 302 (EA). Native distribution range: S Central Ethiopia, Central Kenya.

***Myosotisvestergrenii* Stroh** – Habit: Herb. Habitat: Alpine and heath zone, 2000–4250 m. Vouchers: Bally J 472, Tweedie DR 4, 123 (EA), Trelawny BBR 4467 (EA), Adamson J 472 (EA), Gardner HM 2247 (EA), Hedberg O 215 (EA) Bickford N 6 (EA) Lind EM 2094 (EA), Forbes 259 (EA). Native distribution range: Ethiopia to E Tropical Africa

***Trichodesmaphysaloides* A.DC.** – Habit: Herb. Habitat: Woodland, wooded grassland, bushland, 1050–2400 m. Vouchers: Tweedie 138 (EA), Snowden JD 821 (EA), Webster MVB 8916 (EA). Native distribution range: Ethiopia to South Africa.

#### F49. BRASSICACEAE

11 Genera, 16 species

***Arabidopsisthaliana* (L.) Heynh.** – Habit: Herb. Habitat: Upland bushland and moor, disturbed alpine grassland, 1750–4250 m. Voucher: Sound & Hancock 39 (EA), Tothill BH 2450 (EA), Hedberg 943 (EA). Native distribution range: Temperate Eurasia to Tropical African Mountains.

***Arabisalpina* L.** – Habit: Herb. Habitat: Upland moor, stream banks, cliffs, 2450–4321 m. Vouchers: Taylor 3531 (EA), Wesche K 206 (K), 1247 (EA), Dummer RA 3438 (K), Hedberg O 1002 (EA, K), 969 (EA), Townsend CC 2323 (K), Bickford N 16, 43 (EA), Gardner HM 2244 (EA), Adamson J 473 (EA), Tweedie DR 108 (EA), Synge PM 943 (EA). Native distribution range: N and Central Europe to W Central Siberia, N & E Canada to Greenland.

***Barbareaintermedia* Boreau** – Habit: Herb. Habitat: Upland moor, alpine zone, disturbed ground, streamside, 3000–3900 m. Vouchers: Adamson J 495 (K), Hedberg O 1013 (EA, K), Townsend CC 2331 (EA, K), Dale IR 3391 (EA, K), Major EJ; Lugard C 393 (K), Major EJ; Lugard C 419 (K), Ekkens DB 2476 (EA). Native distribution range: Central & S Europe to NW China and E Uganda.

^**EX**^***Brassicajuncea* (L.) Czern.** – Habit: Herb. Habitat: Cultivation, forest, 1650–2000 m. Voucher: Tweedie 1136A (EA). Native distribution range: North Caucasus, Transcaucus, a cultigen from China.

^**EX**^***Brassicanapus* L.** – Habit: Herb. Habitat: Shrubland, 1700–2300 m. Vouchers: Tweedie 1136 (EA). Native distribution range: A cultigen from S Europe.

**^EX^*Capsellabursa-pastoris* (L.) Medik.** – Habit: Herb. Habitat: Cultivated grounds, forest, woodland, 1500–2800 m. Vouchers: Stunrock BM 2207 (EA), Beentje HJ 1945 (EA). Native distribution range: Temperate Eurasia, N Africa.

***Cardamineafricana* L.** – Habit: Herb. Habitat: Forest, stream side, 2200–3400 m. Vouchers: Hedberg O 5081 (UPS), H 1048 (EA, S), Wesche K 480 (EA). Native distribution range: Tropical & S Africa, India to W Malesia, Central & S Tropical America.

***Cardaminehirsuta* L.** – Habit: Herb. Habitat: Forest, 500–4321 m. Vouchers: Hedberg 268 (EA), Rono et al. SAJIT-PR 0147 (EA, HIB). Native distribution range: Temperate & Subtropical Northern Hemisphere to Tropical African Mountains.

***Cardamineobliqua* Hochst. ex A.Rich.** – Habit: Herb. Habitat: Alpine belt, montane forest, 2000–4321 m. Vouchers: Hedberg O 862 (K), 4464 (EA), 4521 (EA, K), Bush RZ 249 (EA), Bickford N 57 (EA), Irwin PH 361 (EA), Major C & Lugard EJ 355 (EA), Synge 958 (EA), Tweedie DR 70 (EA). Native distribution range: Ethiopia to Tanzania, Mexico to Ecuador.

***Cardaminetrichocarpa* Hochst. ex A.Rich.** – Habit: Herb. Habitat: Roadside, forest clearings, 950–3000 m. Vouchers: Tweedie EM (1976) (REF), Agnew ADQ (2013) (REF). Native distribution range: S Cameroon to Ethiopia and Tanzania, South Africa, India to Assam, Sri Lanka.

***Crambekilimandscharica* O.E.Schulz** – Habit: Herb. Habitat: Grassland, forest clearing, weed in cultivated land, 1200–3400 m. Vouchers: Hedberg KO 4474 (EA, BR), Muthoka P & Kirika P 1178 (EA). Native distribution range: S Ethiopia to E DR Congo.

***Erucastrumarabicum* Fisch. & C.A.Mey.** – Habit: Herb. Habitat: Wet savanna, woodland, 1750–3170 m. Voucher: Symes YE 119 (EA). Native distribution range: Egypt to Namibia, Arabian Peninsula.

***Erucastrumelgonense* Jonsell** – Habit: Herb. Habitat: Montane forest, ericaceous scrub, 3050–3400 m. Vouchers: Thomas AS 586 (EA), Tothill BH 2346 (EA), Hedberg Olov 4475 (UPS, EA, K). Native distribution range: Uganda (Mt Elgon).

^**EX**^***Mummenhoffiaalliacea* (L.) Esmailbegi & Al-Shehbaz** – Habit: Herb. Habitat: Montane forests, upland moor, stream banks, bamboo, 3000–3600 m. Voucher: Hedberg 997 (EA). Native distribution range: E Central & S Europe to N Turkey.

***Oreophytonfalcatum* O.E.Schulz** – Habit: Herb. Habitat: Alpine zone, rock crevices, 3800–4321 m. Vouchers: Hedberg in Adamson J 500 & Hedberg 978 (EA), Tweedie 70 (EA). Native distribution range: Ethiopia to E Central & E Tropical African Mountains.

***Subulariamonticola* A.Braun ex Schweinf.** – Habit: Tufted herb. Habitat: Upland rain-forest, moorland, ericaceous and alpine zone, 2750–4321 m. Vouchers: Hedberg 1016 (EA), Synge PM 886 (EA), Wesche K 1941 (EA). Native distribution range: Mountains of Ethiopia to E DR Congo and N Tanzania.

#### F50. CAMPANULACEAE

4 Genera, 24 species

***Canarinaabyssinica* Engl.** – Habit: Climbing herb. Habitat: Forest, wooded grassland, along streams, 1925–2300 m. Vouchers: Tweedie 860 (EA), Lind EM 262 (EA). Native distribution range: Ethiopia to E Tropical Africa.

***Canarinaeminii* Asch. & Schweinf.** – Habit: Climbing (or hanging if epiphyte) herb. Habitat: Montane Forest, Outcrop rocks, 1600–3200 m. Vouchers: Hedberg 158 (EA), Thomson 2253 (EA), Rwaburindore PK 469 (WAG), Norman EM 223 (K), Andersen 327 (S), Granvik H 331 (S). Native distribution range: Ethiopia to N Malawi.

*Lobeliaaberdarica R.E.Fr. & T.C.E.Fr. – Habit: Shrub. Habitat: Swamps in the moorland, 1860–3500 m. Vouchers: Dale IR 3200 (EA), Awich Y 607 (EA), Major C & Lugard EJ 434 (EA), Tweedie R 109 (EA), Knox EB 3769, 3768, 3767, 2615 (EA), Hedberg O 1045 (S), Thomas AS 654 (EA), Synge 872 (EA), Hedberg 4536 (EA). Native distribution range: E Tropical Africa (Aberdare Range).

***Lobeliacheranganiensis* Thulin** – Habit: Trailing herb. Habitat: Upland Forest and moor, 2750–3300 m. Voucher: Agnew ADQ (2013) (REF). Native distribution range: Kenya.

***Lobeliaduriprati* T.C.E.Fr.** – Habit: Herb. Habitat: Wet montane short grassland, 1700–3550 m. Voucher: Rono et al. SAJIT-PR 0109 (EA, HIB). Native distribution range: E Tropical Africa.

***Lobeliafervens* Thunb.** – Habit: Herb. Habitat: Wet savanna, woodland, upper forest edge, 1750–3150 m. Voucher: Tweedie EM (1976) (REF). Native distribution range: Ethiopia to S Tropical Africa, W Indian Ocean.

***Lobeliaflaccida* A.DC.** – Habit: Herb. Habitat: Grassland, forest margin, 1220–3150 m. Vouchers: Tweedie DR 33 (EA), Knox EB 2607, 2631, 2638, 2643, 3833 (EA), Beentje HJ 1936 (EA), Webster MVB 8909 (EA), Gilbert MG & Tadesse M 6553 (EA), Symes YE 415 (EA), Bitsford N 49 (EA), Granvik H 254 (S). Native distribution range: Sudan to S Africa.

**Lobeliaflaccidasubsp.granvikii (T.C.E.Fr.) Thulin** – Habit: Herb. Habitat: Forest, grassland, 1524 m. Vouchers: Lugard E 149 (K), Dummer RA 3722 (K). Native distribution range: South Sudan to Kenya.

***Lobeliagiberroa* Hemsl.** – Habit: Shrub. Habitat: Disturbed places of montane forests, 1590–3000 m. Voucher: Dummer RA 3575 (US). Native distribution range: Eritrea to Zambia.

*Lobeliagregorianasubsp.elgonensis (R.E.Fr. & T.C.E.Fr.) E.B.Knox – Habit: Herb. Habitat: Upland forest, moorland, 3450–4200 m. Vouchers: Adamson J 499 (EA), Hedberg KO 922 (MO, EA), Synge PM 911 (EA, MO), Lindblom sn (K), Liebenberg LCC 1674 (EA, MO), Katende AB 318 (MO), Dale U70 (EA). Native distribution range: E Tropical Africa (Cherangani Hills & Mt Elgon).

**^EX^*Lobeliaheyneana* Roem. & Schult.** – Habit: Herb. Habitat: Woodland, lower and upper forest edge, 1750–3150 m. Voucher: Tweedie EM (1976) (REF). Native distribution range: Eritrea to Zambia, Arabian Peninsula, China (Yunnan) to Tropical Asia.

***Lobeliainconspicua* A.Rich.** – Habit: Herb. Habitat: Short grassland and disturbed grounds, 1220–2400 m. Voucher: Agnew ADQ (2013) (REF). Native distribution range: Tropical Africa.

*Lobelialindblomii Mildbr. – Habit: Creeping/ mat-forming herb. Habitat: Alpine and subalpine grassland, 3100–4250 m. Vouchers: Hedberg 123, 4499 (EA), Major EJ; Lugard C 432 (K), 433 (K), Tweedie DR 4017 (K), Adamson J 502 (K), Mabberley DJ 468 (EA, K), Knox EB 3837 (EA), Williams Y & Beentje 1999 (EA), Rauh W 586 (EA), Bickford N 6 (EA), Thomas AS 602 (EA), Wood 111 (EA). Native distribution range: E Tropical Africa (Aberdare Mountains, Mt Elgon).

***Lobeliaminutula* Engl.** – Habit: Herb. Habitat: Upland grassland, moorland, forest margin, 2125–3350 m. Voucher: Ekkens DB 415 (EA). Native distribution range: Cameroon to E Tropical Africa.

***Lobelianeumannii* T.C.E.Fr.** – Habit: Herb. Habitat: Upland grassland, 1850–2750 m. Voucher: Agnew ADQ (2013) (REF). Native distribution range: Cameroon, Ethiopia to Kenya.

*Lobeliatelekii Schweinf. – Habit: Herb. Habitat: Wet stony grounds in moorlands, 3000–5000 m. Vouchers: Tweedie DR 26 (EA), Synge PM 5901 (EA, BR), Hedberg O 861 (EA) Mwangangi OM 338 (EA), Ekkens DB 776 (EA), Honore EJ 2519 (EA), Dale IR 3194 (EA), Knox EB 2624 (EA), Tothill BH 2412 (BR, EA), Eggeling WJ 5751 (EA), Dummer RA 3385 (US, UPS), Tweedie DR Mrs 8 (K), Liebenberg LCC 1617 (EA), Thomas AS 629 (EA). Native distribution range: E Tropical Africa (Aberdare Range, Mt Kenya, Mt Elgon).

***Monopsisstellarioides* Urb.** – Habit: Trailing herb. Habitat: Paddock, roadside, alpine grassland, 1650–3600 m. Vouchers: Symes YE 519 (EA), Hedberg O 909 (EA), Webster MVB 8912 (EA), Beentje HJ 1964 (EA), KnoX EB 3834 (EA), Tweedie EM 126 (K). Native distribution range: Tropical & S Africa, Comoros

***Wahlenbergiacapillacea* A.DC.** – Habit: Herb. Habitat: Grassland, bamboo zone, 2300–3500 m. Voucher: Agnew ADQ (2013) (REF). Native distribution range: Kenya to S Africa.

***Wahlenbergiahirsuta* (Edgew.) Tuyn** – Habit: Herb. Habitat: Grassland, woodland, waste land, roadside, 1650–2120 m. Voucher: Agnew ADQ (2013) (REF). Native distribution range: Tropical & S Africa to Himalaya.

***Wahlenbergiakrebsii* Cham.** – Habit: Herb. Habitat: Grassland, Lower alpine and heath zones, upper forest margins, 2130–3600 m. Vouchers: Dummer RA 3515 (US), Rono et al. SAJIT-PR 0143, SAJIT-PR 0265 (EA, HIB). Native distribution range: Bioko to Ethiopia and S Africa.

***Wahlenbergianapiformis* (A.DC.) Thulin** – Habit: Herb. Habitat: Wet savanna, grassland, old cultivations, 1750–2250 m. Voucher: Tweedie EM (1976) (REF). Native distribution range: Central African Republic to Ethiopia and Namibia.

***Wahlenbergiapusilla* Hochst. ex A.Rich** – Habit: Mat forming Herb. Habitat: Alpine and moor zone, 2800–4300 m. Vouchers: Hedberg O 140 (K) 3250 (EA), Wesche K 329 (K), Taylor G 3536 (BR). Native distribution range: Ethiopia to Tanzania.

***Wahlenbergiasilenoides* Hochst. ex A.Rich.** – Habit: Herb. Habitat: Montane grassland, moorland, forest margin, 2200–3350 m. Voucher: Tweedie 3534 (EA). Native distribution range: Nigeria to Ethiopia and Tanzania.

***Wahlenbergiavirgata* Engl.** – Habit: Herb. Habitat: Grasslands, 1680–2700 m. Voucher: Hedberg 26 (EA). Native distribution range: Ethiopia to S Africa.

#### F51. CANELLACEAE

1 Genus, 1 species

***Warburgiaugandensis* Sprague** – Habit: Tree. Habitat: Riverine, upland forest, wooded grassland, 1600–2400 m. Voucher: Rono et al. SAJIT-PR 0013 (EA, HIB). Native distribution range: SE Ethiopia to Malawi.

#### F52. CANNABACEAE

1 Genus, 1 species

***Celtisafricana* Burm.f.** – Habit: Tree. Habitat: Forest, 2250–3000 m. Voucher: Rono et al. SAJIT-PR 0042 (EA, HIB). Native distribution range: Tropical & S Africa, SW Arabian Peninsula, Madagascar.

#### F53. CAPPARACEAE

3 Genera, 3 species

***Capparisfascicularis* DC.** – Habit: Shrub. Habitat: Grassland, forest edge, 1750–2250 m. Voucher: Tweedie EM (1976) (REF). Native distribution range: Tropical & S Africa.

***Maeruaangolensis* DC.** – Habit: Shrub or small tree. Habitat: Grassland, forest edge, 1750–2250 m. Voucher: Tweedie EM (1976) (REF). Native distribution range: Tropical & S Africa.

***Ritchieaalbersii* Gilg** – Habit: Shrub or tree. Habitat: Upland forest margin, 2250–3000 m. Vouchers: Rono et al. SAJIT-PR 0066 (EA, HIB). Native distribution range: Nigeria to Ethiopia and S Tropical Africa.

#### F54. CAPRIFOLIACEAE

4 Genera, 5 species

***Cephalariapungens* Szabo** – Habit: Herb. Habitat: Montane and subalpine grassland, 2500–3000 m. Voucher: Agnew ADQ (2013) (REF). Native distribution range: W Kenya to S Africa.

***Dipsacuspinnatifidus* Steud. ex A.Rich.** – Habit: Herb. Habitat: Upper Forest, lower alpine zone, heath woodland, 2000–3950 m. Vouchers: Hedberg 1027 (EA), Lisowski S 11786 (BR), Liebenberg 1614 (EA), Rono et al. SAJIT-PR 0251 (EA, HIB). Native distribution range: Mountains of NE and E Tropical Africa to DR Congo, Cameroon.

***Scabiosacolumbaria* L.** – Habit: Herb. Habitat: Woodland, upland grassland, grass-moor rocky heathland, upper forest bushland, 2200–4000 m. Vouchers: Rono et al. SAJIT-PR 0135 (EA, HIB), Lisowski S 11788 (BR). Native distribution range: Europe to Iran and Arabian Peninsula, NW Africa, Tropical African Mountains.

*Valerianakilimandscharica Engl. – Habit: Herb or subshrub. Habitat: Upland moor, alpine zone, along streams, 2800–4500 m. Vouchers: Hedberg 1549 (EA), Siemens LA 2572 (EA), Adamson J 18226 (EA), 476 (K), Hedberg O 210, 961 (EA), Lugard C & Lugard EJ 2954 (EA), Tweedie DR 73, 119 (EA), Dale IR 3180 (EA, K), Battiscombe E 676 (EA), Lugard EJ 404 (K), Thomas 617 (EA). Native distribution range: E Tropical African Mountains.

***Valerianavolkensii* Engl.** – Habit: Herb. Habitat: Streamside, marshes, bamboo zone, upland forest and upland moor, 2300–3400 m. Vouchers: Irwin PH 313, Hedberg O 257 (S), Synge PM 1872 (S), Tweedie EM 892 (K), Kisalye N; Heist M van 401 (WAG), Gillett JB 18414 (BR), Tothill BH 2357 (EA), Dummer RA 3577 (K). Native distribution range: E Central & E Tropical African Mountains.

#### F55. CARYOPHYLLACEAE

6 Genera, 12 species

***Cerastiumafromontanum* T.C.E.Fr.** – Habit: Prostrate herb. Habitat: Short grassland, upland moor, alpine zone, 2300–4000 m. Vouchers: Friis I & Hansen OJ 2564 (EA), Tweedie 36 (EA), Bickford N 47 (EA), Adamson J 494 (EA), Lugard C 324 (K), Beentje HJ 1981 (EA, WAG), Gillett JB 18455 (K), Dale IR 3203 (EA, K). Native distribution range: South Sudan (Imotong Mountains) to Tanzania.

***Cerastiumlanceolatum* (Poir.) Volponi** – Habit: herb. Habitat: Forest edge, wet grassland, 1500–3600 m. Vouchers: Hamilton & Perrott 76/524 (EA). Native distribution range: Tropical & S Africa, Réunion, S India, Sri Lanka, S Malesia to New Guinea.

***Cerastiumoctandrum* Hochst. ex A.Rich.** – Habit: Herb. Habitat: Disturbed grassland, upland moor and bushland, 2500–4200 m. Vouchers: Rich A 58(EA), Hedberg O 188, 97(EA). Native distribution range: Cameroon to Eritrea and Tanzania.

^**EX**^***Corrigiolalitoralis* L.** – Habit: Prostrate herb. Habitat: Roadside, disturbed grounds, 2350–3600 m. Voucher: Mwangangi OM 448 (BR). Native distribution range: Europe, S Tropical & S Africa.

***Drymariacordata* (L.) Willd. ex Schult.** – Habit: Straggling soft herb. Habitat: Forest edges, roadsides, 1350–2500 m. Vouchers: Beentje HJ 1927 (EA), Kahuho SK 3 (EA). Native distribution range: Mexico to S Tropical America, Tropical & S Africa.

***Saginaabyssinica* Hochst. ex A.Rich.** – Habit: Herb. Habitat: Alpine belt, riverbank, upland moor, 2150–4250 m. Vouchers: Wesche K 1820, 1689 (K), Hedberg O 4548 (EA), Horu PH 364 (EA), Rauh W 591 (EA), Hedberg 870 (S), Lisowski S 80258 (BR). Native distribution range: Bioko to Ethiopia and N Tanzania.

***Saginaafroalpina* Hedberg** – Habit: Herb. Habitat: Upland moorland, 3150–4321 m. Voucher: Tweedie EM (1976) (REF). Native distribution range: Ethiopia to E Central & E Tropical Africa.

***Sileneburchellii* Otth** – Habit: Herb. Habitat: Upland grassland, forest, moorland, alpine zone, 1900–4050 m. Vouchers: Hedberg O 951 (EA, K, S), Tweedie 72 (EA), Jeaudui DR 116 (EA), Bickford NB 44 (EA), Lisowski S 80284 (BR). Native distribution range: South Africa.

^**EX**^***Silenegallica* L.** – Habit: Herb. Habitat: Roadside and in cultivation, 1960–3000 m. Vouchers: Tweedie EM (1976) (REF), Agnew ADQ (2013) (REF). Native distribution range: Macaronesia, W Europe to Mediterranean.

**Silenesyngei (Turrill) T.Harris & Goyder – Habit: Herb. Habitat: Outcrop rock, upland moor 3750 m. Vouchers: Synge PM 1912 (K, MO, NHMUK). Native distribution range: Uganda (Mt Elgon).

**^EX^*Stellariamedia* (L.) Vill.** – Habit: Trailing herb. Habitat: Montane forest floor, Introduced weed in cultivation, 1250–2520 m. Voucher: Wesche K 1692 (EA). Native distribution range: Temperate Eurasia, N & NE Tropical Africa.

***Stellariasennii* Chiov.** – Habit: Trailing herb. Habitat: Forest edges, path sides, 1750–3600 m. Voucher: Tweedie 107 (K). Native distribution range: Cameroon, Ethiopia to S Tropical Africa.

#### F56. CELASTRACEAE

5 Genera, 7 species

***Cathaedulis* (Vahl) Endl.** – Habit: Tree. Habitat: Evergreen forest, woodland on rocky hills, 1100–1800(–2400) m. Vouchers: Snowden JD 872 (EA), Eggeling WJ 2478 (BR), Snowden JD 966 (BR), Styles B 302 (BR). Native distribution range: Eritrea to S Africa, Yemen.

***Elaeodendronbuchananii* Loes.** – Habit: Shrub or tree. Habitat: Forest, 1070–2290 m. Vouchers: Jackson L 328, 318 (EA), Mathews PM 225 (EA), Dale IR 339 (EA). Native distribution range: Tropical Africa.

***Gymnosporiaarbutifolia* Loes.** – Habit: Shrub or small tree. Habitat: Woodland and forest, 1750–3000 m. Vouchers: Irwin PH 525 (BR), Dale IR U12 (BR). Native distribution range: Eritrea to E Central Tropical Africa, SW Arabian Peninsula.

***Gymnosporiaobscura* Loes.** – Habit: Shrub or small tree. Habitat: Riverine forest, forest margin, grassland, 2100–2550 m. Voucher: Lugard C 612 (EA). Native distribution range: Ethiopia to Burundi and N Tanzania.

***Gymnosporiasenegalensis* Loes.** – Habit: Shrub or tree. Habitat: Wet savanna and woodland, 1750–2400 m. Vouchers: Harrington GN 509 (EA), Snowden JD 839 (EA), Dale IR 790(EA), Tnysnell CG 2370 (EA), Rono et al. SAJIT-PR 0011, SAJIT-PR 0188 (EA, HIB). Native distribution range: Tropical & S Africa, Arabian Peninsula.

***Maytenusundata* (Thunb.) Blakelock** – Habit: Shrub or small tree. Habitat: woodland, forest, riverine forest, 1750–3150 m. Vouchers: Bridson D 78 (BR), Rono et al. SAJIT-PR 0044 (EA, HIB). Native distribution range: Tropical & S Africa, Comoros, Madagascar.

***Pristimeragoetzei* (Loes.) R.H.Archer** – Habit: Liana. Habitat: Moist forest, riverine forest, 90–3000 m. Voucher: St Clair Thompson in Eggeling 393 (EA). Native distribution range: Ethiopia to N Malawi.

#### F57. CLEOMACEAE

1 Genus, 2 species

***Cleomegynandra* L.** – Habit: Herb. Habitat: Disturbed grounds, cultivations, roadside, wet savanna, 1750–2250 m. Vouchers: Tweedie EM (1976) (REF), Agnew ADQ (2013) (REF). Native distribution range: Tropical & Subtropical Old World.

***Cleomemonophylla* L.** – Habit: Herb. Habitat: Disturbed grassland/woodland, 20–2100 m. Voucher: Tweedie 668 (EA). Native distribution range: Tropical & S Africa, Arabian Peninsula, India, Sri Lanka.

#### F58. COMBRETACEAE

2 Genera, 5 species

***Combretumcollinum* Fresen.** – Habit: Tree. Habitat: Wet savanna, 1750–2250 m. Vouchers: Dale IR 3098 (EA), Major C & Lugard EJ 524 (EA), Templer JC 8 (EA). Native distribution range: NE Tropical Africa.

**Combretumcollinumsubsp.binderianum (Kotschy) Okafa** – Habit: Tree. Habitat: Wooded grassland, 1100–2300 m. Voucher: Human observation sn (EA). Native distribution range: Tropical & S Africa.

**Combretumcollinumsubsp.elgonense (Exell) Okafa** – Habit: Tree. Habitat: Wooded grassland, 1100–2300 m. Vouchers: Bally 2481 (EA), Major EJ; Lugard C 524 (K). Native distribution range: Ethiopia to Zambia.

***Combretummolle* R.Br. ex G.Don** – Habit: Tree. Habitat: Forest, woodland, 1750–2250 m. Vouchers: Brown ES 755 (EA), Snowden JD 837, 1063 (EA), Dale IR (3105), Major C & Lugard EJ 286 (EA). Native distribution range: Tropical & S Africa, Arabian Peninsula.

***Terminalia mollis* M.A.Lawson** – Habit: Tree. Habitat: Forest, woodland, 1750–2170 m. Voucher: Tweedie EM (1976) (REF). Native distribution range: Tropical Africa.

#### F59. CONVOLVULACEAE

4 Genera, 14 species

***Astripomoeagrantii* (Rendle) Verdc.** – Habit: Herb. Habitat: Forest, grassland, bushland, 1750–2250 m. Voucher: Chater-Jack 125 (EA). Native distribution range: E Central & E Tropical Africa.

**Astripomoeamalvaceavar.parviflora (Rendle) Staples** – Habit: Sub-shrubby herb. Habitat: Forest, grassland, bushland, 1750–2250 m. Voucher: Tweedie EM (1976) (REF). Native distribution range: Tropical & S Africa.

***Convolvuluskilimandschari* Engl.** – Habit: Climber. Habitat: Upland forest, woodland, bamboo thickets, upland moor, 1800–3750 m. Vouchers: Hedberg 155 (EA), Tweedie EM 1786 (K), 1785 (K) 1585 (K), Wesche K 778 (EA), Tweedie 1585 (EA), Dummer RA 3585 (EA), Lienberg 1612 (EA), Rono et al. SAJIT-PR 0176 (EA, HIB). Native distribution range: Ethiopia to Tanzania.

***Cuscutakilimanjari* Oliv.** – Habit: Climber. Habitat: Forest, 1500–1020 m. Vouchers: Tweedie EM 801 (K), Dawkins 783 (EA), Snowden JD 854 (EA). Native distribution range: Ethiopia to S Africa, Madagascar.

***Cuscutaplaniflora* Ten.** – Habit: Twining herb. Habitat: Outcrop rock, 1800–2400 m. Voucher: Tweedie 2055 (K). Native distribution range: Macaronesia, Mediterranean to NW India, Eritrea to S Africa, Madagascar.

***Ipomoeabiflora* Pers.** – Habit: Twinning Herb. Habitat: Forest, grassland, bushlands, 1750–2250 m. Vouchers: Kisalye N; van Heist M 284 (K). Native distribution range: Tropical & Subtropical Old World.

***Ipomoeacairica* (L.) Sweet** – Habit: Twiner. Habitat: Forest, woodland, grassland, 1750–2250 m. Voucher: Tweedie EM (1976) (REF). Native distribution range: Tropical & S Africa, W Indian Ocean, Arabian Peninsula to Temperate E Asia

***Ipomoeafulvicaulis* Boiss. ex Hallier f.** – Habit: Herb. Habitat: Forest, outcrop rock, 1750–2250 m. Vouchers: Tweedie 583, 672 (EA). Native distribution range: Ethiopia to Botswana.

***Ipomoeahildebrandtii* Vatke** – Habit: Sub-woody shrublet. Habitat: Grassland, 150–2000 m. Vouchers: Human observation (1.351001 N 34.507378 E). Native distribution range: Ethiopia to Rwanda and Tanzania.

***Ipomoeakituiensis* Vatke** – Habit: Shrublet. Habitat: Forest, grassland, bushlands, 1750–2250 m. Voucher: Tweedie EM (1976) (REF). Native distribution range: Ethiopia to S Tropical Africa.

***Ipomoeaoenotherae* Hallier f.** – Habit: Erect or prostrate herb. Habitat: Seasonally moist grassland, outcrop rock, 990–2190 m. Vouchers: Tweedie EM (1976) (REF), Agnew ADQ (2013) (REF). Native distribution range: Eritrea to S Africa.

***Ipomoeapolymorpha* Roem. & Schult.** – Habit: Erect or prostrate herb. Habitat: Grassland and moist bushland, 1750–1920 m. Vouchers: Tweedie EM (1976) (REF), Agnew ADQ (2013) (REF). Native distribution range: Tropical Old World to Australia.

***Ipomoeatenuirostris* Choisy** – Habit: Twinning or prostrate herb. Habitat: Forest and woodland, 1650–2710 m. Vouchers: Irwin PH 146 (EA), Tweedie EM 350 (K). Native distribution range: Tropical Africa.

***Ipomoeawightii* Choisy** – Habit: Twinning or prostrate herb. Habitat: Upland grassland, montane forest edges, 1040–2400 m. Vouchers: Howard S, Irwin PH 150 (EA, MO), Tweedie EM 384 (K). Native distribution range: Ethiopia to S Africa, Madagascar, India to Bangladesh, Sri Lanka.

#### F60. CORNACEAE

2 Genera, 2 species

***Alangiumchinense* (Lour.) Harms** – Habit: Tree. Habitat: Upland forest, 750–2000 m. Vouchers: Katende T; Sheil D 697 (K), Snowden JD 1079 (EA). Native distribution range: Cameroon to SE Ethiopia and S Tropical Africa, Tropical & Subtropical Asia.

***Cornusvolkensii* Harms** – Habit: Tree. Habitat: Forest, 2250–3150 m. Vouchers: Eggeling WJ 2458 (MO), Eggeling 1050 (EA), George Taylor 3468 (NHMK), Rono et al. SAJIT-PR 0228 (EA, HIB). Native distribution range: South Sudan to S Tropical Africa

#### F61. CRASSULACEAE

4 Genera, 16 species

***Crassulaalata* (Viv.) A.Berger** – Habit: Herb. Habitat: Forest, shallow soils, 1350–2700 m. Voucher: Agnew ADQ (2013) (REF). Native distribution range: S & E Mediterranean to China (NW Yunnan).

**Crassulaalatasubsp.pharnaceoides (Fisch. & C.A.Mey.) Wickens & M.Bywater** – Habit: Herb. Habitat: Wet grassland, 1750–2250 m. Voucher: Tweedie EM (1976) (REF). Native distribution range: Cameroon, Ethiopia to S Africa and Arabian Peninsula.

***Crassulaalba* Forssk.** – Habit: Herb. Habitat: Forest, rocky grassland, 1500–4000 m. Voucher: Rono et al. SAJIT-PR 0094 (EA, HIB). Native distribution range: Eritrea to S Africa, SW Arabian Peninsula.

***Crassulaalsinoides* Engl.** – Habit: Sprawling or prostrate herb. Habitat: Montane forests, 1475–3600 m. Vouchers: Tweedie EM 1807B (K), Alan C Hamilton 6601 (MO), Rono et al. SAJIT-PR 0266 (EA, HIB). Native distribution range: Tropical & S Africa, SW Arabian Peninsula, Madagascar.

***Crassulagranvikii* Mildbr.** – Habit: Herb. Habitat: Montane forests, 1650–4500 m. Vouchers: Lye 5721 (EA), Lugard EJ; Lugard C 422 (K), Beentje HJ 1993 (WAG), Rono et al. SAJIT-PR 0025 (EA, HIB), PK Rwaburindore 447 (MO). Native distribution range: Eritrea to N Malawi, to Yemen.

***Crassularhodesica* (Merxm.) Wickens & M.Bywater** – Habit: Herb. Habitat: Forest clearings, rocky grassland, 1200–2378 m. Voucher: Human observation sn (EA). Native distribution range: Kenya to Namibia.

***Crassulaschimperi* C.A.Mey.** – Habit: Herb. Habitat: Upper Forest edge, 3000–4100 m. Vouchers: Tweedie 2767, Wood GH (K), Tweedie EM 21 (MO), Rono et al. SAJIT-PR 0092 (EA, HIB). Native distribution range: Tropical & S Africa to Himalaya.

**Crassulaschimperisubsp.phyturus (Mildbr.) R.Fern.** – Habit: Herb. Habitat: Forest on moist rocks in crevices, 1050–2400 m. Vouchers: Hedberg 4492 (EA), Tweedie 2767 (EA), Kisalye N et al. 04 (K), Rono et al. SAJIT-PR 0138 (EA, HIB). Native distribution range: NE & E Tropical Africa, and Yemen.

***Kalanchoecrenata* (Andrews) Haw.** – Habit: Herb. Habitat: Forest, bushland, grassland, moist rocks, 1650–2300 m. Vouchers: Snowden JD 1032 (EA), Tengecho B 383 (EA), Jack C 164 (EA). Native distribution range: Tropical & S Africa, Arabian Peninsula.

***Kalanchoedensiflora* Rolfe** – Habit: Herb. Habitat: Disturbed places, woodland, forest, upper foerest edge, 1750–3150 m. Vouchers: Bamps PRJ 6495 (EA), (BR), Symes YE 246 (EA), Jack C 398 (EA), Rono et al. SAJIT-PR 0221 (EA, HIB). Native distribution range: Ethiopia to E DR Congo and Tanzania.

**^EX^*Kalanchoeintegra* Kuntze** – Habit: Herb. Habitat: Forest, grassland, 1750–2250 m. Voucher: Tweedie EM (1976) (REF). Native distribution range: Tropical & Subtropical Asia.

***Kalanchoelanceolata* Pers.** – Habit: Herb. Habitat: Open woodland, wooded grassland, 950–2100 m. Voucher: Agnew ADQ (2013) (REF). Native distribution range: Tropical & S Africa, SW Madagascar, SW Arabian Peninsula, Indian Subcontinent.

***Kalanchoeprittwitzii* Engl.** – Habit: Herb. Habitat: Forest, outcrop rock, 1800–2400 m. Vouchers: Jack C 396 (EA), Snowden JD 943 (EA), Symes YE 245 (EA), Lugard C & Lugard EJ 115 (EA). Native distribution range: E Ethiopia to E Central & E Tropical Africa.

***Sedummeyeri-johannis* Engl.** – Habit: Herb. Habitat: Upland forest, heathland, 3200–4300 m. Vouchers: Thomas AS 2703 (EA), Dale U 848 (EA). Native distribution range: E Central & E Tropical Africa.

***Sedumruwenzoriense* Baker f.** – Habit: Succulent herb. Habitat: Upland heath and upland moor, in rock crevices in heath zone, 2400–4300 m. Vouchers: Wood GH 124 (EA), Mwangangi OM 3871, Mwangangi OM 318 (WAG, MO), Rono et al. SAJIT-PR 0132 (EA, HIB). Native distribution range: Ethiopia to Rwanda.

***Umbilicus botryoides* Hochst. ex A.Rich.** – Habit: Herb. Habitat: Rock crevices montane forest, 2250–3900 m. Vouchers: Rose 10216 (EA), Wesche K 588 (EA). Native distribution range: Cameroon, Eritrea to Tanzania.

#### F62. CUCURBITACEAE

9 Genera, 13 species

***Cocciniaadoensis* (Hochst. ex A.Rich.) Cogn.** – Habit: Climber herb. Habitat: Grassland, moist forest, woodland, 1600–2300 m. Vouchers: Bally 251 (EA), Polhill 400 (S), Norman EM 225 (K), Taylor G 3787 (S). Native distribution range: Ghana to Eritrea and S Africa.

***Cucumisficifolius* A.Rich.** – Habit: Herb. Habitat: Upland grassland roadsides, 1200–2800 m. Voucher: Snowden JD 1061 (EA). Native distribution range: Mauritania to Ethiopia and Tanzania, SW Arabian Peninsula.

***Cucumismaderaspatanus* L.** – Habit: climber or trailer Herb. Habitat: Riverine margins, woodland, bushland, upland forest, 0–1800 m. Vouchers: Tweedie EM 2247 (K, BR). Native distribution range: Tropical & Subtropical Old World.

***Lagenariaabyssinica* (Hook.f.) C.Jeffrey** – Habit: Climber herb. Habitat: Bushland, montane forest, 900–3000 m. Vouchers: Mwangangi OM 450 (BR), Rono et al. SAJIT-PR 0169 (EA, HIB). Native distribution range: Ethiopia to DR Congo and Tanzania.

***Momordicacissoides* Planch. ex Benth.** – Habit: Climber herb. Habitat: Riverine and rainforest, 1190–1830 m. Voucher: Snowden JD 1040 (EA). Native distribution range: Tropical Africa.

***Momordicafoetida* Schumach.** – Habit: Climber herb. Habitat: Forest edges, old cultivations, disturbed regions, 1200–3000 m. Vouchers: Tweedie 1097 (K), Cheseny CM 40 (BR), Lind EM 114 (BR), Rono et al. SAJIT-PR 0075, SAJIT-PR 0173 (EA, HIB). Native distribution range: Tropical & S Africa.

***Momordicafriesiorum* (Harms) C.Jeffrey** – Habit: Climber herb. Habitat: Upland Forest edges, wet bushlands, 1700–2850 m. Voucher: Tweedie EM 915 (K). Native distribution range: Ethiopia to N Malawi.

***Oreosyceafricana* Hook.f.** – Habit: climber or trailer Herb. Habitat: Bamboo Forest, swamps, grassland edges, 1500–3000 m. Voucher: Tweedie 1190 (EA). Native distribution range: Bioko to Ethiopia and South Africa, Madagascar.

***Peponiumelgonense* N.Wei, G.W.Hu & Q.F.Wang** – Habit: Vine. Habitat: Open grassland, moist rock wall of a steep cliff, 2565 m. Vouchers: Rono et al. SAJIT-PR 0121, SAJIT-PR 0152 (EA, HIB). Native distribution range: Kenya.

**^EX^*Sicyospolyacanthos* Cogn.** – Habit: climber or trailer Herb. Habitat: Woodland, 1750–2400 m. Voucher: Tweedie DR 4133 (WAG). Native distribution range: Bolivia to Brazil and N Argentina.

***Trochomeriamacrocarpa* Harv.** – Habit: Trailing or climbing herb. Habitat: Tree grassland, woodland, 1000–2550 m. Vouchers: Lugard E 143 (K), Tweedie EM 1101 (K), Polhill 414 (BR). Native distribution range: Tropical & S Africa.

***Zehneriaminutiflora* (Cogn.) C.Jeffrey** – Habit: Prostrate herb. Habitat: Upland moist forest edges, bamboo thickets, grassland, 1100–3350 m. Vouchers: Tweedie EM (1976) (REF), Agnew ADQ (2013) (REF). Native distribution range: Tropical Africa.

***Zehneriascabra* Sond.** – Habit: climber or trailer Herb. Habitat: Forest edges, old cultivated bushlands, wet savanna, riverine forest, grass thickets, 1000–3350 m. Vouchers: Tweedie EM 1384 (BR), Tweedie DR 4073 (WAG), Tweedie EM 1384 (K), Rono et al. SAJIT-PR 0030, SAJIT-PR 0079 (EA, HIB). Native distribution range: Tropical & Subtropical Old World.

#### F63. EBENACEAE

2 Genera, 3 species

***Diospyrosabyssinica* (Hiern) F.White** – Habit: Shrub. Habitat: Montane Forest, open bushland woodland, 2250–3000 m. Voucher: Tweedie EM (1976) (REF). Native distribution range: Tropical Africa.

***Eucleadivinorum* Hiern** – Habit: Shrub or small tree. Habitat: Grassland, open bushland, montane forest, 1750–2700 m. Vouchers: Jackson THE 329a (MO), Taiti S 612 (BR), Rono et al. SAJIT-PR 0002 (EA, HIB). Native distribution range: Ethiopia to S Africa.

**Euclearacemosasubsp.schimperi (A.DC.) F.White** – Habit: Shrub or small tree. Habitat: Woodland, 1750–2400 m. Voucher: Tweedie EM (1976) (REF). Native distribution range: Egypt to S Africa, S Arabian Peninsula, Comoros.

#### F64. ERICACEAE

2 Genera, 7 species

***Agaristasalicifolia* G.Don** – Habit: Shrub or tree. Habitat: Woodland, moist forest, heath zones, 1750–3150 m. Vouchers: Dummer RA 3619 (US), St Clair-Thompson GW 2/40 (BR), Wesche K 944 (EA), Rono et al. SAJIT-PR 0129 (EA, HIB). Native distribution range: Tropical Africa, W Indian Ocean

***Ericaarborea* L.** – Habit: Shrub or tree. Habitat: Heath, woodland, alpine, 1800–3900 m. Vouchers: Tweedie DR 2066 (EA), Wesche K 98 (EA), Kokwaro JO 2547 (EA), Taylor G de C 3448 (EA), Watere 10513 (EA), Gardner HM 2253 (EA), Leyard WC 2964 (EA), Tothill BH 2293 (EA), Rono et al. SAJIT-PR 0151 (EA, HIB). Native distribution range: Mediterranean to W Transcaucasus, Tropical African Mountains, Arabian Peninsula.

***Ericafilago* (Alm & T.C.E.Fr.) Beentje** – Habit: Shrub. Habitat: Disturbed or burnt areas in alpine, heath, moorland zone, 2000–4321 m. Vouchers: Wesche K 124, 854 (EA), Major ES; Lugard C 483 (K), Adamson J 485 (K), Hedberg O 874 (K), Dale IR 3096 (K), Dale IR 3184 (K), Dummer RA 3355 (US), Thomas AS 606 (BR). Native distribution range: Kenya to N Malawi.

***Ericamannii* (Hook.f.) Beentje** – Habit: Shrub or tree. Habitat: Upper forest edge, moorland, 3150–4050 m. Vouchers: Tweedie 1154 (BR, K), Hedberg O 850 (K), Synge PM S986 (BR) Native distribution range: Nigeria to Cameroon, Bioko, Uganda to S Tropical Africa.

***Ericasilvatica* (Engl.) Beentje** – Habit: Shrub. Habitat: Short grasslands, moorland, heath zones, bamboo clearing, forest edges, 1650–4321 m. Vouchers: Wesche K 1814 (EA), Dummer RA 3503 (K), 3509 (US), Hedberg KO 4555 (BR), 4535 (EA). Native distribution range: Tropical African Mountains.

***Ericatrimera* (Engl.) Beentje** – Habit: Shrub or small tree. Habitat: Moorland, rain forest, 3150–4321 m. Voucher: Taylor G 3524 (BR). Native distribution range: E Central Tropical Africa (Ruwenzori Mountains).

**Ericatrimerasubsp.elgonensis (Mildbr.) Beentje – Habit: Shrub or small tree. Habitat: Moorland, 3450–3800 m. Vouchers: Saundy & Hancock 63 (EA), Hedberg 4529 (EA), Lye & Pocs 23079 (EA), Lindblom GSN (BR), Wesche K 1014 (EA). Native distribution range: E Tropical Africa (Mt Elgon).

#### F65. EUPHORBIACEAE

8 Genera, 29 species

***Acalyphapolymorpha* Müll.Arg.** – Habit: Herb. Habitat: Disturbed or burnt grassland, open woodland, 1700–2300 m. Voucher: James (EA). Native distribution range: Central & E Tropical Africa.

***Acalyphapsilostachya* Hochst. ex A.Rich.** – Habit: Herb or subshrub. Habitat: Moist rain forest, grassland, rocky woodland, 520–3050 m. Vouchers: Dawkins HC 779 (EA, BR). Native distribution range: Cameroon to Ethiopia and S Tropical Africa.

***Acalyphavolkensii* Pax** – Habit: Herb or subshrub. Habitat: Upland grassland, forest undergrowth, bushland, 800–2700 m. Voucher: Tweedie EM (1976) (REF), Agnew ADQ (2013) (REF). Native distribution range: Ethiopia to Burundi.

***Clutiaabyssinica* Jaub. & Spach** – Habit: Shrub or small tree. Habitat: Upland forest and edges, bushlands, wooded grassland, riverine thickets, 1500–3150 m. Vouchers: Beentje HJ 2062 (WAG), Mwangangi OM 335 (BR), Cheseny CM 15 (RSA), Katende AB 3604 (MO), Rono et al. SAJIT-PR 0208, SAJIT-PR 0074 (EA, HIB). Native distribution range: Ethiopia to S Africa.

**Clutiaabyssinicavar.pedicellaris (Pax) Pax** – Habit: Herb, shrub or small tree. Habitat: Forest, woodland, 1750–3000 m. Voucher: Tweedie EM (1976) (REF). Native distribution range: Ethiopia to Malawi.

***Clutiakilimandscharica* Engl.** – Habit: Shrub. Habitat: Forest, upper forest edge, moorland, 2250–3600 m. Vouchers: Thomas AS 656 (EA), Tothill BH 2303 (EA), Eggeling 2461 (EA). Native distribution range: Somalia to Zimbabwe.

**Clutialanceolatasubsp.robusta (Pax) M.G.Gilbert** – Habit: Shrub. Habitat: Forest, upper forest edge, moorland, 2400–3350 m. Vouchers: Kokwaro JO 2439 (BR), Taylor PG 3621 (BR). Native distribution range: NE Tropical Africa, Arabian Peninsula.

***Crotonmacrostachyus* Hochst. ex Delile** – Habit: Shrub or tree. Habitat: Woodland, 1750–2400 m. Vouchers: Snowden JD 869, 870 (A, EA, BR), Rono et al. SAJIT-PR 0076 (EA, HIB). Native distribution range: Tropical Africa.

***Euphorbiabrevicornu* Pax** – Habit: Herb. Habitat: Moist open forest, 2200–3600 m. Vouchers: Gillett 18429 (EA, BR), Lugard EJ; Lugard C 380 (K), Rono et al. SAJIT-PR 0220 (EA, HIB). Native distribution range: Kenya, Uganda.

***Euphorbiabrunellii* Chiov.** – Habit: hairless from a fleshy tuber with apex at soil level. Habitat: Grassland, 1100–2500 m. Vouchers: Tweedie 355 (EA). Native distribution range: Ethiopia to Uganda.

***Euphorbiacrotonoides* Boiss.** – Habit: Herb. Habitat: Disturbed grounds, outcrop rock, open woodland, forest edge, 800–2400 m. Voucher: Rono et al. SAJIT-PR 0077 (EA, HIB). Native distribution range: Ethiopia to S Africa.

***Euphorbiadepauperata* Hochst. ex A.Rich.** – Habit: Shrub or herb. Habitat: Grassland, 1900–3200 m. Voucher: Tweedie DR 2521 (WAG). Native distribution range: Tropical Africa.

***Euphorbiaengleri* Pax** – Habit: Herb. Habitat: Montane rain forest, 1500–2800 m. Vouchers: Mwangangi OM & Kanuri F. 342 (BR), Tweedie 494 (K), Dale IR U849 (K), Hedberg O 93 (K), Wesche K 1313 (K). Native distribution range: E Tropical Africa.

***Euphorbiaheterospina* S.Carter** – Habit: Shrub. Habitat: Woodland, rock slopes, 550–1600 m. Voucher: Snowden JD 1046 (EA). Native distribution range: Kenya, Uganda.

^**EX**^***Euphorbiahirta* L.** – Habit: Herb. Habitat: Roadsides, cultivation, forest, 1750–2250 m. Voucher: Tweedie EM (1976) (REF). Native distribution range: Tropical & Subtropical America.

***Euphorbiainaequilatera* Sond.** – Habit: Herb. Habitat: Forest, wooded grassland, 1750–2500 m. Voucher: Hedberg 41 (EA). Native distribution range: Africa to Pakistan.

**^EX^*Euphorbiamurielii* N.E.Br.** – Habit: Tree. Habitat: Forest, rocky slopes, open wooded grassland, 1750–2250 m. Voucher: Tweedie EM (1976) (REF). Native distribution range: Sudan.

**^EX^*Euphorbiaprostrata* Aiton** – Habit: Herb. Habitat: Disturbed lawns, grasslands, roadside, 1750–2250 m. Vouchers: Tweedie EM (1976) (REF), Agnew ADQ (2013) (REF). Native distribution range: Central & S USA to Tropical & Subtropical America.

^**EX**^***Euphorbiarubella* Pax** – Habit: Tuber. Habitat: Wet savanna, 1750–2250 m. Voucher: Tweedie EM (1976) (REF). Native distribution range: Ethiopia.

***Euphorbiaschimperiana* Scheele** – Habit: Herb. Habitat: Grassland, montane forest edge, roadside, 1600–3760 m. Vouchers: Tweedie EM (1976) (REF), Agnew ADQ (2013) (REF). Native distribution range: Tropical Africa, Arabian Peninsula.

***Euphorbiasystyloides* Pax** – Habit: Herb. Habitat: wet savanna, 1750–2250 m. Voucher: Tweedie EM (1976) (REF). Native distribution range: Uganda to S Tropical Africa.

*Euphorbiaugandensis Pax & K.Hoffm. – Habit: Woody herb or Shrub. Habitat: Bamboo zone, forest clearing, 1980–3350 m. Vouchers: Tothill BH 2275 (EA), Tweedie EM 2521 (K). Native distribution range: SW Uganda to N Malawi.

***Euphorbiawellbyi* N.E.Br.** – Habit: Herb. Habitat: Upper montane forest edge, moorland, 2300–4000 m. Vouchers: Snowden JD 462 (EA), VC Hedberg E 3 (EA), Hedberg 214 (EA). Native distribution range: Ethiopia to Tanzania.

**Euphorbiawellbyivar.glabra S.Carter** – Habit: Herb. Habitat: Heathland, forest edge, 3200–4080 m. Vouchers: Hedberg O 4550 (K), Lisowski S 12913 (BR), Snowden JD 462 (EA), Liebenberg 1625 (EA), Wood G 150 (EA). Native distribution range: South Sudan to Tanzania.

***Macarangacapensis* (Baill.) Sim** – Habit: Tree. Habitat: Evergreen Forest, 10–3050 m. Vouchers: Snowden JD 815 (EA). Native distribution range: Gabon to Kenya and S Africa.

***Macarangakilimandscharica* Pax** – Habit: Tree. Habitat: Forest, 2250–3000 m. Voucher: Snowden JD 906 (BR). Native distribution range: Ethiopia to S Tropical Africa.

***Neoboutoniamacrocalyx* Pax** – Habit: Tree. Habitat: Forest, 2250–3000 m. Vouchers: Styles B 306 (BR), Synge PM S789 (BR). Native distribution range: Cameroon to Uganda and S Tropical Africa.

**^EX^*Ricinuscommunis* L.** – Habit: Herb or subshrub. Habitat: Woodland edges, 900–2000 m. Vouchers: Padwa JH 40 (EA), Jack C 184 (EA). Native distribution range: NE Tropical Africa.

***Tragiabrevipes* Pax** – Habit: Herb or shrub. Habitat: Forest, woodland, wooded grassland, 600–2600 m. Vouchers: Tweedie EM (1976) (REF), Agnew ADQ (2013) (REF). Native distribution range: Cameroon to Somalia and Zimbabwe.

#### F66. FABACEAE

48 Genera, 168 species

***Adenocarpusmannii* Hook.f.** – Habit: Shrub. Habitat: Montane forest, grassland, rock outcrop on moorland, 2250–3000 m. Voucher: Tweedie EM (1976) (REF). Native distribution range: Tropical African Mountains to Namibia.

***Aeschynomeneabyssinica* Vatke** – Habit: Shrub or herb. Habitat: Grassland, scattered grassland, stream sides, swamp edges, bamboo edges, 1000–3000 m. Vouchers: Hedberg O 54 (UPS), Tweedie 1890 (EA), Rono et al. SAJIT-PR 0053 (EA, HIB). Native distribution range: Tropical Africa.

***Aeschynomeneschimperi* Hochst. ex A.Rich.** – Habit: Shrubby herb or shrub. Habitat: Grassland, marshes, riverside, 670–2500 m. Voucher: Agnew ADQ (2013) (REF). Native distribution range: Tropical Africa.

***Afroamphicaafricana* (Hook.f.) H.Ohashi & K.Ohashi** – Habit: Climber herb. Habitat: Montane Forest edge, woodland, 1750–3000 m. Voucher: Mwangangi OM 485 (BR). Native distribution range: Nigeria to Ethiopia and Malawi.

**Albiziaamarasubsp.sericocephala Brenan** – Habit: Tree. Habitat: Woodland, wooded grassland, 1750–2250 m. Voucher: Tweedie EM (1976) (REF). Native distribution range: Eritrea to S Africa.

***Albiziagrandibracteata* Taub.** – Habit: Tree. Habitat: Forest, riverine forest, grassland, 1750–2250 m. Voucher: Bally 2498 (EA). Native distribution range: Ethiopia to N Tanzania.

***Albiziagummifera* C.A.Sm.** – Habit: Tree. Habitat: Forest, riverine forest, forest clearing, 1750–2440 m. Vouchers: Lugard 511 (EA), Mrs C Jack 434 (BR). Native distribution range: Tropical Africa, Central Madagascar.

***Alysicarpusglumaceus* (Vahl) DC.** – Habit: Herb. Habitat: Flooded grassland, woodland, 1000–2400 m. Voucher: Tweedie EM 907 (K). Native distribution range: Tropical & S Africa, Arabian Peninsula.

**Alysicarpusrugosussubsp.perennirufus J.Léonard** – Habit: Herb. Habitat: Flooded grassland, 940–2500 m. Voucher: Tweedie 907 (EA). Native distribution range: Central African Republic to Eritrea and S Africa.

***Angylocalyxbraunii* Harms** – Habit: Tree. Habitat: Understory of montane forest, riparian forest, 800 m. Voucher: Lugard C & Lugard EJ 334 (EA). Native distribution range: E Tropical Africa. (Kenya, Tanzania)

***Antopetitiaabyssinica* A.Rich.** – Habit: Herb. Habitat: Grassland, moorland, open bush land, 1700–3500 m. Voucher: Rono et al. SAJIT-PR 0270 (EA, HIB). Native distribution range: Tropical Africa.

***Argyrolobiumfischeri* Taub.** – Habit: Herb or subshrub. Habitat: Forest, bushland, grassland, 1700–2700 m. Voucher: Gerh Lindblom sn (S). Native distribution range: South Sudan to S Tropical Africa.

**Argyrolobiumrupestresubsp.kilimandscharicum (Taub.) Polhill** – Habit: Herb. Habitat: Grassland, forest 2250–3300 m. Voucher: Agnew ADQ (2013) (REF). Native distribution range: South Sudan to E Tropical Africa

**Astragalusatropilosulussubsp.burkeanus (Benth. ex Harv.) J.B.Gillett** – Habit: Herb. Habitat: Grassland, open bushland, forest margin, moorland, 3900 m. Voucher: Lugard 334 (EA). Native distribution range: Ethiopia to S Africa, SW Arabian Peninsula.

^**EX**^***Biancaeadecapetala* (Roth) O.Deg.** – Habit: Shrub. Habitat: Rainforest, tree grassland, bushlands, 1750–2400 m. Voucher: Lugard 298 (EA). Native distribution range: India to S Central Japan.

***Calpurniaaurea* (Aiton) Benth.** – Habit: Shrub or tree. Habitat: Montane Forest, 1300–2250 m. Voucher: Tweedie 1584 (EA). Native distribution range: Central African Republic to Eritrea and S Africa.

***Chamaecristafalcinella* (Oliver) Lock** – Habit: Herb. Habitat: Wooded or bushy grassland, 1390–2650 m. Voucher: Agnew ADQ (2013) (REF). Native distribution range: E Central & E Tropical Africa.

**Chamaecristafalcinellavar.parviflora (Steyaert) Lock** – Habit: Herb. Habitat: Grassland, forest, 1750–2250 m. Voucher: Tweedie EM (1976) (REF). Native distribution range: Kenya to Namibia.

***Chamaecristakirkii* Standl.** – Habit: Herb. Habitat: Seasonally flooding grassland, 1600–2500 m. Voucher: Agnew ADQ (2013) (REF). Native distribution range: Tropical Africa.

***Chamaecristamimosoides* (L.) Greene** – Habit: Herb or shrub. Habitat: Forest clearing, forest edge, grassland wooded grassland, 1750–2400 m. Voucher: Tweedie 4095 (K). Native distribution range: Tropical & S Africa, Tropical Asia to N Australia.

***Chamaecristastricta* E.Mey.** – Habit: Herb. Habitat: Disturbed and pusture grounds, 1750–2250 m. Voucher: Agnew ADQ (2013) (REF). Native distribution range: Kenya to S Africa, N & Central Madagascar.

***Chamaecristausambarensis* Standl.** – Habit: Herb. Habitat: Upland forest, grassland, near rocks and on roadsides, 1800–2590 m. Voucher: Mwangangi OM 481 (BR). Native distribution range: E Tropical Africa.

***Chamaecristawittei* (Ghesq.) Lock** – Habit: Herb. Habitat: Upland grassland, bushland, 1740–2590 m. Voucher: Humphreys 1123 (EA). Native distribution range: Cameroon to Eritrea and Mozambique.

***Coluteaabyssinica* Kunth & C.D.Bouché** – Habit: Shrub. Habitat: Montane grassland, forest margin, bushlands, 2000–2800 m. Vouchers: Tweedie 859 (K), Rono et al. SAJIT-PR 0241 (EA, HIB). Native distribution range: Rwanda to NE & E Tropical Africa.

***Craibiabrownii* Dunn** – Habit: Tree. Habitat: Forest, riverine, 1100–2200 m. Vouchers: Dale IR 3241 (BR), Jackson THE 377 (BR), Dale IR MA-261214-1 (MA). Native distribution range: South Sudan to Zambia.

***Crotalariaagatiflora* Schweinf. ex Engl.** – Habit: Shrub. Habitat: Forest, woodland, shrubland, 1170–2100 m. Vouchers: Taylor G 3857 (MO), Rono et al. SAJIT-PR 0083 (EA, HIB). Native distribution range: Ethiopia to S Tropical Africa.

*Crotalariaagatiflorasubsp.engleri (Harms ex Baker f.) Polhill – Habit: Shrub or small tree. Habitat: Forest, wet savanna, 2300–3500 m. Vouchers: Tweedie EM (1976) (REF), Agnew ADQ (2013) (REF). Native distribution range: Central Kenya to N Tanzania.

**Crotalariaagatiflorasubsp.imperialis (Taub.) Polhill** – Habit: Woody herb or shrub. Habitat: Grassland, bushlands, 2000–2300 m. Voucher: Agnew ADQ (2013) (REF). Native distribution range: Ethiopia to E DR Congo.

***Crotalariaanthyllopsis* Welw. ex Baker** – Habit: Herb. Habitat: Forest, rocky or wooded grasslands, 1200–2400 m. Vouchers: Tweedie EM (1976) (REF), Agnew ADQ (2013) (REF). Native distribution range: Tropical Africa to South Africa.

***Crotalariabrevidens* Benth.** – Habit: Herb. Habitat: Woodland, grassland, bushland, 1500–3000 m. Vouchers: Tweedie EM (1976) (REF), Agnew ADQ (2013) (REF). Native distribution range: Nigeria to NE & E Tropical Africa.

***Crotalariacephalotes* Steud. ex A.Rich.** – Habit: Herb. Habitat: Open grassland, woodland, 1750–2250 m. Voucher: Symes YE 232 (MO). Native distribution range: Tropical Africa.

***Crotalariachrysochlora* Baker f. ex Harms** – Habit: Herb. Habitat: Grassland, rocky disturbed places, 1700–2900 m. Voucher: Agnew ADQ (2013) (REF). Native distribution range: Cameroon to Kenya and S Tropical Africa.

***Crotalariacleomifolia* Welw. ex Baker** – Habit: Herb or shrub. Habitat: Forest, grassland, bushland, 1150–2600 m. Voucher: Agnew ADQ (2013) (REF). Native distribution range: Tropical Africa, Madagascar.

***Crotalariacylindrica* A.Rich.** – Habit: Herb. Habitat: Montane grassland, moorland, 2000–3550 m. Vouchers: Tweedie 1418, 2088 (K). Native distribution range: Eritrea to Kenya.

***Crotalariadeserticola* Taub. ex Baker f.** – Habit: Herb. Habitat: Woodland, grassland, 1750–2250 m. Voucher: Tweedie EM (1976) (REF). Native distribution range: Ethiopia to S Tropical Africa.

***Crotalariadewildemaniana* R.Wilczek** – Habit: Herb. Habitat: Grassland, woodland, 1750–2400 m. Vouchers: Irwin PH 116 (EA, K), Tweedie EM 922 (MO). Native distribution range: Cameroon to E Tropical Africa.

***Crotalariaglauca* Willd.** – Habit: Herb. Habitat: Grassland, 1150–2300 m. Vouchers: Tweedie EM (1976) (REF), Agnew ADQ (2013) (REF). Native distribution range: Tropical Africa.

***Crotalariahyssopifolia* Klotzsch** – Habit: Herb. Habitat: Woodland, roadside, outcrop rock, 1750–2400 m. Vouchers: Tweedie 188. Native distribution range: Tropical Africa.

**Crotalariaincanasubsp.purpurascens (Lam.) Milne-Redh.** – Habit: Herb. Habitat: Grassland, bushland, 1250–2600 m. Vouchers: Tweedie EM (1976) (REF), Agnew ADQ (2013) (REF). Native distribution range: Tropical Africa, Madagascar, Arabian Peninsula, SW India.

***Crotalarialebrunii* Baker f.** – Habit: Shrub. Habitat: Streamside, clearings and forest edge, 2200–2750 m. Voucher: Rono et al. SAJIT-PR 0177 (EA, HIB). Native distribution range: Eastern DR Congo to Central Kenya.

***Crotalariakaragwensis* Taub.** – Habit: herb. Habitat: Open and wooded grasslands, bushlands, forest 1600–2300 m. Vouchers: Lugard EJ 197 (K), Lugard EJ; Lugard C 530 (K). Native distribution range: Cameroon to Ethiopia and Tanzania.

***Crotalariakeniensis* Baker f.** – Habit: Herb or shrub. Habitat: Streamside, forest edges, bushland, 1700–3200 m. Vouchers: Thomas AS 463 (MO), Mwangangi OM 465 (MO). Native distribution range: SW Ethiopia to Tanzania.

***Crotalarialaburnifolia* L.** – Habit: Herb or shrub. Habitat: Forest, grassland, 1750–2250 m. Voucher: Tweedie EM (1976) (REF). Native distribution range: Tropical & S Africa, Tropical Asia, N Australia.

***Crotalarialachnocarpoides* Engl.** – Habit: Bushy herb or small shrub. Habitat: Montane grassland, bushland, 1300–3000 m. Vouchers: Snowden JD 956 (MO), Rono et al. SAJIT-PR 0080 (EA, HIB). Native distribution range: Ethiopia to S Tropical Africa.

***Crotalarialachnophora* A.Rich.** – Habit: Herb or shrub. Habitat: Wooded grasslands, roadside, 1500–2200 m. Voucher: Agnew ADQ (2013) (REF). Native distribution range: Tropical Africa.

***Crotalarianatalitia* Meisn.** – Habit: Herb or small shrub. Habitat: Forest edges, bushland, and wooded open grassland, 1600–3000 m. Vouchers: Tweedie EM 1355 (MO), Snowden JD 932 (MO). Native distribution range: Tropical & S Africa, SW Arabian Peninsula.

***Crotalariapetitiana* (A.Rich.) Walp.** – Habit: Herb. Habitat: Grassland, bushland, 700–2200 m. Voucher: Agnew ADQ (2013) (REF). Native distribution range: NE Tropical Africa to N Tanzania.

***Crotalariaquartiniana* A.Rich.** – Habit: Herb. Habitat: Rare in forest edges, 2340–2650 m. Vouchers: Tweedie EM 1711 (K), Irwin PH 133A (BR). Native distribution range: Tropical Africa, Arabian Peninsula.

***Crotalariarecta* Steud. ex A.Rich.** – Habit: Herb or shrub. Habitat: Grassland, swamp margins, cultivated grounds, woodland, 850–2600 m. Vouchers: Tweedie EM 1317 (MO), Snowden JD 931 (MO). Native distribution range: Tropical & S Africa.

***Crotalariaspinosa* Hochst.** – Habit: Herb or shrub. Habitat: Wet savanna, woodland, 1750–2400 m. Voucher: Tweedie EM (1976) (REF). Native distribution range: Tropical Africa, Arabian Peninsula.

***Crotalariavallicola* Baker f.** – Habit: Herb. Habitat: Grassland, bushland, 1300–2500 m. Voucher: Irwin PH 118 (MO). Native distribution range: Sudan to Tanzania.

***Crotalariavatkeana* Engl.** – Habit: Herb. Habitat: Forest, grassland, and bushland, 2000–3300 m. Voucher: Tweedie EM 535 (MO). Native distribution range: Ethiopia to N Tanzania.

***Dalbergialactea* Vatke** – Habit: Small tree or shrub. Habitat: Forest margin, riverine forest, bushland, grassland, 1750–2250 m. Voucher: Snowden JD 492 (NHMUK). Native distribution range: Tropical Africa.

***Dolichoscompressus* R.Wilczek** – Habit: herb. Habitat: Wooded grasslands, bushlands, 1000–2000 m. Vouchers: Tweedie EM (1976) (REF), Agnew ADQ (2013) (REF). Native distribution range: S Sudan to Kenya.

**Dolichossericeussubsp.formosus (Hochst. ex A.Rich.) Verdc.** – Habit: Climbing herb. Habitat: Grassland, 1750–2400 m. Vouchers: Tweedie 1930 (EA), Tothill BH 2699 (EA), Tweedie EM 241 (K). Native distribution range: Eritrea to E Tropical Africa, Angola.

**Dolichossericeussubsp.pseudofalcatus Verdc.** – Habit: Climbing or prostrate herb. Habitat: Montane Forest edges, clearings, woodland, grassland, 1150–2780 m. Voucher: Lugard 89 (EA). Native distribution range: E Tropical Africa to DR Congo.

***Dumasiavillosa* DC.** – Habit: Climber herb or shrub. Habitat: Wet forest edge, woodland, 1800–2550 m. Vouchers: Tweedie EM (1976) (REF), Agnew ADQ (2013) (REF). Native distribution range: Tropical & Subtropical Old World.

***Entadaabyssinica* Steud.** – Habit: Tree. Habitat: Woodland, wooded grassland, 1750–2290 m. Vouchers: Lugard 600 (EA), Hedberg O 1066 (UPS), Tweedie DR 1444 (BR). Native distribution range: Tropical Africa, Madagascar.

***Eriosemacordifolium* Hochst. ex A.Rich.** – Habit: Herb. Habitat: Grassland, 1750– 2290 m. Vouchers: Tweedie EM 198 (K). Native distribution range: Nigeria to Kenya and Angola.

***Eriosemajurionianum* Staner & De Craene** – Habit: Herb. Habitat: Forest edges, wet savanna, 1750–2800 m. Vouchers: Tweedie EM (1976) (REF), Agnew ADQ (2013) (REF). Native distribution range: Eritrea to Tanzania.

***Eriosemamacrostipulum* Baker f.** – Habit: Herb. Habitat: Woodland, grassland, around 2100 m. Voucher: Agnew ADQ (2013) (REF). Native distribution range: Tropical Africa.

***Eriosemamontanum* Baker f.** – Habit: Shrubby herb. Habitat: Wooded grassland, 1500–3300 m. Voucher: Gerh Lindblom (S). Native distribution range: Tropical Africa.

***Eriosemanutans* Schinz** – Habit: Herb. Habitat: Wooded grassland, wet savanna, forest edges, 1200–2400 m. Vouchers: Tweedie EM (1976) (REF), Agnew ADQ (2013) (REF). Native distribution range: Nigeria, Eritrea to S Africa.

**Eriosemascioanumsubsp.lejeunei (Staner & De Craene) Verdc.** – Habit: Herb. Habitat: Montane forest clearings, upland grassland, 2100–2500 m. Voucher: Tweedie EM 913 (K, EA). Native distribution range: Nigeria to E Tropical Africa.

***Eriosemasparsiflorum* Baker f.** – Habit: Herb. Habitat: Grassland, 1860–2250 m. Vouchers: Tweedie EM (1976) (REF), Agnew ADQ (2013) (REF). Native distribution range: W Tropical Africa to Kenya and Angola.

***Erythrinaabyssinica* Lam.** – Habit: Shrub or tree. Habitat: Wooded grassland, 1750–2250 m. Voucher: Rono et al. SAJIT-PR 0096 (EA, HIB). Native distribution range: Eritrea to Botswana.

***Flemingiagrahamiana* Wight & Arn.** – Habit: bushy herb, shrub or small tree. Habitat: Wooded grassland, 1170–2100 m. Vouchers: Tweedie EM (1976) (REF), Agnew ADQ (2013) (REF). Native distribution range: Tropical & S Africa to Arabian Peninsula, S India, S China (Yunnan) to Indo-China.

*Galegalindblomii (Harms) J.B.Gillett – Habit: Herb. Habitat: Grasslands, forest margins and bamboo area, 2000–3000 m. Vouchers: Bogdan A 4143 (K), Snowden 443 (EA), Gillett JB 18404 (US, WAG), BH Tothill 2375 (EA), Lugard 14 (EA), Beentje HJ 1938 (WAG), Lavranos JJ; Newton I 17768 A (WAG). Native distribution range: E Tropical Africa (Mt Elgon, Cherangani Hills).

***Hylodesmumrepandum* (Vahl) H.Ohashi & R.R.Mill** – Habit: Herb or shrub. Habitat: Forest, streamsides, roadsides, 1450–3000 m. Vouchers: Stein W Bie (UPS), Rono et al. SAJIT-PR 0064 (EA, HIB). Native distribution range: Africa, Arabian Peninsula, China (S Yunnan) to Tropical Asia.

***Indigoferaambelacensis* Schweinf.** – Habit: Herb. Habitat: Grassland, bushland, rocky grounds roadsides, 1000–2200 m. Voucher: Tweedie 1735 (EA). Native distribution range: Congo to Eritrea and Tanzania.

***Indigoferaarrecta* Hochst. ex A.Rich.** – Habit: Herb. Habitat: Bushland, grassland and forest edge, 300–2700 m. Voucher: Rwaburindore PK 465 (WAG). Native distribution range: Tropical & S Africa, Arabian Peninsula.

***Indigoferaatriceps* Hook.f.** – Habit: Herb. Habitat: Grassland, montane forest, 1000–3010 m. Vouchers: Tweedie 862 (EA), Mwangangi OM 446 (BR), Wood GHS 427 (EA). Native distribution range: Tropical Africa.

***Indigoferabogdanii* J.B.Gillett** – Habit: Shrub. Habitat: Grassland, roadside, rock outcrop, 1750–2750 m. Vouchers: Beentje HJ 1938 (EA), Tweedie MR 46 (EA), Bogdan A 4143 (EA). Native distribution range: Ethiopia to Tanzania.

***Indigoferabrevicalyx* Baker f.** – Habit: Herb. Habitat: Grassland, woodland, 1200–2500 m. Vouchers: Tweedie 861, Tweedie EM 765 (K). Native distribution range: Eritrea to E Central & E Tropical Africa, SW Arabian Peninsula.

***Indigoferacircinella* Baker f.** – Habit: Herb. Habitat: Shallow soil grassland, roadside, Wooded grassland, 1100–2200 m. Vouchers: Tweedie EM (1976) (REF), Agnew ADQ (2013) (REF). Native distribution range: Kenya to Zimbabwe.

***Indigoferaconjugata* Baker** – Habit: Herb. Habitat: Grassland, wooded grassland, 1750–2250 m. Voucher: Tweedie EM (1976) (REF). Native distribution range: Tropical Africa.

^**EX**^***Indigoferaemarginella* Steud. ex A.Rich.** – Habit: Shrub. Habitat: Grassland, 900–2300 m. Vouchers: Tweedie EM (1976) (REF), Agnew ADQ (2013) (REF). Native distribution range: Nigeria to Eritrea and S Tropical Africa.

^**EX**^***Indigoferahomblei* Baker f. & Martin** – Habit: Shrub. Habitat: Montane grassland, 1600–3100 m. Voucher: Rono et al. SAJIT-PR 0097 (EA, HIB). Native distribution range: Cameroon to Tanzania and Eswatini.

***Indigoferalongistaminata* Schrire** – Habit: Shrub. Habitat: Montane grassland, thickets, 1750–2250 m. Vouchers: Tweedie EM (1976) (REF), Agnew ADQ (2013) (REF). Native distribution range: Cameroon to Kenya and Malawi.

***Indigoferamimosoides* Baker** – Habit: Herb or shrub. Habitat: Grassland, bushland, montane forest margin, 1150–2300 m. Voucher: Tweedie 874 (EA). Native distribution range: Cameroon to Ethiopia and S Africa.

**Indigoferaschimperivar.schimperi Jaub.** – Habit: Herb. Habitat: Montane forest, 1000–3010 m. Voucher: Wood GHS 432 (EA). Native distribution range: Eritrea to S Africa, SW Arabian Peninsula.

**^EX^*Indigoferaschlechteri* Baker f.** – Habit: Woody herb or shrub. Habitat: Grassland, roadside, bamboo zone, forest margin, 1500–2700 m. Vouchers: Tweedie EM (1976) (REF), Agnew ADQ (2013) (REF). Native distribution range: S Africa.

***Indigoferaspicata* Forssk.** – Habit: Herb. Habitat: Disturbed grassland and woodland, 0–2900 m. Vouchers: Tweedie EM (1976) (REF), Agnew ADQ (2013) (REF). Native distribution range: Africa, Arabian Peninsula.

***Indigoferasubargentea* De Wild.** – Habit: Herb. Habitat: Montane grassland, 1800–2000 m. Vouchers: Tweedie EM (1976) (REF), Agnew ADQ (2013) (REF). Native distribution range: Cameroon to Ethiopia and Zambia.

**Indigoferasubulatavar.scabra (Roth) Meikle** – Habit: Herb or shrub. Habitat: Grassland, 1750–2250 m. Voucher: Tweedie EM (1976) (REF). Native distribution range: Tropics & Subtropics.

***Indigoferatrita* L.f.** – Habit: Herb or shrub. Habitat: Grassland, bushland, forest margin, 1700–2500 m. Vouchers: Tweedie EM (1976) (REF), Agnew ADQ (2013) (REF). Native distribution range: Tropical & Subtropical Old World.

***Indigoferavohemarensis* Baill.** – Habit: Herb or shrub. Habitat: Grassland, 1750–2250 m. Voucher: Tweedie EM (1976) (REF). Native distribution range: Eritrea to N Mozambique, Comoros, Madagascar.

***Lablabpurpureus* (L.) Sweet** – Habit: Climbing herb. Habitat: Riverine forest edge, 0001–2400 m. Vouchers: Tweedie EM (1976) (REF), Agnew ADQ (2013) (REF). Native distribution range: Cape Verde, Tropical & S Africa, Madagascar, India.

***Lathyrushygrophilus* Taub.** – Habit: Straggling or climbing herb. Habitat: Swampy grassland, bamboo edges, 1800–4100 m. Vouchers: Tweedie EM (1976) (REF), Agnew ADQ (2013) (REF). Native distribution range: Ethiopia to Malawi.

***Lathyrussphaericus* Retz.** – Habit: herb. Habitat: Open scrubs, upland forest grassland, 1950–3000 m. Vouchers: Tweedie EM (1976) (REF), Agnew ADQ (2013) (REF). Native distribution range: E Central Europe to Mediterranean and Afghanistan, Tropical African Mountains.

***Lotusbecquetii* Boutique** – Habit: Herb. Habitat: Grassland, rocky hillside, 2000–2870 m. Vouchers: Tweedie EM (1976) (REF), Agnew ADQ (2013) (REF). Native distribution range: South Sudan to Burundi.

***Lotusgoetzei* Harms** – Habit: Woody herb. Habitat: Grassland, open bushland, forest margin, on young volcanic soil, 1750–3140 m. Vouchers: Tweedie EM (1976) (REF), Agnew ADQ (2013) (REF). Native distribution range: Ethiopia to S Tropical Africa.

***Macrotylomaaxillare* (E.Mey.) Verdc.** – Habit: Climbing or trailing herb. Habitat: Forest edges, bushlands, 0001–2500 m. Vouchers: Tweedie EM (1976) (REF), Agnew ADQ (2013) (REF). Native distribution range: Tropical & S Africa, SW Arabian Peninsula Sri Lanka, W Indian Ocean.

^**EX**^**Macrotylomaaxillarevar.glabrum (E.Mey.) Verdc.** – Habit: Climbing or trailing herb. Habitat: Woodland, 1750–2400 m. Voucher: Gardner in FD 2855 (EA). Native distribution range: South Sudan to S Africa, W Indian Ocean.

***Mucunastans* Welw. ex Baker** – Habit: Shrub. Habitat: Wooded grassland, 1370–1780 m. Voucher: Tweedie EM 924 (K). Native distribution range: Nigeria to W Ethiopia and S Tropical Africa.

**Neonotoniawightiisubsp.petitiana (A.Rich.) J.A.Lackey** – Habit: Climber herb. Habitat: Forest edges, disturbed grounds, above 1500 m. Voucher: Kahuho S 5 (US). Native distribution range: Ethiopia to Tanzania.

***Piliostigmathonningii* (Schumach.) Milne-Redh.** – Habit: Tree. Habitat: Forest, wet grassland, woodland, 1750–1900 m. Voucher: Padwa JH 29 (EA). Native distribution range: Africa, SW Arabian Peninsula.

***Pseudarthriaconfertiflora* (A.Rich.) Baker** – Habit: Woody herb or subshrub. Habitat: Grassland, thickets, bracken areas, 1750–2280 m. Voucher: Tweedie 1362 (EA). Native distribution range: Tropical Africa.

***Pseudarthriahookeri* Wight & Arn.** – Habit: Woody herb or shrub. Habitat: Wet grassland, 1750–2250 m. Voucher: Tweedie EM (1976) (REF). Native distribution range: Tropical & S Africa, W Indian Ocean.

***Pterolobiumstellatum* (Forssk.) Brenan** – Habit: Shrub. Habitat: Forest, Wooded grassland, grassland, bushland, 1750–2350 m. Voucher: Lugard 237 (EA). Native distribution range: Eritrea to S Africa, SW Arabian Peninsula.

***Rhynchosiaelegans* A.Rich.** – Habit: Climbing or trailing herb. Habitat: Woodland, 1750–2400 m. Vouchers: Tweedie EM (1976) (REF), Agnew ADQ (2013) (REF). Native distribution range: Eritrea to Malawi, SW Arabian Peninsula.

***Rhynchosiaferruginea* A.Rich.** – Habit: Climbing herb. Habitat: Uplands, subalpine grasslands, forest edges, 1750–3000 m. Voucher: Tweedie 1405 (EA). Native distribution range: Eritrea to E Central & E Tropical Africa.

***Rhynchosiahirta* (Andrews) Meikle & Verdc.** – Habit: Climbing herb or shrub. Habitat: Grassland, forest edges, 0001–1800 m. Vouchers: Tweedie EM (1976) (REF), Agnew ADQ (2013) (REF). Native distribution range: Tropical & S Africa, India Sri Lanka.

***Rhynchosiakilimandscharica* Volkens ex Harms** – Habit: Climbing or trailing herb. Habitat: Forest edges, grassland, bushland, 1750–2550 m. Voucher: Tweedie 721 (EA). Native distribution range: E Central & E Tropical Africa.

***Rhynchosiaminima* (L.) DC.** – Habit: Climbing herb. Habitat: Forest, grassland, 150–2100 m. Vouchers: Tweedie EM (1976) (REF), Agnew ADQ (2013) (REF). Native distribution range: Tropics & Subtropics.

**^EX^Rhynchosiaminimavar.prostrata (Harv.) Meikle** – Habit: Climbing herb. Habitat: Forest, grassland, woodland, 1750–2250 m. Voucher: Tweedie EM (1976) (REF). Native distribution range: Tropical & S Africa, SW Arabian Peninsula.

***Rhynchosiaorthobotrya* Harms** – Habit: Herb or undershrub. Habitat: Wooded grassland, 1000–2100 m. Voucher: Tweedie 1140 (K). Native distribution range: W Tropical Africa to Eritrea and Tanzania.

***Rhynchosiaresinosa* (Hochst. ex A.Rich.) Baker** – Habit: Woody climbing herb. Habitat: Bushlands, 1530–2500 m. Vouchers: Tweedie EM (1976) (REF), Agnew ADQ (2013) (REF). Native distribution range: Tropical & S Africa.

***Rhynchosiausambarensis* Taub.** – Habit: Climbing herb. Habitat: Uplands grasslands, forest, 1650–2400 m. Vouchers: Tweedie EM (1976) (REF), Agnew ADQ (2013) (REF). Native distribution range: Eritrea to Rwanda and Tanzania.

**Senegaliagoetzeisubsp.microphylla (Brenan) Kyal. & Boatwr.** – Habit: Tree. Habitat: Wooded grassland, 1750–2250 m. Voucher: Tweedie EM (1976) (REF). Native distribution range: Ethiopia to South Africa.

***Senegaliapersiciflora* (Pax) Kyal. & Boatwr.** – Habit: Tree. Habitat: Woodland, wooded grassland, 1750–2250 m. Vouchers: Tweedie EM 2120 (MO, BR), Eggeling WJ 2490 (BR). Native distribution range: Ethiopia to DR Congo.

^**EX**^***Sennabicapsularis* (L.) Roxb.** – Habit: Shrub or tree. Habitat: Disturbed bushland/cultivation, grassland, roadside, 100–1900 m. Voucher: Agnew ADQ (2013) (REF). Native distribution range: Tropical America.

***Sennadidymobotrya* (Fresen.) H.S.Irwin & Barneby** – Habit: Shrub. Habitat: Forest, woodland, 1750–2440 m. Vouchers: Jackson THE 304, 2940 (EA). Native distribution range: Ethiopia to S Tropical Africa.

***Sennapetersiana* (Bolle) Lock** – Habit: Shrub or small tree. Habitat: Moist forest edge, wooded grassland, riverine forest, 1750–2250 m. Voucher: Tweedie EM (1976) (REF). Native distribution range: Cameroon to Ethiopia and S Africa, Madagascar.

***Sennasingueana* (Delile) Lock** – Habit: Shrub or tree. Habitat: Woodland, wooded grassland, 1750–2250 m. Voucher: Dale IR 664, 684 (EA). Native distribution range: Tropical Africa to NW Namibia.

***Sesbaniadummeri* E.Phillips & Hutch.** – Habit: Shrub or small tree. Habitat: Swamps, 1100–2000 m. Vouchers: Chater-Jack 131 (EA). Native distribution range: SW Kenya to Rwanda.

***Sesbaniasesban* (L.) Merr.** – Habit: Shrub or tree. Habitat: Stream sides, 1750–2200 m. Voucher: Lugard 196 (EA). Native distribution range: Tropical & S Africa, Arabian Peninsula, Indian Subcontinent.

***Smithiaelliotii* Baker f.** – Habit: Herb. Habitat: Swamp in montane forest, 1170–2700 m. Voucher: Tweedie 1325 (EA). Native distribution range: Nigeria to Ethiopia and S Tropical Africa, Madagascar.

***Sphenostylisstenocarpa* Harms** – Habit: Climbing herb. Habitat: Wooded grassland, 50–2300 m. Voucher: Agnew ADQ (2013) (REF). Native distribution range: Tropical Africa.

***Tephrosiaemeroides* A.Rich.** – Habit: Woody herb. Habitat: Upland bushland, 1600–2600 m. Vouchers: Tweedie EM (1976) (REF), Agnew ADQ (2013) (REF). Native distribution range: NE Tropical Africa to Uganda.

***Tephrosiaholstii* Taub.** – Habit: Herb. Habitat: Forest margin, grassland, 1750–2400 m. Voucher: Tweedie DR 718 (BR). Native distribution range: Ethiopia to Tanzania.

***Tephrosiainterrupta* Hochst. & Steud. ex Engl.** – Habit: Shrub. Habitat: Montane grassland, thicket, 1400–3100 m. Vouchers: Tweedie EM (1976) (REF), Agnew ADQ (2013) (REF). Native distribution range: Eritrea to S Tropical Africa.

***Tephrosianyikensis* Baker** – Habit: Herb or shrub. Habitat: Montane grassland, forest, 1750–2250 m. Voucher: Tweedie 465 (K). Native distribution range: Kenya to S Tropical Africa.

***Tephrosiapaniculata* Welw. ex Baker** – Habit: Herb. Habitat: Upland grassland, swamps, 1200–2400 m. Voucher: Lugard 229 (EA). Native distribution range: Tropical Africa.

***Tephrosiavogelii* Hook.f.** – Habit: Woody herb. Habitat: Forest margin, old cultivations, grassland, 0–2300 m. Vouchers: Lugard in Nat. Hist. soc 6023, Lugard C & Lugard EJ 6023 (EA), Beekley 6023 (EA). Native distribution range: Tropical & S Africa.

***Teramnusrepens* Baker f.** – Habit: Climbing herb. Habitat: Grassland, bushland, 1750–2250 m. Voucher: Tweedie EM (1976) (REF). Native distribution range: NE Tropical Africa to Mozambique, Comoros, Madagascar, Arabian Peninsula, Pakistan to India.

***Teramnusuncinatus* Sw.** – Habit: Climber herb. Habitat: Forest, wooded grassland, 1700–2500 m. Vouchers: Tweedie EM (1976) (REF), Agnew ADQ (2013) (REF). Native distribution range: Mexico to Tropical America, Tropical Africa, Comoros, Madagascar.

**Teramnusuncinatussubsp.ringoetii (De Wild.) Verdc.** – Habit: Climber or prostrate herb. Habitat: Forest, 1750–2250 m. Voucher: Tweedie EM (1976) (REF). Native distribution range: Tropical Africa.

*Trifoliumacaule Steud. ex A.Rich. – Habit: Herb. Habitat: Grassland, rocky alpine zone, 3300–4100 m. Voucher: Dummer RA 3309 (US). Native distribution range: Eritrea to Uganda.

***Trifoliumburchellianum* Ser.** – Habit: Herb. Habitat: Forest, moorland, 1700–3700 m. Voucher: Powles 33 (K). Native distribution range: Ethiopia to S Africa.

***Trifoliumcryptopodium* Steud. ex A.Rich.** – Habit: Herb. Habitat: Forest openings, alpine zone, moorland, 2100–4200 m. Vouchers: Gillett JB 18412 (WAG), Tweedie DR 28, 29, 120 (EA), Lugard C 333 (EA), Knight J 55 (EA), Webster MVB 8797 (EA), Rono et al. SAJIT-PR 0164 (EA, HIB). Native distribution range: Eritrea to Tanzania.

*Trifoliumelgonense J.B.Gillett – Habit: Herb. Habitat: Forest, moorland, alpine zone, 2800–3500 m. Vouchers: Hedberg O 266 (K, S, WAG), Gillett JB 18409 (WAG). Native distribution range: Ethiopia to Uganda, (U3, K3).

*Trifoliumlugardii Bullock – Habit: Herb. Habitat: Grassland, forest margin, 1700–2650 m. Voucher: Lugard 656 (EA). Native distribution range: Uganda to Kenya.

***Trifoliummultinerve* A.Rich.** – Habit: Herb. Habitat: Grassland, moorland, 1800–3700 m. Voucher: Gillett JB 18407 (WAG). Native distribution range: Eritrea to Rwanda.

***Trifoliumrueppellianum* Fresen.** – Habit: Herb. Habitat: Grassland, moorland, 1700–3700 m. Vouchers: Tweedie EM (1976) (REF), Agnew ADQ (2013) (REF). Native distribution range: Nigeria to Eritrea and S Tropical Africa.

***Trifoliumsemipilosum* Fresen.** – Habit: Herb. Habitat: Grassland, 1500–3000 m. Voucher: Agnew ADQ (2013) (REF). Native distribution range: Eritrea to S Tropical Africa, SW Arabian Peninsula.

***Trifoliumsimense* Fresen.** – Habit: Herb. Habitat: Forest, woodland, 1750–3150 m. Voucher: Tweedie EM (1976) (REF). Native distribution range: Bioko to Eritrea and Zambia.

***Trifoliumsteudneri* Schweinf.** – Habit: Herb. Habitat: Wet grassland, 1800–2500 m. Vouchers: Tweedie EM (1976) (REF), Agnew ADQ (2013) (REF). Native distribution range: Eritrea to Uganda.

***Trifoliumtembense* Fresen.** – Habit: Herb. Habitat: Grassland, forest, moorland openings, alpine zone, 2000–3800 m. Voucher: Knight J 55 (EA). Native distribution range: Eritrea to Rwanda and Tanzania.

***Trifoliumusambarense* Taub. ex Engl.** – Habit: Herb. Habitat: Forest, 1500–2750 m. Voucher: Beentje HJ 1962 (WAG). Native distribution range: Nigeria to Ethiopia and S Tropical Africa.

***Tylosemafassoglense* (Kotschy ex Schweinf.) Torre & Hillc.** – Habit: Herb or shrub. Habitat: Wooded grassland, grassland, bushland, 90–1900 m. Voucher: Agnew ADQ (2013) (REF). Native distribution range: Ethiopia to S Africa.

**Vachelliaabyssinicasubsp.calophylla (Brenan) Kyal. & Boatwr.** – Habit: Tree. Habitat: Montane forest, wooded grassland, woodland, 1750–2300 m. Voucher: Eggling 2475 (EA). Native distribution range: South Sudan to S Tropical Africa.

***Vachelliaamythethophylla* (Steud. ex A.Rich.) Kyal. & Boatwr.** – Habit: Tree. Habitat: Woodland, wooded grassland, 1750–2400 m. Voucher: Tweedie DR 1531 (BR). Native distribution range: Tropical Africa.

***Vachelliadrepanolobium* (Harms ex Y.Sjöstedt) P.J.H.Hurter** – Habit: Shrub or small tree. Habitat: Wooded grassland, 1750–2250 m. Voucher: Tweedie EM (1976) (REF). Native distribution range: Sudan to Tanzania.

***Vachelliagerrardii* (Benth.) P.J.H.Hurter** – Habit: Shrub or tree. Habitat: Wooded grassland, 1750–2250 m. Voucher: Tweedie EM (1976) (REF). Native distribution range: Tropical & S Africa, S Israel to Arabian Peninsula.

***Vachelliahockii* (De Wild.) Seigler & Ebinger** – Habit: Shrub or tree. Habitat: Woodland, wooded grassland, 1750–2250 m. Voucher: Tweedie EM 2273 (MO). Native distribution range: Tropical Africa, SW Arabian Peninsula.

***Vachellialahai* (Steud. & Hochst. ex Benth.) Kyal. & Boatwr.** – Habit: Tree. Habitat: Woodland, wooded grassland, 1750–2400 m. Vouchers: Eggeling WJ 2477 (MO, BR), Lugard 513 (MO). Native distribution range: Eritrea to N Tanzania.

^**EX**^**Vachelliasieberianavar.woodii (Burtt Davy) Kyal. & Boatwr.** – Habit: Tree or shrub. Habitat: Woodland, wooded grassland, 1750–2250 m. Voucher: Tweedie EM (1976) (REF). Native distribution range: Sudan to S Africa.

^**EX**^***Viciaeriocarpa* (Hausskn.) Halácsy** – Habit: Trailing or climbing herb. Habitat: Wet savanna, 1750–2250 m. Voucher: Tweedie EM (1976) (REF). Native distribution range: Algeria, E Mediterranean to Arabian Peninsula.

***Viciahirsuta* (L.) Gray** – Habit: Trailing or climbing herb. Habitat: Grassland, 1950–3360 m. Voucher: Tweedie EM 875 (K). Native distribution range: Macaronesia, Temperate Eurasia, N Africa to Tanzania.

***Viciapaucifolia* Baker** – Habit: Trailing or climbing herb. Habitat: Grassland, forest edges, 1500–2750 m. Voucher: Agnew ADQ (2013) (REF). Native distribution range: Congo to Ethiopia and Zambia.

***Viciasativa* L.** – Habit: Trailing or climbing herb. Habitat: Grassland, 1700–3350 m. Vouchers: Tweedie EM (1976) (REF), Agnew ADQ (2013) (REF). Native distribution range: Temperate Eurasia to Arabian Peninsula, Macaronesia, N Africa to Kenya.

**Viciasativasubsp.nigra (L.) Ehrh.** – Habit: Herb. Habitat: Woodland, forest, 1750–3000 m. Voucher: Tweedie EM (1976) (REF). Native distribution range: Macaronesia, Temperate Eurasia, N Africa to Kenya.

**^EX^*Viciavillosa* Roth** – Habit: Trailing or climbing herb. Habitat: Escape from upland cultivation, forest, grassland, 1850–2700 m. Vouchers: Tweedie EM (1976) (REF), Agnew ADQ (2013) (REF). Native distribution range: Canary Islands, N Africa, Europe to Central Asia and Afghanistan.

***Vignafriesiorum* Harms** – Habit: Trailing herb. Habitat: Grassland, 1600–2400 m. Vouchers: Lugard EJ (CIAT, BHAM_Maxted). Native distribution range: Ethiopia to E Central & E Tropical Africa.

***Vignafrutescens* A.Rich.** – Habit: Prostrate or climbing herb. Habitat: Forest, grassland, woodland, 1750–2400 m. Voucher: Tweedie DR (CIAT). Native distribution range: Tropical & S Africa.

**Vignafrutescenssubsp.incana (Taub.) Verdc.** – Habit: Trailing woody herb. Habitat: Grassland, bushland, rocky places, 0–2400 m. Vouchers: Tweedie EM (1976) (REF), Agnew ADQ (2013) (REF). Native distribution range: Cameroon, E Tropical Africa.

***Vignaheterophylla* A.Rich.** – Habit: Climber herb. Habitat: Grassland, 1600–2300 m. Vouchers: Tweedie DR (CIAT). Native distribution range: Cameroon to Eritrea and S Tropical Africa, SW Arabian Peninsula.

**^EX^*Vignahosei* (Craib) Backer** – Habit: Climber herb. Habitat: Woodland, lower forest edge, 1750–2400 m. Vouchers: Tweedie EM (1976) (REF), Agnew ADQ (2013) (REF). Native distribution range: Taiwan, W Malesia.

***Vignamembranacea* A.Rich.** – Habit: Climber herb. Habitat: Forest, bushland, woodland, 1750–2250 m. Vouchers: Tweedie EM 2229 (MO), Irwin PH (CIAT), Tweedie DR (CIAT). Native distribution range: Eritrea to Tanzania, SW Arabian Peninsula.

***Vignamonophylla* Taub.** – Habit: Climbing herb. Habitat: Wooded grassland, 1650–2500 m. Vouchers: Tweedie DR (CIAT, BHAM_Maxted). Native distribution range: Ethiopia to Botswana.

***Vignaparkeri* Baker** – Habit: Twining, prostrate, or mat forming herb. Habitat: Grassland, forest margin, 1500–2900 m. Vouchers: Symes YE 252 (EA), Major C & Lugard EJ 180 (EA). Native distribution range: Tropical Africa, Madagascar.

**Vignaparkerisubsp.maranguensis (Taub. ex Engl.) Verdc.** – Habit: Herb. Habitat: Grassland, 1500–2900 m. Vouchers: Snowden JD 433 (NHMUK), Lugard EJ (CIAT), Snowden JD (CIAT), Tweedie DR (CIAT), Rono et al. SAJIT-PR 0224 (EA, HIB). Native distribution range: Tropical Africa, Madagascar.

***Vignaschimperi* Baker** – Habit: Twiner herb. Habitat: Upland grassland, forest margin, riverside, 1250–2800 m. Vouchers: Tweedie EM 423 (K), Jackson G (EA). Native distribution range: Ethiopia to Malawi.

***Vignavexillata* (L.) A.Rich.** – Habit: Climbing herb. Habitat: Grassland, bushlands esp. among rocks, 100–2500 m. Vouchers: Jack C 31 (EA), Boonman 6612 (EA), Webster MVB 9191 (EA). Native distribution range: Tropics & Subtropics.

***Zorniapratensis* Milne-Redh.** – Habit: Herb. Habitat: Wooded grassland, 1200–2300 m. Vouchers: Tweedie EM (1976) (REF), Agnew ADQ (2013) (REF). Native distribution range: Cameroon to Ethiopia and S Tropical Africa.

***Zorniasetosa* Baker f.** – Habit: Herb. Habitat: Grassland, 1560–2350 m. Voucher: Tweedie EM 804 (K). Native distribution range: Ethiopia to Namibia.

**Zorniasetosasubsp.obovata (Baker f.) J.Léonard & Milne-Redh.** – Habit: Herb. Habitat: Grassland, cultivated land, 1100–2400 m. Voucher: Tweedie EM 804 (K). Native distribution range: Ethiopia to Zambia.

#### F67. FUMARIACEAE

1 Genus, 1 species

***Corydalismildbraedii* Fedde** – Habit: Herb. Habitat: Forest, bamboo forest, upland moors, grassland, 2200–3600 m. Vouchers: Tweedie 1282 (K), 1522 (EA), Thomas 523 (EA), Tothill BH 2250 (EA), Williams JG 74/11 (BR). Native distribution range: S Central Ethiopia to E Central & E Tropical Africa.

#### F68. GENTIANACEAE

5 Genera, 16 species

***Anthocleistagrandiflora* Gilg** – Habit: Tree. Habitat: Forest, 1500–2300 m. Vouchers: Dale U78 (EA, MO), Snowden JD 517 (MO). Native distribution range: Cameroon to South Sudan and S Africa, Comoros.

***Chironiaelgonensis* Bullock** – Habit: Herb. Habitat: Swamps in grassland, 1705–2325 m. Vouchers: Lugard C & Lugard EJ 21 (EA/K). Native distribution range: Kenya to N Zambia.

***Exochaeniumgrande* Griseb.** – Habit: Herb. Habitat: Wooded grassland on shallow soils, 1550–2550 m. Vouchers: Tweedie DR 64 (EA), Andersen R 17 (S), Tweedie 586 (EA). Native distribution range: Nigeria to Ethiopia and S Africa.

***Sebaeabrachyphylla* Griseb.** – Habit: Herb. Habitat: Wet path sides, streamside, montane grassland, heath zone, moorland, 1650–3500 m. Vouchers: Mwangangi OM 441 (EA), Knox EB 2618 (EA), Hedberg O 216 (S), Andersen R 288 (S), Rono et al. SAJIT-PR 0254 (EA, HIB). Native distribution range: Nigeria to Ethiopia and S Tropical Africa, Madagascar.

***Sebaealeiostyla* Gilg** – Habit: Herb. Habitat: Wet grassland, heath zone, 2000–3500 m. Vouchers: Hedberg O 216 (EA), Tweedie EM 17 (EA), Sileshi Nemomissa 970828-3/1(EA). Native distribution range: Ethiopia to S Africa.

***Sebaeamicrophylla* Knobl.** – Habit: Saprophytic herb. Habitat: Outcrop rock, forest, moist grassland and woodland, 1800–3000 m. Vouchers: Irwin PH 119 (EA), Jack C 312 (EA), Lind EM 298 (EA), Thomas AS 460 (EA). Native distribution range: Ethiopia to S Tropical Africa, Socotra, SW Arabian Peninsula, Indian Subcontinent to China (Yunnan).

***Swertia Abyssinica* Hochst.** – Habit: Herb. Habitat: Grassland, moorland, 2100–3300 m. Vouchers: Mulugeta Kekede 187, 202 (EA), Knox EB 2644 (EA), Rose 10204 (EA), Marrison 254 (EA), Tothill BH 2354 (S). Native distribution range: Bioko, Nigeria to SW Cameroon, Eritrea to Zambia.

***Swertiabrownii* J.Shah** – Habit: Herb. Habitat: Forest, upland moist grassland, 1500–3000 m. Vouchers: Tweedie 835 (EA), Hedberg O 98 (S), Rono et al. SAJIT-PR 0261 (EA, HIB). Native distribution range: S Ethiopia to E Central & E Tropical Africa.

*Swertiacrassiuscula Gilg – Habit: Herb. Habitat: Stony, swampy shallow black loam soils, lower alpine mooorland zones, 2700–4500 m. Vouchers: Tweedie 44, 3890 (EA), Mwangangi OM 311 (EA), Bickford N 18, 48 (EA), Tweedie DR 128, (EA), Williams 2147 (EA), Taylor G & Verdcourt B 2477 (EA), Rono et al. SAJIT-PR 0140 (EA, HIB), Dummer RA 3750 (K), Hedberg 144 (EA), 963 (S) Rose 10206 (EA), Saund & Hancock 90 (EA), Hedberg 963 (EA), Lye 1412 (EA). Native distribution range: SE Uganda to N Tanzania.

***Swertiakilimandscharica* Engl.** – Habit: Herb. Habitat: Montane, subalpine grassland, 2100–3840 m. Vouchers: Lye KA 1456 (EA), Hedberg O 261 (S), 4501 (EA), Gosnell JM 60, 630 (EA), Rauh W 598 (EA), Kirika P Mbale M & Muthoka P (EA), Rono et al. SAJIT-PR 0137 (EA, HIB). Native distribution range: Ethiopia to N Malawi.

***Swertialugardae* Bullock** – Habit: Herb. Habitat: Upper forest edge, grassland, moorland, 3000–4321 m. Vouchers: Sileshi 970827-2/6 (EA), Tothill BH 2347 (EA), Mulugeta Kekede 203 (EA), Hedberg KO 4508 (K), Tweedie DR 4032 (EA), Mwangangi OM 317 (EA), Major C & Lugard EJ 409 (EA, K), Bickford N 50 (EA), Kirika P & Muthoka P 1171 (EA). Native distribution range: Ethiopia to SE Uganda.

*Swertiasubnivalis T.C.E.Fr. – Habit: Herb. Habitat: Rare in wet mossy communities of alpine zone, along streams, 3600–4321 m. Vouchers: Hedberg O 879 (EA, BR), 1018 (S). Native distribution range: Ethiopia to Central Kenya.

***Swertiatetrandra* Hochst.** – Habit: Herb. Habitat: Moist grassland, 1550–2250 m. Vouchers: Tweedie 397 (EA), Lind EM 294 (EA). Native distribution range: Ethiopia to E Zimbabwe.

**Swertiauniflora Mildbr. – Habit: Herb. Habitat: Rare in alpine grassland, 3750–4321 m. Vouchers: Dale IR 3089 (EA), Simkin J 970828-3/4 (EA), Adamson J 488 (EA) Hedberg O 926 (EA, S), Tweedie DR 10 (EA), Sileshi Nemomissa 970828-3/5 (EA), Eggeling WJ 5754 (EA), Knox EB 2619 (EA). Native distribution range: E Uganda to W Kenya (Mt. Elgon).

***Swertiausambarensis* Engl.** – Habit: Herb. Habitat: Montane grassland, 1600–3900 m. Vouchers: Bie SW 269 (EA), Sileshi Nemomissa 970827-2/3 (EA), Mwangangi OM 317 (EA). Native distribution range: Cameroon, Ethiopia to S Tropical Africa.

***Swertiawelwitschii* Engl.** – Habit: Herb. Habitat: Marshes, roadsides, wet grassland 1845–2400 m. Voucher: Jack C 28 (EA). Native distribution range: Ethiopia to S Africa.

#### F69. GERANIACEAE

3 Genera, 10 species

***Geraniumaculeolatum* Oliv.** – Habit: Trailling or climbing herb. Habitat: Woodland, montane forest, 1800–3400 m. Voucher: Rono et al. SAJIT-PR 0226 (EA, HIB). Native distribution range: Ethiopia to N Mozambique.

***Geraniumarabicum* Forssk.** – Habit: Herb. Habitat: Grassland, montane forest, alpine belt, 1800–3940 m. Vouchers: Tothill BH 2377 (EA), Beentje HJ 1960 (WAG). Native distribution range: Egypt to Tropical Africa, Madagascar, SW Arabian Peninsula.

**^EX^*Geraniumbequaertii* De Wild.** – Habit: Herb. Habitat: Grassland, roadside, 1500–2100 m. Voucher: Snowden JD 425 (NHMUK). Native distribution range: E DR Congo.

*Geraniumkilimandscharicum Engl. – Habit: Prostrate herb. Habitat: Upper moor, upland grassland, forest, alpine zones, 2260–4300 m. Vouchers: Major EJ; Lugard EJ 411 (K), Lugard C 328 (K), Synge 976 (EA), Forbes 271 (EA), Hooper SS; Townsend CC 1463 (K), Irwin PH 122 (K), Tweedie DR 37 (K), Tweedie 1391 (K), Synge PM 1895 (NHMUK). Native distribution range: E Tropical African Mountains.

**^EX^*Geraniummascatense* Boiss.** – Habit: Herb. Habitat: Forest, woodland, 1600–2600 m. Vouchers: Tweedie EM (1976) (REF), Agnew ADQ (2013) (REF). Native distribution range: Egypt and NE Tropical Africa, Arabian Peninsula, SW & S Iran to NW India.

***Geraniumpurpureum* Vill.** – Habit: Herb. Habitat: Montane forest, woodland, 2200–3000 m. Vouchers: Tweedie EM (1976) (REF), Agnew ADQ (2013) (REF). Native distribution range: Macaronesia, Europe to Iran and Tanzania.

***Geraniumvagans* Baker** – Habit: Herb. Habitat: Montane forest grassland, alpine zones, 2640–4321 m. Vouchers: Naiga 52 (K), Hedberg 844, Thomas AS 596 (EA), Rauh W 599 (EA), Adamson J 496 (EA), Luyard EF 356 (EA). Native distribution range: E Tropical Africa to E Zambia.

***Monsoniaangustifolia* E.Mey.** – Habit: Herb. Habitat: Open woodland, grassland, river banks, wet grasslands, 1200–2600 m. Vouchers: Tweedie EM (1976) (REF), Agnew ADQ (2013) (REF). Native distribution range: Nigeria, Eritrea to S Africa, Madagascar.

**^EX^*Pelargoniumalchemilloides* (L.) L’Hér.** – Habit: Herb. Habitat: Grassland, wooded grassland, outcrop rock, 1000–2800 m. Voucher: Rono et al. SAJIT-PR 0082 (EA, HIB). Native distribution range: E Zimbabwe to S Africa.

***Pelargoniumwhytei* Baker** – Habit: Herb. Habitat: Montane forest, heath zone, 1700–3500 m. Voucher: Synge 1925 (EA). Native distribution range: Central Ethiopia to Zambia.

#### F70. HAMAMELIDACEAE

1 Genus, 2 species

***Trichocladusellipticus* Eckl. & Zeyh.** – Habit: Shrub or tree. Habitat: Upland forest, streams, swampy areas, 1750–2700 m. Voucher: Rono et al. SAJIT-PR 0068 (EA, HIB). Native distribution range: Subtropical Africa to South Africa.

**Trichocladusellipticussubsp.malosanus (Baker) Verdc.** – Habit: Shrub or tree. Habitat: Moist forest, 2000–2300 m. Voucher: Pole-Evans IB & Erens J 1502 (US). Native distribution range: Ethiopia to S Tropical Africa.

#### F71. HYPERICACEAE

2 Genera, 11 species

***Harunganamadagascariensis* Poir.** – Habit: Tree. Habitat: Forest, 1200–1800 m. Voucher: Snowden JD 967 (EA). Native distribution range: Tropical & S Africa, W Indian Ocean.

***Hypericumannulatum* Moris** – Habit: Herb. Habitat: Grassland, 1100–2700 m. Vouchers: Maitland 1180 (EA), Snowden JD 522 (EA). Native distribution range: Sardegna, N & Central Balkan Peninsula, NE & E Tropical Africa, Arabian Peninsula.

*Hypericumannulatumsubsp.afromontanum (Bullock) N.Robson – Habit: Herb. Habitat: Upland and moor grassland, 3000–3700 m. Vouchers: Snowden JD 479 (EA), Thomas AS 2700 (EA), Dummer RA 3301 (EA), Lugard 338a (EA), Gardner 2259 (EA). Native distribution range: SE Ethiopia to N Tanzania.

*Hypericumkiboense Oliv. – Habit: Shrub. Habitat: Forest edge, 2100–3900 m. Vouchers: Dummer RA 3504 (EA), Liebenberg 1659 (EA), Soumdy & Hancock 60 (EA), Lugard C 323 (K), Townsend CC 2324 (K), Kisalye N 108 (K), George Taylor 3614 (BR), Katende 1120 (K). Native distribution range: E Tropical African Mountains.

**^EX^*Hypericumlanceolatum* Lam.** – Habit: Shrub or tree. Habitat: Forest, bushland, streamside, grassland, 1800–3360 m. Vouchers: Eggeling 2460 (EA), Tweedie 876 (EA). Native distribution range: Comoros, Réunion.

***Hypericumpeplidifolium* A.Rich.** – Habit: Herb. Habitat: Alpine and subalpine grassland and stream edges, 1350–3600 m. Vouchers: Tweedie 455 (EA), George Taylor 3660 (BM), Lugard EJ 205 (EA), Adamson J 467 (EA), Jack C 74 (EA), Mamwaring Miss 2667 (EA), Rono et al. SAJIT-PR 0060 (EA, HIB). Native distribution range: Bioko to Eritrea and S Tropical Africa.

***Hypericumquartinianum* A.Rich.** – Habit: Shrub. Habitat: Grassland, woodland, gullies, river banks, 1750–2250 m. Vouchers: Chandler 432 (EA), Thomas AS 2607 (EA), Lugard 59 (EA), Tweedie 560, Tweedie DR 7 (EA), Gardner HM 2255 (EA), Jack C 39 (EA), Webb P 8751 (EA). Native distribution range: Ethiopia to S Tropical Africa, SW Arabian Peninsula.

**Hypericumrevolutumsubsp.keniense (Schweinf.) N.Robson** – Habit: Shrub or small tree. Habitat: Forest, 2250–3550 m. Vouchers: Thorold 2747 (EA), Humphreys, G 1026 €, Agnew ADQ; Agnew S 7284 (WAG), Rono et al. SAJIT-PR 0249 (EA, HIB). Native distribution range: E Central & E African Mountains.

***Hypericumrevolutum* Vahl** – Habit: Shrub or small tree. Habitat: Forest, 2250–3150 m. Vouchers: Mulugeta Kebede 205, 204, 185 (EA), Tweedie 63 (EA), Milne-Redhead E 181 (EA), Lugard EJ 472 (EA). Native distribution range: SE Nigeria to Bioko, Eritrea to South Africa, SW Arabian Peninsula.

***Hypericumroeperianum* G.W.Schimp. ex A.Rich.** – Habit: Shrub or small tree. Habitat: Forest, bamboo thickets, grassland, 1500–2900 m. Voucher: Tothill BH 2688 (EA). Native distribution range: Guinea, Nigeria to W Cameroon, Ethiopia to South Africa.

***Hypericumscioanum* Chiov.** – Habit: Herb. Habitat: Upland moor, 1830–3600 m. Vouchers: Thomas AS 570 (EA), Hedberg O 182 (EA), 4502 (EA), Arnstein Lyle 5730 (EA), Mwangangi OM 477 (EA), Thorold CA 2744 (EA), Muthama M & Gehrke B 128 (EA), Knox EB 3844 (EA). Native distribution range: Ethiopia to NE Zambia.

#### F72. LAMIACEAE

25 Genera, 79 species

***Achyrospermumschimperi* Perkins** – Habit: Shrub. Habitat: Montane forest undergrowth, forest edge, associated bushland, 1200–3000 m. Vouchers: Snowden JD 1017 (EA), Chandler 1000 (EA), Mwangangi OM 305 (EA). Native distribution range: Ethiopia to N Tanzania.

**^EX^*Aeollanthuspubescens* Benth.** – Habit: Herb. Habitat: Outcrop rock, woodland, 1800–2400 m. Voucher: Tweedie EM (1976) (REF). Native distribution range: W Tropical Africa to W Tanzania and Zambia.

***Aeollanthusrepens* Oliv.** – Habit: Herb. Habitat: Forest, wooded grassland, 1550–3000 m. Vouchers: Lind EM 449 (EA), Rono et al. SAJIT-PR 0040 (EA, HIB). Native distribution range: South Sudan to Mozambique.

***Aeollanthussuaveolens* Mart. ex Spreng.** – Habit: Herb. Habitat: Outcrop rock, cultivation, 1550–2400 m. Voucher: Rono et al. SAJIT-PR 0112 (EA, HIB). Native distribution range: Tropical & S Africa.

***Ajugaintegrifolia* Buch.-Ham.** – Habit: Herb. Habitat: Grassland, forest, woodland, forest, 1100–3400 m. Voucher: Rono et al. SAJIT-PR 0148 (EA, HIB). Native distribution range: NE & E Tropical Africa, Arabian Peninsula, Afghanistan to W New Guinea.

***Clerodendrumjohnstonii* Oliv.** – Habit: Shrub or small tree. Habitat: Montane forest, thicker or disturbed scrubland, 1300–2550 m. Voucher: Snowden JD 515 (EA). Native distribution range: SW Ethiopia to Zambia.

***Clerodendrumrotundifolium* Oliv.** – Habit: Shrub or small tree. Habitat: Wet savanna, bushland, rocky places, stream banks, short grassland, 1750–2250 m. Voucher: Agnew ADQ (2013) (REF). Native distribution range: Cameroon to South Sudan and Mozambique.

***Clinopodiumabyssinicum* Kuntze** – Habit: Herb. Habitat: Woodland, 1750–3000 m. Vouchers: Beentje HJ 1934 (UPS), Dummer RA 3609 (US). Native distribution range: NE Tropical Africa to N Tanzania, SW Arabian Peninsula.

*Clinopodiumkilimandschari (Gürke) Ryding – Habit: Trailing herb. Habitat: Alpine zones, 2900–4400 m. Vouchers: Tothill BH 2444 (EA), Wesche K 63 (EA), Hedberg O 264 (K), Tweedie 3 (K). Native distribution range: Ethiopia (Bale Mountains) to N Tanzania.

***Clinopodiumsimense* Kuntze** – Habit: Herb. Habitat: Upper montane forest edges, heath zone, 1700–3500 m. Vouchers: Dummer RA 3662 (EA), Beentje 1942 (EA). Native distribution range: Ethiopia to E Central & E Tropical Africa.

*Clinopodiumuhligii(Gürke)Rydingvar.uhligii – Habit: Herb/subshrub. Habitat: Heaths, woodland, upper forest edge, 1750–4180 m. Vouchers: Townsend CC 2325 (EA), Hedberg 4562 (EA), Dummer RA 3337 (EA), Wood GH 141 (EA), Lugard 363 (EA). Native distribution range: Tropical African Mountains.

**Clinopodiumuhligiivar.obtusifolium (Avetta) Ryding** – Habit: Herb/subshrub. Habitat: Woodland, forest, upper forest edge, 1750–3150 m. Voucher: Thomas AS 2687 (EA). Native distribution range: Tropical African Mountains.

***Coleusaegyptiacus* (Forssk.) A.J.Paton** – Habit: Herb or shrub. Habitat: Outcrop rock, old cultivation, 1300 m. Voucher: Chandler 590 (EA). Native distribution range: Eritrea to Tanzania, SW Arabian Peninsula.

***Coleusalpinus* Vatke** – Habit: Herb. Habitat: Woodland, 1750–2400 m. Voucher: Tweedie EM (1976) (REF). Native distribution range: Nigeria to Ethiopia and Malawi.

***Coleusbarbatus* (Andrews) Benth. ex G.Don** – Habit: Shrub. Habitat: shallow soil, 880–2950 m. Vouchers: Padwa JH 36 (EA), Fack WC (92), Templer JT 244 (EA) Bush RZ 255 (EA). Native distribution range: Eritrea to Tanzania, Arabian Peninsula Indian Subcontinent to S Central China.

***Coleusbojeri* Benth.** – Habit: Herb. Habitat: Rocky grassland, woodland, disturbed grounds, forest clearing, 1200–2670 m. Voucher: Agnew ADQ (2013) (REF). Native distribution range: Tropical & S Africa, Madagascar.

**Coleuscaninussubsp.flavovirens (Gürke) A.J.Paton** – Habit: Herb. Habitat: Rock outcrops, eroded grasslands, 1750–2250 m. Voucher: Tweedie 1736 (EA). Native distribution range: Ethiopia to S Africa.

***Coleuscomosus* Hochst. ex Gürke** – Habit: Herb or weak shrub. Habitat: acacia-commiphora bushland, 1000–2790 m. Voucher: Rono et al. SAJIT-PR 0207 (EA, HIB). Native distribution range: Eritrea to E Tanzania.

***Coleuscylindraceus* (Hochst. ex Benth.) A.J.Paton** – Habit: Herb. Habitat: Forest, grassland, outcrop rock, 1750–2400 m. Voucher: Tweedie EM (1976) (REF). Native distribution range: Tropical & S Africa, Arabian Peninsula, S India.

***Coleusdeflexifolius* (Baker) A.J.Paton** – Habit: Herb. Habitat: Wooded grassland, weed in disturbed grounds, 1705–2210 m. Voucher: Agnew ADQ (2013) (REF). Native distribution range: E Tropical Africa.

***Coleusdefoliatus* (Hochst. ex Benth.) A.J.Paton** – Habit: Herb. Habitat: Wooded grassland, Forest, 1700–2400 m. Vouchers: Gillett JB 20949 (EA, WAG), Gardener 2254. Native distribution range: Ethiopia to S Tropical Africa.

***Coleuskivuensis* Lebrun & L.Touss.** – Habit: Herb. Habitat: Stony bushland, thicket edges, 1500–2500 m. Voucher: Agnew ADQ (2013) (REF). Native distribution range: Eritrea to N Tanzania.

***Coleuslactiflorus* Vatke** – Habit: Shrub. Habitat: Rock outcrop, forest, woodland, wooded grassland, 1100–2250 m. Vouchers: Tweedie EM (1976) (REF), Agnew ADQ (2013) (REF). Native distribution range: Ethiopia to N & NW Tanzania.

***Coleuslanuginosus* Hochst. ex Benth.** – Habit: Herb. Habitat: Woodland, rock outcrop, grassland, forest, 1750–2400 m. Vouchers: Irwin PH 211 (EA), Rono et al. SAJIT-PR 0122 (EA, HIB). Native distribution range: Eritrea to N Tanzania, SW Arabian Peninsula

**Coleusmaculosussubsp.edulis (Vatke) A.J.Paton** – Habit: Herb. Habitat: Woodland, bamboo zone, forest, upper forest edge, 1750–3150 m. Voucher: Tweedie EM (1976) (REF). Native distribution range: Ethiopia to E Central & E Tropical Africa.

***Coleusmeyeri* (Gürke) A.J.Paton** – Habit: herb or shrub. Habitat: Montane forest, by streams, 1550–3100 m. Voucher: Dale U19 (EA). Native distribution range: W Tropical Africa to Ethiopia and Tanzania.

***Coleusniamniamensis* (Gürke) A.J.Paton** – Habit: Herb. Habitat: Swampy grassland, 900–2100 m. Vouchers: Lindblom sn (K) Tweedie 2918 (EA). Native distribution range: South Sudan to Kenya.

**^EX^*Coleusrotundifolius* (Poir.) A.Chev. & Perrot** – Habit: Herb. Habitat: Grassland, wet savanna, outcrop rock, 1400–2400 m. Voucher: Agnew ADQ (2013) (REF). Native distribution range: Tropical Asia.

***Coleusstachyoides* (Oliv.) E.A.Bruce** – Habit: Shrub. Habitat: Grassland or open acacia woodland. Voucher: Chandler 949 (EA). Native distribution range: Central African Republic to E Tropical Africa.

***Coleusstuhlmannii* (Gürke) A.J.Paton** – Habit: Herb. Habitat: Marshes, wet savanna, 1860–2400 m. Voucher: Agnew ADQ (2013) (REF). Native distribution range: Kenya to S Tropical Africa.

***Coleussylvestris* (Gürke) A.J.Paton & Phillipson** – Habit: Shrub. Habitat: Montane forest, bamboo zone, 1750–3300 m. Vouchers: Tweedie EM 882 (K), Mwangangi OM 374 (BR). Native distribution range: Tropical Africa, Madagascar.

***Coleustetradenifolius* (A.J.Paton) A.J.Paton** – Habit: Shrub. Habitat: Rocky places, cliffs 1350–2700 m. Voucher: Rono et al. SAJIT-PR 0113 (EA, HIB). Native distribution range: Cameroon, South Sudan to E Tropical Africa.

***Coleusxylopodus* (Lukhoba & A.J.Paton) A.J.Paton** – Habit: Subshrub. Habitat: Open Combretum woodland, rock outcrop, grassland prone to burning, 1200–2250 m. Voucher: Tweedie 1799 (EA). Native distribution range: S Ethiopia to Uganda.

***Equilabiumagnewii* (Lukhoba & A.J.Paton) Mwany. & A.J.Paton** – Habit: Herb. Habitat: Rocky grassland, 1700–1900 m. Voucher: Agnew ADQ (2013) (REF). Native distribution range: E Tropical Africa.

***Equilabiumcaespitosum* (Lukhoba & A.J.Paton) Mwany. & A.J.Paton** – Habit: Herb. Habitat: Rocky wooded grassland, 1850–2850 m. Voucher: Agnew ADQ (2013) (REF). Native distribution range: Kenya to N Tanzania.

***Equilabiumkamerunense* (Gürke) Mwany. & A.J.Paton** – Habit: shrub. Habitat: Montane forest margins, bamboo zone, 1500–2950 m. Voucher: Agnew ADQ (2013) (REF). Native distribution range: Nigeria to Cameroon, S Ethiopia to E Tropical Africa.

***Equilabiumlaxiflorum* (Benth.) Mwany., A.J.Paton & Culham** – Habit: Herb. Habitat: Woodland, forest, bamboo zone, 1750–3000 m. Voucher: Rono et al. SAJIT-PR 0182 (EA, HIB). Native distribution range: Ethiopia to S Africa.

***Equilabiumparvum* (Oliv.) Mwany. & A.J.Paton** – Habit: Herb. Habitat: Forest, grassland, woodland, 1700–2900 m. Vouchers: Tweedie 1332 (EA), Bogdan 1411 (EA). Native distribution range: Uganda to N Zambia.

***Equilabiumpauciflorum* (Baker) Mwany. & A.J.Paton** – Habit: Herb. Habitat: Woodland, thickets, grassland, rock outcrop, 1750–2100 m. Voucher: Tweedie EM (1976) (REF). Native distribution range: Uganda to Zambia.

***Fuerstiaafricana* T.C.E.Fr.** – Habit: Herb or subshrub. Habitat: Grassland, Acacia scrub, 1200–2550 m. Voucher: Lind EM 2584 (EA). Native distribution range: Ethiopia to Tanzania.

***Haumaniastrumcaeruleum* (Oliv.) P.A.Duvign. & Plancke** – Habit: Herb. Habitat: Wooded grassland, 2015–2325 m. Vouchers: Tweedie EM (1976) (REF), Agnew ADQ (2013) (REF). Native distribution range: Tropical Africa.

***Haumaniastrumvillosum* (Benth.) A.J.Paton** – Habit: Herb. Habitat: Moist outcrop rock, open woodland, open grassland, 1700–2400 m. Vouchers: Tweedie 2920, 4139 (EA). Native distribution range: Tropical Africa, Madagascar.

***Hoslundiaopposita* Vahl** – Habit: Shrub. Habitat: Roadside, forest, grassland, woodland, 750–2170 m. Voucher: Jack C 227 (EA). Native distribution range: Tropical & S Africa, Madagascar.

***Leonotisnepetifolia* (L.) R.Br.** – Habit: Herb. Habitat: Forest, weed of cultivation, 1000–2290 m. Voucher: Agnew ADQ (2013) (REF). Native distribution range: Africa, Indian Subcontinent.

***Leonotisocymifolia* (Burm.f.) Iwarsson** – Habit: Herb or shrub. Habitat: Forest, disturbed places, 1770–3360 m. Vouchers: Tweedie 8 (EA), Lugard C 113 (EA). Native distribution range: Eritrea to S Africa.

**Leonotisocymifoliavar.raineriana (Vis.) Iwarsson** – Habit: Shrub. Habitat: Woodland, lower and upper forest edge, grassland, 1750–3150 m. Voucher: Tweedie EM (1976) (REF). Native distribution range: Eritrea to S Africa.

*Leucasargentea Gürke – Habit: Herb or shrub. Habitat: Disturbed bushland, 1200–3350 m. Vouchers: Gillett JB20959 (EA), Thomas AS 554, 2382 (EA), Wood GH 123 (EA), Dummer RA 3581 (US), Rono et al. SAJIT-PR 0209 (EA, HIB). Native distribution range: Ethiopia to Kenya.

***Leucascalostachys* Oliv.** – Habit: Shrub. Habitat: Secondary bushlands, forest, grassland, 1600–2575 m. Vouchers: Hedberg 318 (EA). Native distribution range: S Ethiopia to Tanzania and DR Congo.

***Leucasdeflexa* Hook.f.** – Habit: Herb. Habitat: Open grassland, disturbed moist forest, 1600–2500 m. Voucher: Mwangangi OM 306 (EA). Native distribution range: Tropical Africa.

***Leucasmartinicensis* (Jacq.) R.Br.** – Habit: Herb. Habitat: Forest, in cultivation, 0–2200 m. Vouchers: Tweedie EM (1976) (REF), Agnew ADQ (2013) (REF). Native distribution range: Tropical & Subtropical Old World.

*Leucasmasaiensis Oliv. – Habit: Trailing herb. Habitat: Upland grassland, 1280–2735 m. Voucher: Agnew ADQ (2013) (REF). Native distribution range: Kenya to N Tanzania.

*Leucasmasaiensisvar.tricrenata (Bullock) Sebald – Habit: Herb. Habitat: Forest, 2250–3150 m. Vouchers: Tweedie 1525 (K), Tweedie EM 1425 (MO), Lugard CE|Lugard EJ 471 (MO), Irwin PH 221 (K), Wesche K 1437 (K), Sheil D; Katende T 2063 (K). Native distribution range: Kenya.

*Leucasmasaiensisvar.venulosa (Baker) Sebald – Habit: Herb. Habitat: Woodland, forest, 1750–3000 m. Voucher: Tweedie EM (1976) (REF). Native distribution range: Kenya to N Tanzania.

**^EX^*Leucastettensis* Vatke** – Habit: Herb. Habitat: Woodland, forest, 1750–3000 m. Voucher: Tweedie EM (1976) (REF). Native distribution range: Rwanda to S Tropical Africa.

***Menthaaquatica* L.** – Habit: Herb. Habitat: Marshes, 1950–2600 m. Vouchers: Tweedie EM (1976) (REF), Agnew ADQ (2013) (REF). Native distribution range: Africa, Europe to Central Siberia and W Asia.

***Micromeriaimbricata* C.Chr.** – Habit: Subshrub or woody herb. Habitat: Upland grassland, 1750–4300 m. Vouchers: Mwangangi OM 370 (BR), Rono et al. SAJIT-PR 0210 (EA, HIB). Native distribution range: Africa to Arabian Peninsula.

**Micromeriaimbricatavar.villosa (Elly Walther & K.H.Walther) Ryding** – Habit: Subshrub. Habitat: Heath zone, grasslands, bushlands, 1850–4100 m. Voucher: Wesche K 192 (EA). Native distribution range: Ethiopia to E Tropical Africa.

***Nepetaazurea* R.Br.** – Habit: Herb. Habitat: Bushed grassland, forest, upper forest edge, 1700–3700 m. Vouchers: Tothill BH 2315 (EA), Thomas 2656 (EA), Hedberg 4530 (EA), Tweedie EM 880 (K), Synge PM 854 (BR), Dummer RA 3307 (K), Battiscombe E 673 (US). Native distribution range: NE & E Tropical Africa.

***Ocimumafricanum* Lour.** – Habit: Herb. Habitat: Damp disturbed grounds, 1800–2250 m. Voucher: Hancock 2388 (EA). Native distribution range: Tropical & Subtropical Old World.

***Ocimumgratissimum* L.** – Habit: Shrub. Habitat: Bushland, forest, woodland, 1100–2400 m. Voucher: Hurni MPP? 215 (EA). Native distribution range: Tropical & Subtropical Old World.

***Ocimumkilimandscharicum* Gürke** – Habit: Shrub. Habitat: Wet savanna, woodland, 1750–2400 m. Voucher: Webster MVB 8974 (EA). Native distribution range: Ethiopia to E Tropical Africa.

***Ocimumlamiifolium* Hochst.** – Habit: Herb or shrub. Habitat: Montane forest edges, grassland, 1550–2500 m. Vouchers: Irwin PH 213 (EA), Rono et al. SAJIT-PR 0073 (EA, HIB). Native distribution range: Cameroon to Eritrea and Zambia.

^**EX**^***Orthosiphonrubicundus* Benth.** – Habit: Herb. Habitat: Forest, grassland, woodland, 1750–2400 m. Voucher: Tweedie EM (1976) (REF). Native distribution range: Himalaya to Hainan and Indo-China.

***Orthosiphonschimperi* Benth.** – Habit: Herb. Habitat: Wooded grassland, grassland, 1600–2635 m. Voucher: Agnew ADQ (2013) (REF). Native distribution range: Tropical & S Africa.

***Orthosiphonthymiflorus* (Roth) Sleesen** – Habit: Herb. Habitat: Wooded grassland, forest, bushland, 1750–2400 m. Voucher: Agnew ADQ (2013) (REF). Native distribution range: Africa, India to Indo-China, Central.

***Platostomarotundifolium* (Briq.) A.J.Paton** – Habit: Herb. Habitat: Swampy grassland, 1500–2800 m. Voucher: Agnew ADQ (2013) (REF). Native distribution range: Tropical & S Africa.

***Premnaangolensis* Gürke** – Habit: Tree. Habitat: Forest edge, bushland, grassland, 900–1800 m. Voucher: Snowden JD 887 (EA). Native distribution range: Tropical Africa.

***Rothecamyricoides* (Hochst.) Steane & Mabb.** – Habit: Subshrub or shrubby herb or small tree. Habitat: Grassland, scrub, forest clearing, grazed bushland, 1180–2400 m. Vouchers: Tweedie DR 159 (BR), Eggeling WJ 2443 (BR), Rono et al. SAJIT-PR 0078 (EA, HIB). Native distribution range: Eritrea to S Africa.

**^EX^*Salviacoccinea* Buc’hoz ex Etl.** – Habit: Herb. Habitat: Forest, 500–2000 m. Voucher: Padwa JH 28 (EA). Native distribution range: SE America to N Argentina.

***Salviamerjamie* Forssk.** – Habit: herb. Habitat: Burnt grassland, 2300–4100 m. Vouchers: Lye 1457 (EA), Wesche K 101 (EA), Adane Asefa (O). Native distribution range: Eritrea to N Tanzania, SW Arabian Peninsula.

***Salvianilotica* Juss. ex Jacq.** – Habit: Herb. Habitat: Grassland and forest edges, 1800–3700 m. Vouchers: Hedberg O 3600 (EA), Tothill BH 2386 (EA), Stein W Bie (UPS). Native distribution range: Eritrea to S Tropical Africa.

**Scutellariaschweinfurthiisubsp.paucifolia (Baker) A.J.Paton** – Habit: Herb. Habitat: Wooded grassland, grassland subject to burning, 900–2600 m. Voucher: Snowden JD 1048 (EA). Native distribution range: Tropical Africa.

***Scutellariaviolascens* Gürke** – Habit: Herb. Habitat: Bushland, grassland, montane forest, 1200–2400 m. Voucher: Irwin PH 150 (EA). Native distribution range: Tropical Africa.

***Stachysaculeolata* Hook.f.** – Habit: Trailing herb. Habitat: Montane forest, alpine zone, 2920–3650 m. Voucher: Beentje HJ 1991 (WAG). Native distribution range: Tropical Africa.

**Stachysaculeolatavar.aculeolata** – Habit: Herb. Habitat: Bamboo/ericaceous belt, upper edge of forest, 1400–3750 m. Vouchers: Tweedie 1427 (EA), Tothill BH 2379 (EA). Native distribution range: Tropical Africa.

***Stachysargillicola* Sebsebe** – Habit: Herd or shrub. Habitat: Grassland, 1500–2550 m. Voucher: Taylor 3868 (EA). Native distribution range: NE & E Tropical Africa.

***Tinneaaethiopica* Kotschy ex Hook.f.** – Habit: Shrub or herb. Habitat: Grassland, wooded grassland, 1750–2300 m. Vouchers: Hedberg O (UPS), Rono et al. SAJIT-PR 0041 (EA, HIB). Native distribution range: Tropical Africa.

***Vitexdoniana* Sweet** – Habit: Tree. Habitat: Combretum woodland, elephant grassland, wet forest, 0–1950 m. Voucher: Chater-Jack 2821 (EA). Native distribution range: Tropical Africa, W Indian Ocean.

***Vitexfischeri* Gürke** – Habit: Tree. Habitat: Wooded grassland, thickets, 1200–2080 m. Vouchers: Ahenda J sn (WAG), Ahenda J; Meroka D sn (EA, WAG), Maesen LJG van der; Ahenda J 6212 (WAG). Native distribution range: South Sudan to S Tropical Africa.

#### F73. LENTIBULARIACEAE

1 Genus, 4 species

***Utriculariaarenaria* A.DC.** – Habit: Herb. Habitat: Grassland, seasonal swamps, outcrop rock, 387–2950 m. Vouchers: Tweedie EM 339 (K), 868(K), 578 (K). Native distribution range: Tropical & S Africa, Madagascar, Central India.

***Utriculariagibba* L.** – Habit: Aquatic/subaquatic herb. Habitat: Streams, fresh water pools, outcrop rock, 0010–2550 m. Vouchers: Tweedie EM (1976) (REF), Agnew ADQ (2013) (REF). Native distribution range: Tropical & Subtropical Old World, America.

***Utriculariapentadactyla* P.Taylor** – Habit: Herb. Habitat: Outcrop rock, grassland, 1800–2400 m. Voucher: Tweedie EM 1789 (K). Native distribution range: Central Ethiopia to S Tropical Africa.

***Utriculariareflexa* Oliv.** – Habit: Aquatic herb. Habitat: Fresh water pools, 1860–2790 m. Voucher: Agnew ADQ (2013) (REF). Native distribution range: Tropical Africa to N Botswana, Central Madagascar.

#### F74. LINACEAE

1 Genus, 2 species

***Linumkeniense* T.C.E.Fr.** – Habit: Herb. Habitat: Bushland in shade, roadside, streamside, 2200–3360 m. Vouchers: Hedberg 142 (EA). Native distribution range: S Ethiopia to E Tropical Africa.

***Linumvolkensii* Engl.** – Habit: Herb. Habitat: Grassland, by streams, 1100–2900 m. Vouchers: Tweedie EM (1976) (REF), Agnew ADQ (2013) (REF). Native distribution range: Cameroon, Ethiopia to S Tropical Africa.

#### F75. LINDERNIACEAE

6 Genera, 12 species

^**EX**^***Bonnayaciliata* Spreng.** – Habit: Herb. Habitat: Outcrop rock, 1800–2400 m. Voucher: Tweedie EM (1976) (REF). Native distribution range: Tropical & Subtropical Asia to N Australia.

***Craterostigmaabyssinicum* (Engl.) Eb.Fisch., Schäferh. & Kai Müll.** – Habit: Herb. Habitat: Rocky grasslands, woodland, grassland, riverine forest, 1750–2500 m. Vouchers: Garderner 2258 (EA), Tweedie 295 (EA). Native distribution range: Nigeria to Ethiopia and Tanzania.

***Craterostigmahirsutum* S.Moore** – Habit: Herb. Habitat: Outcrop rock, 350–2100 m. Vouchers: Tweedie EM (1976) (REF), Agnew ADQ (2013) (REF). Native distribution range: Ethiopia to S Tropical Africa.

*Craterostigmalindernioides E.A.Bruce – Habit: Herb. Habitat: Wet seasonal marshes, open grassland ponds, 1300–1600 m. Voucher: Lind EM 238 (EA). Native distribution range: E Uganda to Tanzania.

***Craterostigmanewtonii* (Engl.) Eb.Fisch., Schäferh. & Kai Müll.** – Habit: Herb. Habitat: Marshes, 1700–2200 m. Vouchers: Rono et al. SAJIT-PR 0029, SAJIT-PR 0115 (EA, HIB). Native distribution range: Togo to Ethiopia and S Tropical Africa.

***Craterostigmaplantagineum* Hochst.** – Habit: Herb. Habitat: Outcrop rock, 1800–2400 m. Voucher: Tweedie 308 (EA). Native distribution range: Tropical & S Africa, SW Arabian Peninsula, India.

***Craterostigmapumilum* Hochst.** – Habit: Herb. Habitat: Montane grassland, outcrop rock, upland grassland, 1800–3300 m. Vouchers: Bamps PRJ 6505 (WAG), Rono et al. SAJIT-PR 0018, SAJIT-PR 0091 (EA, HIB), Snowden JD 934 (EA), Norman 261 (EA). Native distribution range: Eritrea to Botswana, Arabian Peninsula.

***Crepidorhopalonwhytei* (Skan) Eb.Fisch.** – Habit: Herb. Habitat: Marshes, montane forests, 1300–2500 m. Voucher: Agnew ADQ (2013) (REF). Native distribution range: SW Ethiopia to E Central & E Tropical Africa.

***Linderniaparviflora* Haines** – Habit: Herb. Habitat: Ephemeral pools on rocky outcrop, 1200–2000 m. Voucher: Rono et al. SAJIT-PR 0026 (EA, HIB). Native distribution range: Africa, Indian Subcontinent to Indo-China.

***Linderniellapulchella* (Skan) Eb.Fisch., Schäferh. & Kai Müll.** – Habit: Herb. Habitat: Pools on outcrop, outcrop rocks, 400–2300 m. Vouchers: Lind EM 296 (EA), Rono et al. SAJIT-PR 0038, SAJIT-PR 0115 (EA, HIB). Native distribution range: Ethiopia to S Africa.

***Linderniellawilmsii* (Engl.) Eb.Fisch., Schäferh. & Kai Müll.** – Habit: Herb. Habitat: Granite rock outcrop, 900–2300 m. Voucher: Tweedie 1192 (EA). Native distribution range: W Central Kenya to S Africa.

***Vandelliadiffusa* L.** – Habit: Herb. Habitat: Woodland, 1750–2400 m. Voucher: Tweedie EM (1976) (REF). Native distribution range: NE Turkey to Caucasus, Tropical Africa, Madagascar.

#### F76. LORANTHACEAE

7 Genera, 9 species

***Agelanthusbrunneus* (Engl.) Tiegh.** – Habit: Shrub. Habitat: Forest, swamps, riverine forest, 1000–1800 m. Vouchers: Tweedie 1951, Tweedie EM 925 (K). Native distribution range: Tropical Africa.

**Agelanthusentebbensis (Sprague) Polhill & Wiens – Habit: Shrub. Habitat: Forest, 1100–2100 m. Vouchers: Snowden JD 1053 (MO), Chater-Jack EM 372 (MO). Native distribution range: Uganda to W Kenya.

***Englerinawoodfordioides* (Schweinf. ex Engl.) Balle** – Habit: Shrub. Habitat: Montane forest, 1350–3050 m. Vouchers: Jackson L 314 (EA), Tweedie EM, Snowden JD 1194 (EA), Tweedie EM 1269 (K), Katende 1121 (K), Trapson Clair 1760 (EA), Jackson THE 314, (EA), Polhill RM 4863 (WAG, EA), Mwangangi OM 445 (BR), Rono et al. SAJIT-PR 0054, SAJIT-PR 0065 (EA, HIB). Native distribution range: Ethiopia to E Central & E Tropical Africa.

***Erianthemumdregei* Tiegh.** – Habit: Shrub. Habitat: Forest edges, bushlands, 1750–2590 m. Voucher: Tweedie EN 863 (K). Native distribution range: Eritrea to S Africa.

***Oncocalyxsulfureus* (Engl.) Wiens & Polhill** – Habit: Shrub. Habitat: Upland forest, 0–2680 m. Voucher: Agnew ADQ (2013) (REF). Native distribution range: Kenya to N Tanzania.

***Phragmantherausuiensis* (Oliv.) M.G.Gilbert** – Habit: Shrub. Habitat: Montane forest, 1150–2600 m. Vouchers: Padwa JH 35 (EA), Chetea J 272 (EA), Jackson THE 2993 (EA), Napier ER 2529 (EA), Kokwaro JO 2506 (EA), Tweedie EM 1131 (K), Rono et al. SAJIT-PR 0067 (EA, HIB). Native distribution range: Cameroon to SW Ethiopia and Zambia.

***Plicosepaluscurviflorus* Tiegh.** – Habit: Shrub. Habitat: Bushland, wooded grassland, 500–2320 m. Vouchers: Tweedie EM 1153 (K, UPS). Native distribution range: SE Egypt to E Central & E Tropical Africa.

***Tapinanthusbuvumae* (Rendle) Danser** – Habit: Shrub. Habitat: Wooded grasslands, 750–1200 m. Voucher: Tweedie 118 (EA). Native distribution range: E Central & E Tropical Africa.

***Tapinanthusconstrictiflorus* (Engl.) Danser** – Habit: Shrub. Habitat: Forest, 1750–2250 m. Voucher: Tweedie EM (1976) (REF). Native distribution range: Gabon to Kenya and Angola.

#### F77. LYTHRACEAE

3 Genera, 6 species

**^EX^*Ammanniaaegyptiaca* Willd.** – Habit: Herb. Habitat: Forest, grassland, outcrop rock, 1750–2400 m. Voucher: Tweedie EM (1976) (REF). Native distribution range: Egypt to Angola, India.

***Ammanniaprieuriana* Guill. & Perr.** – Habit: herb. Habitat: Grassland, pools on rock outcrop, cultivation, 1800–2250 m. Voucher: Tweedie 1492 (EA). Native distribution range: Tropical & S Africa to Israel.

***Ammanniaschinzii* (Koehne) S.A.Graham & Gandhi** – Habit: Herb or subshrub. Habitat: Outcrop rock, 1800–2400 m. Voucher: Syme 317 (EA). Native distribution range: S Tropical & S Africa.

***Ammanniawormskioldii* Fisch. & C.A.Mey.** – Habit: herb. Habitat: Roadsides, pools on rock outcrop, 1800–2400 m. Voucher: Tweedie 3473 (EA). Native distribution range: W Ethiopia to Namibia.

***Lythrumrotundifolium* Hochst. ex A.Rich.** – Habit: Aquatic or semiaquatic herb. Habitat: Along streams in open places, 1700–3300 m. Voucher: Rono et al. SAJIT-PR 0259 (EA, HIB). Native distribution range: Ethiopia to Malawi.

***Rotalarepens* (Hochst.) Koehne** – Habit: Aquatic herb. Habitat: Forest, spray of waterfall, 1200–1900 m. Voucher: Wallace JRM 10a (EA). Native distribution range: Ethiopia to S DR Congo

#### F78. MALVACEAE

13 Genera, 36 species

***Abutilonlongicuspe* Hochst. ex A.Rich.** – Habit: Shrub. Habitat: Upland forest edges, 1650–3300 m. Vouchers: Katende; Sheil 1865 (K), Sheil; Van Heist 1980 (K), Irwin PH 46 (BR), Bickford N 15 (EA), Jack C 178 (EA). Native distribution range: NE Sudan to S Tropical Africa.

***Abutilonmauritianum* (Jacq.) Medik.** – Habit: Herb or subshrub. Habitat: Forest edge, disturbed grounds, 0–2300 m. Vouchers: Padwa JH 33, Tweedie DR 177 (BR). Native distribution range: Tropical & S Africa, Arabian Peninsula, Comoros.

***Dombeyaburgessiae* Gerrard ex Harv.** – Habit: Tree or shrub. Habitat: Woodland, lower forest edge, 1750–2400 m. Vouchers: Oxtoby E 6 (EA), Dale IR 808 (EA), Fairbairn G 799 (EA), Jack C 13 (EA), Rono et al. SAJIT-PR 0049 (EA, HIB). Native distribution range: South Sudan to S Africa.

***Dombeyarotundifolia* Planch.** – Habit: Shrub or small tree. Habitat: Woodland, lower forest edge, 1750–2400 m. Voucher: Eggeling 2487 (EA). Native distribution range: S Ethiopia to S Africa.

***Dombeyatorrida* (J.F.Gmel.) Bamps** – Habit: Tree. Habitat: Forest, bamboo woodland, 2250–3050 m. Voucher: Tweedie EM (1976) (REF). Native distribution range: Central African Republic to NE & E Tropical Africa, Arabian Peninsula.

***Grewiasimilis* K.Schum.** – Habit: Shrub or small tree. Habitat: Grassland, woodland, thickets, 1750–2250 m. Voucher: Tweedie EM (1976) (REF). Native distribution range: Ethiopia to E Central & E Tropical Africa.

***Grewiastolzii* Ulbr.** – Habit: Shrub or small tree. Habitat: Riverine forest, woodland, 1750–2250 m. Voucher: Jackson 436 (EA). Native distribution range: South Sudan to S Tropical Africa.

***Grewiatrichocarpa* Hochst. ex A.Rich.** – Habit: Shrub or small tree. Habitat: Acacia bush, riverine forest, grassland, 900–2150 m. Voucher: Templer JT 67 (EA). Native distribution range: Eritrea to N & Central Tanzania, SW Arabian Peninsula.

***Hibiscusaethiopicus* L.** – Habit: Herb. Habitat: Grassland, bushland, 1750–2250 m. Voucher: Symes 360 (EA). Native distribution range: Eritrea to E Central & E Tropical Africa, S Africa, and Yemen.

***Hibiscusaponeurus* Sprague & Hutch.** – Habit: Herb. Habitat: Grassland, 250–2250 m. Vouchers: Tweedie EM (1976) (REF), Agnew ADQ (2013) (REF). Native distribution range: Djibouti to N Mozambique.

***Hibiscusarticulatus* Hochst. ex A.Rich.** – Habit: Herb. Habitat: Seasonally water-logged grasslands, 1750–2250 m. Voucher: Tweedie 1314 (EA). Native distribution range: W Tropical Africa to Ethiopia and Botswana.

***Hibiscuscalyphyllus* Cav.** – Habit: Shrub or herb. Habitat: Woodland, forest, 700–2250 m. Vouchers: Tweedie EM (1976) (REF), Agnew ADQ (2013) (REF). Native distribution range: Eritrea to S Africa, W Indian Ocean.

***Hibiscuscannabinus* L.** – Habit: Herb. Habitat: Grassland, 600–2600 m. Vouchers: Tweedie EM (1976) (REF), Agnew ADQ (2013) (REF). Native distribution range: Tropical & S Africa, SW Arabian Peninsula.

***Hibiscusdiversifolius* Jacq.** – Habit: Small tree, shrub or herb. Habitat: Forest, swamps, savanna, 1330–2800 m. Vouchers: Tweedie EM (1976) (REF), Agnew ADQ (2013) (REF). Native distribution range: Africa.

***Hibiscusfuscus* Garcke** – Habit: Woody herb or shrub. Habitat: Grassland, bushland, thickets, 1400–2650 m. Voucher: Rono et al. SAJIT-PR 0062 (EA, HIB). Native distribution range: Djibouti to S Africa.

***Hibiscusludwigii* Eckl. & Zeyh.** – Habit: Shrub or herb. Habitat: Roadside, forest, 1800–3000 m. Vouchers: Snowden JD 818 (EA), Tiyoy L 1331 (K). Native distribution range: Ethiopia to Zimbabwe, South Africa.

***Hibiscusmacranthus* Hochst. ex A.Rich.** – Habit: Shrub or woody herb. Habitat: Forest grassland, 1500–2900 m. Voucher: Symes 387 (EA). Native distribution range: Cameroon, Eritrea to Tanzania.

***Hibiscusvitifolius* L.** – Habit: Shrub or herb. Habitat: Forest, woodland, 420–2250 m. Voucher: Lugard 454 (EA). Native distribution range: Tropical & Subtropical Old World.

***Kosteletzkyaadoensis* Mast.** – Habit: Herb or subshrub (procumbent, climbing or scrambling). Habitat: Montane forests, grassland thickets, 1200–2700 m. Vouchers: Tweedie EM (1976) (REF), Agnew ADQ (2013) (REF). Native distribution range: Tropical Africa, Madagascar.

***Kosteletzkyabegoniifolia* Ulbr.** – Habit: Herb or subshrub. Habitat: Upland montane forest, swamps, marshes, moist thickets, 1450–2400 m. Voucher: Tweedie 820 (EA). Native distribution range: Cameroon, Ethiopia to South Tropical Africa

**^EX^*Malvaverticillata* L.** – Habit: Herb. Habitat: Bushlands, bamboo zones, alpine zones, woodland, forest, 1500–4000 m. Voucher: Tweedie DR 6 (EA). Native distribution range: NE Tropical Africa, Central Asia to Korea.

***Melhaniavelutina* Forssk.** – Habit: Herb or subshrub. Habitat: Riverine forest, grassland, 0010–2000 m. Voucher: Agnew ADQ (2013) (REF). Native distribution range: Eritrea to Angola, SW Arabian Peninsula.

***Melochiacorchorifolia* L.** – Habit: Herb or subshrub. Habitat: Woodland, forest edge, 1750–2400 m. Voucher: Tweedie EM (1976) (REF). Native distribution range: Tropical & Subtropical Old World.

***Pavoniaburchellii* (DC.) R.A.Dyer** – Habit: Herb. Habitat: Woodland, forest edge, 1750–2400 m. Voucher: Rono et al. SAJIT-PR 0059 (EA, HIB). Native distribution range: Egypt to S Africa.

***Pavoniakilimandscharica* Gürke** – Habit: Shrub. Habitat: Bamboo zone, *Ocotea* and *Podocarpus* forest, 1200–3000 m. Voucher: Irwin PH 107 (EA). Native distribution range: Bioko, Cameroon, Ethiopia to E Central & E Tropical Africa.

***Pavoniaschimperiana* Hochst. ex A.Rich.** – Habit: Shrubby herb or shrub. Habitat: Grassland, swamp edges, thickets forest, 1100–2400 m. Voucher: Tweedie 1374 (EA). Native distribution range: Tropical Africa.

***Pavoniaurens* Cav.** – Habit: Shrubby herb or shrub. Habitat: Forest, 1200–3000 m. Vouchers: Ross R 1340 (BR), 1345 (BR). Native distribution range: Tropical & S Africa, Madagascar.

**Sidarhombifoliavar.serratifolia (R.Wilczek & Steyaert) Verdc.** – Habit: Suffrutex shrub. Habitat: Forest, disturbed places, 900–2600 m. Vouchers: Tweedie EM (1976) (REF), Agnew ADQ (2013) (REF). Native distribution range: Ethiopia to S Africa.

***Sidaschimperiana* Hochst. ex A.Rich** – Habit: Shrub. Habitat: Grassland, bushland, overgrazed areas, roadsides, 1200–2700 m. Voucher: Lind EM 292 (EA). Native distribution range: Eritrea to N Tanzania.

***Sidaternata* L.f.** – Habit: Herb or undershrub. Habitat: Montane forest, bamboo, pathsade, 1350–3280 m. Voucher: Naiga 503 (K). Native distribution range: Eritrea to S Africa.

***Sparrmanniaricinocarpa* Kuntze** – Habit: Shrub. Habitat: Forest, 1200–3350 m. Voucher: Dummer RA 3586 (US). Native distribution range: E Cameroon to Eritrea and S Africa, Madagascar.

***Triumfettabrachyceras* K.Schum.** – Habit: Shrub. Habitat: Woodland, forest edge, 1750–3000 m. Voucher: Dummer RA 3629 (EA). Native distribution range: Ethiopia to Burundi and Tanzania.

***Triumfettacordifolia* A.Rich.** – Habit: Woody herb or shrub. Habitat: Montane forest, woodland, 1000–3000 m. Vouchers: Tweedie 1533 (EA), Kahuko 14 (EA). Native distribution range: W & W Central Tropical Africa.

***Triumfettarhomboidea* Jacq.** – Habit: Herb or undershrub. Habitat: Forest, disturbed places, roadsides, 1100–2200 m. Vouchers: Chair-Jack 70 (EA), Goldschmidt W 37 (EA). Native distribution range: Tropical & Subtropical Old World.

***Triumfettatomentosa* Bojer** – Habit: Shrub. Habitat: Forest clearings and old cultivation, 350–2100 m. Voucher: Jack C 155 (EA). Native distribution range: Tropical Africa to Namibia.

***Urenalobata* L.** – Habit: Herb. Habitat: Disturbed cultivations, roadsides, 1250–2300 m. Voucher: Rono et al. SAJIT-PR 0192 (EA, HIB). Native distribution range: Tropical & Subtropical.

#### F79. MELASTOMATACEAE

3 Genera, 4 species

***Antherotomanaudinii* Hook.f.** – Habit: Herb. Habitat: Shallow soils by streams, outcrop rock, 500–2300 m. Vouchers: Tweedie EM (1976) (REF), Agnew ADQ (2013) (REF). Native distribution range: Tropical & S Africa, Comoros, Madagascar.

***Antherotomasenegambiensis* (Guill. & Perr.) Jacq.-Fél.** – Habit: Herb. Habitat: Roadside, disturbed grassland, outcrop rock, 1350–2650 m. Vouchers: Tweedie EM (1976) (REF), Agnew ADQ (2013) (REF). Native distribution range: Tropical Africa.

***Argyrellacanescens* (Graham) Harv.** – Habit: Woody herb. Habitat: Burnt or swampy grassland, 800–2200 m. Voucher: Jack C 89 (EA). Native distribution range: Nigeria to Ethiopia and S Africa.

***Dissotisspeciosa* Taub.** – Habit: Woody herb or shrub. Habitat: Streamside, marshes, 1000–2250 m. Vouchers: Tweedie EM (1976) (REF), Agnew ADQ (2013) (REF). Native distribution range: Uganda to NE Zambia.

#### F80. MELIACEAE

4 Genera, 6 species

***Ekebergiacapensis* Sparrm.** – Habit: Tree. Habitat: Woodland, upper forest adge, 1750–3000 m. Vouchers: Styles BT 315 (EA, MO), Snowden JD 965 (MO), Gillett JB 20948 (MO), Rono et al. SAJIT-PR 0105 (EA, HIB). Native distribution range: Tropical & S Africa.

***Entandrophragmaangolense* C.DC.** – Habit: Tree. Habitat: Forest, 1100–1830 m. Vouchers: Styles 213 (EA), Eggeling 5602 (EA). Native distribution range: Tropical Africa.

***Entandrophragmaexcelsum* Sprague** – Habit: Tree. Habitat: Montane Forest, riverine forest, 1525–2150 m. Voucher: Styles 319 (EA). Native distribution range: Uganda to N Malawi.

***Lepidotrichiliavolkensii* (Gürke) J.-F.Leroy** – Habit: Tree. Habitat: Forest, 1550–2600 m. Vouchers: Style 180 (EA), Rono et al. SAJIT-PR 0046 (EA, HIB). Native distribution range: Ethiopia to Burundi and Malawi.

***Turraeafloribunda* Hochst.** – Habit: Shrub or tree. Habitat: Woodland, 1750–2400 m. Voucher: Tweedie EM (1976) (REF). Native distribution range: South Sudan to S Africa.

***Turraeaholstii* Gürke** – Habit: Tree. Habitat: Forest, 1750–2500 m. Vouchers: Bamps PRJ 6510 (WAG), Smyes 264 (EA), Rono et al. SAJIT-PR 0006, SAJIT-PR 0194 (EA, HIB). Native distribution range: Ethiopia to Malawi.

#### F81. MELIANTHACEAE

1 Genus, 2 species

***Bersamaabyssinica* Fresen.** – Habit: Tree or shrub. Habitat: Grassland, 1140–2550 m. Voucher: Rono et al. SAJIT-PR 0217 (EA, HIB). Native distribution range: Tropical Africa.

**Bersamaabyssinicasubsp.paullinioides (Planch.) Verdc.** – Habit: Tree. Habitat: Thickets, upland grassland, 1800–2400 m. Vouchers: Lugard 325 (EA), Rono et al. SAJIT-PR 0056 (EA, HIB). Native distribution range: Tropical Africa.

#### F82. MENISPERMACEAE

3 Genera, 5 species

***Cissampelosmucronata* A.Rich.** – Habit: Liane. Habitat: Swamps, forest edge, 1750–2400 m. Voucher: Tweedie EM 1304 (K). Native distribution range: Tropical & S Africa.

***Cissampelospareira* L.** – Habit: Liane. Habitat: Rain forest, 1750–3000 m. Vouchers: Beentje HJ 1971 (WAG), Rono et al. SAJIT-PR 0052 (EA, HIB). Native distribution range: Tropics & Subtropics.

***Hyalosepalumcaffrum* (Miers) Troupin** – Habit: Liane. Habitat: Forest, rock outcrop, bushland, 1750–2400 m. Vouchers: Snowden JD 1034 (EA), Tweedie EM 3126 (K). Native distribution range: Ethiopia to S Africa.

***Stephaniaabyssinica* Walp.** – Habit: Liane. Habitat: River edges in forest, grassland, woodland, 2120–3300 m. Vouchers: Lugard 447 (EA), Gillett JB 20947 (WAG), Dummer RA 3641 (EA), Andersen R 364 (S), Granvik H 134 (S), Rono et al. SAJIT-PR 0051 (EA, HIB). Native distribution range: Tropical & S Africa.

***Stephaniacyanantha* Welw. ex Hiern** – Habit: Liane. Habitat: Swamps, volcanic lava, 3000–3150 m. Vouchers: Major EJ; Lugard Cyril 502a (K) 502 (EA). Native distribution range: Sierra Leone, Bioko to Ethiopia and S Tropical Africa.

#### F83. MONIMIACEAE

1 Genus, 1 species

***Xymalosmonospora* Baill.** – Habit: Shrub or small to medium size tree. Habitat: Forest, 2250–3000 m. Vouchers: Mwangangi OM 417 (MO), Wesche K 1850 (MO), Rono et al. SAJIT-PR 0187 (EA, HIB). Native distribution range: SE Nigeria to W Cameroon, Gulf of Guinea Islands, S Sudan to South Africa.

#### F84. MORACEAE

3 Genera, 7 species

**Antiaristoxicariavar.usambarensis (Engl.) C.C.Berg** – Habit: Tree. Habitat: Forest, riverine or semi-swampy forest, up to 1700 m. Voucher: St Clair Thomson in Eggeling 3957 (EA). Native distribution range: Uganda to N Zambia.

***Dorsteniabarnimiana* Schweinf.** – Habit: Herb. Habitat: Stony grasses, 1080–2170 m. Voucher: Adamson J 450 (EA). Native distribution range: Cameroon to Yemen and Zambia.

***Dorsteniabenguellensis* Welw.** – Habit: Herb. Habitat: Woodland, wooded grassland, 1000–2450 m. Voucher: Jack 262 (EA). Native distribution range: Cameroon to W Ethiopia and S Tropical Africa.

***Ficusingens* Miq.** – Habit: Tree. Habitat: Forest, grassland, 1750–2250 m. Voucher: Walter 6 (EA). Native distribution range: S Algeria to Tropical & S Africa, Arabian Peninsula.

***Ficusnatalensis* Hochst.** – Habit: Tree or shrub. Habitat: Forest, woodland, riverine forest, 1750–3000 m. Voucher: Tweedie EM (1976) (REF). Native distribution range: Ivory Coast to Sudan and S Africa.

***Ficussur* Forssk.** – Habit: Tree. Habitat: Forest, riverine forest, wooded grassland, 1750–2400 m. Voucher: Tweedie EM (1976) (REF). Native distribution range: Africa to Arabian Peninsula.

***Ficussycomorus* L.** – Habit: Tree. Habitat: Forest, woodland, outcrop rock, 1750–2400 m. Voucher: Tweedie EM (1976) (REF). Native distribution range: Africa to Syria.

#### F85. MYRICACEAE

1 Genus, 2 species

***Myricameyerijohannis* Engl.** – Habit: Shrub. Habitat: Upland grassland, moor, ericaceous zone, (1950)–2700–3700 m. Vouchers: Harnilton & Perrott 756/433 (EA), Snowden JD 814 (EA), Wesche K 1182 (EA). Native distribution range: Central Kenya to N Tanzania.

***Myricasalicifolia* Hochst. ex A.Rich.** – Habit: Shrub or tree. Habitat: Forest, 3150–4321 m. Voucher: Tweedie EM (1976) (REF). Native distribution range: Cameroon, Eritrea to Burundi, Madagascar, SW Arabian Peninsula.

#### F86. MYRTACEAE

1 Genus, 2 species

***Syzygiumcordatum* Hochst.** – Habit: Tree. Habitat: Forest, grassland, 1750–2400 m. Vouchers: Edward Mwangi 216 (EA), Jackson THE 345 (EA), Jack C 207 (EA), Eggeling WJ 2483 (EA, MO). Native distribution range: Uganda to S Africa.

***Syzygiumguineense* DC.** – Habit: Tree, shrub or pyrophytic subshrub. Habitat: Riverine forest, woodland, 1750–2250 m. Vouchers: Taylor 3847 (MO), Naiper 2528 (MO), Snowden JD 1080 (MO). Native distribution range: Tropical & S Africa, SW Arabian Peninsula.

#### F87. NYMPHAEACEAE

1 Genus, 2 species

***Nymphaeanouchali* Burm.f.** – Habit: Aquatic herb. Habitat: Permanent water bodies, 0–2680 m. Voucher: Agnew ADQ (2013) (REF). Native distribution range: Tropical & Subtropical Old World to N Australia.

**Nymphaeanouchalivar.caerulea (Savigny) Verdc.** – Habit: Aquatic herb. Habitat: Permanent water bodies, 1750–2250 m. Voucher: Tweedie EM (1976) (REF). Native distribution range: Egypt to S Africa, S Arabian Peninsula, Comoros.

#### F88. OCHNACEAE

1 Genus, 4 species

***Ochnaafzelii* R.Br. ex Oliv.** – Habit: Shrub or tree. Habitat: Grassland on rocky hills, bushland, forest, 1150–1350 m. Voucher: Snowden JD 873 (EA). Native distribution range: Tropical Africa.

***Ochnaholstii* Engl.** – Habit: Shrub or small tree. Habitat: Forest, riverine forest, thickets, woodlands, 2250–3000 m. Vouchers: Dale IR 3121 (EA, BR), Rono et al. SAJIT-PR 0069 (EA, HIB). Native distribution range: S Central Ethiopia to S Africa.

***Ochnainsculpta* Sleumer** – Habit: Shrub or tree. Habitat: Grassland, woodland, lower forest edge, 1750–2400 m. Voucher: Tweedie 21 (EA). Native distribution range: S Ethiopia to N Tanzania.

***Ochnamembranacea* Oliv.** – Habit: Shrub or small tree. Habitat: Forest, 1050–2250 m. Voucher: Jackson 417 (EA). Native distribution range: Tropical Africa.

#### F89. OLACACEAE

1 Genus, 1 species

***Strombosiascheffleri* Engl.** – Habit: Tree. Habitat: Rain forest, 800–2500 m. Voucher: St Clair-Thomson in Eggeling 3940 (K). Native distribution range: Nigeria to South Sudan and S Tropical Africa.

#### F90. OLEACEAE

4 Genera, 10 species

^**EX**^***Fraxinusquadrangulata* Michx.** – Habit: Tree. Habitat: Roadside, forest, 2590 m. Voucher: Rono et al. SAJIT-PR 0257 (EA, HIB). Native distribution range: N Central & E Central America.

***Jasminumabyssinicum* R.Br.** – Habit: Climbing shrub. Habitat: Shrubland, open and riverine forest, 900–3000 m. Voucher: Rono et al. SAJIT-PR 0225 (EA, HIB). Native distribution range: Cameroon to Eritrea and S Africa.

***Jasminumfluminense* Vell.** – Habit: Climbing shrub. Habitat: Woodland on shallow soil, 280–2650 m. Voucher: Agnew ADQ (2013) (REF). Native distribution range: Tropical & S Africa to Arabian Peninsula.

**Jasminumgrandiflorumsubsp.floribundum (R.Br. ex Fresen.) P.S.Green** – Habit: Shrub. Habitat: Bush on open ground, 400–2480 m. Vouchers: Snowden JD 831 (EA), Rono et al. SAJIT-PR 0085 (EA, HIB). Native distribution range: Eritrea to Rwanda, Arabian Peninsula.

***Oleacapensis* L.** – Habit: Tree. Habitat: Wet land forest, 1150–2550m. Voucher: Rono et al. SAJIT-PR 0007 (EA, HIB). Native distribution range: Tropical & S Africa, Comoros, Madagascar.

**Oleacapensissubsp.macrocarpa (C.H.Wright) I.Verd.** – Habit: Tree. Habitat: Forest, 2250–3000 m. Voucher: Tweedie EM (1976) (REF). Native distribution range: Tropical & S Africa, Comoros, Madagascar.

***Oleaeuropaea* L.** – Habit: Shrub or tree. Habitat: Forest, 2250–3000 m. Vouchers: Ashburner M LCA 8 (EA), Taiti S 2 (EA), Fairbairn G 801 (EA). Native distribution range: Africa, Mediterranean to S Central China.

**Oleaeuropaeasubsp.cuspidata (Wall. & G.Don) Cif.** – Habit: Tree. Habitat: Forest, 950–2400 m. Vouchers: Fairbairn G 801 (US), Rono et al. SAJIT-PR 0017 (EA, HIB). Native distribution range: Eritrea to S Africa, Mascarenes, Arabian Peninsula to China (Yunnan).

***Oleawelwitschii* Gilg & G.Schellenb.** – Habit: Tree. Habitat: Forest, 2250–3000 m. Voucher: Tweedie EM (1976) (REF). Native distribution range: Ethiopia to S Tropical Africa.

***Schreberaalata* Welw.** – Habit: shrub or tree. Habitat: Bushland and forest, 1950–2400 m. Vouchers: (Fr), Jackson THE 344a (EA), Snowden JD 1016 (EA). Native distribution range: Eritrea to S Africa.

#### F91. ONAGRACEAE

2 Genera, 4 species

***Epilobiumhirsutum* L.** – Habit: Herb. Habitat: Swamps, woodland, 11900–2690 m. Vouchers: Major and Mrs Lugard 499 (EA), Symes YE 332 (EA), Rono et al. SAJIT-PR 0263 (EA, HIB). Native distribution range: Temperate Eurasia to Africa.

***Epilobiumsalignum* Hausskn.** – Habit: Woody herb. Habitat: Swampy places near streams, woodland, 1800–2830 m. Vouchers: Mrs Powles 35 (EA), Symes YE 520 (EA). Native distribution range: Tropical & S Africa, Madagascar.

***Epilobiumstereophyllum* Fresen.** – Habit: Stoloniferous herb. Habitat: Swamps, moor grassland, along water course, upper forest edge, 2600–3660 m. Vouchers: Hedberg O 1000 (EA), 4449 (EA), Adamson J 491 (EA), Svein Manum 64 (O), Tweedie DR 21 (EA), Taylor G 3695 (EA), Bush RZ 250 (EA), Lisowski S 56570 (BR), Rono et al. SAJIT-PR 0260 (EA, HIB). Native distribution range: Ethiopia to Malawi.

***Ludwigiaabyssinica* A.Rich.** – Habit: Herb. Habitat: Swamps, along rivers, woodland, 1000–2300 m. Voucher: Lugard C & Lugard EJ 335 (EA). Native distribution range: Tropical & S Africa, Comoros, Madagascar.

#### F92. OROBANCHACEAE

9 Genera, 26 species

***Alectraorobanchoides* Benth.** – Habit: Herb. Habitat: Woodland, grassland, montane scrub, rock outcrop, 1750–2000 m. Voucher: Tweedie 2230 (EA). Native distribution range: Uganda to S Africa.

***Alectrasessiliflora* (Vahl) Kuntze** – Habit: Herb. Habitat: Montane grassland, forest, 1850–3000 m. Vouchers: Major Lugard sn (EA), Mwangangi OM 411 (EA), Rono et al. SAJIT-PR 0157 (EA, HIB). Native distribution range: Tropical & S Africa to Philippines.

***Alectravogelii* Benth.** – Habit: Herb. Habitat: Forest, grassland, 1750–2250 m. Voucher: Tweedie EM (1976) (REF). Native distribution range: Tropical & S Africa.

***Bellardiatrixago* (L.) All.** – Habit: Herb. Habitat: Alpine zone, rocky places, grassland, 930–3100 m. Voucher: Agnew ADQ (2013) (REF). Native distribution range: Macaronesia, Mediterranean to Iran and W Central Kenya, South Africa.

***Buchneracapitata* Burm.f.** – Habit: Herb. Habitat: Grassland, woodland, forest, 1750–2540 m. Vouchers: Wood 483 (EA), Irwin PH 245 (EA). Native distribution range: Tropical Africa, Madagascar.

***Buchnerahispida* Buch.-Ham. ex D.Don** – Habit: Herb. Habitat: Outcrop rock, 1800–2400 m. Voucher: Tweedie EM (1976) (REF). Native distribution range: Africa, Arabian Peninsula, Indian Subcontinent.

***Buchneranuttii* Skan** – Habit: Herb. Habitat: Wooded grassland, 1670–2170 m. Voucher: Jack C 97 (EA). Native distribution range: South Sudan to S Tropical Africa.

***Buchneraruwenzoriensis* Skan** – Habit: Herb. Habitat: Grassland, 1200–1900 m. Vouchers: Wood 478 (EA). Native distribution range: Uganda, Angola, Zambia.

***Cycniumadonense* E.Mey. ex Benth.** – Habit: Herb. Habitat: Forest, 1750–2500 m. Vouchers: Lugard C 568 (EA), Jack C 234 (EA). Native distribution range: Tropical & S Africa.

***Cycniumerectum* Rendle** – Habit: Woody herb. Habitat: Wooded grassland, forest, grassland, 1500–2500 m. Vouchers: Tweedie 1724 (EA), Snowden JD 935 (EA), Dale 3094 (EA), Irwin PH 449 (EA). Native distribution range: Ethiopia to Uganda.

***Cycniumherzfeldianum* (Vatke) Engl.** – Habit: Herb. Habitat: Grassland, woodland, 1750–2500 m. Vouchers: Irwin PH 72 (EA), Rono et al. SAJIT-PR 0058 (EA, HIB). Native distribution range: Ethiopia to DR Congo.

***Cycniumjamesii* (Skan) O.J.Hansen** – Habit: Herb. Habitat: Marshy grasslands, wooded grassland, 1500–2550 m. Vouchers: Snowden JD 867 (EA), James E sn (K). Native distribution range: SW Ethiopia to Burundi.

***Cycniumrecurvum* Engl.** – Habit: Herb. Habitat: Disturbed grassland, outcrop rock, forest, 1500–3150 m. Voucher: Tweedie 1477 (EA). Native distribution range: South Sudan to N Malawi.

***Cycniumtenuisectum* (Standl.) O.J.Hansen** – Habit: Herb or week shrub. Habitat: Upland grassy marshes, 1800–3500 m. Vouchers: Tweedie 17 (EA), Dummer RA 3399 (EA), Williams JW 74/5 (EA), Rono et al. SAJIT-PR 0127 (EA, HIB). Native distribution range: S Ethiopia to E Central & E Tropical Africa.

***Cycniumtubulosum* Engl.** – Habit: Herb. Habitat: Upland bushland, outcrop rock, 1800–3000 m. Vouchers: Symes YE 243 (EA), Lugard EJ 139 (EA), Lind EM 2153 (EA). Native distribution range: Tropical & S Africa, Madagascar.

***Hedbergiaabyssinica* (Benth.) Molau** – Habit: Herb. Habitat: Woodland, upper forest edge, 1750–4300 m. Vouchers: Snowden JD 450 (S), Hedberg et al. 2101 (EA). Native distribution range: S Nigeria to Cameroon, Ethiopia to Malawi.

***Hedbergiadecurva* (Hochst. ex Benth.) A.Fleischm. & Heubl** – Habit: Herb or undershrub. Habitat: Alpine belt, moorland, upper forest edge, 3150–4321 m. Vouchers: Wesche K 09 (EA), Osmaston 4011 (EA), Leibenberg 1649 (EA), Bush RZ 254 (EA), Hedberg O 868 (EA), Tweedie DR 60 (EA), Thomas AS 538 (EA), Ekkens DB 409 (EA), Mwangangi OM 316 (EA), Gardiner N 21877 (EA), Gardiner N 1345 (EA), Bitzford N 23 (EA). Native distribution range: Ethiopia to E Uganda and N Tanzania.

***Hedbergialongiflora* (Hochst. ex Benth.) A.Fleischm. & Heubl** – Habit: Herb or Undershrub. Habitat: Forest, heath zone, 2950–3840 m. Vouchers: Wilson G 1240 (EA), Hedberg O 4459 (EA), Lye and Po in Lye 23072 (EA), Tweedie 2425 (K). Native distribution range: Ethiopia to E Uganda and N Tanzania.

**Hedbergialongiflorasubsp.macrophylla (Hedberg) A.Fleischm. & Heubl** – Habit: herb or subshrub. Habitat: Upper forest edge, heath, afro-alpine zone, 2400–3800 m. Vouchers: Lye & Poe in Lye 23072 (EA), Hedberg 4459 (EA). Native distribution range: E Central Tropical Africa (Ruwenzori Mountains, Virunga Volcanoes).

***Micrargeriafiliformis* (Schumach. & Thonn.) Hutch. & Dalziel** – Habit: Herb. Habitat: Wet grassland, seepages in woodland, outcrop rock, 1800–2400 m. Vouchers: Lind EM 472 (EA). Native distribution range: Tropical Africa, Madagascar.

***Orobancheminor* Sm.** – Habit: Herb. Habitat: Grassland, forest, 800–2480 m. Voucher: Rono et al. SAJIT-PR 0163 (EA, HIB). Native distribution range: Europe to N Iraq, Macaronesia to Arabian Peninsula.

***Sopubiaeminii* Engl.** – Habit: Herb. Habitat: Grassland, 1000–2790 m. Voucher: Tweedie 691 (EA). Native distribution range: Ethiopia to S Tropical Africa.

***Sopubiaramosa* Hochst.** – Habit: Herb or undershrub. Habitat: Wooded grassland, 1600–2170 m. Vouchers: Tweedie EM (1976) (REF), Agnew ADQ (2013) (REF). Native distribution range: Tropical Africa.

**^EX^*Sopubiatrifida* Buch.-Ham. ex D.Don** – Habit: Herb. Habitat: Outcrop rock, 1800–2400 m. Voucher: Tweedie EM (1976) (REF). Native distribution range: S China to Tropical Asia.

***Strigaasiatica* (L.) Kuntze** – Habit: Herb. Habitat: Grassland, 0–2480 m. Voucher: Agnew ADQ (2013) (REF). Native distribution range: Tropical & Subtropical Old World.

***Strigaforbesii* Benth.** – Habit: Herb. Habitat: Grassland, 1060–2350 m. Voucher: Agnew ADQ (2013) (REF). Native distribution range: Tropical & S Africa, Madagascar.

#### F93. OXALIDACEAE

2 Genera, 5 species

***Biophytumabyssinicum* Steud. ex A.Rich.** – Habit: Herb. Habitat: Rocky grounds, grassland, forest, 1200–2650 m. Voucher: Jack C 49 (EA). Native distribution range: Nigeria to Eritrea.

***Oxalisanthelmintica* A.Rich.** – Habit: Herb. Habitat: Grassland, riverine forest edges, 830–2400 m. Voucher: Hedberg 810 (EA). Native distribution range: Sudan to S Tropical Africa.

^**EX**^***Oxaliscorniculata* L.** – Habit: Herb. Habitat: Upper forest edge and moorland, 0–4100 m. Vouchers: Dummer RA 3517 (EA), Tweedie 2674, 2675, Jack C 135 (EA), Synge PM s 942 (WAG). Native distribution range: Mexico to Venezuela and Peru, Caribbean.

^**EX**^***Oxalislatifolia* Kunth** – Habit: Herb. Habitat: Woodland, forest, cultivation, 1300–2500 m. Vouchers: Tweedie EM (1976) (REF), Agnew ADQ (2013) (REF). Native distribution range: Tropical & Subtropical America.

***Oxalisobliquifolia* Steud. ex A.Rich.** – Habit: Herb. Habitat: Moist rock outcrops, woodland, lower forest edge, 1750–3400 m. Voucher: Wesche K 1558 (EA). Native distribution range: Eritrea to S Africa.

#### F94. PAPAVERACEAE

1 Genus, 1 species

***Fumariaabyssinica* Hammar** – Habit: Herb. Habitat: Upland Forest, bamboo, upland moor, 2100–3400 m. Vouchers: Wesche K 1781 (K, EA), Hedberg O 4473 (EA), Tothill BH 2277 (EA). Native distribution range: Eritrea to Malawi, SW Arabian Peninsula.

#### F95. PASSIFLORACEAE

1 Genus, 1 species

***Adeniabequaertii* Robyns & Lawalrée** – Habit: Woody climber. Habitat: Upland forest, riverine forest, 1350–2300 m. Voucher: Tweedie EM (1976) (REF). Native distribution range: E DR Congo to SW Kenya.

#### F96. PEDALIACEAE

1 Genus, 1 species

^**EX**^***Sesamumindicum* L.** – Habit: Herb or subshrub. Habitat: Forest, grassland, roadside 1750–2250 m. Voucher: Tweedie EM (1976) (REF). Native distribution range: Indian Subcontinent.

#### F97. PENAEACEAE

1 Genus, 1 species

***Oliniarochetiana* A.Juss.** – Habit: Shrub or tree. Habitat: Forest, fire degraded forest, 1680–3000 m. Vouchers: Ekkens DB 418 (EA), Eggeling 2484 (EA), Cheseny CMC 31 (EA), Napier ER 2535 (EA), Major & Lugard EJ 202 (EA), Jackson THE 1088 (EA), Tweedie DR 1287 (BR), Jackson 337 (EA), Rono et al. SAJIT-PR 0213 (EA, HIB). Native distribution range: Ethiopia to N Tanzania.

#### F98. PHYLLANTHACEAE

2 Genera, 3 species

***Brideliamicrantha* (Hochst.) Baill.** – Habit: Shrub or tree. Habitat: Woodland, forest, along rivers, 1750–2400 m. Voucher: Jackson THE 346 (EA). Native distribution range: Tropical & S Africa, Mascarenes.

***Phyllanthusfischeri* Pax** – Habit: Shrub or herb. Habitat: Forest edges, 1650–2960 m. Vouchers: Tweedie EM (1976) (REF), Agnew ADQ (2013) (REF). Native distribution range: Eritrea to E Central & E Tropical Africa.

***Phyllanthusnummulariifolius* Poir.** – Habit: Herb or shrub. Habitat: Woodland, wooded grassland, forest edge, 630–2650 m. Voucher: Agnew ADQ (2013) (REF). Native distribution range: Africa.

#### F99. PHYTOLACCACEAE

1 Genus, 1 species

***Phytolaccadodecandra* L’Hér.** – Habit: Climbing or scrambling shrub. Habitat: Riverine forest, 1650–2450 m. Vouchers: Tweedie EM (1976) (REF), Agnew ADQ (2013) (REF). Native distribution range: Tropical & S Africa, Madagascar.

#### F100. PIPERACEAE

2 Genera, 5 species

***Peperomiaabyssinica* Miq.** – Habit: Epiphytic herb in leaf-litters. Habitat: Montane forest, 1600–2950 m. Vouchers: Bamps PRJ 6490 (BR), Rono et al. SAJIT-PR 0196 (EA, HIB). Native distribution range: Cameroon to Eritrea and Malawi.

***Peperomiaretusa* A.Dietr.** – Habit: Epiphytic herb. Habitat: Mist forest, streams, rocky places, mossy trunks, 1400–2400 m. Voucher: Tweedie 3335 (EA), RH Goodwin 120 (GH). Native distribution range: Tropical & S Africa, Madagascar.

***Peperomiatetraphylla* Hook. & Arn.** – Habit: Epiphytic herb. Habitat: Forest, 1400–2450 m. Vouchers: Hedberg O (UPS), Rono et al. SAJIT-PR 0195 (EA, HIB). Native distribution range: Tropics & Subtropics.

***Pipercapense* L.f.** – Habit: Shrub, subshrub, liana or herb. Habitat: Forest undergrowth, mixed bamboo-forest, 1200–2700 m. Vouchers: Tweedie EM (1976) (REF), Agnew ADQ (2013) (REF). Native distribution range: Tropical & S Africa, Comoros, Madagascar.

^**EX**^***Piperumbellatum* L.** – Habit: Shrub or woody herb. Habitat: Moist forest, 1190–2100 m. Vouchers: Katende 651 (K), Tiyoy LM 1505 (K). Native distribution range: Mexico to Tropical America.

#### F101. PITTOSPORACEAE

1 Genus, 2 species

***Pittosporumabyssinicum* Delile** – Habit: Tree. Habitat: Forest, 2250–2400 m. Voucher: Tweedie EM (1976) (REF). Native distribution range: Ethiopia to E & E Central Tropical Africa.

***Pittosporumviridiflorum* Sims** – Habit: Tree or shrub. Habitat: Forest, bushland, riverine forest, 900–2550 m. Vouchers: Gardner HM 610 (EA), Katende AB 3603 (UPS). Native distribution range: Tropical & S Africa, Madagascar, Arabian Peninsula.

#### F102. PLANTAGINACEAE

5 Genera, 11 species

*Callitrichekeniensis Schotsman – Habit: Aquatic herb. Habitat: Small pools, streams, 2100–4250 m. Vouchers: Wesche K 116 (EA), O Hedberg 939 (EA). Native distribution range: Uganda (Mt Elgon) to Kenya (Mountains).

^**EX**^***Callitrichestagnalis* Scop.** – Habit: Aquatic herb. Habitat: Streams, ponds, crater moorland, 2150–4250 m. Vouchers: Hedberg O 939 (EA). Native distribution range: Macaronesia, NW Africa, Europe to Turkey.

***Misopatesorontium* (L.) Raf.** – Habit: Herb. Habitat: Grassland, cultivation, roadside, 1650–2730 m. Vouchers: Tweedie EM (1976) (REF), Agnew ADQ (2013) (REF). Native distribution range: Macaronesia, Mediterranean to NE Sudan.

**^EX^*Plantagolanceolata* L.** – Habit: Herb. Habitat: Roadside, wooded grassland, 1650–2750 m. Voucher: Rono et al. SAJIT-PR 0072 (EA, HIB). Native distribution range: Macaronesia, N Africa to Mauritania, Temperate Eurasia.

***Plantagopalmata* Hook.f.** – Habit: Herb. Habitat: Montane forest, roadside, 1705–4030 m. Vouchers: Jack C 177 (EA), 228 (EA), Beentje HJ 1970 (EA), Mwangangi OM 323 (EA), Rono et al. SAJIT-PR 0165 (EA, HIB). Native distribution range: Bioko to Ethiopia and Zimbabwe.

***Sibthorpiaeuropaea* L.** – Habit: Herb. Habitat: Montane grassland, roadsides, 2150–3750 m. Vouchers: Hedberg sn, Dummer RA 3521 (EA), Hedberg 270 (EA), Kisalye N; van Heist 134 (K). Native distribution range: Azores, W & S Europe to Tropical African Mountains.

***Veronicaabyssinica* Fresen.** – Habit: Trailing herb. Habitat: Grassland, woodland edges, 1560–3900 m. Vouchers: Tothill BH 2335 (EA), Beentje HJ 1969 (EA, WAG), Hedberg O 989 (EA), Webster MVB 8943 (EA), Bush RZ 285 (EA), Taylor G 3723 (BR, EA). Native distribution range: Nigeria to Somalia and S Tropical Africa.

***Veronicaanagallis-aquatica* L.** – Habit: Herb. Habitat: Woodland, permanent shallow running waters, 1750–2400 m. Vouchers: Tweedie 2237 (EA), Hedberg O (UPS). Native distribution range: Temperate Eurasia to Tropical Mountains.

**^EX^*Veronicacalycina* R.Br.** – Habit: Trailing herb. Habitat: Rocky upland soils, 2700–4300 m. Vouchers: Wesche K 88 (EA), Hedberg 9411 (EA). Native distribution range: SW Western Australia, SE Queensland to SE Australia.

***Veronicaglandulosa* Hochst. ex Benth.** – Habit: Trailing herb. Habitat: Upper montane forest, alpine, moorland, 2000–4100 m. Vouchers: Irwin PH 363 (EA), Hedberg O 857, 4549 (EA), Lye KA 5724 (EA), Webster MVB 8942 (EA), Tweedie DR 16, 69, 129, 4030 (EA), Dale IR 3187 (EA), Mwangangi OM 309 (EA), Bickford N 51 (EA). Native distribution range: Ethiopia to E Central & E Tropical Africa.

***Veronicajavanica* Blume** – Habit: Herb. Habitat: Grassland, 1800–2790 m. Voucher: Tweedie 1448 (EA). Native distribution range: Eritrea to S Tropical Africa, Tropical & Subtropical Asia.

#### F103. PLUMBAGINACEAE

1 Genus, 1 species

***Plumbagomontis-elgonis* Bullock** – Habit: Herb. Habitat: Forest, riverine, 1350–2170 m. Vouchers: Lugard C 657 (K), 637 (EA). Native distribution range: SW Ethiopia to NW Tanzania.

#### F104. PODOSTEMACEAE

2 Genera, 2 species

*Ledermanniellamaturiniana Beentje – Habit: Herb. Habitat: Waterfalls, fast flowing water, 800–1500 m. Voucher: Taylor G 3140 (EA, MO). Native distribution range: Kenya.

***Tristichatrifaria* Spreng.** – Habit: Aquatic herb. Habitat: Streams and rivers, woodland, 1300–2300 m. Voucher: Thomas AS 2013 (EA). Native distribution range: Tropics & Subtropics.

#### F105. POLYGALACEAE

1 Genus, 10 species

***Polygalaalbida* Schinz** – Habit: Herb. Habitat: Roadsides, wooded grassland, cultivated fields, 1400–2300 m. Voucher: Symes YE 233 (EA). Native distribution range: E Ethiopia, Rwanda to S Africa.

**Polygalaalbidasubsp.stanleyana (Chodat) Paiva** – Habit: Herb. Habitat: Roadsides, woodland, cultivation, grassland, 1750–2400 m. Voucher: Tweedie EM (1976) (REF). Native distribution range: Tropical Africa to Namibia.

***Polygalaerioptera* DC.** – Habit: Herb or shrublet. Habitat: Grassland, bushland, woodland, rock outcrop, 120–2000 m. Voucher: Agnew ADQ (2013) (REF). Native distribution range: Arabian Peninsula, India subcontinent to Indo-China, Africa.

***Polygalaohlendorfiana* Eckl. & Zeyh.** – Habit: Prostrate herb. Habitat: Burnt grassland, 1800–2750 m. Voucher: Jex-Blake B 1259 (EA). Native distribution range: Kenya to S Africa.

***Polygalapersicariifolia* DC.** – Habit: Herb. Habitat: Grassland, wooded grassland, 1750–2250 m. Voucher: Tweedie 4096 (EA). Native distribution range: Tropical Africa, Tropical & Subtropical Asia to N Australia.

***Polygalapetitiana* A.Rich.** – Habit: Herb. Habitat: Grassland, 1750–2400 m. Vouchers: Webster 8716 (EA), Jack C 34 (EA). Native distribution range: Tropical Africa.

***Polygalasadebeckiana* Gürke** – Habit: Herb or shrublet. Habitat: Forest, riverine forest, grassland, 1500–2400 m. Vouchers: Tweedie EM (1976) (REF), Agnew ADQ (2013) (REF). Native distribution range: Sudan to Mozambique.

**Polygalasparsifloravar.ukirensis (Gürke) Paiva** – Habit: Herb. Habitat: Grassland, 1000–2250 m. Voucher: Major EJ & Lugard C 20 (EA). Native distribution range: Sierra Leone, Cameroon, Uganda to S Tropical Africa.

***Polygalasphenoptera* Fresen.** – Habit: Shrubby herb. Habitat: Forest, grassland 1750–3300 m. Vouchers: Bamps PRJ 6512 (EA, WAG), Lugard EJ 477 (EA), Jack C 71 (EA), Webster MVB 8714 (EA), Ross R 1360 (BR), Rono et al. SAJIT-PR 0098 (EA, HIB). Native distribution range: Eritrea to S Africa.

***Polygalasteudneri* Chodat** – Habit: Herb. Habitat: Afro alpine grassland, rocky grassland, 3000–4050 m. Vouchers: Dummer RA 3365 (EA), Dale 3101, Wesche K 1827, Tweedie DR 54 (EA). Native distribution range: Ethiopia to N Tanzania.

#### F106. POLYGONACEAE

5 Genera, 12 species

^**EX**^***Fallopiaconvolvulus* (L.) Á.Löve** – Habit: Climbing, twining herb. Habitat: Forest, cultivation, grassland, 1750–2250 m. Voucher: Irwin PH 228 (EA). Native distribution range: Macaronesia to N Africa, Temperate Eurasia.

***Harpagocarpussnowdenii* Hutch. & Dandy** – Habit: Climbing herb. Habitat: Forest, 1350–2400 m. Voucher: Katende T; Sheil D 2257 (K). Native distribution range: Cameroon to South Sudan and E Tanzania

***Oxygonumsinuatum* (Hochst. & Steud. ex Meisn.) Dammer** – Habit: Herb or shrub. Habitat: Cultivation, roadsides, 600–2250 m. Voucher: Symes YE 121 (EA). Native distribution range: Tropical & S Africa.

***Persicariadecipiens* (R.Br.) K.L.Wilson** – Habit: Herb. Habitat: Marshes, waterside, 1000–3170 m. Vouchers: Beentje HJ 1966 (EA, WAG), Lugard C & Lugard EJ 589 (EA). Native distribution range: Tropical & Subtropical Old World to Australasia.

***Persicarianepalensis* (Meisn.) H.Gross** – Habit: Herb. Habitat: Forest edges, marshes, in cultivation, streams and rivers, 1140–2700 m. Vouchers: Katende T; Sheil, D 667 (K), van Heist, M. 570 (K), Hedberg O 105 (EA). Native distribution range: Eritrea to South Africa, Madagascar, Tropical & Subtropical Asia.

**^EX^*Persicariapulchra* Soják** – Habit: Herb. Habitat: Waterside plant, upto 2500 m. Voucher: Agnew ADQ (2013) (REF). Native distribution range: India to SE China and W & Central Malesia.

***Persicariasenegalensis* (Meisn.) Soják** – Habit: Herb. Habitat: Waterside, marshes, up to 2850 m. Voucher: Agnew ADQ (2013) (REF). Native distribution range: Africa to Arabia Peninsula.

***Persicariasetosula* (A.Rich.) K.L.Wilson** – Habit: Herb. Habitat: Waterside, 1330–3000 m. Vouchers: Katende 1193 (K), Tiyoy L 1392 (K), Hedberg KO 32 (BR), Beentje HJ 1967 (EA), Jack C 500, 131 (EA), Hedberg O 109 (EA), Rono et al. SAJIT-PR 0181 (EA, HIB). Native distribution range: Tropical Africa to SW Syria.

***Rumexabyssinicus* Jacq.** – Habit: Herb. Habitat: Upland grassland, forest, 1600–3300 m. Voucher: Tiyoy LM 1389 (K). Native distribution range: Nigeria to Eritrea and S Tropical Africa, Madagascar.

***Rumexnepalensis* Spreng.** – Habit: Herb. Habitat: Montane grassland, woodland, forest, 1750–3000 m. Vouchers: Sheil and Vanheist 362 (K), Naiga; Lawrence 301 (K). Native distribution range: Cameroon to Eritrea and S Africa, Madagascar, SE Europe to China, Jawa.

***Rumexruwenzoriensis* Chiov.** – Habit: Herb. Habitat: Upland grassland, upland moor, bamboo forest, 1950–3700 m. Voucher: Human observation sn (EA). Native distribution range: E Central & E Tropical Africa.

***Rumexsteudelii* Hochst. ex A.Rich.** – Habit: Herb. Habitat: Upland forest in streamside, roadside, 1200–3240 m. Voucher: Agnew ADQ (2013) (REF). Native distribution range: Cameroon to Eritrea and S Africa, Madagascar, SE Europe to China.

#### F107. PORTULACACEAE

1 Genus, 3 species

***Portulacaafricana* (Danin & H.G.Baker) Danin** – Habit: Herb. Habitat: Grassland, in cultivation, ruderal sites, 500–2350 m. Voucher: Tweedie 1996 (EA). Native distribution range: Mauritania to Mali, Egypt to Malawi, India, S China.

***Portulacaoleracea* L.** – Habit: Herb. Habitat: Wet savanna, woodland, lower forest edge, 500–2400 m. Vouchers: Tweedie EM (1976) (REF), Agnew ADQ (2013) (REF). Native distribution range: Macaronesia, Tropical Africa, Mediterranean to Pakistan and Arabian Peninsula.

***Portulacaquadrifida* L.** – Habit: Herb. Habitat: Stony grassland, shrubland, 300–2400 m. Vouchers: Tweedie EM (1976) (REF), Agnew ADQ (2013) (REF). Native distribution range: Tropical & Subtropical Old World to SW Pacific.

#### F108. PRIMULACEAE

4 Genera, 9 species

***Ardisiandrawettsteinii* J.Wagner** – Habit: Herb. Habitat: Subalpine bushlands, moist bamboo thickets, 1580–3500 m. Vouchers: Tweedie EM (1976) (REF), Agnew ADQ (2013) (REF). Native distribution range: Ethiopia to S Tropical Africa.

***Lysimachiaadoensis* (Kunze) Klatt** – Habit: Herb. Habitat: Grassland, cultivation, 2015–3500 m. Voucher: Tweedie 855 (EA). Native distribution range: Eritrea to N Tanzania.

**^EX^*Lysimachiaarvensis* (L.) U.Manns & Anderb.** – Habit: Herb. Habitat: Grassland, bushland, 1400–2635 m. Voucher: Tweedie 849 (EA). Native distribution range: Europe to Central Asia and Himalaya, N Africa to Ethiopia and Arabian Peninsula.

***Lysimachiaruhmeriana* Vatke** – Habit: Herb. Habitat: Grasslands, woodland, riverine, marshes, forest, 2020–3500 m. Vouchers: Taylor G 3650, 3559 (EA), Tweedie 1408 (EA), Dummer RA 3561 (US). Native distribution range: Cameroon to Eritrea and S Africa, Madagascar.

***Lysimachiaserpens* (Hochst. ex A.DC.) U.Manns & Anderb.** – Habit: Herb. Habitat: Forest, moorland, 3000–3150 m. Vouchers: Forbes 275 (EA), Thomas AS 2688 A (EA). Native distribution range: Ethiopia to E Zimbabwe.

^**EX**^***Lysimachiatenella* L.** – Habit: Herb. Habitat: Grassland, 1650–2040 m. Voucher: Gilbert MG & Tadesse M 6544 (EA). Native distribution range: W & S Europe, NW Africa.

***Maesalanceolata* Forssk.** – Habit: Tree. Habitat: Grassland, forest, 1750–3000 m. Vouchers: Snowden JD 832 (EA), Taylor 3419 (MO). Native distribution range: Tropical & S Africa, Madagascar, Arabian Peninsula.

***Myrsineafricana* L.** – Habit: Shrub, undershrub or tree. Habitat: Upland forest edge, open wooded grassland, 2250–3600 m. Vouchers: Wesche K 1008 (EA), Webster MVB 8821 (EA), Tweedie DR 1994 (B), Rono et al. SAJIT-PR 0036 (EA, HIB). Native distribution range: Azores, Eritrea to S Africa, Arabian Peninsula to China, Taiwan.

***Myrsinemelanophloeos* R.Br. ex Sweet** – Habit: Tree or shrub. Habitat: Riverine, swamp forest, upland grassland, open woodland, 900–3750 m. Voucher: Eggeling 2453 (EA). Native distribution range: Nigeria to Ethiopia and S Africa, Comoros, Madagascar.

#### F109. PROTEACEAE

2 Genera, 6 species

***Faurearochetiana* Chiov. ex Pic.Serm.** – Habit: Shrub or small tree. Habitat: Wet savanna on rocky slops, 1750–2400 m. Voucher: Taylor G 3727 (EA). Native distribution range: Tropical & S Africa.

***Faureasaligna* Harv.** – Habit: Tree. Habitat: Forest, woodland, outcrop rock, 3000–3150 m. Voucher: JR Dale (MA). Native distribution range: South Sudan to S Africa.

***Proteacaffra* Meisn.** – Habit: Shrub or tree. Habitat: Rock outcrop, grassland, woodland, 1000–2300 m. Vouchers: Taylor G (S), Bush RZ 278 (EA). Native distribution range: South Sudan to S Africa.

**Proteacaffrasubsp.kilimandscharica (Engl.) Chisumpa & Brummitt** – Habit: Shrub or tree. Habitat: Forest, 2300–3700 m. Vouchers: Eggeling WJ 5739 (EA), Snowden JD 469 (EA). Native distribution range: E Central & E Tropical African Mountains.

***Proteagaguedi* J.F.Gmel.** – Habit: Large bush or small tree. Habitat: Bushland, tree grassland, 1500–2900 m. Vouchers: Gerti Lindblom (S), Snowden JD 1068 (EA), Rono et al. SAJIT-PR 0130 (EA, HIB). Native distribution range: Eritrea to S Africa.

***Proteamadiensis* Oliv.** – Habit: Shrub or tree. Habitat: Grassland, woodlands, 1750–2250 m. Voucher: Tweedie EM (1976) (REF). Native distribution range: S Nigeria to Ethiopia and S Tropical Africa.

#### F110. RANUNCULACEAE

5 Genera, 14 species

***Anemonethomsonii* Oliv.** – Habit: Herb. Habitat: Moist/boggy grassland, outcrop rocks, 2500–4000 m. Vouchers: Dale 3090 (EA), Tweedie 56 (EA), Lugard C 305 (EA), Liebenberg 1605 (EA), Manum 45 (O), Rono et al. SAJIT-PR 0141 (EA, HIB). Native distribution range: S Central Ethiopia to E DR Congo.

***Clematisbrachiata* Thunb.** – Habit: Woody climber. Habitat: Forest, wooded grassland, 720–3150 m. Vouchers: E Hind 429 (BR), Jack C 18 (EA), Lugard C & Lugard EJ 65 (EA), Smeri PH 39 (EA), Symes YE 240 (EA). Native distribution range: Senegal, Cameroon to Ethiopia and S Africa, Madagascar.

***Clematishirsuta* Guill. & Perr.** – Habit: Woody climber. Habitat: Forest, woodland grassland, 1750–2400 m. Voucher: PH Sruin 39 (BR). Native distribution range: Cape Verde, Sahara to Tropical Africa, SW Arabian Peninsula, S India.

***Clematissimensis* Fresen.** – Habit: Shrubby climber. Habitat: Forest, roadsides, grassland woodland, 1600–3250 m. Vouchers: Beentje HJ 1985 (EA, WAG), Irwin PH 375 (EA), Irving FR 38 (EA), Tweedie DR 87 (EA). Native distribution range: Tropical Africa, SW Arabian Peninsula, Madagascar.

***Clematisvillosa* DC.** – Habit: Herb. Habitat: Burnt grassland, rock outcrop, 1500–2100 m. Voucher: Snowden JD 840 (BR). Native distribution range: S Uganda to Angola.

*Delphiniummacrocentrum Oliv. – Habit: Herb. Habitat: Rocky grassland, moist bamboo thickets, 1650–3900 m. Vouchers: Tothill BH 2411 (EA), Allen T 3675 (EA), Hedberg O 4512 (EA), Bickford N 25 (EA), 41 (EA), Tweedie 13 (EA), Jack C 2643 (EA), Lugard C & Lugard EJ 26 (EA), Ritchie AT & Beentje 1998 (EA), Bogdan A 5407 (EA), Webster MVB 8711 (EA). Native distribution range: E Tropical Africa (Kenya Highlands, Mt Elgon), N Malawi.

*Delphiniumwellbyi Hemsl. – Habit: Herb. Habitat: Grassland, 1200–1500 m. Vouchers: Tweedie 722 (EA), Greenway PJ & Daughty LR 8532 (EA). Native distribution range: Ethiopia, Central Kenya.

*Ranunculusaberdaricus Ulbr. – Habit: Herb. Habitat: Rare in wet alpine turf, bamboo thickets, 2550–3660 m. Voucher: Agnew ADQ (2013) (REF). Native distribution range: Ethiopia, Kenya (Aberdare Mountains)

**Ranunculuscryptanthus Milne-Redh. & Turrill – Habit: Herb. Habitat: Upland moor, 4050–4100 m. Vouchers: Taylor 3542 (EA), Hedberg K.O 1005 (EA, UPS, K, S). Native distribution range: E Tropical Africa (Mt Elgon).

***Ranunculusmultifidus* Forssk.** – Habit: Herb. Habitat: Forest, stream sides, 1530–3450 m. Vouchers: Beentje HJ 1968 (WAG), Mwangangi OM 380 (BR), Rono et al. SAJIT-PR 0235 (EA, HIB), Naiga 450 (UPS), Symes YE 251 (EA), Hamilton & Perrott 76/408 (EA). Native distribution range: Nigeria to Eritrea and S Africa, SW Arabian Peninsula, Madagascar.

***Ranunculusoreophytus* Delile** – Habit: Herb. Habitat: Forest, rocky clef, grassland, by streamside, upland moor, 2550–4200 m. Vouchers: Svein Manum 16 (LD), Mathama M & Gehrke B 137 (EA), Ekkens DB 413 (EA), Tweedie 12 (EA), Knox EB 3839, 3845 (EA). Native distribution range: Sudan to Tanzania.

***Ranunculusstagnalis* Hochst. ex A.Rich.** – Habit: Herb. Habitat: Upland moorland, 3000–4321 m. Vouchers: Mrs J Adamson 484 (EA), Muthama M & Berit Gehrke 142 (EA), Milne-Redhead E & Hedberg O 994 (EA). Native distribution range: Ethiopia to E DR Congo.

***Ranunculusvolkensii* Engl.** – Habit: Herb. Habitat: Peaty grassland, 2550–3400 m. Vouchers: Dummer RA 3312 (K), Muthama M & Gehrke B 143 (EA), Knox EB 3840 (EA). Native distribution range: W Central Ethiopia, Mountains of E Central & E Tropical Africa.

***Thalictrumrhynchocarpum* Quart.-Dill. & A.Rich.** – Habit: Herb. Habitat: Path sides, forest, bushland on stream sides, grassland, 1550–3275 m. Vouchers: Lugard 660 (EA), Irwin PH 309 (EA), Rono et al. SAJIT-PR 0047 (EA, HIB). Native distribution range: Bioko to Ethiopia & S Africa.

#### F111. RHAMNACEAE

4 Genera, 5 species

***Helinusmystacinus* E.Mey. ex Steud.** – Habit: Woody climber. Habitat: Wooded grassland, forest margin, secondary bushland, 1450–2400 m. Vouchers: Cheseny CM 1 (BR), Rono et al. SAJIT-PR 0070 (EA, HIB). Native distribution range: Eritrea to S Africa.

***Rhamnusprinoides* L’Hér.** – Habit: Shrub or tree. Habitat: Forest edges, evergreen bushlands, thickets, 1750–3700 m. Vouchers: Lugard 576 (EA), Bamps PRJ 6493 (WAG), Rono et al. SAJIT-PR 0015 (EA, HIB). Native distribution range: Cameroon to Eritrea & S Africa.

***Rhamnusstaddo* A.Rich.** – Habit: Shrub or small tree. Habitat: Forest edges, bushland, woodland, 1750–3000 m. Voucher: Tweedie EM (1976) (REF). Native distribution range: Eritrea to Zimbabwe, Arabian Peninsula.

***Scutiamyrtina* (Burm.f.) Kurz** – Habit: Shrub or small tree. Habitat: Forest edges, woodland, 1750–2400 m. Vouchers: Tweedie DR 2508 (BR), Taylor G 3430 (S), Jackson THE 323 (EA), Taiti S 588 (EA), Lugard C 621 (EA). Native distribution range: Ethiopia to S Africa, W Indian Ocean, India to China (S Yunnan, SW Guangxi) and Indo-China.

***Ziziphusabyssinica* Hochst. ex A.Rich.** – Habit: Tree or shrub. Habitat: Scattered-tree grassland, 700–2200 m. Voucher: Dale IR 3092 (BR). Native distribution range: Tropical Africa.

#### F112. RHIZOPHORACEAE

1 Genus, 1 species

***Cassipoureamalosana* (Baker) Alston** – Habit: Tree. Habitat: Forest, 2250–3000 m. Vouchers: Styles B 293 (BR), Rono et al. SAJIT-PR 0008 (EA, HIB). Native distribution range: Cameroon to Eritrea and S Africa.

#### F113. ROSACEAE

5 Genera, 18 species

***Alchemillaabyssinica* Fres.** – Habit: Herb. Habitat: Heath, bamboo zones, 2700–4100 m. Voucher: Dummer RA 3559 (EA). Native distribution range: Ethiopia to Kenya.

***Alchemillaargyrophylla* Oliv.** – Habit: Herb. Habitat: Upland moor, moor grassland, often dominant in rocky grounds, 2250–4500 m. Voucher: Rono et al. SAJIT-PR 0250 (EA, HIB). Native distribution range: S Sudan to N Tanzania.

***Alchemillacryptantha* Steud. ex A.Rich.** – Habit: Herb. Habitat: Alpine, montane grassland, 1725–3200 m. Vouchers: Strid A 3431 (EA), Hedberg O 104, 849 (EA), Knox EB 2635 (EA). Native distribution range: Cameroon to Eritrea and S Africa, Madagascar.

**Alchemillaelgonensis Mildbr. – Habit: Shrub. Habitat: Upland moor, moist bamboo-thickets, 2700–4350 m. Vouchers: Lugard 369 (EA), Tweedie 24 (EA), Hedberg KO 4452 (BR), Gillett JB 18437 (WAG), Liebenberg 1616 (EA), Sounders & Hancock 60 (EA), Thomson AS 559 (EA). Native distribution range: E Uganda to W Central Kenya.

***Alchemillaellenbeckii* Engl.** – Habit: Herb or shrub. Habitat: Moist bamboo tickets, moor grassland, forest margin, 2100–4250 m. Vouchers: Hedberg O 843 (EA), Lugard EJ 2961 (EA), Knox EB 2621 (EA), Arnstein KL 5733 (EA), Hedberg O 4546 (EA). Native distribution range: Ethiopia to E Central & E Tropical Africa.

***Alchemillajohnstonii* Oliv.** – Habit: Shrub. Habitat: Moist bamboo tickets, moor grassland, 2900–4321 m. Vouchers: Gillett JB 18448 (EA), Hedberg O 1954, 272, 1958 (EA), Knox EB 2610 (EA). Native distribution range: E Central & E Tropical African Mountains.

***Alchemillakiwuensis* Engl.** – Habit: Herb. Habitat: Grassland, forest, bamboo thickets, 200–3000 m. Voucher: Rono et al. SAJIT-PR 0162 (EA, HIB). Native distribution range: Cameroon to Eritrea and S Africa.

*Alchemillamicrobetula T.C.E.Fr. – Habit: Herb. Habitat: Rare alpine, 3500–4150 m. Vouchers: Hedberg O 896, 967, 4511, 4516, 4523 (EA), Granvik H sn (S). Native distribution range: Ethiopia to E Central & E Tropical Africa.

***Alchemillavolkensii* Engl.** – Habit: Herb. Habitat: Upland bushlands, moist bamboo thickets, 1500–2900 m. Vouchers: Tothill BH 2464, 2341 (EA), Thomas AS 531 (EA). Native distribution range: Uganda, N Tanzania.

***Cliffortianitidula* R.E.Fr. & T.C.E.Fr.** – Habit: Shrub. Habitat: Moorland, 2920–3490 m. Voucher: Agnew ADQ (2013) (REF). Native distribution range: Kenya to South Africa.

***Hageniaabyssinica* (Bruce) J.F.Gmel.** – Habit: Tree. Habitat: Forest, moist bamboo thickets, 2250–3600 m. Voucher: Tothill BH 2297 (MO). Native distribution range: Burundi, Eritrea, Ethiopia, Kenya, Malawi, Rwanda, Sudan, Tanzania, Uganda, Zambia, Zaïre.

***Prunusafricana* (Hook.f.) Kalkman** – Habit: Tree. Habitat: Riverine Forest, 900–3000 m. Voucher: Lugard 445 (EA). Native distribution range: Ghana to Ethiopia and S Africa, Comoros, Madagascar.

***Rubusapetalus* Poir.** – Habit: Shrub. Habitat: Montane forests, bushlands, 1600–3000 m. Vouchers: Tweedie EM (1976) (REF), Agnew ADQ (2013) (REF). Native distribution range: Nigeria to Eritrea and S Africa, W Indian Ocean, N Yemen.

**Rubusfriesiorumsubsp.elgonensis (Gust.) R.A.Graham – Habit: Shrub. Habitat: Forest, 3300 m. Vouchers: Liebenburg 1645 (EA), Thomas AS 591 (EA), Synge 913 (EA), Dummer RA 3541 (MO), Synge PM 913 (K). Native distribution range: Uganda (Mt Elgon).

^**EX**^***Rubusniveus* Thunb.** – Habit: shrub. Habitat: Disturbed woodlands, 2400–3000 m. Voucher: Agnew ADQ (2013) (REF). Native distribution range: Afghanistan to Central China and Indo-China.

***Rubuspinnatus* Willd.** – Habit: Shrub. Habitat: Forest, moist bamboo thickets, 1950–2200 m. Voucher: Lugard 509 (EA). Native distribution range: Ascension, St Helena, Tropical & S Africa.

***Rubussteudneri* Schweinf.** – Habit: Shrub. Habitat: Montane forests, 2000–3480 m. Vouchers: Thomas AS 489 (EA), Tothill BH 238 (EA), Saundy & Hancock 103 (EA), Mwangangi OM 307 (BR), Rono et al. SAJIT-PR 0123, SAJIT-PR 0171 (EA, HIB). Native distribution range: Ethiopia to E Central Tropical Africa.

***Rubusvolkensii* Engl.** – Habit: Herb or shrub. Habitat: Forest, bamboo zone, 2600–4100 m. Vouchers: Tweedie EM (1976) (REF), Agnew ADQ (2013) (REF). Native distribution range: Ethiopia to N Tanzania.

#### F114. RUBIACEAE

24 Genera, 58 species

***Anthospermumherbaceum* L.f.** – Habit: Herb. Habitat: Forest edges, grassland, woodland, heathland, 1560–3240 m. Vouchers: Tweedie 1997 (K), Wesche K 1438 (K), Irwin PH Mrs 57 (K), Lewis WH 5959 (K). Native distribution range: SW Arabian Peninsula, Eritrea to S Africa.

***Anthospermumusambarense* K.Schum.** – Habit: Shrub. Habitat: Alpine bushland, forest, 2150–4050 m. Vouchers: Dummer RA 3348 (MO), Dale 48 (MO). Native distribution range: S Sudan to S Tropical Africa.

***Canthiumoligocarpum* Hiern** – Habit: Shrub or tree. Habitat: Forest, 1800–2600 m. Vouchers: Dale 88 (MO), 455 (EA). Native distribution range: Ethiopia to W Mozambique.

***Coffeaeugenioides* S.Moore** – Habit: Bush or small tree. Habitat: Riverine forest, woodland, 1750–2400 m. Voucher: Tweedie EM (1976) (REF). Native distribution range: South Sudan to E Central & E Tropical Africa.

***Dolichopentasdecora* (S.Moore) Karehed & B.Bremer** – Habit: Woody herb. Habitat: Wooded grassland, 1590–2700 m. Vouchers: Tweedie EM (1976) (REF), Agnew ADQ (2013) (REF). Native distribution range: Tropical Africa.

***Dolichopentaslongiflora* (Oliv.) Karehed & B.Bremer** – Habit: Woody herb. Habitat: Woodlands with combretum, 1590–2400 m. Vouchers: Holm Å 103 (S), Lewis WH 5962 (US), Rono et al. SAJIT-PR 0055 (EA, HIB). Native distribution range: E Central & E Tropical Africa to Malawi.

**^EX^*Galiumaparine* L.** – Habit: Climbing herb. Habitat: Cultivation, wasteland, 1600–2790 m. Voucher: Andersen R 89 (S). Native distribution range: Macaronesia to Temperate Eurasia.

***Galiumaparinoides* Forssk.** – Habit: Herb. Habitat: Upland forest, rocky streams, grassland, 1679–3700 m. Vouchers: Tothill BH 2374 (EA), Leibenberg 1630 (EA), Knox EB 2641 (EA), Knox A.B 3777 (EA). Native distribution range: Eritrea to Tanzania, SW Arabian Peninsula.

***Galiumglaciale* K.Krause** – Habit: Herb. Habitat: Alpine zone, along river banks 3510–4350 m. Vouchers: Wesche K 1135 (K), Hedberg O 892 (K). Native distribution range: DR Congo to E Tropical Africa.

***Galiumossirwaense* K.Krause** – Habit: Straggling herb. Habitat: Grassland, montane forest edge, 1900–3750 m. Vouchers: Lugard EJ 400a (K), Wesche K 462 (BR), Hooper SS; Townsend CC 1461 (K), Lisowski S 10577 (K), Hedberg O 4476 (EA, K), Hedberg O 889 (S), Liebenberg 1619 (EA, K). Native distribution range: E Tropical African Mountains to N Mozambique.

***Galiumruwenzoriense* (Cortesi) Ehrend. ex Hedberg** – Habit: Climbing herb. Habitat: Forest, forest clearing, alpine, moorland, rocky grounds, 2350–4200 m. Vouchers: Tothill BH 2258 (EA), Tweedie 2543 (EA), Lugard 365 (EA), Wesche K 10 (BR). Native distribution range: E Central & E Tropical African Mountains.

***Galiumsimense* Fresen.** – Habit: Climbing herb. Habitat: Wet grassland, woodland, forest, 1750–2700 m. Voucher: Tweedie 2412 (EA). Native distribution range: Tropical African Mountains.

**^EX^*Galiumtanganyikense* Ehrend. & Verdc.** – Habit: Herb. Habitat: Grassland, among rocks, streamsides, 2400–2820 m. Vouchers: Lugard EJ 365 (K). Native distribution range: Tanzania.

***Galiumthunbergianum* Eckl. & Zeyh.** – Habit: Herb. Habitat: Montane forest, 2050–3750 m. Vouchers: Knox EB, 3836 (EA), Knox EB 3787 (EA), Dummer RA 3472 (US), Lugard EJ 365 (K). Native distribution range: Cape Verde, Cameroon Sudan and S Africa, Madagascar.

**Gardeniaternifoliasubsp.jovis-tonantis (Welw.) Verdc.** – Habit: Shrub or small tree. Habitat: Forest, grassland, woodland, 1750–2250 m. Voucher: Tweedie EM (1976) (REF). Native distribution range: Tropical Africa.

***Heinseniadiervilleoides* K.Schum.** – Habit: Shrub or small understory tree. Habitat: Forest edges, thickets, 800–2250 m. Vouchers: Thomson in Eggeling 3175 (EA), Eggeling WJ 3947 (MO). Native distribution range: S Sudan to Zimbabwe.

***Hymenodictyonfloribundum* B.L.Rob.** – Habit: Shrub or small tree. Habitat: Outcrop rock, bushland, woodland, wooded grassland, 1800–2400 m. Vouchers: Tweedie 2633, Adamson J 453 (EA), Synnott TJ 541 (EA), Rono et al. SAJIT-PR 0093 (EA, HIB), JR Dale 3091 (MA). Native distribution range: Tropical Africa to NW Namibia.

***Keetiagueinzii* (Sond.) Bridson** – Habit: Shrub or liana. Habitat: Woodlands, 1750–2400 m. Voucher: Tweedie EM (1976) (REF). Native distribution range: Cameroon to Ethiopia and S Africa.

***Kohautiaaspera* (Heyne ex Roth) Bremek.** – Habit: Herb. Habitat: Grassland, open bushland, 1750–2250 m. Voucher: Tweedie 2008 (EA). Native distribution range: Cape Verde, Dry Tropical & S Africa, Arabian Peninsula, Pakistan to India.

***Kohautiacoccinea* Royle** – Habit: Herb. Habitat: Grasslands, wet savanna, 1250–2500 m. Voucher: Lewis WH 5952 (US). Native distribution range: Tropical Africa, Yemen, Iran to Nepal.

***Oldenlandiacorymbosa* L.** – Habit: Herb. Habitat: Short grassland, bushlands, shallow pans, 001–2500 m. Vouchers: Lewis WH 5977, 5953 (US). Native distribution range: Tropical & Subtropical Old World.

**Oldenlandiacorymbosavar.linearis (DC.) Verdc.** – Habit: Herb. Habitat: Grassland, 1750–2250 m. Vouchers: Lewis WH 5977 (EA), Tweedie EM 757 (K). Native distribution range: Africa, Indian Subcontinent.

**Oldenlandiacorymbosavar.nana (Bremek.) Verdc.** – Habit: Herb. Habitat: Short grassland, paddocks, path sides, 780–2280 m. Vouchers: Lewis WH 5970 (K), Lind EM 245 (K). Native distribution range: E Tropical Africa.

***Oldenlandiaherbacea* (L.) Roxb.** – Habit: Herb. Habitat: Forest, grassland 1750–2250 m. Voucher: Tweedie EM (1976) (REF). Native distribution range: Africa, Indian Subcontinent to Andaman Islands.

***Oldenlandiamonanthos* Hiern** – Habit: Mat forming herb. Habitat: Montane grassland, roadsides, 1350–3500 m. Vouchers: Dummer RA 8684 (EA), Wood OH 114 (EA), Tweedie 712 (EA), Synge PM 1064 (S). Native distribution range: Ethiopia to E Tropical Africa.

***Oldenlandiascopulorum* Bullock** – Habit: Herb. Habitat: Grassland, rock outcrop, 1200–2500 m. Vouchers: Lugard EJ; Lugard C 346 (K). Native distribution range: E Central & E Tropical Africa.

***Paraknoxiaparviflora* (Stapf ex Verdc.) Verdc.** – Habit: herb. Habitat: Short grasslands, bushland, old cultivations, 350–2280 m. Vouchers: Lewis WH 5967 (EA, S) 5954 (US), Tweedie EM 515 (BR). Native distribution range: Central African Republic to Kenya and Zimbabwe.

***Pavettaabyssinica* Fresen.** – Habit: Bush or small tree. Habitat: Woodland, riverine forest, 1750–3000 m. Vouchers: Hamilton, PH 76/ 779 (U), Rono et al. SAJIT-PR 0178 (EA, HIB). Native distribution range: Eritrea to Tanzania.

***Pavettaoliveriana* Hiern** – Habit: Bush or small tree. Habitat: Riverine forest, woodland, 1750–2400 m. Voucher: Irwin PH 32 (WAG). Native distribution range: Eritrea to Burundi and Tanzania.

***Pavettaternifolia* Hiern** – Habit: Shrub or small tree. Habitat: Riverine thickets, grassland, forest, woodland, 1150–1950 m. Voucher: Rono et al. SAJIT-PR 0191 (EA, HIB). Native distribution range: East Central & Eastern Tropical Africa.

***Pentanisiaouranogyne* S.Moore** – Habit: herb. Habitat: Disturbed grassland, along the track, 300–2400 m. Vouchers: Lewis WH 5965 (US), Holm Å 102 (S), Andersen R 76 (S). Native distribution range: Ethiopia to Tanzania.

***Pentanisiaschweinfurthii* Hiern** – Habit: Pyrophytic herb. Habitat: Grassland, 840–2250 m. Vouchers: Evans James (EA). Native distribution range: Nigeria to South Sudan and S Tropical Africa.

***Pentasarvensis* Hiern** – Habit: Pyrophytic herb. Habitat: Grassland, 1200–1530 m. Voucher: James 1905 (EA). Native distribution range: Nigeria to South Sudan and SW Kenya.

***Pentaslanceolata* (Forssk.) Deflers** – Habit: Shrub or woody herb. Habitat: Forest, 1200–2830 m. Vouchers: Synge PM 1079 (S). Native distribution range: Ethiopia to Mozambique, Comoros, Arabian Peninsula.

^**EX**^**Pentaslanceolatasubsp.quartiniana (A.Rich) Verdc.** – Habit: Herb or subshrub. Habitat: Forest, 1750–2400 m. Voucher: Tweedie EM (1976) (REF). Native distribution range: Eritrea to DR Congo.

**Pentaslanceolatavar.leucaster (K.Krause) Verdc.** – Habit: Herb or subshrub. Habitat: Forest margin, 1200–2830 m. Voucher: Tweedie EM (1976) (REF). Native distribution range: E DR Congo to Ethiopia and N Tanzania.

***Pentaspubiflora* S.Moore** – Habit: Herb or shrubby herb. Habitat: Woodlands, montane and riverine forests, 1230–2550 m. Vouchers: Tweedie 1267 (EA), Lewis WH 5963 (US), Irwin PH 55 (S). Native distribution range: Cameroon to Uganda and Mozambique.

**Pentaszanzibaricavar.tenuifolia Vatke – Habit: Herb or shrubby herb. Habitat: Evergreen forest, 1800–2440 m. Voucher: Amani 9644 (EA). Native distribution range: Kenya (Trans-Nzoia).

***Phyllopentasschimperi* (Hochst.) Y.D.Zhou & Q.F.Wang** – Habit: Shrub or woody herb. Habitat: Forest edge, 1450–2710 m. Voucher: Rono et al. SAJIT-PR 0120 (EA, HIB). Native distribution range: Tropical Africa.

***Psychotriafractinervata* E.M.A.Petit** – Habit: Shrub or small tree. Habitat: Forest, 1500–2600 m. Vouchers: Wood 113b (EA, MO). Native distribution range: E Tropical Africa.

***Psychotriaorophila* E.M.A.Petit** – Habit: Shrub or small tree. Habitat: Forest, 1650–3000 m. Vouchers: Wood C113 (EA), Dummer RA 3582 (EA), Snowden JD 1196 (EA), Hedberg O (WAG). Native distribution range: South Sudan to E Tropical Africa.

***Psydraxparviflorus* (Afzel.) Bridson** – Habit: Shrub or tree. Habitat: Forest, thicket, grassland, 1400–2250 m. Voucher: Jackson THE 322 (EA). Native distribution range: Tropical Africa.

**Psydraxparviflorussubsp.rubrocostatus (Robyns) Bridson** – Habit: Shrub or tree. Habitat: Forest, 1750–2750 m. Vouchers: Thomson T 1798 (EA), St Clair-Thompson T 1798 (EA). Native distribution range: South Sudan to Malawi.

***Rothmanniamanganjae* (Hiern) Keay** – Habit: Shrub or tree. Habitat: Riverine forest, woodland, 1750–2400 m. Voucher: Jex -Blake in CM 6864 (EA). Native distribution range: Cameroon, Kenya to S Tropical Africa.

***Rothmanniaurcelliformis* Bullock ex Robyns** – Habit: Bush or small tree. Habitat: Riverine forest, woodland, 1750–2400 m. Voucher: Tweedie EM (1976) (REF). Native distribution range: Tropical Africa.

***Rubiacordifolia* L.** – Habit: Climbing herb. Habitat: Woodland, 1240–3120 m. Voucher: Beentje HJ 1929 (WAG). Native distribution range: Greece, Sudan to S Africa, Asia.

***Rutideaorientalis* Bridson** – Habit: Shrub or climber. Habitat: Moist forest, 375–2400 m. Voucher: Eggeling 3627 (EA). Native distribution range: Kenya to S Tropical Africa.

***Rytigyniaacuminatissima* Robyns** – Habit: Shrub or small tree. Habitat: Riverine forest understory, 1650–2400 m. Vouchers: Maitland 1231 & 1245 (EA), Dale in FD 3119 (EA), Hamilton & Perrott 76/770 (EA), Snowden JD 907/b (WAG). Native distribution range: E Central & E Tropical Africa.

***Rytigynianeglecta* Robyns** – Habit: Shrub or small tree. Habitat: Riverine forest, 1750–2400 m. Voucher: Tweedie EM (1976) (REF). Native distribution range: Cameroon to Ethiopia and Kenya.

***Rytigyniauhligii* (K.Schum. & K.Krause) Verdc.** – Habit: Shrub or tree. Habitat: Riverine forest, woodland, 1750–2400 m. Voucher: Dale IR 3389 (BR). Native distribution range: Kenya to S Tropical Africa.

***Spermacoceminutiflora* (K.Schum.) Verdc.** – Habit: Herb. Habitat: Wooded grassland, 1500–2350 m. Vouchers: Lugard EJ 231 (K), Lewis WH 5971 (K), Tweedie 516, 739, 807, 2479, (K). Native distribution range: S Sudan to Kenya.

***Spermacoceprinceae* (K.Schum.) Verdc.** – Habit: Herb. Habitat: Grassland, bamboo forest, forest, 2250–3150 m. Voucher: Tweedie EM (1976) (REF). Native distribution range: Tropical Africa.

**^EX^*Spermacocepusilla* Wall.** – Habit: Herb. Habitat: Grassland, woodland, roadsides, rocky areas, 1750–2250 m. Voucher: Lewis WH 5966 (US). Native distribution range: S China to Tropical Asia.

***Spermacocesphaerostigma* Oliv.** – Habit: Herb. Habitat: Grassland, 1750–2250 m. Voucher: Tweedie EM (1976) (REF). Native distribution range: Tropical Africa, Arabian Peninsula.

***Vangueriaapiculata* K.Schum.** – Habit: Shrub or small tree. Habitat: Forest, grassland, riverine, marshes, rock outcrop, 2190–2251 m. Vouchers: Lugard 234A (EA), Rono et al. SAJIT-PR 0001 (EA, HIB). Native distribution range: Ethiopia to S Tropical Africa.

**Vangueriainfaustasubsp.rotundata (Robyns) Verdc.** – Habit: Shrub or small tree. Habitat: Forest, bushlands, rocky thickets, 30–2100 m. Vouchers: Padwa JH 3 (EA), Rono et al. SAJIT-PR 0024 (EA, HIB). Native distribution range: Sudan to S Tropical Africa.

***Vangueriamadagascariensis* J.F.Gmel.** – Habit: Shrub or small tree. Habitat: Riverine forest, woodland, 1750–2400 m. Voucher: Tweedie EM (1976) (REF). Native distribution range: Tropical & S Africa, Comoros, Madagascar.

***Vangueriavolkensii* K.Schum.** – Habit: Shrub, subscandent shrub or small tree. Habitat: Riverine forest, woodland, 1750–2400 m. Vouchers: Bridson D 65 (EA/BR/K/WAG), Mrs Tweedie 1315 (S), Andersen R 236 (S), Lugard C & Lugard EJ 622 (EA), Fairbairn G 894 (EA). Native distribution range: Ethiopia to S Tropical Africa.

#### F115. RUTACEAE

2 Genera, 2 species

***Veprisnobilis* (Delile) Mziray** – Habit: Shrub or tree. Habitat: Lower edge of forest, 1750–2400 m. Voucher: Tweedie EM (1976) (REF). Native distribution range: Ethiopia to S Tropical Africa.

***Zanthoxylumasiaticum* (L.) Appelhans, Groppo & J.Wen** – Habit: Shrub or liane. Habitat: Lower edge of forest, wooded grassland, bushland, 1750–2800 m. Vouchers: Jackson THE 302 (EA), Lugard C & Lugard EJ 537 (EA) Fack WE 284 (EA). Native distribution range: Ethiopia to Eswatini, W Indian Ocean, Tropical & Subtropical Asia.

#### F116. SALICACEAE

6 Genera, 7 species

***Caseariabattiscombei* R.E.Fr.** – Habit: Tree. Habitat: Upland moist forest, 2250–3000 m. Vouchers: St Clair-Thompson in Eggeling 3954 & 3955 (EA), Fairbairn G 3384 (BR). Native distribution range: E & S Tropical Africa.

***Dovyalisabyssinica* (A.Rich.) Warb.** – Habit: Shrub or tree. Habitat: Moist forest, open wooded grassland, 1750–3000 m. Vouchers: Snowden JD 1084 (EA), Bamps PRJ 6516 (EA), Hamilton & Perrott 76/364 (EA), Rono et al. SAJIT-PR 0050 (EA, HIB). Native distribution range: NE & E Tropical Africa to Malawi.

***Dovyalismacrocalyx* Warb.** – Habit: Shrub or small tree. Habitat: Moist Forest, bushland, wooded grassland, 1750–3000 m. Voucher: Major C & Lugard EJ 577 (EA). Native distribution range: S Sudan to S Tropical Africa.

***Flacourtiaindica* (Burm.f.) Merr.** – Habit: Shrub or tree. Habitat: Woodland, wooded grassland, bushland, 2250–3000 m. Vouchers: Snowden JD 826 (EA), Major Jack 2857 (EA), Jackson THE 422 (MA), Trapnell CG 2367 (EA), Katende AB 3606 (MO), Rono et al. SAJIT-PR 0099 (EA, HIB). Native distribution range: Ethiopia to S Africa, SE China to Tropical Asia.

***Oncobaspinosa* Forssk.** – Habit: Shrub or tree. Habitat: Woodland, riverine forest, bushland, 1750–2400 m. Voucher: Padwa JH 2 (EA). Native distribution range: Tropical & S Africa, SW Arabian Peninsula.

***Salixmucronata* Thunb.** – Habit: Tree. Habitat: Riverine forest, bushland, grassland, 1550–2600 m. Vouchers: Dale U 11 (EA), Kimera 21 (EA), Thomas 2607 (EA). Native distribution range: Africa to Arabian Peninsula.

***Trimeriagrandifolia* (Hochst.) Warb.** – Habit: Shrub or tree. Habitat: Woodland, riverine forest, wooded grassland, 1750–2450 m. Voucher: Jack C 263 (EA). Native distribution range: E Zimbabwe to S Africa.

#### F117. SANTALACEAE

3 Genera, 7 species

***Osyrislanceolata* Hochst. & Steud.** – Habit: Shrub or a small tree. Habitat: Woodland, upland evergreen forest and mist forest, 1750–2700 m. Vouchers: Eggeling 2485 (EA), Rono et al. SAJIT-PR 0084 (EA, HIB). Native distribution range: S Iberian Peninsula Baleares, Canary Islands, Sahara to S Africa, Socotra, Indian Subcontinent to S China and Indo-China.

***Thesiumkilimandscharicum* Engl.** – Habit: Herb. Habitat: Montane grassland, heath scrub, afro alpine zone, 2200–4200 m. Voucher: Jex-Blake in Bally 1955 (EA). Native distribution range: Kenya to N Malawi.

***Thesiummukense* A.W.Hill** – Habit: Herb. Habitat: Burnt grassland, 1800–2700 m. Voucher: Agnew ADQ (2013) (REF). Native distribution range: Kenya to S Africa.

***Thesiumschweinfurthii* Engl.** – Habit: Herb. Habitat: Upland grassland, woodland, mixed bushland, 1050–2300 m. Voucher: Tweedie 3555 (EA). Native distribution range: Nigeria to W & S Ethiopia and Zambia.

***Viscumschimperi* Engl.** – Habit: Shrub. Habitat: Dry evergreen forest, bushland, 1160–2140 m. Voucher: Tweedie EM 1166 (K). Native distribution range: Eritrea to Tanzania, Arabian Peninsula.

***Viscumtriflorum* DC.** – Habit: Shrub. Habitat: Forest, 100–2300 m. Voucher: Agnew ADQ (2013) (REF). Native distribution range: São Tomé, Central African Republic to N Somalia and S Africa, W Indian Ocean.

***Viscumtuberculatum* A.Rich.** – Habit: Shrub. Habitat: Dry evergreen forest, woodland, bushland, 1650–2400 m. Vouchers: Tweedie DR 130 (BR), Cheseny CM 19 (BR), Rono et al. SAJIT-PR 0061 (EA, HIB). Native distribution range: Eritrea to South Africa.

#### F118. SAPINDACEAE

2 Genera, 5 species

***Allophylusabyssinicus* Radlk.** – Habit: Tree. Habitat: Woodland, moist forest, evergreen thickets, 1750–2550 m. Vouchers: Styles 298 (EA), Tweedie 2243 (EA). Native distribution range: Eritrea to S Tropical Africa.

***Allophylusafricanus* P.Beauv.** – Habit: Shrub or tree. Habitat: Forest, grassland, 1750–2400 m. Vouchers: Tweedie 2415 (EA), Jackson THE 320 (EA), Rono et al. SAJIT-PR 0200 (EA, HIB). Native distribution range: Tropical & S Africa.

***Allophylusferrugineus* Taub.** – Habit: Tree or shrub or climber. Habitat: Forest, stream sides, 1050–2400 m. Vouchers: Lugard Mrs C 671 (EA). Native distribution range: S Nigeria to Ethiopia and Namibia.

***Allophylusrubifolius* Engl.** – Habit: Shrub or medium sized tree. Habitat: Grassland, thickets, cultivations, riverine, 0–2250 m. Vouchers: Tweedie 2016 (EA), 2016B, Jackson THE 316 (EA). Native distribution range: Eritrea to S Africa, Arabian Peninsula.

***Deinbolliakilimandscharica* Taub.** – Habit: Small tree or shrub. Habitat: Evergreen forest, upland moist forest, 1100–2400 m. Vouchers: Snowden JD 796 (EA, MO). Native distribution range: Ethiopia to E Central & E Tropical Africa.

#### F119. SAPOTACEAE

3 Genera, 3 species

***Aningeriaadolfi-friederici* (Engl.) Robyns & Gilbert** – Habit: Tree. Habitat: Forest, 2250–3000 m. Vouchers: Eggeling WJ 3956 (EA, MO). Native distribution range: SW Ethiopia to Zimbabwe.

***Manilkarabutugi* Chiov.** – Habit: Tree. Habitat: Upland forest, riverine forest, 1500–2300 m. Voucher: Eggeling WJ 5734 (EA). Native distribution range: Ethiopia to Tanzania.

***Mimusopskummel* Bruce ex A.DC.** – Habit: Tree or shrub. Habitat: Riverine forest, upland evergreen forest, 1200–2250 m. Voucher: Snowden JD 1066 (EA). Native distribution range: Tropical Africa.

#### F120. SCROPHULARIACEAE

8 Genera, 12 species

***Buddlejapolystachya* Fresen.** – Habit: Shrub. Habitat: Grassland, woodland, forest, 1750–3000 m. Vouchers: Bamps PRJ 6492 (WAG), Katende AB 3602 (MO), Beentje HJ 1926 (WAG), Rono et al. SAJIT-PR 0206 (EA, HIB). Native distribution range: NE Tropical Africa to N Tanzania, SW Arabian Peninsula.

***Cycniopsishumifusa* Engl.** – Habit: Herb. Habitat: Wet grassland, 1000–2500 m. Voucher: Evans James sn (EA). Native distribution range: Ethiopia to N Tanzania, and Yemen.

***Diclisbambuseti* R.E.Fr.** – Habit: Herb. Habitat: Forest, bamboo zone, 2000–3720 m. Vouchers: Tweedie 842 (BR), Rono et al. SAJIT-PR 0238, SAJIT-PR 0268 (EA, HIB). Native distribution range: SW Ethiopia to E Tropical Africa.

***Hebenstretiaangolensis* Rolfe** – Habit: Herb or shrub. Habitat: Grassland, rocky heathland, 1600–4000 m. Vouchers: Wesche K 245 (EA), Taylor G 3450 (S), Rono et al. SAJIT-PR 0150 (EA, HIB). Native distribution range: Eritrea to S Africa.

***Hebenstretiadentata* L.** – Habit: Shrub. Habitat: Upper forest edge, moorland, 3000–4321 m. Voucher: Tweedie EM (1976) (REF). Native distribution range: Ethiopia to E Central & E Tropical Africa, South Africa.

***Limosellaafricana* Glück** – Habit: Herb. Habitat: Ephemeral pools, alpine peaty soils, 1600–4200 m. Vouchers: Lind EM 291 (EA), Forbes 280 (EA), Rono et al. SAJIT-PR 0039 (EA, HIB). Native distribution range: Cameroon, Mali, Eritrea to N Tanzania, Namibia to South Africa, Yemen.

***Limosellamacrantha* R.E.Fr.** – Habit: Herb. Habitat: Moorland zone, upper forest margin, moorland, 2500–4300 m. Vouchers: Wood GHS 912(K), Taylor G 3670 (BR) 9670 (BR), Gillett JB 18480 (K, BR), Wesche K 1094 (EA). Native distribution range: SE Ethiopia to Rwanda and N Tanzania, Yemen.

***Limosellamajor* Diels** – Habit: Herb. Habitat: Temporary pools, grassland, 1800–2700 m. Voucher: Tweedie 2848 (EA). Native distribution range: Eritrea to S Africa.

***Rhabdotospermabrevipedicellata* (Engl.) Hartl** – Habit: Herb. Habitat: Upland grassland, woodland forest, 3600–4100 m. Voucher: Liebenberg (EA). Native distribution range: Ethiopia to E Central & E Tropical Africa.

***Rhabdotospermascrophulariifolia* (Hochst.) Hartl** – Habit: Herb. Habitat: Montane grassland, upper forest margin, 1800–3600 m. Vouchers: Thomas AS 525 (EA), Lugard 491 (EA). Native distribution range: Cameroon, Ethiopia to Burundi and N Tanzania.

***Selagothomsonii* Rolfe** – Habit: Herb or shrub. Habitat: Dry subalpine heathland, 1860–3380 m. Vouchers: Hedberg O (UPS), James E sn (K). Native distribution range: Kenya to Tanzania.

***Zaluzianskyaelgonensis* Hedberg** – Habit: Herb. Habitat: Alpine zone on rocky grounds, 3800 m. Voucher: Hedberg O 4478 (K). Native distribution range: SE Uganda, N Tanzania.

#### F121. SOLANACEAE

5 Genera, 24 species

^**EX**^***Daturastramonium* L.** – Habit: Herb. Habitat: Wasteland, woodland, grassland, 1750–2400 m. Voucher: Tweedie 1176 (EA). Native distribution range: Texas to Central America, Caribbean.

***Discopodiumpenninervium* Hochst.** – Habit: Tree or small shrub. Habitat: Moorland, upper montane, woodland, 2100–3500 m. Vouchers: Wood 254 (EA), Katende & Lye 466 (EA), Tweedie 1103 (EA), Rono et al. SAJIT-PR 0212 (EA, HIB). Native distribution range: Benin to Eritrea and Mozambique.

**^EX^*Physalisperuviana* L.** – Habit: Herb. Habitat: Cultivations, secondary bushland, forest, 900–2500 m. Vouchers: Lugard & Lugard 641 (EA), Tiyoy LM 1334 (MNHN). Native distribution range: Bolivia to W Brazil

***Solanumaculeastrum* Dunal** – Habit: Shrub or small tree. Habitat: Upland forest, 2170–2400 m. Vouchers: Lugard EJ 238 (K), Cheseny, CM 21 (K), Tweedie J 696 (K), Mwangangi OM 461 (K), Rono et al. SAJIT-PR 0104 (EA, HIB). Native distribution range: Nigeria to South Sudan and S Africa.

**^EX^*Solanumaculeatissimum* Jacq.** – Habit: Herb or shrub. Habitat: Montane forest, 1425–2640 m. Vouchers: Tweedie EM 715 (K), Symes YE 521 (K), Hedberg, KO 280 (K). Native distribution range: SE & S Brazil to S Central Paraguay.

***Solanumanguivi* Lam.** – Habit: Woody herb. Habitat: Cultivation, 1230–2700 m. Vouchers: Lugard EJ 291 (K), Mwangangi OM; Kariuki F 375 (K), Katende T; Sheil D 1150 (K), Holm Å 91 (S), Hedberg O 279 (K), Synge PM1071 (NHMUK). Native distribution range: Tropical & S Africa, Comoros, Madagascar.

***Solanumcampylacanthum* Hochst. ex A.Rich.** – Habit: Herb or shrub. Habitat: Wooded grassland, grassland, roadsides, cultivation, 1000–2000 m. Vouchers: Padwa JH 6 (K), Lugard EJ 90 (K). Native distribution range: Eritrea to S Africa.

^**EX**^***Solanumcapsicoides* All.** – Habit: Herb or shrub. Habitat: Forest, woodland, 1750–3000 m. Voucher: Tweedie EM (1976) (REF). Native distribution range: S Tropical America.

^**EX**^***Solanumdasyphyllum* Schumach. & Thonn.** – Habit: Woody herb. Habitat: Forest, 2250–3150 m. Voucher: Tweedie EM (1976) (REF). Native distribution range: Tropical & S Africa.

***Solanumgiganteum* Jacq.** – Habit: Shrub. Habitat: Grassland, riverine, bamboo zone, thickets, secondary bushland, 800–2450 m. Vouchers: Dummer RA 3611 (K), Coll S 1664 (K). Native distribution range: Tropical & S Africa.

***Solanumincanum* L.** – Habit: Herb or shrub. Habitat: Wet savanna, woodland, 15–2400 m. Vouchers: Tweedie EM (1976) (REF), Agnew ADQ (2013) (REF). Native distribution range: Africa, Arabian Peninsula, Iran to India.

***Solanumlanzae* J.-P.Lebrun & Stork** – Habit: Woody herb. Habitat: Bushland, thickets, 1200–2100 m. Voucher: Tweedie 1108(K). Native distribution range: Ethiopia to E Tropical Africa.

***Solanummauense* Bitter** – Habit: Shrub. Habitat: Montane forest, 1650–2640 m. Vouchers: Tweedie 734 (K), Holm Å 92 (S), Jack Mrs C 186 (K). Native distribution range: Kenya to Tanzania.

**^EX^*Solanummauritianum* Scop.** – Habit: Shrub or small tree. Habitat: Forest paths and margins, river banks, 1150–2800 m. Voucher: Katende T; Sheil D 656 (K). Native distribution range: SE & S Brazil to Argentina (Buenos Aires).

***Solanummemphiticum* J.F.Gmel.** – Habit: Herb or subshrub. Habitat: Forest, bushlands, grassland, riverbanks, 950–2450 m. Vouchers: Tweedie 1068 (K), 1586(K). Native distribution range: Jordan to Sinai, Eritrea to E Central & E Tropical Africa, SW Arabian Peninsula.

***Solanumnakurense* C.H.Wright** – Habit: Herb or subshrub. Habitat: Evergreen upland bushland, 900–3050 m. Vouchers: Snowden JD 889, (NHMUK), 889/a (S), Katende AB 3660 (MO), Hedberg O (UPS). Native distribution range: Ethiopia to E Tropical Africa.

***Solanumnigriviolaceum* Bitter** – Habit: Climbing subshrub. Habitat: Montane forest, 2130–2990 m. Vouchers: Major EJ; Lugard EJ 35 (K), Tweedie 850 (K), George Taylor 3722 (BM), Gerh Lindblom sn (S), Bridson DM 69 (BR), Bridson DM 89 (K), Rono et al. SAJIT-PR 0102 (EA, HIB). Native distribution range: Kenya.

**^EX^*Solanumnigrum* L.** – Habit: Herb. Habitat: Forest, woodland, grassland, shrubland, 0–3070 m. Vouchers: Synge PM 856 (NHMUK), Gerh Lindblom sn (S). Native distribution range: Eurasia, Macaronesia, N & NE Tropical Africa.

**^EX^*Solanumpseudospinosum* C.H.Wright** – Habit: Herb. Habitat: Montane grassland, bushland, woodland, bamboo thickets, 2000–2300 m. Vouchers: Cyril Lugard EJ 492 (K), Symes YE 392 (K). Native distribution range: Bioko, W Cameroon

***Solanumrunsoriense* C.H.Wright** – Habit: Shrub or liana, vine or herb. Habitat: Montane forest, 2400–3200 m. Vouchers: Sheil D 1813 (K), Synge PM 892 (BM), 1877 (BM), Tothill BH 2260 (K), Liebenberg LCC 1637 (K), Fishlock 43 (K), Samdy 14 (K), Samdy 14 (K). Native distribution range: E Central & E Tropical Africa.

***Solanumtarderemotum* Bitter** – Habit: Herb. Habitat: Moist forest margins, 550–2950 m. Vouchers: Lugard EJ 209 (K), Wesche K 500 (K), Katende T; Sheil D 1099 (K), Tiyoy L 1209 (K), Tweedie 725 (K), 1480 (K) 1481 (K). Native distribution range: Cape Verde, Tropical & S Africa.

***Solanumterminale* Forssk.** – Habit: Shrub, vine or liana. Habitat: Woodland, 1750–2400 m. Vouchers: Tweedie EM 714 (K), Snowden JD 889 (BR). Native distribution range: Tropical & S Africa, Arabian Peninsula.

**^EX^*Solanumwendlandii* Hook.f.** – Habit: Climber or liana. Habitat: Roadsides, montane forest, 1650–1800 m. Voucher: Padwa JH 42 (K). Native distribution range: Mexico to S Tropical America

***Withaniasomnifera* (L.) Dunal** – Habit: Herb or shrub. Habitat: Bushlands, grassland, wooded grassland, forests, river-banks, 2780 m. Voucher: Andersen R 306 (S). Native distribution range: S Europe to Central China, Africa to Myanmar.

#### F122. STILBACEAE

2 Genera, 2 species

***Hallerialucida* L.** – Habit: Shrub or small tree. Habitat: Undergrowth of montane forest and bushland, 900–2700 m. Vouchers: Dale 56 (EA), Bridson DM 71 (BR), Katende AB 3628 (MO), Bamps PRJ 6520 (BR, WAG), Tweedie DR 893 (BR), Rono et al. SAJIT-PR 0016 (EA, HIB). Native distribution range: Ethiopia to S Africa, Yemen.

***Nuxiacongesta* R.Br.** – Habit: Tree. Habitat: Woodland, upland rain forest, 1750–2700 m. Vouchers: Tweedie 1369 (EA), Bamps PRJ 6517 (WAG, BR), Rono et al. SAJIT-PR 0003 (EA, HIB), Mulugeta Kebede 188 (EA). Native distribution range: Tropical & S Africa, SW Arabian Peninsula.

#### F123. THYMELAEACEAE

1 Genus, 3 species

***Lasiosiphonglaucus* Fresen.** – Habit: Tree. Habitat: Forest, open woodland, 2250–3048 m. Vouchers: Wesche K 972 (K), Mwangangi OM 456 (BR), Lavranos J & Newton 17790 (BR), Irwin PH 40 (BR), Taylor G 3456 (BR), Dale IR 681 (EA). Native distribution range: Tropical Africa, S India, Sri Lanka.

***Lasiosiphonkraussianus* (Meisn.) Hutch. & Dalziel** – Habit: Herb. Habitat: Grassland, woodland, 1650–2650 m. Voucher: Tweedie EM (1976) (REF). Native distribution range: Tropical & S Africa.

***Lasiosiphonlampranthus* (Gilg)** – Habit: Shrub or tree. Habitat: Woodland, 1750–2400 m. Voucher: Chater-Jack 41 (EA). Native distribution range: Ethiopia to NW Tanzania.

#### F124. ULMACEAE

1 Genus, 1 species

***Chaetachmearistata* Planch.** – Habit: Tree. Habitat: Woodland, 1750–2400 m. Voucher: Tweedie EM (1976) (REF). Native distribution range: Tropical & S Africa, Madagascar.

#### F125. URTICACEAE

9 Genera, 11 species

***Australinaflaccida* (A.Rich.) Wedd.** – Habit: Herb. Habitat: Stream banks, forest, 2500–2650 m. Vouchers: Tweedie 2410, 2677, 3334 (EA). Native distribution range: Ethiopia to Kenya.

***Droguetiadebilis* Rendle** – Habit: Herb. Habitat: Forest, 1550–3090 m. Vouchers: Tweedie EM (1976) (REF), Agnew ADQ (2013) (REF). Native distribution range: E DR Congo to Kenya and N Tanzania.

***Droguetiainers* (Forssk.) Schweinf.** – Habit: Herb or subshrub. Habitat: Bamboo forest, upland moist forest, 1600–3250 m. Vouchers: Mwangangi OM 406 (BR), Tweedie EM 3330 (K), Naiga 252 (K). Native distribution range: Tropical Africa, SW Arabian Peninsula, South Africa, Madagascar.

***Girardiniadiversifolia* (Link) Friis** – Habit: Herb. Habitat: Wet forest, 1200–2500 m. Voucher: Tweedie 2240 (EA). Native distribution range: Tropical & Subtropical Old World.

***Laporteaalatipes* Hook.f.** – Habit: Herb. Habitat: Upper forest margin, bamboo thickets, 1400–3500 m. Voucher: Tweedie EM (1976) (REF). Native distribution range: Bioko, Cameroon to Ethiopia and S Africa.

***Parietariadebilis* G.Forst.** – Habit: Herb. Habitat: Upper forest margins, 2510–4200 m. Vouchers: Hedberg O 925 (EA), Taylor G 3541, 3699 (EA). Native distribution range: Old World.

***Pileajohnstonii* Oliv.** – Habit: Herb. Habitat: Forest, 1680–2910 m. Vouchers: Tweedie EM 3316 (K), 3332 (University of Alberta Museums), Snowden JD 945 (K). Native distribution range: SE Ethiopia to E Zimbabwe.

***Pilearivularis* Wedd.** – Habit: Herb. Habitat: Montane forest, 1485–3100 m. Voucher: Hedberg O (UPS). Native distribution range: Nigeria to Ethiopia and S Africa, Comoros, Madagascar.

***Pouzolziaparasitica* Schweinf.** – Habit: Herb. Habitat: Montane moist forest, grasssland, 900–2400 m. Voucher: Dawnskins 782 (EA). Native distribution range: Central America to Bolivia, Tropical & S Africa, SW Arabian Peninsula.

***Scepocarpushypselodendron* (Hochst. ex A.Rich.) T.Well & A.K.Monro** – Habit: Climbing shrub or liana. Habitat: Woodland, moist grassland, forest, upper forest edge, 1750–3150 m. Vouchers: Tweedie EM 1557 (K), Rono et al. SAJIT-PR 0227 (EA, HIB). Native distribution range: Congo to Ethiopia and S Tropical Africa.

***Urticamassaica* Mildbr.** – Habit: Herb. Habitat: Montane forest, 2000–3400 m. Voucher: Rono et al. SAJIT-PR 0269 (EA, HIB). Native distribution range: E Central & E Tropical Africa.

#### F126. VERBENACEAE

4 Genera, 8 species

**^EX^*Lantanatrifolia* L.** – Habit: Shrub or sub-shrubby herb. Habitat: Wet savanna, woodland, 1750–2400 m. Vouchers: Jackson THE 360 (EA), Stein W Bie (UPS), Rono et al. SAJIT-PR 0202 (EA, HIB). Native distribution range: Mexico to Tropical America.

***Lantanaukambensis* (Vatke) Verdc.** – Habit: Sub-shrubby herb. Habitat: Forest, wooded grassland, open woodland, 1750–2250 m. Voucher: Tweedie 1078 (EA). Native distribution range: Tropical Africa.

***Lippiaabyssinica* (Otto & A.Dietr.) Cufod.** – Habit: Shrub or subshrub. Habitat: Forest, grassland, woodland, 1950–2250 m. Voucher: Lugard 109 (EA). Native distribution range: NE & E Tropical Africa to Angola.

***Lippiajavanica* Spreng.** – Habit: Shrub. Habitat: Rocky woodland, forest, plantations, 1300–2350 m. Vouchers: Chandler 1007 (EA), Wood 433 (EA), SCL 36 (EA), Padwa JH 5 (EA), Hedberg O 1059 (EA), Webster MVB 8964 (EA), Dummer RA 3736 (EA, US). Native distribution range: Ethiopia to S Africa.

***Lippiawoodii* Moldenke** – Habit: Herb or subshrub. Habitat: Burnt grassland, open woodland, wet savanna, 1550–2280 m. Vouchers: Holm Å 32 (S), Hedberg O (UPS). Native distribution range: South Sudan to South Africa.

***Privacurtisiae* Kobuski** – Habit: Herb. Habitat: Forest, grassland, 1750–2250 m. Voucher: Tweedie EM (1976) (REF). Native distribution range: NE & E Tropical Africa to Rwanda.

**^EX^*Verbenabrasiliensis* Vell.** – Habit: Herb. Habitat: Bushland, grassland, roadside, 1750–2350 m. Voucher: Rono et al. SAJIT-PR 0201 (EA, HIB). Native distribution range: Brazil to Chile.

***Verbenaofficinalis* L.** – Habit: Herb. Habitat: Grassland, bushland, woodland, 1500–2100 m. Vouchers: Tweedie 1529 (EA). Native distribution range: Old World to Australia

#### F127. VIBURNACEAE

1 Genus, 1 species

***Sambucusafricana* Standl.** – Habit: Shrubby herb. Habitat: Bamboo zones, montane rain-forest tracks, forest floor, upland grassland, 1750–3370 m. Voucher: Major EJ; Lugard C 427 (K), Elliot GF 12, 177 (K), Snowden JD 908 (EA), Beentje HJ 1984 (EA), Hedberg O 173 (EA). Native distribution range: Madeira, NW Africa, Europe to S Turkmenistan.

#### F128. VIOLACEAE

1 Genus, 2 species

***Violaabyssinica* Steud. ex Oliv.** – Habit: Herb. Habitat: Upland forest, grassland, bushland, bamboo thickets, 1600–3740 m. Vouchers: Bridson D 63 (BR), Tweedie EM 1275, 815 (K), Rono et al. SAJIT-PR 0172 (EA, HIB). Native distribution range: Nigeria to Ethiopia and South Africa, Madagascar.

***Violaeminii* R.E.Fr.** – Habit: Herb. Habitat: Upland forest margin, grassland, moor, bamboo thickets, 2100–4050 m. Vouchers: Tweedie 1280 (K), Wesche K 1263 (EA), Granvik H 152 (S), Thorold CA 2760, 2740 (EA), Hedberg O 4556 (EA), Irwin PH 373 (EA), Bush RZ 242 (EA), Tweedie DR 5440 (EA), Bickford N 5 (EA), Dummer RA 3502 (UPS), Synge PM S 1883 (WAG), Rono et al. SAJIT-PR 0131 (EA, HIB). Native distribution range: South Sudan to Burundi and Tanzania.

#### F129. VITACEAE

5 Genera, 13 species

***Ampelocissusafricana* (Lour.) Merr.** – Habit: Liana. Habitat: Seasonal swampy grassland, woodland, riverine forest, 1800–2160 m. Voucher: Padwa JH 20 (EA). Native distribution range: Tropical Africa to Botswana.

***Cayratiagracilis* (Guill. & Perr.) Suess.** – Habit: Climber. Habitat: Wet savanna, 1750–2250 m. Voucher: Tweedie EM (1976) (REF). Native distribution range: Tropical & S Africa, Yemen.

***Cissuspetiolata* Hook.f.** – Habit: Liana. Habitat: Forest edge, riverine forest, rocky grounds, 790–1770 m. Voucher: Padwa JH 37 (EA). Native distribution range: Tropical Africa.

***Cyphostemmabambuseti* (Gilg & M.Brandt) Desc. ex Wild & R.B.Drumm.** – Habit: Climber. Habitat: Forest, bamboo forest, 1800–2100 m. Vouchers: Jackson THE 384 (EA), Lugard 343 (EA). Native distribution range: South Sudan to N Zambia.

***Cyphostemmacyphopetalum* (Fresen.) Desc. ex Wild & R.B.Drumm.** – Habit: Climbing herb. Habitat: Bushlands, evergreen forest, 470–2450 m. Voucher: Rono et al. SAJIT-PR 0086 (EA, HIB). Native distribution range: Cameroon to Eritrea and Zambia.

**Cyphostemmacyphopetalumvar.nodiglandulosum (T.C.E.Fr.) Verdc.** – Habit: Climbing herb. Habitat: Wet savanna and woodland, 1750–2400 m. Voucher: Tweedie EM (1976) (REF). Native distribution range: Kenya to N Tanzania.

***Cyphostemmaheterotrichum* (Gilg & R.E.Fr.) Desc. ex Wild & R.B.Drumm.** – Habit: Herb. Habitat: Roadsides, forest, bushland, old cultivations, burnt grassland, 1600–2300 m. Voucher: Tweedie 1123 (EA). Native distribution range: Kenya to Zimbabwe.

***Cyphostemmajunceum* (Webb) Desc. ex Wild & R.B.Drumm.** – Habit: Herb. Habitat: Burnt grassland, swamp edge, bushland, 1650–2280 m. Vouchers: Snowden JD 1050 (EA), Padwa JH 8 (EA). Native distribution range: Tropical Africa.

**^EX^Cyphostemmajunceumsubsp.jatrophoides (Baker) Verdc.** – Habit: Herb. Habitat: Wet savanna, 1750–2250 m. Voucher: Agnew ADQ (2013) (REF). Native distribution range: W Tropical Africa to Angola and Tanzania.

***Cyphostemmakilimandscharicum* (Gilg) Desc. ex Wild & R.B.Drumm.** – Habit: Climber herb. Habitat: Miost forest, 1600–3040 m. Voucher: Hedberg O (UPS). Native distribution range: SW Ethiopia to Zimbabwe.

***Cyphostemmaserpens* (Hochst. ex A.Rich.) Desc.** – Habit: Climbing herb. Habitat: Wet savanna, 1750–2250 m. Voucher: Tweedie, E.M 1122 (K). Native distribution range: NE & E Tropical Africa to Burundi.

***Cyphostemmaukerewense* (Gilg) Desc.** – Habit: Climbing or prostrate herb. Habitat: grassland, secondary forest, 1110–2520 m. Vouchers: Tweedie 3285 (EA), Rono et al. SAJIT-PR 0081 (EA, HIB). Native distribution range: Cameroon to South Sudan and W Tanzania.

***Rhoicissustridentata* (L.f.) Wild & R.B.Drumm** – Habit: Shrub or treelet or woody climber. Habitat: Forest, grassland, thickets and scrub, forest edge, woodland, 1750–2700 m. Vouchers: Hedberg O (UPS), Lugard EJ 283 (EA), Rono et al. SAJIT-PR 0100 (EA, HIB). Native distribution range: Eritrea to S Africa, SW Arabian Peninsula.

#### F130. XIMENIACEAE

1 Genus, 1 species

***Ximeniaamericana* L.** – Habit: Shrub or small tree. Habitat: Forest, woodland, 1750–2400 m. Vouchers: Snowden JD 848 (EA), Lind EM 5212 (EA), Padwa JH 21 (EA). Native distribution range: Tropics & Subtropics.

#### F131. ZYGOPHYLLACEAE

1 Genus, 1 species

***Tribulusterrestris* L.** – Habit: Herb. Habitat: Forest, paths, wet savanna, 10–2300 m. Vouchers: Tweedie EM (1976) (REF), Agnew ADQ (2013) (REF). Native distribution range: Old World.
